# Impact of summer programmes on the outcomes of disadvantaged or ‘at risk’ young people: A systematic review

**DOI:** 10.1002/cl2.1406

**Published:** 2024-06-13

**Authors:** Daniel Muir, Cristiana Orlando, Becci Newton

**Affiliations:** ^1^ Institute of Employment Studies – Economist Function Brighton East Sussex UK

**Keywords:** crime and justice outcomes, disadvantaged youth, education, employment, meta‐analysis, summer programmes

## Abstract

**Review Rationale and Context:**

Many intervention studies of summer programmes examine their impact on employment and education outcomes, however there is growing interest in their effect on young people's offending outcomes. Evidence on summer employment programmes shows promise on this but has not yet been synthesised. This report fills this evidence gap through a systematic review and meta‐analysis, covering summer education and summer employment programmes as their contexts and mechanisms are often similar.

**Research Objective:**

The objective is to provide evidence on the extent to which summer programmes impact the outcomes of disadvantaged or ‘at risk’ young people.

**Methods:**

The review employs mixed methods: we synthesise quantitative information estimating the impact of summer programme allocation/participation across the outcome domains through meta‐analysis using the random‐effects model; and we synthesise qualitative information relating to contexts, features, mechanisms and implementation issues through thematic synthesis. Literature searches were largely conducted in January 2023. Databases searched include: Scopus; PsychInfo; ERIC; the YFF‐EGM; EEF's and TASO's toolkits; RAND's summer programmes evidence review; key academic journals; and Google Scholar. The review employed PICOSS eligibility criteria: the *population* was disadvantaged or ‘at risk’ young people aged 10–25; *interventions* were either summer education or employment programmes; a valid *comparison* group that did not experience a summer programme was required; studies had to estimate the summer programme's impact on violence and offending, education, employment, socio‐emotional and/or health *outcomes*; eligible *study designs* were experimental and quasi‐experimental; eligible *settings* were high‐income countries. Other eligibility criteria included publication in English, between 2012 and 2022. Process/qualitative evaluations associated with eligible impact studies or of UK‐based interventions were also included; the latter given the interests of the sponsors. We used standard methodological procedures expected by The Campbell Collaboration. The search identified 68 eligible studies; with 41 eligible for meta‐analysis. Forty‐nine studies evaluated 36 summer education programmes, and 19 studies evaluated six summer employment programmes. The number of participants within these studies ranged from less than 100 to nearly 300,000. The PICOSS criteria affects the external applicability of the body of evidence – allowances made regarding study design to prioritise evidence on UK‐based interventions limits our ability to assess impact for some interventions. The risk of bias assessment categorised approximately 75% of the impact evaluations as low quality, due to attrition, losses to follow up, interventions having low take‐up rates, or where allocation might introduce selection bias. As such, intention‐to‐treat analyses are prioritised. The quality assessment rated 93% of qualitative studies as low quality often due to not employing rigorous qualitative methodologies. These results highlight the need to improve the evidence.

**Results and Conclusions:**

*Quantitative synthesis* The quantitative synthesis examined impact estimates across 34 outcomes, through meta‐analysis (22) or in narrative form (12). We summarise below the findings where meta‐analysis was possible, along with the researchers' judgement of the security of the findings (high, moderate or low). This was based on the number and study‐design quality of studies evaluating the outcome; the consistency of findings; the similarity in specific outcome measures used; and any other specific issues which might affect our confidence in the summary findings.

Below we summarise the findings from the meta‐analyses conducted to assess the impact of allocation to/participation in summer education and employment programmes (findings in relation to other outcomes are also discussed in the main body, but due to the low number of studies evaluating these, meta‐analysis was not performed). We only cover the pooled results for the two programme types where there are not clear differences in findings between summer education and summer employment programmes, so as to avoid potentially attributing any impact to both summer programme types when this is not the case. We list the outcome measure, the average effect size type (i.e., whether a standardised mean difference (SMD) or log odds ratio), which programme type the finding is in relation to and then the average effect size along with its 95% confidence interval and the interpretation of the finding, that is, whether there appears to be a significant impact and in which direction (positive or negative, clarifying instances where a negative impact is beneficial). In some instances there may be a discrepancy between the 95% confidence interval and whether we determine there to be a significant impact, which will be due to the specifics of the process for constructing the effect sizes used in the meta‐analysis. We then list the *I* ^2^ statistic and the *p*‐value from the homogeneity test as indications of the presence of heterogeneity. As the sample size used in the analysis are often small and the homogeneity test is known to be under‐powered with small sample sizes, it may not detect statistically significant heterogeneity when it is in fact present. As such, a 90% confidence level threshold should generally be used when interpreting this with regard to the meta‐analyses below. The presence of effect size heterogeneity affects the extent to which the average effects size is applicable to all interventions of that summer programme type. We also provide an assessment of the relative confidence we have in the generalisability of the overall finding (low, moderate or high) – some of the overall findings are based on a small sample of studies, the studies evaluating the outcome may be of low quality, there may be wide variation in findings among the studies evaluating the outcome, or there may be specific aspects of the impact estimates included or the effect sizes constructed that affect the generalisability of the headline finding. These issues are detailed in full in the main body of the review.

– Engagement with/participation in/enjoyment of education (SMD):
  ∘ Summer education programmes: +0.12 (+0.03, +0.20); positive impact; *I* ^2^ = 48.76%, *p* = 0.10; moderate confidence.
– Secondary education attendance (SMD):
  ∘ Summer education programmes: +0.26 (+0.08, +0.44); positive impact; *I* ^2^ = N/A; *p* = N/A; low confidence.
  ∘ Summer employment programmes: +0.02 (−0.03, +0.07); no impact; *I* ^2^ = 69.98%; *p* = 0.03; low confidence.
– Passing tests (log OR):
  ∘ Summer education programmes: +0.41 (−0.13, +0.96); no impact; *I* ^2^ = 95.05%; *p* = 0.00; low confidence.
  ∘ Summer employment programmes: +0.02 (+0.00, +0.04); positive impact; *I* ^2^ = 0.01%; *p* = 0.33; low confidence.
– Reading test scores (SMD):
  ∘ Summer education programmes: +0.01 (−0.04, +0.05); no impact; *I* ^2^ = 0.40%; *p* = 0.48; high confidence.
– English test scores (SMD):
  ∘ Summer education programmes: +0.07 (+0.00, +0.13); positive impact; *I* ^2^ = 27.17%; *p* = 0.33; moderate confidence.
  ∘ Summer employment programmes: −0.03 (−0.05, −0.01); negative impact; *I* ^2^ = 0.00%; *p* = 0.76; low confidence.
– Mathematics test scores (SMD):
  ∘ All summer programmes: +0.09 (−0.06, +0.25); no impact; *I* ^2^ = 94.53%; *p* = 0.00; high confidence.
  ∘ Summer education programmes: +0.14 (−0.09, +0.36); no impact; *I* ^2^ = 94.15%; *p* = 0.00; moderate confidence.
  ∘ Summer employment programmes: +0.00 (−0.04, +0.05); no impact; *I* ^2^ = 0.04%; *p* = 0.92; moderate confidence.
– Overall test scores (SMD):
  ∘ Summer employment programmes: −0.01 (−0.08, +0.05); no impact; *I* ^2^ = 32.39%; *p* = 0.20; high confidence.
– All test scores (SMD):
  ∘ Summer education programmes: +0.14 (+0.00, +0.27); positive impact; *I* ^2^ = 91.07%; *p* = 0.00; moderate confidence.
  ∘ Summer employment programmes: −0.01 (−0.04, +0.01); no impact; *I* ^2^ = 0.06%; *p* = 0.73; high confidence.
– Negative behavioural outcomes (log OR):
  ∘ Summer education programmes: −1.55 (−3.14, +0.03); negative impact; *I* ^2^ = N/A; *p* = N/A; low confidence.
  ∘ Summer employment programmes: −0.07 (−0.33, +0.18); no impact; *I* ^2^ = 88.17%; *p* = 0.00; moderate confidence.
– Progression to HE (log OR):
  ∘ All summer programmes: +0.24 (−0.04, +0.52); no impact; *I* ^2^ = 97.37%; *p* = 0.00; low confidence.
  ∘ Summer education programmes: +0.32 (−0.12, +0.76); no impact; *I* ^2^ = 96.58%; *p* = 0.00; low confidence.
  ∘ Summer employment programmes: +0.10 (−0.07, +0.26); no impact; *I* ^2^ = 76.61%; *p* = 0.02; moderate confidence.
– Complete HE (log OR):
  ∘ Summer education programmes: +0.38 (+0.15, +0.62); positive impact; *I* ^2^ = 52.52%; *p* = 0.06; high confidence.
  ∘ Summer employment programmes: +0.07 (−0.19, +0.33); no impact; *I* ^2^ = 70.54%; *p* = 0.07; moderate confidence.
– Entry to employment, short‐term (log OR):
  ∘ Summer employment programmes: −0.19 (−0.45, +0.08); no impact; *I* ^2^ = 87.81%; *p* = 0.00; low confidence.
  ∘ Entry to employment, full period (log OR)
  ∘ Summer employment programmes: −0.15 (−0.35, +0.05); no impact; *I* ^2^ = 78.88%; *p* = 0.00; low confidence.
– Likelihood of having a criminal justice outcome (log OR):
  ∘ Summer employment programmes: −0.05 (−0.15, +0.05); no impact; *I* ^2^ = 0.00%; *p* = 0.76; low confidence.
– Likelihood of having a drug‐related criminal justice outcome (log OR):
  ∘ Summer employment programmes: +0.16 (−0.57, +0.89); no impact; *I* ^2^ = 65.97%; *p* = 0.09; low confidence.
– Likelihood of having a violence‐related criminal justice outcome (log OR):
  ∘ Summer employment programmes: +0.03 (−0.02, +0.08); no impact; *I* ^2^ = 0.00%; *p* = 0.22; moderate confidence.
– Likelihood of having a property‐related criminal justice outcome (log OR):
  ∘ Summer employment programmes: +0.09 (−0.17, +0.34); no impact; *I* ^2^ = 45.01%; *p* = 0.18; low confidence.
– Number of criminal justice outcomes, during programme (SMD):
  ∘ Summer employment programmes: −0.01 (−0.03, +0.00); no impact; *I* ^2^ = 2.17%; *p* = 0.31; low confidence.
– Number of criminal justice outcomes, post‐programme (SMD):
  ∘ Summer employment programmes: −0.01 (−0.03, +0.00); no impact; *I* ^2^ = 23.57%; *p* = 0.37; low confidence.
– Number of drug‐related criminal justice outcomes, post‐programme (SMD):
  ∘ Summer employment programmes: −0.01 (−0.06, +0.06); no impact; *I* ^2^ = 55.19%; *p* = 0.14; moderate confidence.
– Number of violence‐related criminal justice outcomes, post‐programme (SMD):
  ∘ Summer employment programmes: −0.02 (−0.08, +0.03); no impact; *I* ^2^ = 44.48%; *p* = 0.18; low confidence.
– Number of property‐related criminal justice outcomes, post‐programme (SMD):
  ∘ Summer employment programmes: −0.02 (−0.10, +0.05); no impact; *I* ^2^ = 64.93%; *p* = 0.09; low confidence.

We re‐express instances of significant impact by programme type where we have moderate or high confidence in the security of findings by translating this to a form used by one of the studies, to aid understanding of the findings. Allocation to a summer education programme results in approximately 60% of individuals moving from never reading for fun to doing so once or twice a month (engagement in/participation in/enjoyment of education), and an increase in the English Grade Point Average of 0.08. Participation in a summer education programme results in an increase in overall Grade Point Average of 0.14 and increases the likelihood of completing higher education by 1.5 times. Signs are positive for the effectiveness of summer education programmes in achieving some of the education outcomes considered (particularly on test scores (when pooled across types), completion of higher education and STEM‐related higher education outcomes), but the evidence on which overall findings are based is often weak. Summer employment programmes appear to have a limited impact on employment outcomes, if anything, a negative impact on the likelihood of entering employment outside of employment related to the programme. The evidence base for impacts of summer employment programmes on young people's violence and offending type outcomes is currently limited – where impact is detected this largely results in substantial reductions in criminal justice outcomes, but the variation in findings across and within studies affects our ability to make any overarching assertions with confidence. In understanding the effectiveness of summer programmes, the order of outcomes also requires consideration – entries into education from a summer employment programme might be beneficial if this leads towards better quality employment in the future and a reduced propensity of criminal justice outcomes.

**Qualitative Synthesis:**

Various shared features among different summer education programmes emerged from the review, allowing us to cluster specific types of these interventions which then aided the structuring of the thematic synthesis. The three distinct clusters for summer education programmes were: catch‐up programmes addressing attainment gaps, raising aspirations programmes inspiring young people to pursue the next stage of their education or career, and transition support programmes facilitating smooth transitions between educational levels. Depending on their aim, summer education programme tend to provide a combination of: additional instruction on core subjects (e.g., English, mathematics); academic classes including to enhance specialist subject knowledge (e.g., STEM‐related); homework help; coaching and mentoring; arts and recreation electives; and social and enrichment activities. Summer employment programmes provide paid work placements or subsidised jobs typically in entry‐level roles mostly in the third and public sectors, with some summer employment programmes also providing placements in the private sector. They usually include components of pre‐work training and employability skills, coaching and mentoring. There are a number of mechanisms which act as facilitators or barriers to engagement in summer programmes. These include tailoring the summer programme to each young person and individualised attention; the presence of well‐prepared staff who provide effective academic/workplace and socio‐emotional support; incentives of a monetary (e.g., stipends and wages) or non‐monetary (e.g., free transport and meals) nature; recruitment strategies, which are effective at identifying, targeting and engaging participants who can most benefit from the intervention; partnerships, with key actors who can help facilitate referrals and recruitment, such as schools, community action and workforce development agencies; format, including providing social activities and opportunities to support the formation of connections with peers; integration into the workplace, through pre‐placement engagement, such as through orientation days, pre‐work skills training, job fairs, and interactions with employers ahead of the beginning of the summer programme; and skill acquisition, such as improvements in social skills. In terms of the causal processes which lead from engagement in a summer programme to outcomes, these include: skill acquisition, including academic, social, emotional, and life skills; positive relationships with peers, including with older students as mentors in summer education programmes; personalised and positive relationships with staff; location, including accessibility and creating familiar environments; creating connections between the summer education programme and the students' learning at home to maintain continuity and reinforce learning; and providing purposeful and meaningful work through summer employment programmes (potentially facilitated through the provision of financial and/or non‐financial incentives), which makes participants more likely to see the importance of education in achieving their life goals and this leads to raised aspirations. It is important to note that no single element of a summer programme can be identified as generating the causal process for impact, and impact results rather from a combination of elements. Finally, we investigated strengths and weaknesses in summer programmes at both the design and implementation stages. In summer education programmes, design strengths include interactive and alternative learning modes; iterative and progressive content building; incorporating confidence building activities; careful lesson planning; and teacher support which is tailored to each student. Design weaknesses include insufficient funding or poor funding governance (e.g., delays to funding); limited reach of the target population; and inadequate allocation of teacher and pupil groups (i.e., misalignment between the education stage of the pupils and the content taught by staff). Implementation strengths include clear programme delivery guidance and good governance; high quality academic instruction; mentoring support; and strong partnerships. Implementation weaknesses include insufficient planning and lead in time; recruitment challenges; and variability in teaching quality. In summer employment programmes, design strengths include use of employer orientation materials and supervisor handbooks; careful consideration of programme staff roles; a wide range of job opportunities; and building a network of engaged employers. Design weaknesses are uncertainty over funding and budget agreements; variation in delivery and quality of training between providers; challenges in recruitment of employers; and caseload size and management. Implementation strengths include effective job matching; supportive relationships with supervisors; pre‐work training; and mitigating attrition (e.g., striving to increase take up of the intervention among the treatment group). Implementation weaknesses are insufficient monitors for the number of participants, and challenges around employer availability.

## 1 PLAIN LANGUAGE SUMMARY

Do summer education and employment programmes make a difference to young people who are disadvantaged or at risk of achieving poor outcomes, and if so, to what extent and how do they do this?

### 1.1 Key messages

▪ The evidence suggests that summer education programmes lead to an improvement in some education outcomes among the disadvantaged or ‘at risk’ young people involved.
▪ The evidence suggests that summer employment programmes have a limited or no impact on the employment or education outcomes of disadvantaged or ‘at risk’ young people, although there appears to be an impact on ‘softer’ outcomes, such as job readiness or socio‐emotional skills, which might lead to improvements in other outcomes over the longer‐term, and where impacts on violence and offending outcomes are identified these are substantial.
▪ There are issues with the quality of evidence that this review draws upon, which affects our confidence in the findings. Additionally, there are some types of outcomes, including violence and offending, health, and socio‐emotional, that are not studied as extensively as education or employment related outcomes. These need to be studied further to fully understand the impact summer programmes have.

### 1.2 What is a summer programme?

Summer programmes take place in the long vacation between academic years or after the final academic year and are additional to the usual curriculum. This review centres on two types of summer programme): summer *education* programmes which involve educational instruction; and summer *employment* programmes that include a fixed‐term job placement.

### 1.3 How might summer programmes benefit disadvantage or ‘at risk’ young people?

Summer programmes might improve the outcomes of disadvantaged or ‘at risk’ young people by:

▪ offering them provision that is alternative and extra to the usual curriculum for their age and stage;
▪ providing them with productive activities, such as coursework or an internship;
▪ distracting them from unproductive activities, such as antisocial behaviour or criminal activity;
▪ raising their aspirations to, for example, progress to higher education;
▪ supporting their transition from one education stage to another; and
▪ teaching them new hard and soft skills.

### 1.4 What did we want to find out?

We wanted to understand whether summer programmes lead to improvements for disadvantaged or ‘at risk’ young people on five different types of outcome, namely education, employment, violence and offending, health and socio‐emotional outcomes, and if so, how, by identifying key features of successful summer programmes that lead to these outcomes.

### 1.5 What did we do?

We searched for studies that examined the impact of summer programmes on the outcomes of disadvantaged young people, and other studies associated with these that examined what affected the impact the summer programme had. We also searched for the latter study type of summer programmes implemented in the UK, regardless of whether there was a study that examined the impact of the summer programme. We compared and summarised the results of the studies and rated our confidence in the evidence, based on factors such as study methods and sample sizes.

### 1.6 What did we find?

We found 68 studies that engaged between less than 100 to nearly 300,000 individuals in a summer programme. Forty‐one of these studies estimated the impact of the summer programme on the young person's outcomes.

We have some degree of confidence that summer education programmes appear to have had no impact on reading and mathematics test scores, although there are significant differences in this finding for mathematics test scores between the studies included. We have some degree of confidence that summer education programmes appear to have had a beneficial impact on: English and all forms of test scores (although there are significant differences in this finding for all forms of test scores between the studies included); the likelihood of completing higher education (although there are significant differences in this finding between the studies included); and STEM‐related higher education outcomes.

We have some degree of confidence that summer employment programmes appear to have had no impact on: mathematics, overall, and all forms of test scores; the likelihood of having negative behavioural outcomes at school such as a suspension (although there are significant differences in this finding for mathematics test scores between the studies included); and the likelihood of progressing to, or completing higher education (although there are significant differences in this finding for mathematics test scores between the studies included). Where summer education programmes appear to have had a beneficial or detrimental impact on outcomes, due to the evidence available we cannot be certain about the finding.

### 1.7 What are the limitations of the evidence?

Several outcomes, including violence and offending, health and socio‐emotional outcomes, are only evaluated by a small number of studies. Additionally, outcomes are generally only measured over a relatively short time period whereas data over a longer time period is needed to understand the longer‐term effects of summer programmes. Lastly, some aspects of the quality of the evidence are limited: studies estimating the impact of summer programmes often suffer from individuals allocated to participate in the summer programme not taking up their place, which could affect the reliability of the estimates of impact; studies evaluating how summer programmes affect young people's outcomes often only do so informally as part of a study estimating their impact.

### 1.8 How up to date is this evidence?

The evidence covers from 2012 up to the end of 2022.

## 2 BACKGROUND

Many intervention studies of summer programmes examine their impact on the outcome domains of employment (e.g., Alam et al., 2013; Valentine et al., 2017) and education (e.g., Leos‐Urbel, 2014; Schwartz et al., 2020). However, there is growing interest in the impact of participating in summer programmes on the reduction of anti‐social behaviour, including young people's violence and criminal activity. Evaluating a Boston‐based summer employment programme using a randomised controlled trial (RCT), Modestino (2019b) observed a reduction in violent‐crime and property‐crime arrests among programme participants – a pattern that persisted up to 17 months after participation. Furthermore, programme participants showed significant increases to community engagement, social skills, job readiness, and future intentions to work (Modestino & Paulsen, 2019a). This reduction in criminal behaviour was also observed in other summer employment programmes implemented in other cities across the United States (Davis & Heller, 2020; Heller, 2014, 2022). The initial evidence base on summer programmes offers some promise in terms of improving young people's outcomes and life chances. However, given the lack of a systematic review that estimates the extent of this, we cannot yet fully assert this. This current review seeks to fill this evidence gap.

In the body of available literature, there is no common definition of what constitutes a ‘summer programme’; most authors also do not propose a definition. However, there are some common groupings of summer programmes. There are numerous summer ‘education’ programmes – those that incorporate some form of academic instruction or support. Within this grouping there is significant heterogeneity in the type of intervention. Some summer education programmes are focused on ‘catch‐up’ for students who are falling behind their peers, for instance New York's Summer Success Academy (see Mariano & Martorell, 2013). Others aim to support young people through transitions between stages of education – bridge programmes for incoming college students are particularly common in the US (e.g., Barnett et al., 2012), while outreach programmes, commonly run by universities in the UK such as the Aimhigher West Midlands UniConnect programme (see Horton & Hilton, 2020), look to raise the aspirations of school leavers and encourage entry to higher education.

‘Employment’ summer programmes instead focus on transitions to the labour market, typically through some form of job placement often alongside wider employment support including careers guidance and skills development – examples include the Boston Summer Youth Employment Program (see Modestino & Paulsen, 2019a; Modestino, 2019b) and One Summer Chicago (see Davis & Heller, 2020; Heller, 2014, 2022).

While there is wide variation in the features of different types of summer programmes, the literature identifies some areas where there are commonalities within and across summer programme types, namely:

- the period in which the programme is delivered;
- the programme duration;
- the type of organisation delivering the programme;
- the programme's participants;
- the types of tasks and activities included in the programme; and
- the programme's target outcomes (primarily short‐term).

For the purposes of this review, the authors considered these features to construct operational definitions for different types of summer programmes.

### 2.1 Policy relevance

A preliminary literature review to scope evaluations of summer programmes identified that they may result in outcomes across the following domains:

- education (e.g., school participation, school completion, academic attainment, school readiness);
- employment (e.g., job readiness, soft skills, unemployment, job search skills);
- violence and offending (e.g., likelihood of reoffending, likelihood of involvement in illegal activity);
- socio‐emotional (e.g., resilience, confidence, social skills, community engagement, emotion management); and
- health (e.g., understanding of health issues, such as substance abuse, physical activity, nutrition, and condition management).

This review is sponsored by Youth Endowment Fund (YEF, 2022) and Youth Futures Foundation (YFF). It is therefore anchored on the Outcomes Framework of YEF – which focuses on reducing offending among young people and takes account of the outcomes prioritised by YFF – which focus on supporting better employment for young people.

Given the early evidence showing reductions in criminal activity due to young people's participation in summer employment programmes (see Davis & Heller, 2020; Heller, 2014, 2022; Modestino & Paulsen, 2019a; Modestino, 2019b), a systematic review and meta‐analysis was an appropriate next step to be able to verify the positive impact observed in previous studies and to estimate the magnitude of this positive impact (if it is found to be present). However, we expanded the coverage of this systematic review to include summer education programmes (e.g., summer schools, summer learning programmes), as well as to look at a broader set of outcomes across the domains outlined above.

The rationale to do this is the inverse relationship between educational outcomes and violence among young people, which has been extensively documented (and is neatly summarised in Bushman et al., 2016). Given the currently mixed evidence regarding the effect of summer education programmes and summer work programmes on educational outcomes (see Barnett et al., 2012; Gonzalez Quiroz & Garza, 2018; Kallison & Stader, 2012; Lynch et al., 2021; Sablan, 2014; Terzian et al., 2009), this review presents an opportunity to examine their impact – though indirect – on violence among young people. Education and employment are linked and interact in deterring the production of antisocial behaviour (Lochner, 2004), a relationship acknowledged by the Outcomes Frameworks of both YEF and YFF; the expert reference group for the development of YEF's Outcomes Framework acknowledged that engagement in education is one of the most important factors in protecting young people from crime and violence. Further, young people who become involved in the youth justice system are also disproportionately likely to have mental health problems including anxiety and depression, and there is clear evidence of the links between work and health and socio‐emotional wellbeing (Waddell & Burton, 2006), and education and health and socio‐emotional wellbeing (Brooks, 2014; Department of Health, 2008). In light of this interrelatedness, considering education and employment‐oriented summer programmes in this systematic review, and considering their effects across a wide range of highly interrelated outcome domains through direct, moderated and indirect effects is pertinent. It is also consistent with contemporary theories of the development of young people (Lerner & Castellino, 2002; YFF, 2021).

Disadvantaged young people are those who are at risk of poorer outcomes, including educational, economic, health, and social outcomes, as a result of one or more adverse situational and behavioural factors faced in childhood and/or the transition to adulthood. Situational factors that increase risks include race and ethnicity, low socioeconomic status, low parental attainment, being in care or being a care giver, and/or having disabilities or health conditions including mental health conditions. Behavioural factors that increase risk include involvement in crime or anti‐social behaviour, a low level or lack of parental support, truanting and being excluded from school, teenage pregnancy and poor school performance in early years (Kritikos & Ching, 2005; Machin, 2006; Pring et al., 2009; Rathbone/Nuffield Foundation, 2008).

Summer ‘education’ and ‘employment’ summer programmes warrant considering together within this review as the contexts of and mechanisms employed by these programmes are often similar, and while there may be differences in the proximal outcomes they typically aim to achieve (summer education programmes are typically focused on educational attainment, and have successful completion of and transitions between stages as their primary outcomes while summer employment programmes are typically focused on entry to employment and labour market outcomes), as discussed these outcomes are highly interrelated, with programmes having a range of indirect effects on participants. Additionally, any variation in the outcomes typically achieved by different summer programme types is important for policymakers to be aware of.

### 2.2 How the intervention might work

#### 2.2.1 Rationale for delivery and key assumptions

Summer programmes aim to improve the outcomes of young people through offering them alternative and extra provision; that is, additional to the usual curriculum for their age and stage (which may be considered ‘service as usual’) (Barnett et al., 2012; EEF, n.d.; Heller, 2022; Hutchinson et al., 2001; Modestino, 2019b; Tarling & Adams, 2012). The intention is to avoid interference with the standard curriculum and to build additional support to improve outcomes in ‘service as usual’ including progression through education as well as into the labour market. An assumption is that targeted young people will find programmes attractive and engage in them, with a further assumption that they will be supported by their families and/or carers to do so.

While the characteristics of the target group may vary – from those with offending histories or at risk of these (Modestino, 2019b; Tarling & Adams, 2012), to those with low attendance and low attainment (Hutchinson et al., 2001) and to what is sometimes described as the grey or middle group who fail to grab attention but also are at risk of poorer outcomes due to not having firm ambitions (Barnett et al., 2012) – there is also recognition that the selected target group is not engaging with ‘service as usual’ as effectively as other groups, or not engaging at all. Therefore, the assumption is that an alternative approach is required to foster more positive engagement or re‐engagement to achieve outcomes.

Summer education programmes may focus on ‘catch up’ with aims of closing the attainment gap for disadvantaged learners (EEF, n.d.; Tarling & Adams, 2012) or be aimed to support transitions between education phases (Hutchinson et al., 2001) and to accelerate achievement in the next education phase (Barnett et al., 2012). They may offer learning in an alternative format (Tarling & Adams, 2012), as part of smaller groups or with more staff support which can lead towards better attainment (EEF, n.d.). The underlying assumption, as identified by the Education Endowment Foundation (EEF) toolkit (n.d.), is simply that more time in school/education leads to better educational outcomes.

Summer employment programmes may share similar aims. For example, Alam et al. (2013) explore summer employment programmes that aim to support and improve transitions to the next stage of education. The assumption is that the job placement creates an early insight into the labour market that builds ambition. This, in turn, increases understanding of the importance of educational credentials to good quality work. As a result, motivation for achieving in the next phase of education is increased. Summer employment programmes may also aim to divert or distract those who have been involved in or are at risk of offending away from harmful or unproductive activities (Leos‐Urbel, 2014; Modestino, 2019b). The underlying assumption is that through providing alternative uses for the time over summer that otherwise would be unallocated, this reduces the risk of that time being used for criminal or anti‐social activity.

#### 2.2.2 Mechanisms

There are a number of mechanisms through which summer programmes work, with many of these shared across job and education programmes. This stems in large part from common intermediate outcomes relating to personal and social development, and vocational and applied skill acquisition. For example, Modestino (2019b) identifies a mechanism through building aspiration, self‐belief, emotion control and a longer‐term work ambition. The summer employment programme encourages young people to improve their engagement with education as a precursor to achieving newly found higher quality employment goals. This, in turn, leads to better attainment – which was an outcome not originally anticipated. The commonalities with the summer education programme concern the soft skill development including self‐esteem and confidence, emotion control, leadership skills, communication, problem‐solving, and responsibility and time management (Hutchinson et al., 2001; Leos‐Urbel, 2014).

A common mechanism in the summer programmes targeted at disadvantaged or ‘at risk’ young people is the opportunity to form better relationships. In summer education programmes, this can result from the group of young people formed for the programme (EEF, n.d.; Hutchinson et al., 2001). It also results where delivery teams are new to the young people. Hence, in summer education programmes that are delivered by staff who are different from those in ‘service as usual’, there is a chance to re‐set engagement with adults, which can then set the tone for the next stage of ‘service as usual’. In summer employment programmes, the adult relationship is formed with employees in the employing organisation. This, along with the employers' expectation of performance from the young person, builds responsibility, maturity and self‐esteem (Alam et al., 2013; Modestino, 2019b). Improved interpersonal relationships might also contribute towards feeling more settled thereby supporting improved wellbeing – although evidence for these outcomes is weak (Terzian et al., 2009).

In both summer employment and summer education programmes, financial incentives can be a mechanism for change (Barnett et al., 2012; Modestino, 2019b). Providing financial recognition can have an important effect on how the opportunity is valued within the young person's household – which can support engagement from families and/or carers, as well as providing a reward for the young person's time. Financial incentives may also help to alleviate financial constraints on future education, increasing investment in human capital and improving longer‐term outcomes.

Location is an important mechanism to the outcomes for some summer programmes. For summer employment programmes, young people are exposed to the world of work, and are located in an organisation for a job placement. This builds familiarity and confidence in this new setting as well as increasing expectations for conduct in an adult environment (Heller, 2022; Modestino, 2019b). Where summer education programmes support transitions to the next phase of education they may take place on the campus of that next phase. This similarly builds familiarity and confidence to be in this new environment. In these programmes, building familiarity with the campus and the services available can increase likelihood to seek out and use support services post‐transition, which provides crucial underpinning to sustaining this destination that is, reducing the likelihood of drop‐out, particularly important when transitioning to higher education. Finally, summer education programmes may be located in alternative settings, such as the outdoors, providing a different context for learning that can support young people to engage differently and to achieve, thereby building confidence for learning in the traditional classroom setting (Tarling & Adams, 2012; Terzian et al., 2009).

These are all positive causal mechanisms to the achievement of outcomes; however, some studies identify the potential for negative effects from summer programme participation resulting from some of these mechanisms. This includes, for example, Alam et al. (2013) who suggest that requiring disadvantaged young people to attend summer employment programmes at a time when their peers are at rest and on vacation can leave them exhausted and not well placed for the start of the new term. This is a risk that intuitively reads across to summer education programmes. Consequently, duration and intensity of the programmes are important contextual factors in the analysis. Alam et al. (2013) also indicate that the positive effect on attainment established by Modestino (2019b) may not result from all summer employment programmes. Rather than build motivation through understanding why education is important, the ability to earn ‘easy’ money from summer jobs may deter young people from engaging in their further studies. Quality of and safeguarding in the job placement are also a key consideration to ensure young people do not see negative consequences, such as from encountering poor social behaviour among permanent or standard employees. For economically disadvantaged young people, a further negative consequence of being part of summer employment or education programmes may be that they are unavailable for activities such as standard employment that is better paid. This may have consequences for short‐ and long‐term financial returns as well as for engagement and attrition in programmes.

#### 2.2.3 Outcomes

Considering the mechanisms through which summer programmes may affect positive outcomes over the longer‐term, summer employment programmes provide meaningful employment experiences which can provide alternative pathways for disadvantaged young people, opening up economic opportunities to them which, because of their disadvantage, may be limited outside of public interventions, relative to more advantaged young people (Modestino & Paulsen, 2019a). Summer education programmes, through the mechanisms discussed above, may lead to improved academic attainment in ensuing phases of education, which also improve future economic opportunities by increasing the individuals' skills and desirability in the labour market. As a result, both summer education and summer employment programmes can improve violence and offending outcomes – by improving the individuals' economic opportunities. This can set expectations about their future quality of life, and mean they are less likely to offend as the opportunity costs of the punishment are increased (Heller, 2014).

Wider evidence supports these causal pathways. For example, Bell et al. (2018) demonstrates the links between improved education attainment and prolonged education on criminal justice outcomes, citing research by Lochner (2004). Additionally, statistics from the UK Department for Education demonstrate the links between higher levels of education and higher skilled employment, comparing graduates and postgraduates with non‐graduate outcomes, as well as the higher propensity to be employed rather than economically inactive by the higher level of qualification attained. Further evidence is supplied by the OECD (2020) that sets out the higher likelihood of being employed by the higher level of education. In this way, education outcomes can be seen as intermediaries in the path to better employment outcomes, and better offending outcomes.

Improved economic opportunities resulting from participation in a summer programme may also affect positive health and socio‐emotional outcomes, by potentially improving nutritional choices, reducing anxiety and stress, and increasing self‐confidence and self‐worth as a result of increased financial resources. Given the interrelatedness between education, employment, violence and offending, health and socio‐emotional outcomes, intermediate improvements in outcomes within one domain, as a direct result of participation in a summer education or employment programme, is likely to result in improved outcomes across the other domains.

The points raised in this background section are not intended to be comprehensive – further assumptions, mechanisms and outcomes are likely to be identified as the literature is systematically searched.

## 3 RESEARCH OBJECTIVES

### 3.1 Research questions

Based on the findings of the preliminary literature review covering types of summer programmes, their outcomes, and the goals of the funding organisations, this review aims to answer the following research questions:

#### 3.1.1 Meta‐analysis

1. To what extent does participation in summer employment programmes:
  a. improve violence and offending outcomes?
  b. improve educational outcomes?
  c. improve employment outcomes?
  d. improve socio‐emotional outcomes?
  e. improve health outcomes?
2. To what extent does participation in summer education programmes:
  a. improve violence and offending outcomes?
  b. improve educational outcomes?
  c. improve employment outcomes?
  d. improve socio‐emotional outcomes?
  e. improve health outcomes?
3. To what extent do the outcomes achieved by summer programmes vary based on the study, participant and intervention characteristics including the racial and ethnic make‐up of participants?

#### 3.1.2 Thematic synthesis

4. What are common features of summer employment and education programmes that produce positive outcomes? Which features contribute most to the achievement of:
  a. improved violence and offending outcomes?
  b. improved educational outcomes?
  c. improved employment outcomes?
  d. improved socio‐emotional outcomes?
  e. improved health outcomes?
5. In which contexts are summer employment and education programmes most or least able to produce positive outcomes? Are any contexts more or less able to produce:
  a. improved violence and offending outcomes?
  b. improved educational outcomes?
  c. improved employment outcomes?
  d. improved socio‐emotional outcomes?
  e. improved health outcomes?
6. Which mechanisms inhibit or enable the effectiveness of summer employment and education programmes? Are any mechanisms particularly important to achieving:
  a. improved violence and offending outcomes?
  b. improved educational outcomes?
  c. improved employment outcomes?
  d. improved socio‐emotional outcomes?
  e. improved health outcomes?
7. In what ways do factors such as targeting, retention, and dropout affect the achievement of outcomes by summer employment and education programmes?

Additionally, data on the cost of delivering summer programmes, where reported on, is also to be synthesised.

#### 3.1.3 Why is this review needed in light of existing reviews?

While still limited, the evidence base for the impact of summer programmes is growing. There have been a number of intervention studies that examine the effect of summer employment programmes on antisocial behaviour among young people (e.g., Davis & Heller, 2020; Heller, 2014, 2022; Modestino & Paulsen, 2019a; Modestino, 2019b). Findings from these individual intervention studies are promising, showing a relationship between participation in summer employment programmes and reduced antisocial behaviour. However, the lack of a systematic review makes it difficult to assert this relationship exists and to estimate the extent to which positive behavioural outcomes can be attributed to participation in the programmes. This review examines the impact of summer employment programmes on other outcomes that influence young people's life chances, such as education and employment – both outcome domains that at least some of the evidence on summer employment programmes has examined – as well as violence and offending, socio‐emotional and health outcomes.

There is a well‐documented link between educational outcomes and violence among young people (Bushman et al., 2016), so it is also important to take stock of these summer programmes. EEF sponsored a systematic review of summer schools and their impact on educational outcomes among 3‐to‐18‐year‐olds for their Teaching and Learning Toolkit (EEF, n.d.). This found that summer schools have a moderate impact on educational outcomes. Other evidence of the positive impact of summer schools is also conclusive (see Cooper et al., 2000; Lauer et al., 2006). However, evaluations of other forms of education‐oriented summer programmes have yielded more mixed results (see Barnett et al., 2012; Gonzalez Quiroz & Garza, 2018; Kallison & Stader, 2012; Lynch et al., 2021; Terzian et al., 2009). The inclusion of literature on education‐oriented summer programmes offers an opportunity to clarify the currently mixed evidence regarding their impact. There is also a need to update the existing evidence in light of policy changes, such as the transition to and implementation of the Raised Participation Age (RPA) policy in England in 2012; of the 59 studies included in the EEF systematic review, only five were published since 2012, with the most recent published in 2014. Additionally, summer education programmes may affect outcomes across domains other than education, which warrants investigation. Furthermore, given the age range employed by EEF, summer schools, which constitute a large component of summer education programmes, have not been synthesised for their effects beyond the age of 18. This means current analyses do not cover any studies focused on post‐18 study which may be compensatory (catching up on what should have been achieved in compulsory schooling) or at the further or higher level.

To support the Wallace Foundation's Summer Learning Toolkit, RAND have also performed a systematic review of summer programmes, covering programmes focused on education and employment as well as wellbeing and enrichment (McCombs et al., 2019; Wallace Foundation, n.d.). The review covered only interventions from the US, and across age groups from pre‐kindergarten through to the summer before grade 12 (ages 17–18). Outcomes relating to academic achievement, academic and career attainment, engagement with schooling, social and emotional competencies, physical and mental health, and the avoidance of risky behaviour were considered, although no meta‐analyses were conducted. Similarly, the Transforming Access and Student Outcomes in Higher Education centre (TASO) has a stream relating to summer schools in their Evidence toolkit. It is based on a collection of UK interventions centred on transitions to higher education (TASO, n.d.), which finds that summer schools have a positive impact on student aspirations and attitudes. The strength of evidence is noted as ‘emerging’, that is, relatively weak, with many of the studies covered not employing robust experimental/quasi‐experimental designs.

## 4 OVERVIEW OF APPROACH

The protocol for this review (Muir et al., 2023) outlined the review's intended approach. A section below details all the deviations from this.

This systematic review and meta‐analysis are underpinned by four key stages:

1. Searching the appropriate literature through an agreed list of search terms.
2. Selecting relevant studies based on specified and agreed inclusion and exclusion criteria.
3. Extracting relevant evidence using an agreed protocol.
4. Synthesising and interpreting the evidence to inform high quality, user friendly, accessible, engaging, relevant and useful reviews.

This systematic review has examined out‐of‐school‐time programmes conducted throughout or at some point during the summer months (i.e., the period in which the long vacation takes place between academic years or after the final academic year before moving into economic activity). These programmes include summer employment programmes and summer education programmes.

The focus is how these programmes improve outcomes among young people who are disadvantaged and/or at risk of poorer outcomes in later life (‘at risk’), including educational, economic, health, and social outcomes, as a result of one or more adverse situational and behavioural factors faced in childhood or as a young adult. While the experience of even one disadvantage factor may lead to young people facing difficulties in transitioning into adulthood, disadvantage factors often interact and compound each other leading to severe adverse impacts for young people and society, including decreased productivity and the perpetuation of poverty and social exclusion. On this, a pertinent example is that disadvantaged young people are twice as likely to be long‐term NEET (Not in Education, Employment or Training) as their better off peers (Gadsby, 2019)

As is standard with systematic reviews, content experts were consulted with to refine the search terms and define the list of databases to search. For this study, the content experts were drawn from the review advisory group.

To determine whether summer programmes produce improvements in outcomes of interest, and to estimate the magnitude of this relationship (where it exists) (see research questions 1 through 3), meta‐analyses have been conducted (where possible), employing the random effects model.

Since this systematic review also sought to identify components and features shared across successful summer programmes (see research questions 4 through 7), qualitative evaluations of the interventions shortlisted for meta‐analyses were examined to understand the causal pathway to outcomes. This was expanded by examples found in the UK where these met the inclusion criteria for the review, except for study design. This approach, recommended by the expert panel, sought to enable the review to tap into the UK context, particularly for implementation data. Where outcomes of interest were not observed or covered by these studies, these interventions do not feed into the analysis of the causal pathway.

## 5 SEARCH STRATEGY

Various electronic databases were systematically searched to identify studies for inclusion in the review:

- Key databases including Scopus, PsycInfo, Child Development and Adolescent Studies (CDAS), the Education Resources Information Centre (ERIC), and the British Education Index (BEI).
- Wider resources including the current unpublished updated YFF EGM, which includes studies from the 3ie Evidence and Gap Map/Kluve synthesis, the summer school streams of EEF's Teaching and Learning Toolkit and TASO's Evidence toolkit, and RAND's summer programmes evidence review (McCombs et al., 2019) that supports the Wallace Foundation's Summer Learning Toolkit.
- The Pathways to Work Evidence Clearinghouse, Clearinghouse for Labour Evaluation and Research; Office of Planning, Research and Evaluation (OPRE), Administration for Children and Families; MDRC; the National Bureau of Economic Research (NBER); and the Trip database.
- The most relevant journals – the Journal of Youth Studies, Youth & Society, International Journal of Adolescence and Youth (IJAY), Journal of Social Policy, and Youth.
- Sources of grey literature – Google Scholar, gov.uk, gov.scot, wales.gov.uk, northernireland.gov.uk, gov.ie, National Lottery Community Fund, Care Leavers Association, Children's and Young People's Centre for Justice, Joseph Rowntree Foundation (JRF), Centre on the Dynamics of Ethnicity, Nuffield Foundation, Regional Studies Association (RSA), Centrepoint, Youth Employment UK, Impetus, Edge, Education and Employers, National Foundation for Educational Research (NFER), the Sutton Trust, and TASO.

Several other databases that might be expected to be included in this list were not, as during piloting and testing of the search string, these additional databases surfaced limited/no additional studies of relevance. These additional databases cover Medline, the World Health Organization's International Clinical Trials Registry Platform, databases in the US National Library of Medicine including ClinicalTrials.gov, Social Care Online from SCIE, Epistemonikos, the libraries of Cochrane and the Campbell Collaboration, additional What Works Centres including the What Works Wellbeing, the Wales Centre for Public Policy and the What Works Centre for Children's Social Care.

Scopus is available through Elsevier, PsycInfo is available through the American Psychological Association; CDAS and BEI are available through EBSCO; the Journal of Youth Studies and the International Journal of Adolescence and Youth are available through Taylor & Francis Online; and the Journal of Social Policy is available through Cambridge Uni Press. In implementing the search strategy, the template and guidance provided by the Campbell Collaboration was followed (Kugley et al., 2017).

We used the following basic string to interrogate the identified databases:

(‘summer school*’ OR ‘summer learn*’ OR ‘summer education*’ OR ‘educational summer’ OR ‘summer bridge’ OR ‘summer employ*’ OR ‘summer work’ OR ‘summer place*’ OR ‘summer job*’ OR ‘summer apprentice*’ OR ‘summer intern*’ OR ‘summer camp*’ OR ‘summer program*’) AND (‘youth’ OR ‘young’ OR ‘child*’ OR ‘student*’ OR ‘pupil*’ OR ‘teenage*’ OR ‘adolescen*’ OR ‘juvenile’) AND (‘disadvantage*’ OR ‘vulnerab*’ OR ‘at risk’ OR ‘at‐risk’ OR ‘marginalised’ OR ‘marginalized’ OR ‘youth offend*’ OR ‘young offend*’ OR ‘delinquent’ OR ‘anti‐social’)

This string was developed through initial piloting and discussions with the review's advisory group. We used the full‐search string where possible. Where databases limit the length of the search string that can be used (either through physical limits or where the search function is too sensitive so that inputting the full search string is inappropriate), a hierarchical approach was employed, inputting as many of the key terms (ordered in terms of relevance) as possible, starting with those relating to the intervention before adding those relating to the population of interest (firstly age‐related, secondly disadvantage‐related). Depending on the size of the database and its subject‐matter focus, where a full advanced search was not possible searches centred on ‘summer’ or used individual searches for each of the terms relating to the programmes of interest that is, ‘summer school’ then ‘summer learn’ through to ‘summer program’. All fields of each record within each database were searched unless the number of hits was excessive and the relevancy of hits was too low. In these instances, searches were conducted within the abstract, title and/or key words.

The terms relating to the intervention type and the age/demographic group of participants were the predominant terms used in the literature. During piloting, a series of terms were tested relating to the disadvantage characteristics for the population of interest to identify whether these captured all of the literature of interest – some studies for instance might use specific disadvantage terms such as ‘poverty’ or ‘ethnic minority’ or ‘special educational needs’, raising a risk that they would not getting picked up by the search string. In each of the databases where it was possible to input the full search string, the piloting tested using 40 different search terms relating to specific forms of disadvantage. These additional searches yielded 1229 additional hits compared to the original shorter search string – of these, only six merited full text screening.

Where databases permitted, date limiters were applied to include studies published since January 1st, 2012, that is, approximately the last decade's research and covering the transition to and implementation of the Raised Participation Age (RPA) policy in England which affects education and training participation, and up to December 31st, 2022. This maximised the policy relevance of this review's findings. Only studies written in the English language were included; this is common practice across systematic reviews (Jackson & Kuriyama, 2019) despite potentially introducing bias to the review, although it has been shown that excluding non‐English language studies does not affect the main findings from meta‐analyses (Morrison et al., 2012). Additionally, the focus of the review on high income countries should also alleviate this as an issue, as studies based on interventions in high income countries may be more likely to be available in English, either primarily or as an alternative to the main non‐English language version. Furthermore, the saturation principle (discussed further in relation to the study design inclusion criteria) provides further support for this.

Searching Scopus surfaced relevant conference proceedings. Dissertations were included in the review where these were surfaced through the process detailed above, but no dissertation‐specific databases were searched. The references of the most relevant evidence reviews were searched thoroughly, namely the EEF and TASO toolkits and RAND's summer programmes evidence review (McCombs et al., 2019), as well as those of any additional systematic reviews surfaced through the search process.

We used ‘pearling’ (searching the citations of published evidence) to establish whether process studies were available for impact studies selected for the review, as well as to surface process studies related to UK interventions that were eligible for inclusion other than on study design (these are not included in the meta‐analyses or in the analysis of the causal pathway).

Where studies have an online appendix, these were also sourced manually and included alongside the main text if applicable.

The specific search string and details of the search process for each of the databases interrogated can be found in Supporting Information: Appendix 1.

## 6 STUDY ELIGIBILITY

### 6.1 Selection criteria

The following PICOSS criteria underpinned study selection.

#### 6.1.1 Population

The population of interest was individuals aged 10–25 years (where necessary, judgements were based on whether students typically turned this age in the academic year of study). The upper end of this range covers the age at which the vast majority of individuals in the UK will have exited education and entered economic activity as well as corresponding to the upper end of the age range of interest to YFF. The lower end covers the transition from primary to secondary education in the UK as well as the lower end of the age range of interest to YEF. Where the age range of the participants in a study spanned the eligibility boundary/ies, the study was included where the majority of the sample met the inclusion criteria, or if the impact of the programme for those meeting the inclusion criteria could be separated from those that did not.

The young people taking part in summer schools also had to be considered as disadvantaged or at risk of poorer outcomes. These terms are used widely throughout the literature despite not being strictly defined. The review was not bounded by a concrete definition of characteristics of disadvantage or that might make an individual at risk of poorer outcomes, rather, this was determined by the descriptions given in the sources material. All groups who face disadvantage or are at risk of poorer outcomes across the domains of interest compared to the wider population were included, such as (but not be limited to), racial and ethnic minorities, individuals of low socioeconomic status, individuals who have experienced care, students with Special Educational Needs, individuals with health conditions or disabilities, as well as those who have already offended or have experience of the criminal justice system, and those who are already experiencing poorer outcomes including poor academic performance or those truanting or being excluded from school. Where both disadvantaged and non‐disadvantaged individuals were included in any study population, the study was included where the majority of individuals met the inclusion criteria, or if the impact of the programme for those that met the inclusion criteria could be separated from those that did not.

#### 6.1.2 Intervention

As previously noted, the review centres on two main types of summer programme, which were surfaced by the preliminary literature review. These are summer employment and summer education programmes. For the purposes of this review, these programme types were operationally defined as follows:

▪ Summer employment programme: an out‐of‐school‐time programme that takes place during the summer months in whole or in part and includes a fixed‐term job placement.
▪ Summer education programme: an out‐of‐school‐time programme that takes place during the summer months in whole or in part, where content is majority administered through education‐focused instruction.

The summer months refer to the period in which the long vacation takes place between academic years or after the final academic year before moving into economic activity (referring to the employed, unemployed, and economically inactive populations above working age). Interventions that take place during the summer but are targeted at individuals who have already transitioned into economic activity were not of interest. Summer programmes that were a part of a wider intervention, for instance, including term‐time provision, were eligible for inclusion although the features, mechanisms and/or outcomes of the summer programme should be able to be separated out from the other components of the intervention, and/or the summer programme should constitute a substantial enough component of the whole for it to be reasonable to include. This was determined on a case‐by‐case basis – the reasoning behind decisions for any marginal cases are made transparent.

As part of the peer review process, we were challenged on the decision to exclude one cohort participating in the summer employment programme evaluated by Davis and Heller (2020) based on the population being ineligible. The individuals in what is described as the 2013 cohort in the study (note that the 2012 cohort was eligible for inclusion in the review) were aged 16–22 and approximately 41% came directly from the criminal justice system, and 59% came from high‐violence neighbourhoods. Both groups were deemed ineligible for inclusion based on the combination of their age and lack of requirement to be in education. The latter meant that the intervention would take place in a non‐transitional summer for these individuals.

The exclusion of the former group raises an issue as to how our population and intervention criteria interact. Individuals currently or previously involved in the criminal justice system are of significant interest to this review, but those entering a summer programme directly from the criminal justice system would not be ‘between academic years’. Studies are eligible for inclusion where the population comes from the criminal justice system at an age where, were they not involved in the criminal justice system, they would theoretically be in compulsory education or required to participate in education or training. For those young people that are in the criminal justice system and that, were they not, would be in economic activity instead, there is no need for the education/employment intervention they participate in to occur during the summer period as there is no reason for their transition out of the criminal justice system to be synchronised with the academic calendar (i.e., the summer period between academic years or after the final academic year which we are focused on). As such, theoretically for this population all education/employment interventions that occur in the period when they are transitioning from the community justice system back into the community could be included, but the review would lose its focus on *summer* programmes. Therefore, we stand behind our decision to exclude the cohort from Davis and Heller (2020) that in part comes directly from the criminal justice system.

This issue plays a role in determining the eligibility of only one other study surfaced by the review (in all other instances, another eligibility criteria results in the study's exclusion). Tarling and Adams (2012) evaluates the Summer Arts Colleges programme which targets at risk young people in England and Wales, including those leaving the criminal justice system. While the age range of the participant population was 12–19, the vast majority of participants were 15–17 and the average age was 16.4 years. Therefore, this study is included in the review, as for the vast majority of participants their involvement in the criminal justice system is at the expense of compulsory education, and therefore the intervention for them occurs during the summer months between what would be academic years, or after what would be their final academic year before moving into economic activity.

Sports programmes (which according to a broader definition could be considered education programmes) that were subjected to the systematic review of Malhotra et al. (2021) were not included. The definition of these sports programmes did not overlap with the interventions of interest to us – however interventions that met the definition of summer employment or summer education programmes which also featured sports activities were eligible for inclusion. Programmes of educational instruction that did not serve academic purpose, for instance cycle training programmes, were not considered – programmes where the educational instruction related to understanding of/familiarisation with transitions such as to higher education, were eligible as these employed various mechanisms of interest and in the broader sense constituted a summer education and not enrichment programme. Residential programmes which aimed to achieve this through familiarising students with a new environment were considered, provided that there was some taught element and the programme was not solely focused on enrichment activities. Programmes such as reading challenges or book gifting programmes without guided instruction were also not considered.

These definitions made the programme types mutually exclusive, and studies that evaluated a summer employment or summer education programme were included.

Interventions for inclusion should have been targeted at the population groups identified above. Universal interventions where young people that are disadvantaged or at risk of poorer outcomes fall into the intervention population but were not specifically targeted were not considered. The interventions should also have been provided directly to the population of interest, as opposed to indirectly through a third party such as their parents or teachers.

#### 6.1.3 Comparison group

Primary studies were included in the systematic review where they drew on a comparison group (QED) or control group (RCT). The comparison group were young people who do not participate in summer programmes covered by the evaluation but who are similar to those who do participate. Typically, primary studies would draw comparison with groups of young people experiencing business as usual (BAU). Being able to access comparative analysis between intervention strands within primary evaluation reports was crucial for studies to be included. This requirement was dropped for studies evaluating UK‐based interventions which meet all the criteria for inclusion except for study design. These latter studies were only included in the analysis covering implementation and were not included in the meta‐analyses or in the analysis of the causal pathway, unless a relevant outcome was observed.

#### 6.1.4 Outcomes

The review examined the impact of different types of summer programmes across the five outcome domains of interest: (1) violence and offending; (2) education; (3) employment; (4) socio‐emotional; and (5) health (where these are included alongside other outcomes of interest). To be included, a study must have evaluated the intervention according to an outcome within at least one of these domains, with those studies considering health outcomes included only where outcomes within another domain were also covered. This was to avoid ‘weight loss camps’ or programmes solely at helping young people to manage health conditions/disabilities from inclusion. Where these health interventions only looked to affect socio‐emotional outcomes which can be thought of as direct consequences of potential health outcomes, as opposed to distinctly separate outcomes (for instance, weight loss camps may also consider impacts on confidence and self‐esteem), then these were also not considered. Within the context of this systematic review, violence and offending also includes anti‐social behaviour.

The preliminary literature review identified that the outcomes measured as part of the evaluation of relevant interventions were mostly relatively short term, with studies often not following‐up after programme end. As such, outcomes that would usually be considered as intermediate, such as the acquisition of skills and attributes outlined in YEF's Outcomes Framework, were also considered as outcomes of interest to this review.

The specific outcomes that are of interest were guided by the Outcomes Framework of YEF and the outcomes of interest to YFF, as well as the preliminary literature review to scope existing evaluations of summer programmes. These include:

▪ Violence and offending – reduced offending and reoffending; reduced likelihood of carrying weapons. Both the severity and intensity of violent and offending behaviour were appropriately considered, as was the differentiation between self‐reported measures and measures based on recording from the police and/or criminal justice system.
▪ Educational – education and qualification completion (including performance and attainment in courses/exams); access to/in education (including application, participation, and completion in courses); education quality; technical skills & vocational training; improved study skills and academic mindset; improved critical and analytical skills.
▪ Employment – employment status; whether actively seeking employment; employment expectation; whether found appropriate employment; hours worked; job quality; earnings & salary; development of work appropriate ‘soft‐skills’ including job‐search skills.
▪ Socio‐emotional – resilience and persistence; increased confidence; improved behavioural adjustment indicators; improved social skills; community engagement; ability to manage emotions and resolve conflicts.
▪ Health – better understanding of health issues including substance use, physical activity, and nutrition; improved family well‐being; improved access to health‐related support services.

Where relevant, outcomes from longitudinal analyses are differentiated from those from correlational or cross‐sectional analyses. This can be seen explicitly as part of the thematic synthesis as these outcomes are more naturally differentiated in the meta‐analysis.

#### 6.1.5 Study design

Experimental (RCT) and quasi‐experimental designs (QEDs) (including but not limited to regression discontinuity designs (RDDs), difference in differences (DIDs) and matching approaches) as part of evaluation studies with a robust and credible comparison group were included. Study designs that do not include a parallel cohort that establishes or adjusts for baseline equivalence are not considered to employ a robust and credible comparison group. These invalid designs could include single group pre‐post designs; control group designs without matching in time and establishing baseline equivalence; cross‐sectional designs; non‐controlled observational (cohort) designs; case‐control designs; and case studies/series.

Empirical studies looking at the implementation of an approach or process evaluations were included in the review to examine implementation questions – these were sourced from the pearling the citations of included counterfactual impact evaluations. Qualitative evaluations of this latter type of UK‐based interventions where these meet the inclusion criteria except for study design were also included.

To include qualitative evaluations in the thematic synthesis where we consider how the contexts of summer programmes affect the outcomes achieved through various mechanisms, a credible indication of what impacts the intervention achieved was necessary. Therefore, we required qualitative studies to be linked to a robust impact evaluation. Nonetheless, as this review is most interested in policies in the UK where there has until recently, with the development of the What Works movement, been a lack of tradition for robust impact evaluation, this requirement was suspended in this context.

Initial piloting of the search string to support the development of the protocol suggested that applying these requirements on study design would still result in a substantial amount of literature to review. Theoretical saturation, a well‐established approach in qualitative primary research (e.g., Hennik & Kaiser, 2022; Morgan et al., 2002), suggests that beyond a certain point any additional qualitative evaluations would not provide new information to inform the findings of the review. As such, not including qualitative evaluations of eligible interventions that do not fulfil the requirements on study design is unlikely to affect the main findings of the thematic synthesis. Furthermore, any impact is outweighed by the certainty with which we are able to make assertions relating to outcomes, given that the qualitative and process information is related to an intervention that has been subject to a robust impact evaluation.

#### 6.1.6 Setting

The review covers summer programmes implemented in high‐income countries (as defined by the World Bank (2023) for July 2022 to July 2023) at any level (i.e., national, regional and local programmes).

#### 6.1.7 Summary of PICOSS criteria

Table 1 summarises the PICOSS inclusion and exclusion criteria detailed above.

**Table 1:** PICOSS criteria.

	Inclusion criteria	Exclusion criteria
Population	Young people that are disadvantaged or at risk people of poorer outcomes aged between 10 and 25.	Young people aged less than 10 or more than 25 or not disadvantaged or at risk of poorer outcomes.
Intervention	**Summer employment programme**: an out‐of‐school‐time programme that takes place during the summer months in whole or in part and includes a fixed‐term job placement. **Summer education programme**: an out‐of‐school‐time programme that takes place during the summer months in whole or in part, where content is majority administered through education‐focused instruction.	Programmes that do not fulfil the criteria of either a summer employment or summer education programme.
Comparison	Treatment as usual, another intervention, no intervention, or wait‐list control.	Studies that cover a population that is different in observable characteristics and that receive an alternative intervention not tracked by evaluation. Studies that mobilise non‐counterfactual measures except eligible studies of UK‐based interventions.
Outcome	Studies that examine: (1) violence and offending; (2) academic; (3) employment; (4) socio‐emotional; or (5) health outcomes.	Studies that examine other outcomes while not covering the outcome domains of interest. Studies that only consider health outcomes or health outcomes plus socio‐emotional outcomes that are direct consequences of health outcomes.
Study design	Randomised Controlled Trials (RCT) including individual and cluster level randomisation. Step‐Wedge designs with random time allocation. Non‐equivalent control group designs using parallel cohorts that adjust for baseline equivalence. Difference‐in‐Difference estimation. Interrupted time‐series. Synthetic control group methods. Studies based on: − covariate matching; − propensity score‐based methods; − doubly robust methods^a^; − regression adjustment; − regression discontinuity designs; and − instrumental variable estimation. Qualitative studies and economic evaluations will be included if they are conducted as part of a qualifying study and will be used only to generate hypotheses, inform us about the interventions and populations, and inform or deepen our understanding of the quantitative findings. They will be included however if they are evaluating UK‐based interventions and are identified via the searches or recommended to this study by experts.	Non‐primary studies (except studies of this type that are evaluating UK‐based interventions), including: − literature reviews; − systematic reviews; − meta‐analysis; and − non‐primary QEDs. Studies without a valid counterfactual, including designs that do not include a parallel cohort that establish or adjust for baseline equivalence (except studies of this type that are evaluating UK‐based interventions), including: − single group pre‐post designs; − control group designs without matching in time and establishing baseline equivalence; − cross‐sectional designs; − non‐controlled observational (cohort) designs; − case‐control designs; − case studies/series; and − surveys.
Setting	Studies that are undertaken in high income countries, as defined by the World Bank (2023).	Studies that are not undertaken in high income countries, as defined by the World Bank (2023).
Other	Studies that are published in English.	Studies that are not published in English.
	Studies published since 2012 up to the end of 2022.	Studies published before 2012 or since 2023.

^a^ ‘Combines a form of outcome regression with a model for the exposure (i.e., the propensity score) to estimate the causal effect of an exposure on an outcome’ (Funk et al., 2011, p. 761)

*Source*: IES (2024).

### 6.2 Study selection process

Covidence was used as the data screening software. Once the longlist of studies had been compiled, the abstracts and summaries were screened against the agreed inclusion/exclusion, quality, scope, and applicability criteria. Titles and abstracts were initially screened by two reviewers. Where a conflict arose, it was resolved by a third reviewer. Where evidence failed to meet criteria, it fell out‐of‐scope. The output from this stage was a sub‐set of the search database tagged ‘for review’.

Subsequently, the full text of all potentially eligible evaluations was retrieved and reviewed for eligibility, independently by two members of the team using our a priori eligibility criteria. Full‐text review was completed by two reviewers for both inclusion and exclusion. Reasons for exclusion were recorded in the notes of each study in Covidence.

Where the full text of a potentially eligible evaluation was not available online, either for free or behind a paywall for which access to was acquired, the authors of the study were contacted. This was done across multiple communication channels (largely email, LinkedIn and Twitter) and was successful in several cases. These along with all of the studies included in the review were retained for reference. There were three cases where full texts could not be sourced for the original paper or it was not published. These were (first author (publication date) *title*): Johnson‐Weeks (2014) *An evaluation of the academic effectiveness of a summer bridge programme*; MacRae (2014) Media Space; Sutton Trust (2013, https://www.suttontrust.com/our-research/evaluation-of-us-summer-school-programme/) *Evaluation of US summer school programme*.

## 7 QUANTITATIVE DATA EXTRACTION PROCESS

Excel spreadsheets were used for data extraction. The shortlist of included papers was extracted from, using standardised pro‐forma to ensure consistency of data capture. An initial pilot of extraction using the pro‐forma was undertaken supported by team meetings to build consensus about what to extract and how, in particular related to the qualitative (thematic) data where extraction is more subjective than for quantitative data.

For included studies, data was extracted by a single reviewer (with a peer reviewer process to check accuracy) into an online version of the spreadsheet pro‐forma (enabling multiple simultaneous users) developed for this review. This form was drafted with multiple individuals inputting into its design and was tested via a dry run with an individual who had not yet seen it, to examine its usability and ability to capture all the necessary information for the synthesis of findings.

Once the study had passed the full‐text review, one reviewer extracted the necessary information into the extraction form, and another reviewer with a significant quantitative background checked the accuracy and relevance of the extracted information, in particular the extraction relating to the estimates of impact. Points of contention around the extraction, including when extracting datapoints that are subjective such as whether the population could be considered disadvantage/at risk of poorer outcomes, were discussed with the wider review team through the Microsoft Teams channel dedicated to the research project before reaching a consensus verdict.

Alongside quantitative information relating to the impact of summer programmes, we also extracted from both quantitative and qualitative evaluations data relating to the costs associated with delivering summer programmes. These are analysed separately to the synthesis of findings relating to the impact of summer programmes.

## 8 RESULTS FROM SEARCH AND SELECTION

### 8.1 Flow of studies through the review

The search process, including the pearling of studies that would go on to be included in the review, resulted in a pool of 1332 studies, out of which 652 were duplicates, leaving 707 studies for title and abstract screening. Of these, 210 were full‐text screened – 142 of these studies were excluded, with the most common main reason for exclusion being the study design. This left a pool of 68 studies to be included in the review, of which 41 were impact evaluations with a study design eligible for meta‐analysis. 49 of these studies evaluated 36 different summer education programmes, and 19 studies evaluated 6 summer employment programmes, with three of these programmes (New York City and Boston Summer Youth Employment Programs, and One Summer Chicago) being responsible for 15 of the studies.

**Figure 1:**
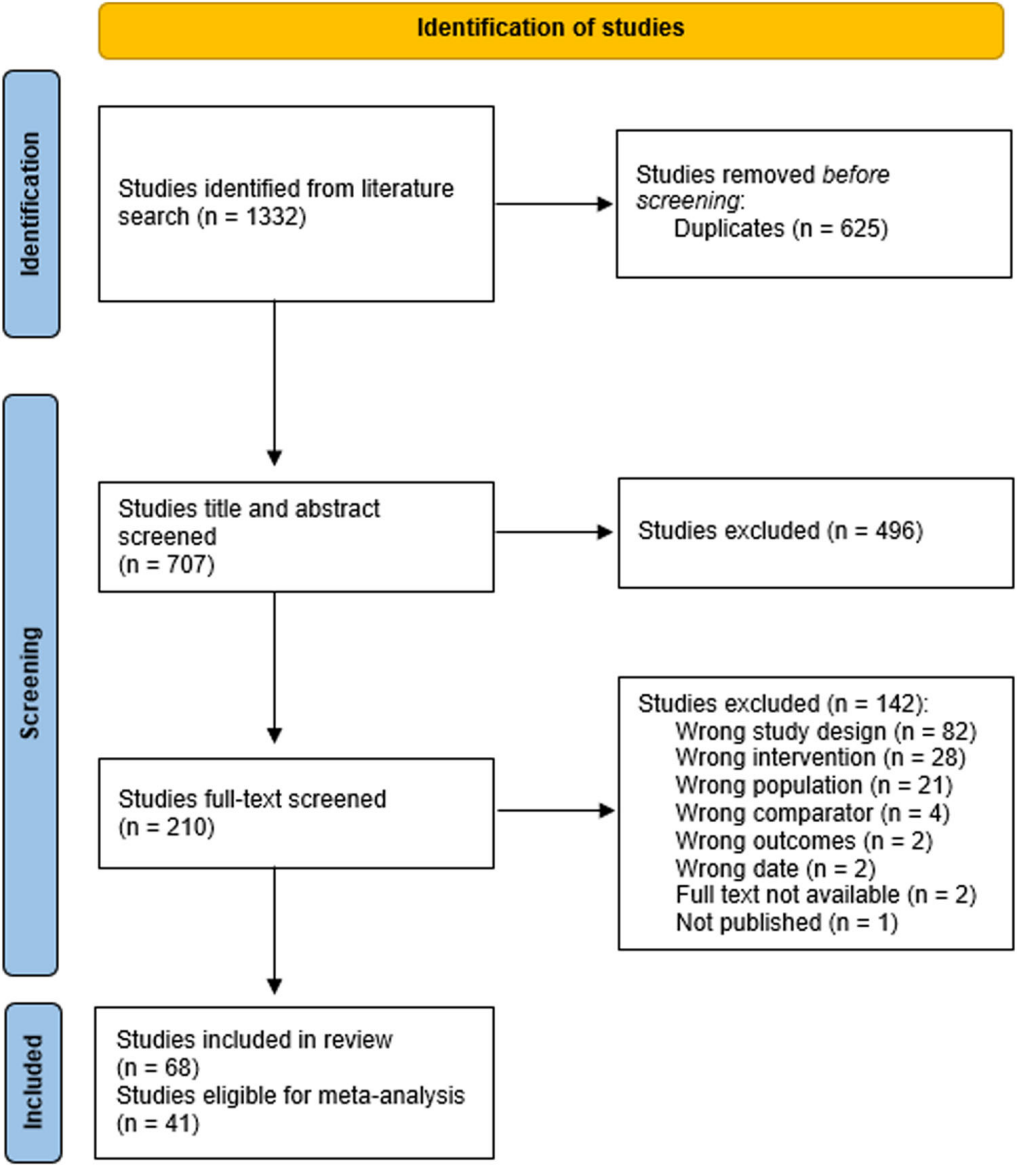
PRISMA diagram. *Source*: IES (2024).

Figure 1 displays the PRISMA diagram for this review, showing the flow of studies through the identification, screening and inclusion stages (Page et al., 2021). The main reason for exclusion (note that a study could have been excluded for multiple reasons) at the full text screening stage is listed.

### 8.2 Notable excluded studies

Various studies that might be expected to have been included in the review were not, as they were deemed ineligible based on our PICOSS criteria. Table 2 details some of these studies with reasons as to why they were excluded from the review.

**Table 2:** Notable excluded studies.

Study name	Intervention name	Description of study and intervention	Reason for exclusion
Alam et al. (2013)	Summer job experience, Falun, Sweden	Exploits lottery to estimate impact of allocation to summer jobs experience with local council on accumulated work experience and earnings.	Participants are not disadvantaged.
Gonzalez Quiroz and Garza (2018)	Laredo Community College summer bridge programme	Use propensity score matching to construct comparison group that treatment group that the academic outcomes of participants in college bridge programme were compared to.	Participants are not sufficiently disadvantaged (majority of students in the US complete FAFSA),^a^ and limited details on study design and baseline characteristics means that we cannot be certain that they have appropriately adjusted for baseline equivalence.
Kallison and Stader (2012)	Bridge programmes, Texas colleges	Investigates effectiveness of two community college programmes whose participants saw high achievement gains.	Do not employ a comparison or control group, no indication that examining the same intervention(s) as Barnett et al. (2012) or Wathington et al. (2016).
Sablan (2014)	Various summer bridge programmes	Characterises summer bridge programmes, reviews the existing literature and discussed implications of findings from these reviews on future summer bridge programmes.	Only performs secondary research (literature review) – references were pearled.
Lynch et al. (2021)	Various summer learning programmes	Assess the impact of summer learning programmes on low‐income children's mathematics achievement.	Only performs secondary research (meta‐analysis) on population that is ineligible based on age – references were pearled.
Sinatra & Eschenauer (2012)	Summer academy programmes, Department of Homeless Services	Examine impact of innovative learning programmes for homeless children and adults on academic performance.	Unclear as to whether experimental design or not, unsuccessfully contacted authors for clarification but based on available information assume that is not and therefore ineligible on study design.
Augustine et al. (2016)	Voluntary summer learning programmes, part of National Summer Learning Project	Range of RCTs evaluating impact of summer learning programmes on academic and socio‐emotional outcomes among low‐income urban young people.	Population ineligible based on age (third grade).
Williams & Mellors‐Bourne (2019)	Realising Opportunities	Evaluation of included intervention, using surveys and matched comparison group.	There is no focus on the summer programme component of the intervention that can be used in the thematic synthesis; estimates of impact cannot be considered to be representative of the summer programme component.
Zajic (2017)	Lions Academy and traditional summer school	Looks at effect of summer programme for students from low socioeconomic backgrounds on reading and mathematics performance.	Ineligible study design as does not sufficiently adjust for baseline equivalence with parallel cohort when producing estimates of impact on outcomes of interest.

^a^ Bahr (2018) found that 65% of students reported completing a FAFSA.

*Source*: IES (2024).

### 8.3 Eligible studies that are not included

Aside from interim evaluations of other included studies – for instance, Theodos et al. (2016) which is entirely superseded by Theodos et al. (2017) – all eligible studies are included in the review.

### 8.4 Ongoing studies

No ongoing studies were identified as being relevant to the review.

### 8.5 Characteristics of included studies

Supporting Information: Appendix 2 details the key characteristics of the included studies (location and period covered by the evaluation, characteristics of study population, the main sample size, features of the intervention, the study's methods/design, whether it is eligible for inclusion in the meta‐analysis, and the outcome domain which it evaluates).

## 9 STUDY CONFIDENCE

### 9.1 Impact evaluation risk of bias assessment

As recommended by the YFF EGM protocol, the Quality Assessment of Impact Evaluations Tool (Saran et al., 2020) was used to evaluate the methodological quality of the 41 included impact evaluation studies. The tool scores studies as high, medium and low confidence across seven items: *study design* (related to confounders); *sample size*; level of *attrition* or losses to follow up; *definition of intervention*; *definition of outcomes*; *baseline balance* in characteristics; and *overall confidence* (the lowest confidence level across each of the other items). Table 3 details the criteria used to assess studies against each of the main items.

**Table 3:** Quality Assessment of Impact Evaluations Tool.

Confidence level	Study design	Adequate sample size	Level of attrition or losses to follow up^a^	Definition of intervention	Definition of outcomes	Baseline balance
High	Randomised controlled trial (RCT), regression discontinuity design, interrupted time series, instrumental variable	Sample size ≥100 or cluster ≥60	Attrition within Institute of Education Science (IES) bounds	Intervention clearly and fully described	Outcome measure clearly and fully described, preferably with reference to validation	RCT or baseline balance report and satisfactory (imbalance of 5 or less than 5%)
Medium	Difference in differences with matching, propensity score matching	Sample size <100 or cluster <60	Attrition close to IES bounds	Brief description of intervention	Brief description of outcome	Imbalance between 5% and 10%
Low	Other matching	No power calculation or sample size <30 or cluster <30	Attrition not reported or attrition outside IES bounds	Intervention named but not described or named	Outcome named but not described	Baseline balance not reported, or reported and lack of balance on 10 or more than 10%

^a^ To operationalise these definitions, we used the standards set out in the What Works Clearinghouse Procedures and Standards Handbook (Institute of Education Sciences, 2011) for assessing attrition bias. Using figure A.1, a high confidence level is assigned to studies falling in the green region, medium confidence to studies falling in the yellow region, and low confidence falling in the blue region/outside the green and yellow regions.

*Source*: Saran et al. (2020).

Table 4 shows the results of the risk of bias assessment across each of the seven items, displaying the number and proportion of included impact evaluations evaluated at each confidence level.

**Table 4:** Results from risk of bias assessment.

	Study design	Adequate sample size	Level of attrition or losses to follow up	Definition of intervention	Definition of outcomes	Baseline balance	Overall confidence level
Low	7 (17%)	0 (0%)	27 (66%)	0 (0%)	0 (0%)	6 (15%)	30 (73%)
Moderate	7 (17%)	3 (7%)	7 (17%)	6 (15%)	6 (15%)	14 (34%)	9 (22%)
High	27 (66%)	38 (93%)	7 (17%)	35 (85%)	35 (85%)	21 (51%)	2 (5%)

*Source*: IES (2024).

Nearly three‐quarters of the included impact evaluations were categorised as low confidence. Attrition was the item that the included studies performed the worst on, with two‐thirds of all included studies receiving a low confidence categorisation for this one item, which was due largely to relatively high levels of individuals assigned to treatment not taking up their allocation. This might indicate the potential for selection bias in the results, as the decision to take up treatment allocation may not be random. As such and as detailed further later in the review, analyses based on intention‐to‐treat estimates should thus be prioritised. Meanwhile, two‐thirds of studies received a high confidence categorisation for their study design, with seven studies each receiving moderate and low confidence categorisations.

As study design is the main determinant of whether an impact evaluation can remove bias from estimates, in the sub‐group analysis it was important to explore how effect sizes varied in relation to whether a study was categorised as having a high (RCT, RDD, interrupted time series, instrumental variable), moderate (DID with matching, PSM) or low confidence (DID, other matching, other eligible designs) study design. It should be noted that the study design for evaluations of summer employment programmes is generally higher than for valuations of summer education programmes. Of the 14 studies eligible for meta‐analysis evaluating summer employment programmes, 12 have high‐quality study designs, driven by the large number of RCT‐based studies evaluating the New York Summer Youth Employment Program, Boston Summer Youth Employment Program and One Summer Chicago. Conversely, only 15 of the 27 studies evaluating summer education programmes eligible for meta‐analysis have high‐quality study designs.

### 9.2 Qualitative data quality assessment

**Figure 2:**
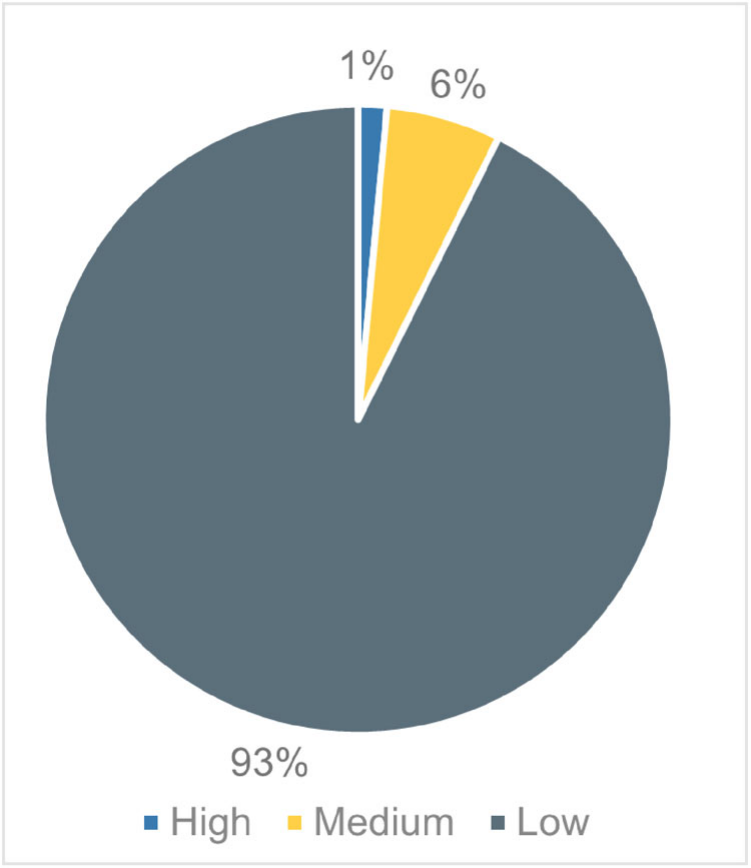
Overall quality of studies included process and qualitative studies (*n* = 68). *Source*: IES (2024).

As set out by the YFF EGM protocol, the Questions for Process Evaluations are used to assess quality, in terms of the qualitative and process information, of all 68 studies included in the review. Given the large number of them and the general lack of specific process/qualitative evaluations, impact evaluations are an important source of information for the thematic synthesis, therefore these studies are also subjected to the qualitative data quality assessment. As this was largely not the intended purpose of the original authors, these studies were generally rated low quality with regard to their qualitative information. These questions cover whether: methodology is described and appropriate to the research questions; the sampling strategy is described and appropriate; the researcher(s) has identified potential sources of bias from their own position, and ethical issues; the approach to analysis is identified and robust; and, whether evidence supports any recommendations. From this, an overall quality assessment (high, medium or low) is derived based on the lowest quality level across each of the other questions. Figure 2 shows the results from the quality assessment of all the studies included in the review.

The majority (93%) of the reviewed studies were rated as low quality. This is because many studies did not discuss any ethical considerations (59 studies, 86%) or highlight any researcher assumptions (42 studies, 61%). In addition, many studies were either purely quantitative (41 studies, 60%), or focused only on quantitative aspects of the research despite taking a mixed methods approach (6 studies, 9%). Some studies did not discuss other aspects of the research; most commonly the recommendations or implications were missing (14 studies, 21%). However, several of the studies in this review were considered to have clearly described their approach to sampling (42 studies, 61%) and data analysis (45 studies, 66%).

The high proportion of low‐quality studies in this review highlights the need for improvements in the quality of evidence, particularly qualitative insights, available to more accurately assess the impact of summer education and employment programmes on disadvantaged or ‘at risk’ young peoples' outcomes.

## 10 DATA ANALYSIS AND PREPARATION, AND APPROACH TO QUANTITATIVE SYNTHESIS

### 10.1 Constructing effect sizes

Our approach to constructing effect sizes is based on that recommended by the Cochrane Handbook for Systematic Reviews of Intervention. Where effect sizes with confidence intervals are reported, these are used as is. Where the results from logistic regressions are provided, the reported odds ratio and reported or derived confidence interval are converted directly to log odds ratios by taking the natural log. Whatever information was available in the study was used to derive the necessary information to construct effect sizes from the impact estimates. This includes, where necessary:

▪ using reported *p*‐values to calculate the *Z* value/*T*‐statistic using formulae for the standard normal/*t* distributions – where levels of significance are reported, for example, *p* < 0.05, we conservatively take the *p*‐value to be at the upper limit, that is, *p* = 0.05 – if a result is reported only as being insignificant, we assume *p* = 0.5 (the use of this assumption is required only for the impacts on test scores from Herrera et al. (2013) where they report effect sizes themselves with only this type of information about variability. As such, sensitivity analysis around this is not performed);
▪ using the *Z* value/*T*‐statistic to construct the standard error by dividing the treatment effect estimate/mean difference by this; and
▪ using the standard error to construct confidence intervals.

Where a treatment effect that is the percentage point or mean increase in the outcome is reported, this is added to the prevalence/mean of the outcome among the control group to derive the adjusted prevalence/mean of the outcome among the treatment group. Should the prevalence/mean of the outcome be reported among the treatment and not the control group, then the treatment effect is subtracted from the prevalence/mean of the outcome among the treatment group to derive the adjusted prevalence/mean of the outcome among the control group. Prevalence/mean of the outcome post intervention are used where reported, otherwise baseline/pre‐intervention figures are used.

Where the split in the number of observations between the treatment and control groups are missing from the analysis sample but the overall analysis sample size is available, the split is assumed to be the same as that used for any descriptive analysis.

Where the standard deviation of the dependent variable is not reported separately for both the treatment and control groups, depending on what information is available we either:

▪ assume that figure for the treatment group is the same as for the control group (which is often reported on its own);
▪ use the reported figure from a sample external to the intervention, for example, they may report the standard deviation in scores among all students taking a test nationally;
▪ impute it directly from other studies also evaluating the same outcome, which is shown to produce approximately correct results (Furukawa et al., 2006);
▪ impute it from other studies by exploiting a strong linear relationship between the log of the mean and the log of the standard deviation (see e.g., Marinho et al., 2003); or
▪ use the method suggested by Walter and Yao (2007) and estimate it as the range of observed values divided by 4.

We use the formula provided by Cohen (1988) to calculate the pooled standard deviation where required. Despite the range of potential approaches to solving the issue of missing standard deviations, for some outcomes there is insufficient information available to perform any of these or any other approaches. This is usually the case where the only measure of variability reported relates to the treatment effect (e.g., the standard error of a regression coefficient), not the dependent variable itself.

In all cases where there was insufficient or missing information that was required to construct effect sizes, the study's authors were contacted to acquire the information. This, as is common, was largely unsuccessful, although in some cases the required information was provided:

▪ Theodos et al. (2017) – missing splits between the number of treatment and control observations for each evaluated outcome were provided.
▪ Williamson et al. (2020) – all the necessary data behind the results that were reported graphically was provided.

Where studies only reported results graphically (only the case for Valentine et al. (2017)), Windows Paint was used to derive the numerical results behind these – PlotDigitizer was also used but using the former was found to produce more precise results.

Where moderators are used in regression analysis to derive the impact estimates, the treatment effect can be derived relatively easily by combining the coefficient associated with the unmoderated dummy of allocation to/participation in the summer programme with those associated with the interaction terms of the allocation/participation and sub‐group dummies, each multiplied by the number of individuals in the sub‐group.

There are no included studies that employ cluster‐level randomisation, therefore there is no need to calculate effective sample sizes or modified results based on the potential dependence in outcomes between individuals.

Where results are reported from multiple specifications of the estimation model, results from specifications including baseline covariates and/or the most other controls which will produce the least biased estimate are preferred. Other decisions about which specifications' results were used are detailed below.

The Campbell Collaboration's and/or the University of Cambridge Centre for Evaluation & Monitoring's effect size calculators were used to calculate standardised effect sizes have been used. For dichotomous outcomes, such as whether in employment or not, we construct (log) odds ratios. For continuous outcomes, where possible we use Hedges' *g* to report standardised mean differences (SMDs). However, given that both the standard deviation of the dependent variable among the treatment and control groups are frequently missing, it is not always possible to calculate Hedges' *g* and instead Cohen's *d* is reported. Where a meta‐analysis finds a significant average effect size, to aid understanding this is translated back into one of the natural units of measurement used by one of the studies that evaluated the outcome for continuous outcomes, or the number needed to treat (the number of individuals that need to be allocated to/participate in a summer programme to achieve the outcome concerned) for dichotomous outcomes.

### 10.2 Approach to quantitative synthesis

The main approach used to estimate average effect size and the variability of effect sizes is to perform meta‐analysis using the random‐effects model (specifically the restricted maximum likelihood method which produces an unbiased, non‐negative estimate of the measure of between‐study variability) – a consensus approach commonly used in meta‐analysis. The model assumes that studies are estimating different yet related intervention effects and incorporates heterogeneity into the estimated average effect size (through adjustments to the study weights), on the assumption that the underlying effects follow a normal distribution. This was selected over fixed effects to enable the results of the analysis to be applicable beyond the included studies and given study heterogeneity (in terms of intervention population, form of the intervention, labour market context and so forth) it is unsound to assume that there is a common effect across the included interventions. Across the twenty‐two outcomes (excluding impacts on employment outside of the summer employment programme which is an additional exploratory analysis and not one of the main outcomes considered) for which meta‐analysis was performed, thirty‐two separate main meta‐analyses (based on the impact of allocation or the impact of participation if this was not run) across the different summer programme types (all summer programmes, summer education programmes and summer employment programme) were performed. We explored the presence of heterogeneity through the *I* ^2^ statistic, a commonly used measure of consistency in effect sizes between studies (Higgins, 2003), and the homogeneity test based on the *Q* statistic. For the thirty separate main meta‐analyses where multiple studies evaluated the summer programme type (and therefore the *I* ^2^ statistic and *p*‐value from the homogeneity test are reported), the *I* ^2^ was high (over 75%) for ten of them and was between moderate and high (50%–75%) for another six, and the *p*‐value indicated statistically significant (at the 90% level which was used to account for small sample sizes, discussed further later in the review) heterogeneity in effect sizes between studies for 15 of the meta‐analyses. This somewhat justifies our decision to be conservative in the assumptions we were willing to make when selecting our model.

Initially it was planned that studies analysing participants as members of the groups to which they were originally assigned (intention‐to‐treat analysis), studies including only those participants who were willing or able to provide data (available‐case analysis), and studies analysing participants who adhered to the study's design (per‐protocol analysis) would be analysed separately. However, due to the numbers and distribution of studies across these categories, it was decided that the analysis would be performed separately for those studies performing intention‐to‐treat (ITT) analysis, that is those estimating the impact of allocation to a summer programme, and those performing non‐ITT, so those estimating the impact of participation in a summer programme. Furthermore, we planned to contact authors to provide additional information to permit ITT analysis across all studies. However, given the low success rate of author contact, to increase sample sizes to allow us to perform more and more powerful main and sub‐group analyses, and to perform meta‐analyses from studies that estimate only non‐ITT impacts (while also running analyses separately based on the type of analysis performed by studies as specified in the protocol), we transform the impact estimates from ITT analysis into non‐ITT estimates by dividing the estimated treatment effect by the differences in treatment rates between the treatment and control/comparison group. See, for instance, Valentine et al. (2017) as an example of a study that perform this naïve transformation from impacts of allocation to impacts of participation themselves. We do not transform the results from non‐ITT analyses to ITT analyses given that information about initial allocation/eligibility are often not reported. Results from ITT analysis are the preferred ones, as results from non‐ITT may be biased if there are systematic reasons as to why certain individuals do/do not provide data/have their data available or comply with the study protocol. Those estimates of the impact of participation in a summer programme that are based on results reported by the study should be prioritised over those derived through transformation of estimates of the impact of allocation to a summer programme, as the transformation process is imperfect and is also affected by missing data/data limitations. The extent to which transformed results affect the non‐ITT findings is examined through sensitivity analysis.

Transformation of results from non‐ITT analyses to ITT results is not required should a study perform both forms of analyses or should all participants comply with their allocation to the treatment or control/comparison group. Both the treatment effect and treatment and control/comparison group sample sizes are adjusted to reflect the actual difference in treatment rates. As an example, suppose that allocation to a summer programme increased the likelihood of applying to higher education by five percentage points, only 90% of those allocated to the treatment group end up participating in the programme and 10% of those allocated to the control group actually participate in the programme – the transformed impact of participation in the summer programme would be 6.25 percentage points (five percentage points divided by the difference in treatment rates between the treatment and control groups). The standard deviation of the dependent variable is assumed to remain the same. If it is the standard error of the dependent variable that is reported from which the standard deviation is derived, this is transformed by dividing by the root of the ratio of the sample size before and after transformation. If the study only reports an effect size that then requires transformation, this is converted back into the prevalence/mean of the outcome, the difference in which between the treatment and control group is then transformed before re‐converting this into an effect size. In certain instances, insufficient information is provided to convert an effect size back into the prevalence/mean of the outcome, therefore deriving transformed estimates of impact of participation in the summer programme is not possible. Table 5 displays the difference in treatment rates between the treatment and control group used to transform the results in studies that only perform ITT analyses.

**Table 5:** Difference in treatment rates used to transform intention‐to‐treat estimates.

Study (Outcome)	Treatment rate among treated	Treatment rate among control	Source
Cohodes et al. (2022)			
Six‐week arm	87%	0%	Table A.2:
One‐week arm	85%	0%	Table A.2:
Online arm	77%	0%	Table A.2:
Gehring et al. (2018) and Henson (2018) – progression to higher education	84%	0%	Average across other studies evaluating the outcome
Gorard et al. (2014)	79%	0%	Page 18
Herrera et al. (2013)/Garcia et al. (2020) (4 years only)			
1st spring post‐programme	75%	0%	Page 8
2nd spring post‐programme	70%	0%	Page 8
4th spring post‐programme	47%	0%	Page 8
Johnson (2020)	34%	0%	Page 1764
Leos‐Urbel (2014)	73%	0%	Page 896
Lynch and Kim (2017)	60%	0%	Page 44
Modestino and Paulsen (2019a)/Modestino (2019b)	84%	0%	Page 10
Somers et al. (2015)	92%	0%	Page 22
Torgerson et al. (2014)	63%	0%	Page 13
Valentine et al. (2017)	67%	6%	Page 41
Wathington et al. (2016)	87%	0%	Table 3

*Source*: IES (2024).

It should be noted that what constitutes participation in the programme will differ by study, as does the information on this that can be used to transform impacts of allocation to impacts of participation. This will also lead to differences in the estimates of impact – a study with a looser definition of what constitutes participation (for instance, attending one session) evaluating one intervention would, assuming the intervention has some positive impact on an outcome which increases with the intensity of treatment, produce a lower estimate of the impact of participation than another study with a stricter definition evaluating the same intervention (for instance, completing the entire programme).

The results of different studies evaluating the same intervention are combined together to prevent interventions that have been studied frequently (such as the New York City Summer Youth Employment Program) from being overrepresented in the analysis. The results are combined through meta‐analysis. This approach was also employed when combining results for narrow outcomes within a broader outcome measure to be evaluated. The sample size taken from these analyses, should it differ across studies/outcomes/time points, was a weighted average with the weights coming from the random‐effects model estimate. Results for the same outcomes within studies over time were generally bundled together, through meta‐analyses as described previously, except in cases where using the longest follow‐up was logical, for instance if the outcome related to the completion of something such as higher education.

Four studies (groups of studies across different interventions) were set as the threshold required to run a meta‐analysis. Meta‐analysis can be performed with a minimum of two studies, however this will have limited power and given the wide range of outcomes of interest to the study, performing a full meta‐analysis for every outcome evaluated by just two studies would dilute the attention paid to outcomes with a greater number of studies and thus more powerful results. Four studies (i.e., the minimum number required to potentially contain two summer education and two summer employment programmes) was thus set as the minimum number of studies to run meta‐analysis. Should the impact of allocation to or participation in a summer programme be evaluated by two or three studies, the estimates of impact are converted to effect sizes and discussed narratively. If only one study reports estimates of the impact of participation in a summer programme no no t requiring transformation, this is summarised narratively and a meta‐analysis including this and transformed estimates of the impact of allocation to a summer programme is not performed unless, by doing so, this takes the number of studies over the threshold set to perform meta‐analysis (see below). Statistical significance is, as default, the 95% confidence level, unless specified otherwise.

To explore heterogeneity in effect sizes across studies, we perform sub‐group analyses. As well as exploring differences in impact between summer education and employment programmes, a central objective of this research, other sub‐groups used are: whether the intervention occurs in the UK or not (aside from one intervention that occurs in New Zealand, all other interventions occurs in the US); the specific intervention type; whether the summer programme component is part of a wider intervention (i.e., an ‘in part’ summer programme) or whether the intervention is focused on the summer component entirely (i.e., an ‘in whole’ summer programme); whether the intervention targeted various measures of disadvantage; and, the quality of the study's design. Additionally, where a sufficient number of studies evaluated an outcome (10, the minimum number recommended by the Cochrane Handbook) we also perform meta‐regression.

The moderators included as standard in the meta‐regressions are dummies equal to one if the intervention occurs in the UK, the intervention was a summer education programme, the intervention was an ‘in whole’ summer programme, and whether the study design was high‐quality by default. Dummies equal to one for each of the forms of disadvantage targeted by the intervention are also included should there be statistically significant differences between interventions that do and do not target them, identified through the sub‐group analysis, except where there is a high degree of collinearity between these sub‐groups in which case only the most statistically significant of these is included.

Multiplicity is a common issue in systematic reviews. The Cochrane Handbook for Systematic Reviews of Intervention does not recommend adjusting for multiple tests, for instance in the sub‐group analysis, although it is an issue worth considering. We conduct the same sub‐group analyses for each outcome that is subjected to meta‐analysis and for which study heterogeneity was identified through tests. Approximately 1 in 20 independent statistical tests will be statistically significant due to chance alone when there is no real difference between the groups (Higgins, et al., 2022) – given the number of outcomes and sub‐group analyses that we test, this issue should be borne in mind.

Given the large number of studies surfaced by the review and the high degree of commonality in the specific outcome measures evaluated, we perform the analysis and produce average effect sizes across specific outcome measures (e.g., likelihood of applying to higher education) as opposed to selecting the most relevant/most commonly used outcome measures to represent each broad outcome domain, for which we would then have performed the analysis and produced average effect sizes. The specific outcome measures of interest that feature in the synthesis were determined by first splitting all the outcomes evaluated across the five broad outcome domains of interest and then within these, further splitting the outcomes into common groupings. This was an iterative process, with several revisions made to the groupings. All outcomes or closely related outcomes that were evaluated by multiple studies are included in the synthesis.

### 10.3 Specific decisions made in preparing data for meta‐analysis

In addition to following the general principles outlined above in preparing the data for meta‐analysis, several specific decisions and/or assumptions were made. Table 6 details these – the problem that required solving, the solution, and the reasoning behind this.

**Table 6:** Specific solutions to issues encountered in preparing data for meta‐analysis.

Study	Problem	Solution and reasoning
Garcia et al. (2020) and Gehring et al. (2018)	Missing sample size split between treatment and control.	Assume 50–50 split as study design is an RDD.
Lynch and Kim (2017)	Missing sample size split between treatment and control.	Assume even split between treatment arms as study design is an RCT.
Herrera et al. (2013)	Missing sample size split between treatment and control.	Take from Garcia et al. (2020) which evaluates same intervention and same sample, assume one less in total sample size is from treatment group.
Theodos et al. (2017)	Missing standard deviation for all non‐ITT results.	Assume same as the one derived from the respective ITT results.
Martin et al. (2013b)	Missing standard deviation and insufficient information to follow fully other potential solutions.	Apply method from Walter and Yao (2007) assuming that the range of the observed results (which is not reported) covers the range of potential results on the scale.
Modestino (2019b)	Missing prevalence of outcome among treatment and/or control group for passing tests outcome.	Assume 50% of control group passes MCAS English language/mathematics test, based on Schliemann et al. (2022).
Schwartz et al. (2021)	Missing prevalence of outcome among treatment and/or control group for passing tests outcome.	Use pass rate for treatment and control groups combined, then apply treatment effect to this to estimate adjusted treatment group outcome.
Martin et al. (2013b)	Missing mean of outcome among treatment and/or control group for education skills, confidence and self‐efficacy outcome.	Assume control group mean equal to constant from regression.
Kessler et al. (2022)	Not clear whether bracketed figures reported below control means in result tables are standard deviations or standard errors.	Assume they are standard deviations given magnitude in relation to mean figures.
Herrera et al. (2013)	Report dichotomous measures of education engagement/participation/enjoyment whilst all others report continuous measures.	Construct log odds ratio and then convert to SMD using $\mathrm{SMD}=\mathrm{Log}\times \frac{\sqrt{3}}{\pi }$.
Modestino and Paulsen (2019a)	Report dichotomous measures of socio‐emotional engagement/skills whilst all others report continuous measures.	Construct log odds ratio and then convert to SMD using $\mathrm{SMD}=\mathrm{Log}\times \frac{\sqrt{3}}{\pi }$.
Heller (2014), Davis and Heller (2020) and Heller (2022)	Report number of days attended school whilst other studies evaluating outcome report attendance rate.	Assume maximum number of school days is 178, based on Illinois State Board of Education (2022).
Davis and Heller (2020)	2013 sample ineligible as for them intervention does not take place during transitional summer.	Use results from 2012 sample only.
Robles (2018)	Report results from multiple specifications.	Use results from 3‐nearest‐neighbours specification as nearest neighbour matching models have generally lower bias than inverse propensity score weighting, and 1‐nearest‐neighbour matching is done without replacement reducing common support.
Heller (2014)	Report results from multiple specifications.	Use results from the means and 0 s imputed model to minimise any potential bias introduced by missing data.
Heller (2022)	Report results from multiple specifications.	Use results from the means model to minimise any potential bias introduced by missing data.
Mariano and Martorell (2013)	Report results from multiple specifications.	Use results from English language arts/mathematics only groups as these produce the most logical comparison at the discontinuity; use results from fixed effect model as it best fits the data.
Johnson (2020)	Report results from multiple specifications.	Use results from DDD which reduces bias associated with unobserved characteristics.
Wachen (2016)	Report results from multiple specification.	Use results from linear regression model as results from logistic regression model result in highly asymmetric confidence interval when transformed to log odds ratio.
Henson (2018)	Report result for impact of allocation on progression to HE is logistic regression coefficient which cannot be transformed to impact of participation.	Calculate proportion of treatment and control individuals progressing to HE based on odds ratio (exponentiated coefficient), using this not the original odds ratio to calculate effect size (difference in figure is marginal whilst increasing relatability across impact types), take treatment effect as difference in proportions and transform this to estimate impact of participation, from which construct effect size.
Cohodes et al. (2022) and Robles (2018)	Cohodes et al. (2022) evaluates three forms (six‐week, one‐week, online) of the intervention, Robles (2018) evaluates one of these (six‐week).	When both studies are included in any analyses, the results from Robles (2018) are combined with the results from Cohodes et al. (2022) for the treatment arm that they both evaluate, before then combining the results across the different treatment arms evaluated by Cohodes et al. (2022).
Cohodes et al. (2022) and Robles (2018)	Evaluations of the same intervention have differing study design quality.	Use Cohodes et al. (2022) rating (high) as this is the larger of the studies and produces more result estimates.
Lynch and Kim (2017)	Evaluates two arms of the same intervention – one that is ‘in whole’ a summer programme and one that is the summer programme plus the provision of a laptop.	Exclude results for the summer programme plus laptop arm, as these are least representative of the true impact of the summer programme.

*Source*: IES (2024).

To explore the sensitivity of the results to the quality of studies included in the analysis, as previously mentioned, one of the sub‐groups we analyse is based on the study design quality assessment from the risk of bias assessment.

### 10.4 Reporting/publication bias

**Figure 3:**
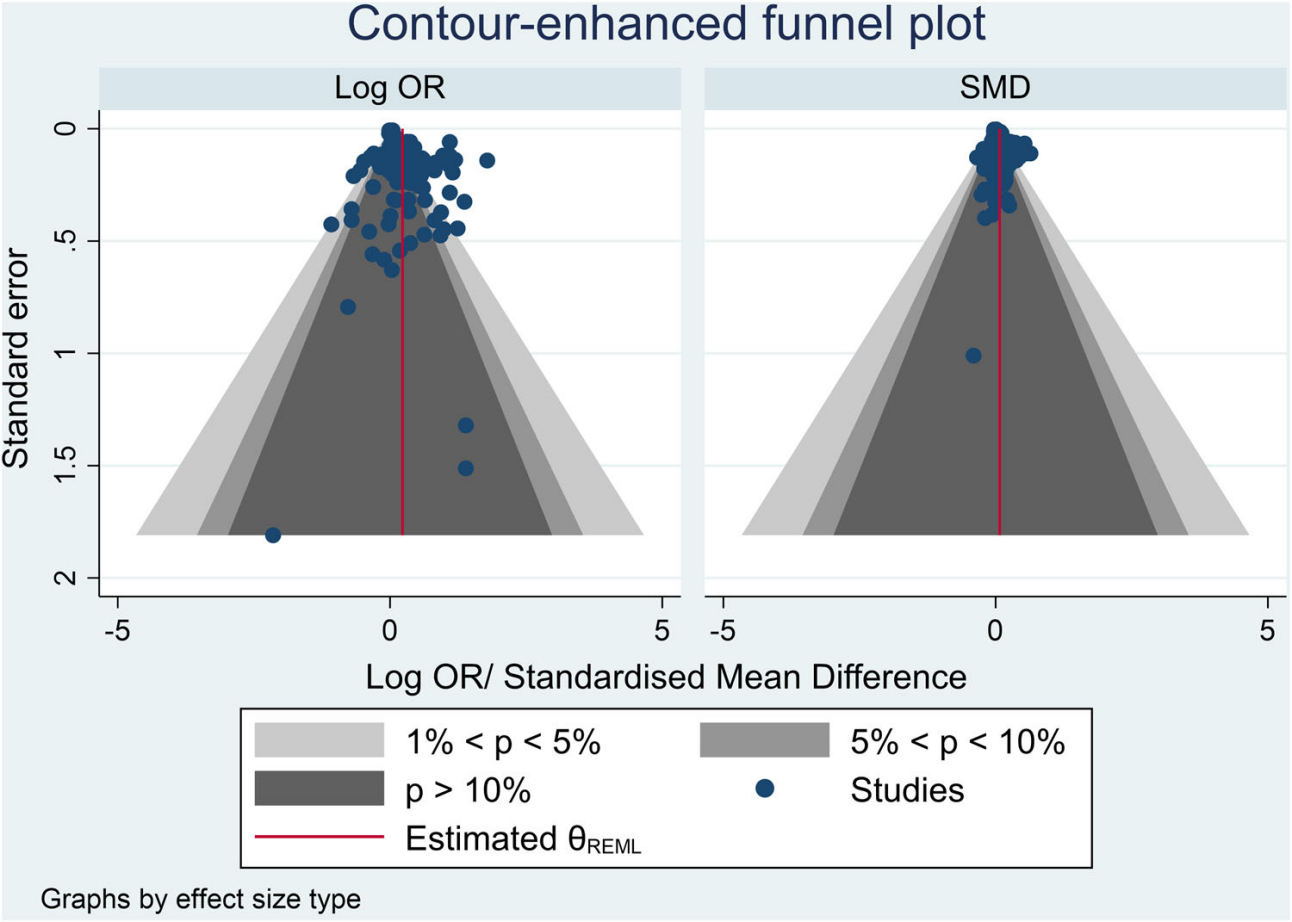
Funnel plot of effect sizes (log odds rations and standardised mean differences). *Source*: IES (2024).

Figure 3 displays the funnel plots of all of the log odds ratio and standardised mean differences used in the analysis that follows. Results for which it would be desirable for the effect size to be negative, for example, number of arrests, had their signs inverted.

The funnel plots of both the log odds ratios and standardised mean differences are relatively symmetrical, with effect sizes fairly evenly distributed either side of the estimated central effect sizes. There are also a relatively large number of studies that produce negative effect sizes. The majority of studies have relatively small standard errors, and those outlier smaller studies with larger standard errors generally fall under the pseudo confidence interval region with what appears to be a somewhat random distribution. These plots thus suggest an absence of publication bias and heterogeneity in effect sizes by study size.

## 11 APPROACH TO THEMATIC SYNTHESIS – DATA EXTRACTION AND STRUCTURING

Since this systematic review also seeks to identify components and features shared across and between successful summer programmes (and specific to employment separately from summer education programmes), we attended to qualitative evaluations of the interventions examined in the included studies.

We piloted our approach through team workshops and pilot coding to ensure our understandings of contexts, causal mechanisms, and facilitators and barriers were shared, and fixed onto a plausible causal pathway. A code book was developed to guide our extraction of studies. We piloted coding and held team reviews to ensure there was sufficiency in the detail coded to support later analysis. Using the codebook means that studies were coded according to a predefined theoretical framework (Thomas & Harden, 2008). However, it is important to note that not all codes and themes can be predefined; it is inevitable for each coder to extract inductive themes in the process (Harden & Thomas, 2005). We facilitated this by adding subcodes both in initial coding and then in later synthetic analysis to deepen understanding through a dedicated Microsoft Teams channel. While, thematic synthesis introduces a significant level of subjectivity into the analysis, this was reduced by making the process more transparent by including information in the codebook on whether each code was predefined or defined inductively, as recommended by Fereday and Muir‐Cochrane (2006).

The thematic synthesis for this systematic review proceeded according to the steps below, as outlined by Thomas and Harden (2008):

(1) Line‐by‐line coding.
(2) Organising codes along a hierarchical coding structure.
(3) Interpreting analytical themes.

To achieve the final step, we interrogated the Excel workbook codes on each main theme and the related subthemes, drawing and testing patterns within each to arrive at a synthesis of key issues as well as factors that outlie these. Team workshops throughout enabled discussion and consensus building to ensure consistency in approach. Two members of the team collaborated to interrogate the data against each theme, to act against bias emerging as data was broken down by programme, population and outcome type. The Excel framework and coding provided the underpinning evidence for the decisions reached.

The qualitative analysis also served the key purpose of enabling us to capture information related to the causal pathway, assumptions, moderators and contexts, as well as subgroups. Some of the themes that emerged from the review included the role of targeting and marketing to support positive engagement, engagement with new or different environments, people, and experiences, and the role played by relationships built with staff in building confidence to transition to and succeed in positive destinations. This process also allowed us to identify implementation issues and insights into how results could be replicated in practice, including what the critical success factors are for delivery.

There were various commonalities in the features of the different types of summer programmes surfaced. Based on these, we identified clusters of the specific type of intervention that the summer programme constituted. We use this cluster approach as one way of structuring the thematic synthesis, to examine whether the differences in the contexts of, and mechanisms used by, these different intervention types result in differences in the outcomes achieved. We identified three distinctive clusters for summer education programmes, alongside a single cluster of workplace exposure for summer employment programmes. The distinctive intervention types identified for summer education programmes are:

▪ **Catch‐up programmes**: The primary goal of catch‐up (or ‘remediation’ in the US context) programmes is to address attainment gaps and prevent summer learning loss among students. Catch‐up programmes typically target students who may be falling behind academically or are at risk of not meeting grade‐level expectations. These programmes focus on improving student's academic skills, filling knowledge gaps, and providing targeted instruction in specific subjects, usually core curriculum subjects, such as literacy, mathematics, and science. Catch‐up programmes make up one‐fifth of the interventions in the review. The majority of catch‐up programmes in our review take place in the US (6 programmes) or UK (5), with one in New Zealand. In terms of targeting, programmes are evenly split between those targeting young people mainly based on academic underperformance, and those that target using socioeconomic disadvantage indicators alongside academic performance. Most programmes target secondary school students, with three programmes targeting primary school students as well, and two programmes targeting students in their first year of higher education. Two US programmes specifically target ethnic minority students, both have a majority of Latino and Hispanic participants, while one UK programme targets speakers of English as a second language.
▪ **Raising aspirations programmes**: Raising aspirations programmes aim to inspire and motivate young people to pursue the next stages of education, usually higher education, or explore various career paths. These programmes often target students from underrepresented backgrounds (e.g., first‐generation, ethnic minority) or those who may have limited exposure to education opportunities. Raising aspirations programmes focus on increasing students' awareness of the next stages of education (secondary, higher) and advanced disciplines, providing exposure to different academic disciplines, and building confidence and self‐belief in their ability to pursue higher education and advanced careers. Raising aspirations programmes make up just above two‐fifths of the interventions in the review (44%) The majority of raising aspirations programmes in our review take place in the UK (14 programmes), with the remaining programmes in the US (7). In terms of targeting, the majority of the programmes (11) target young people based on academic underperformance, socioeconomic disadvantage indicators (7) as well as SEND (1) and being first‐generation (2). Most programmes target secondary school students in Year 10 to Year 12, with two programmes targeting primary school students in Year 5. Ten programmes specifically targeted ethnic minority students. Of these, nine were in the US and targeted a majority of African American and Latino students, and two programmes had a majority of Asian students.
▪ **Transition support programmes**: transition support programmes aim to facilitate a smooth transition for young people from one educational level to another, such as from primary to secondary school or from secondary school to higher education. These programmes primarily target students who are transitioning between different educational stages and may need support in adapting to new environments or expectations. Transition support programmes focus on helping students familiarise themselves with new learning environments, acquire essential skills for the transition, and provide support and guidance during this period of change. Transition support programmes make up around one‐third of the interventions in the review. The majority of transition support programmes in our review take place in the UK (8 programmes), with the remaining programmes being based in the US (6). In terms of targeting, the majority of programmes target using socioeconomic disadvantage indicators (11), and the rest target based on academic underperformance (5) and being first‐generation (1). Most programmes are targeted to students transitioning from primary to secondary school (9), with some targeted to those transitioning from secondary school to higher education (5), and one programme targeted to those moving from middle school to high school. Three transition support programmes specifically targeted ethnic minority students. These programmes are all based in the US and two included a majority of African American students, while one had a majority of Latino students.

In addition to the intervention type clusters, we also examine differences in the contexts, mechanisms and outcomes of different interventions/studies based on other characteristics – as with the quantitative synthesis, we examine commonalities and differences in these, based on whether the intervention occurs in the UK or not and whether the intervention targeted various measures of disadvantage. We do not consider differences based on whether the intervention is an ‘in whole’ or ‘in part’ summer programme as we are only interested in features of the summer programme component and this sub‐group is used in the meta‐analysis, only to examine whether the outcomes of ‘in part’ programmes may result in overestimation of the impact of summer programmes. Supporting Information: Appendix 2 details the clusters to which each study and/or intervention belong.

## 12 DEVIATION FROM PROTOCOL

There was only one minor deviation from the approach specified in the review protocol (Muir et al., 2023) to that implemented in the review. It was specified that only one vote would be required to exclude studies during screening, that is, the Cochrane method, however during the review two votes to exclude were required – this only adds to the thoroughness of the review process.

Various decisions regarding the exact implementation of the specified approach had to be made, although these decisions were written into the protocol. All specific decisions made in implementing the approach outlined in the protocol have been detailed in the relevant sections.

## 13 DESIGN FEATURES OF SUMMER PROGRAMMES AND THEIR THEORIES OF CHANGE

### 13.1 Summary of findings

▪ Summer education programmes typically target students who would benefit from additional academic support either because they are currently falling behind their peers academically or because they have characteristics that are associated with poorer future outcomes (such as coming from low‐income backgrounds, from areas with relatively poor higher education participation, or from groups that are underrepresented in higher education). Summer employment programmes typically target those from specific communities facing high disadvantage (high rates of poverty, unemployment, urban violence, or being at risk of not transitioning to higher education or meaningful work).
▪ Most summer education programmes in the review tend to target a ‘grey middle’, that is, students with poor performance but who may still be meaningfully supported by a developmental summer education programme. Young people with the poorest performance, who are often also those with the highest vulnerabilities, tend to receive more specialised and/or intensive alternative support outside of a summer education programme.
▪ Summer education programmes use referrals from schools and academic staff, and parent involvement as part of their recruitment approach. Summer employment programmes use outreach strategies and awareness‐raising by school staff, non‐profit and community‐based organisations.
▪ There are various components to summer education programmes, both academic (academic classes, homework help, mentoring sessions) and social or enrichment activities (e.g., team building, arts, sports, and creative writing, field trips, career‐shadowing opportunities, and community service projects). Employment programmes provide paid work placements or subsidised jobs in entry‐level roles with local non‐profit and community‐based organisations, government agencies and for‐profit businesses. Participants typically work around 25 h a week and access pre‐work training, mentoring, and employability skills support.

### 13.2 Structure of summer education programmes

#### 13.2.1 Delivery period and duration

Summer education programmes included in this review take place between July and September (these are the summer months, when summer breaks commonly take place, in countries of the northern hemisphere, including the UK and US where studies for this review are concentrated). In the UK and US, some transition support programmes are offered either at the very beginning of summer vacation (Day et al., 2013b), to promote sustained engagement, or right before the start of new academic year, to facilitate the transition process (Anthony, 2019; Henson, 2018). Other summer education programmes are more diverse in terms of when they take place.

Programme durations vary: short summer programmes, of several days or up to 1 week, are more frequent in the UK (Burgess et al., 2021; Church, 2018; CooperGibson, 2022; Hayes et al., 2018; Lei et al., 2020; Sharp, 2018; Thompson et al., 2017) than the US where programmes tend to last between 2 and 6 weeks (Garcia et al., 2020; Henson, 2018; Herrera et al., 2013; Johnson, 2020; Mac Iver & Mac Iver, 2019; Mariano & Martorell, 2013; McEvoy, 2012; Robles, 2018; Snipes et al., 2015; Somers et al., 2015; Wachen et al., 2018). In the UK these can take the form of short residentials which are not common in the US. However, there is one example of a residential summer programme in the US taking place over 5 weeks (Wachen et al., 2018). Catch‐up programmes are similar in duration in the UK and US. They tend to last 4 to 5 weeks, delivered over 4 to 5 days per week for around 5 h per day (Gorard et al., 2014; Gorard et al., 2015; Mariano & Martorell, 2013; McEvoy, 2012; Snipes et al., 2015; Somers et al., 2015; Torgerson et al., 2014).

#### 13.2.2 Targeting approach

Summer education programmes tend to target students identified as being most able to benefit from additional academic support, such as those who come from low‐income backgrounds, with lower academic attainment, or from areas with relatively poor higher education participation. Programmes also commonly target young people experiencing other forms of disadvantage, including those from ethnic minority backgrounds (Cohodes et al., 2022; Cosentino et al., 2015; Robles, 2018), disabled young people or those with a health condition (CooperGibson, 2022; Lei et al., 2020), students from the first‐generation in the family to attend university (Anthony, 2019; Cohodes et al., 2022; Henson, 2018; Robles, 2018; Smith et al., 2013; Wachen et al., 2018), students in care (Kettlewell & Aston, 2014a; 2014b; Lamont et al., 2014; Martin et al., 2013b), immigrant students (Johnson, 2020), and young people at risk of or with a history of offending (Tarling & Adams, 2012). Some programmes target areas that have generally poor academic performance, but then select individuals within those areas that have high academic performance (Hoare & Mann, 2012; Smith et al., 2013). In both the US and the UK, summer education programmes target disadvantage at the individual, family, school or neighbourhood level (Church, 2018; Day et al., 2013b; Ferguson, 2018; Gorard et al., 2015; Herrera et al., 2013; Horton & Hilton, 2020; Lynch & Kim, 2017; Maxwell et al., 2014; McEvoy, 2012; Thompson et al., 2017).

It should be noted that some programmes have hard criteria that participants need to meet to be eligible, usually around socioeconomic status (e.g., coming from a neighbourhood with a high deprivation index) or academic performance (i.e., performing at/under a certain grade level), while others take a more holistic approach whereby the combination of a range of disadvantage factors may be taken into account, when deciding which young people get offered a place.

It should also be noted that most programmes in the review tend to target a ‘grey middle’, when it comes to academic performance, meaning students with poor performance but who may still be meaningfully supported by a developmental summer education programme. Young people with the poorest performance, who are often also those with the highest vulnerabilities, are often already recipients of more specialised and/or intensive alternative support outside of a summer programme.

Summer education programmes in the US tend to have a stronger academic focus, with a higher concentration of catch‐up, alongside transition and raising aspirations programmes aimed at supporting increased participation in higher education and STEM (science, technology, engineering and mathematics) subjects. There is a stronger tendency in the US, compared to the UK programmes, to target first‐generation (Anthony, 2019; Cohodes et al., 2022; Henson, 2018; Robles, 2018; Wachen et al., 2018) and ethnic minority students (Cohodes et al., 2022; Cosentino et al., 2015; Lynch & Kim, 2017; Robles, 2018). In the UK, programmes tend to look at wider social mobility factors, and targeting is more likely to be based on areas of socioeconomic disadvantage and where progression in education is low (Church, 2018; Day et al., 2013b; Ferguson, 2018; Gorard et al., 2015; Horton & Hilton, 2020; Maxwell et al., 2014; Thompson et al., 2017). Similarly, while both countries have programmes targeting transitions, the focus differs in each. In the UK, there is a strong emphasis in the literature surfaced on transitions from primary to secondary school (CooperGibson, 2022; Day et al., 2013a; 2013b; Martin et al., 2013a; 2013b; Maxwell et al., 2014; Sharp, 2018; Siddiqui et al., 2014), whereas US programmes most often target transitions from secondary to higher education – college ‘bridge’ programmes are common (Anthony, 2019; Barnett et al., 2012; Garcia et al., 2020; Gehring et al., 2018; Henson, 2018; Wachen et al., 2018).

There is a further distinction key to the way different programmes target eligible participants. Several programmes target individuals within a specific geographic area, community, or school district with high levels of disadvantage. Programmes that target disadvantage mainly by area (in terms of academic performance and socioeconomic status) are more concentrated in the UK. Specific criteria used include the proportion of students in a school eligible for Free School Meals (FSM) (Maxwell et al., 2014; Smith et al., 2013); the National Collaborative Outreach Programme (NCOP) (Church, 2018; Horton & Hilton, 2020) and the Participation of Local Areas in higher education (POLAR) (Ferguson, 2018) – two area‐based indicators of low rates of progression to higher education; and the index of multiple disadvantage (IMD) (Taylor, 2022).

In the US, programmes rely far more on targeting disadvantage using individual‐level criteria, particularly by targeting young people with relatively poor academic performance based on the individual's grades and/or test scores (Barnett et al., 2012; Gehring et al., 2018; Ghazzawi et al., 2022; Mac Iver & Mac Iver, 2019; Mariano & Martorell, 2013; Snipes et al., 2015; Somers et al., 2015; Wathington et al., 2016), those eligible for federal subsidies such as the Pell Grant (Anthony, 2019; Ghazzawi et al., 2022; Wachen et al., 2018), or who are from groups under‐represented in specific academic disciplines (Cohodes et al., 2022; Ghazzawi et al., 2022; Mac Iver & Mac Iver, 2019; Robles, 2018; Snipes et al., 2015).

There are then some distinctions in targeting between different types of summer education programmes. Catch‐up programmes tend to occur in schools located in socioeconomically disadvantaged areas, where students are at the highest risk of summer learning loss, and target students who are performing below the expected level or are at risk of falling behind academically (Gorard et al., 2015; Lynch & Kim, 2017; Williamson et al., 2020). By targeting schools in these areas, the programmes aim to provide educational opportunities to underperforming students who may otherwise have limited access to academic support. This can include learners of English as a second language or students who have been grade retained or are at risk of grade retention (the practice in the US whereby students repeat a grade level) (Johnson, 2020; Mariano & Martorell, 2013).

Transition support programmes tend to focus on supporting first‐generation and low‐income students, students from ethnic minority backgrounds, and those from rural areas, with the aim of supporting educational progression, particularly to higher education (Anthony, 2019; Day et al., 2013b; Henson, 2018; Martin et al., 2013b; Siddiqui et al., 2014). This is on the basis that students within these groups may lack external support systems in particular as they may have few, if any, relationships with people who have participated in higher education. Similarly, programmes aimed at raising students' aspirations are more likely to target disadvantaged learners who come from areas with lower‐than‐expected higher education participation rates (Hayes et al., 2018; Hoare & Mann, 2012; Kettlewell & Aston, 2014b; Taylor, 2022).

#### 13.2.3 Recruitment methods

Approaches to recruitment tend to be similar in the UK and US with referrals from schools and academic staff, and parent involvement playing a significant role. Common advertising/awareness raising methods include flyers, posters, letters to parents, information evenings for pupils and parents, and referrals from school staff.

For catch‐up programmes, which appear more widespread in the US than UK, recruitment methods include identifying eligible students from specific geographic areas or schools, often those with higher rates of underperforming students (Snipes et al., 2015; Somers et al., 2015; Torgerson et al., 2014). Information sessions are common to raise awareness among parents (Snipes et al., 2015; Torgerson et al., 2014). School staff play a crucial role as a source of information for eligible young people (Snipes et al., 2015; Wathington et al., 2016). In raising aspirations and transition support programmes, participants are often recruited through school referrals, or online application forms hosted on programme or university websites (CooperGibson, 2022; Herrera et al., 2013; Lei et al., 2020). Schools with higher rates of disadvantaged students are often targeted, and recruitment strategies include school visits, presentations, school assemblies, and word‐of‐mouth referrals (Cosentino et al., 2015; Ferguson, 2018). Many of these programmes identify parents as key, hence invitation letters, telephone calls, and face‐to‐face engagement through parent evenings, alongside use of multilingual teachers to remove barriers for parents with English as an additional language, support engagement (CooperGibson, 2022; Garcia et al., 2020; Henson, 2018; Martin et al., 2013b; Snipes et al., 2015).

### 13.3 Structure of summer employment programmes

#### 13.3.1 Delivery period and duration

Summer employment programmes that met the inclusion criteria for this review were found only in the US. They tend to last between six and 7 weeks – the duration of the summer break (Gelber et al., 2016; Lansing et al., 2018; Leos‐Urbel, 2014; Modestino & Paulsen, 2019a). Participants usually spend around 25 h a week on a work placement, alongside a training component, such as pre‐work training or employability skills (Gelber et al., 2016; Heller, 2022; Leos‐Urbel, 2014; Modestino, 2019b; Schwartz et al., 2021; Valentine et al., 2017). One programme, Urban Alliance, has a year‐round training component, which requires participants to attend employability workshops from late September to July, plus 3 to 6 weeks of pre‐work training before the work placement in the summer break (Theodos et al., 2017).

#### 13.3.2 Targeting approach

Summer employment programmes tend to prioritise individuals from specific age groups and communities, often those facing high rates of poverty, unemployment, urban violence, or being at risk of not transitioning to higher education or meaningful work (Davis & Heller, 2020; Modestino & Paulsen, 2019a; Reich, 2018; Sum, 2015; Theodos et al., 2014, 2017). Geographic targeting is common, where programmes concentrate their efforts on neighbourhoods with significant socioeconomic disadvantages including high levels of poverty and unemployment as well as high levels of violence, with a particular focus on neighbourhoods with high rates of crime and high‐violence schools (Davis & Heller, 2020; Sum, 2015).

Targeting based on academic outcomes is also common, with some programmes targeting students with lower attainment, or at risk of not transitioning to higher education or meaningful work (Theodos et al., 2014, 2017). Most programmes have a high proportion of ethnic minority participants, particularly African American and Hispanic participants – in many programmes, these groups make up over half of participants, although this is often a result of self‐selection and the overlap between ethnic identity and the forms of disadvantage used as eligibility criteria, rather than an explicit eligibility criteria (Gelber et al., 2016; Heller, 2014; Leos‐Urbel, 2014; Modestino & Paulsen, 2019a; 2019c; Modestino, 2019b; Reich, 2018; Sum, 2015; Theodos et al., 2014, 2017; Valentine et al., 2017).

#### 13.3.3 Recruitment methods

Recruitment strategies for summer employment programmes include school staff leading general or targeted group presentations, individual meetings with students, and sharing information in newsletters and on posters to raise awareness (Reich, 2018; Theodos et al., 2014). Non‐profit and community‐based organisations and partners also spread awareness through newsletters and outreach, and may support potential participants to complete applications (Valentine et al., 2017). As a result, participants may apply for the programmes through specific providers or intermediaries, sometimes contracted by the relevant local authority or government agency (Modestino, 2019b; Modestino & Paulsen, 2019a; Modestino, 2019b; Valentine et al., 2017).

The application period for summer employment programmes is generally in early spring, and there tends to be more applicants than places, which sometimes leads to lottery systems for selection (N.B., this might be specific to the studies included in this review, as evaluators may have selected programmes where this was the case to randomise treatment status, which may not be generalisable to all summer employment programmes) (Gelber et al., 2016; Heller & Kessler, 2017; Leos‐Urbel, 2014; Schwartz et al., 2021; Valentine et al., 2017). Other allocation methods include ‘first‐come first‐served’, merit‐based assignment, or meeting eligibility criteria based on specified disadvantage characteristics, such as coming from a high‐poverty or low educational progression area (Heller & Kessler, 2017; Lansing et al., 2018).

### 13.4 Features of summer education programmes

Summer education programmes tend to centre on offering additional instruction on core subjects, including mathematics, English, and science, or in subjects where participants may wish to pursue further studies, such as advanced STEM subjects. The latter is more so the case for raising aspirations programmes (Cohodes et al., 2022; Hayes et al., 2018; Mac Iver & Mac Iver, 2019; Robles, 2018) while catch‐up programmes have a stronger focus on core subjects where students need catch‐up support (Gorard et al., 2014; Gorard et al., 2015; Johnson, 2020; Mariano & Martorell, 2013; McEvoy, 2012; Somers et al., 2015; Wathington et al., 2016). There are various components to summer education programmes, such as academic classes, homework help, arts or recreation electives, and mentoring sessions. The programmes often include additional components covering social or enrichment activities (e.g., team building, arts, sports, and creative writing), field trips, career‐shadowing opportunities, and community service projects (Cohodes et al., 2022; CooperGibson, 2022; Gorard et al., 2014; Gorard et al., 2015; Herrera et al., 2013; Martin et al., 2013b; McEvoy, 2012; Siddiqui et al., 2014; Torgerson et al., 2014).

Catch‐up programmes, for which evidence is mostly available from the US, are primarily aimed to help students catch up. As such they are targeted at students who require additional academic support and extra instruction to meet the attainment level required to successfully progress to the next academic year and sustain their education. Programmes focus on academic skill‐building and catch up in specific subject areas, such as English and mathematics (Gorard et al., 2015; Johnson, 2020; McEvoy, 2012; Somers et al., 2015; Wathington et al., 2016). These programmes tend to employ highly structured and supportive learning environments, with strong focus on mentoring and small class sizes, to enhance learning and provide individualised support to struggling students (Garcia et al., 2020; Gorard et al., 2014; Wathington et al., 2016).

Transition support programmes provide a blend of academic instruction, guidance on educational readiness and success, and social integration and enrichment activities. They can incorporate elements that help students become familiar with the new learning environment, such as introductions to student services or faculty in the new educational setting, and campus tours (Anthony, 2019; Day et al., 2013b; Garcia et al., 2020; Henson, 2018; Lei et al., 2020; Martin et al., 2013a; 2013b). Academic instruction is often delivered in an accelerated format with contextualised and active learning (Barnett et al., 2012; Garcia et al., 2020; Siddiqui et al., 2014). The focus is on helping students develop essential skills for success in the next education phase. Transition support programmes can also include residentials, providing participants with an immersive experience on a university or other educational campus (Day et al., 2013b; Martin et al., 2013b; Wachen et al., 2018). In the UK studies, these programmes seek to balance academic support with social and enrichment activities (CooperGibson, 2022; Day et al., 2013b; Martin et al., 2013b; Siddiqui et al., 2014), while US programmes (which still include enrichment activities) place stronger emphasis on academic skill building and preparation (Anthony, 2019; Barnett et al., 2012; Garcia et al., 2020; Gehring et al., 2018; Henson, 2018; Wachen et al., 2018).

Raising aspirations programmes stress increasing students' motivation, engagement, and interest in education, particularly in pursuing higher education, and in careers in highly specialised fields such as STEM. These programmes aim to broaden students' horizons and provide holistic development. They typically provide combinations of academic learning, mentorship, enrichment, and exposure to campus life. Academic subjects such as mathematics and science may be covered, along with hands‐on classes and workshops, exposing students to real‐world applications of the academic content (Burgess et al., 2021; Church, 2018; Cohodes et al., 2022; Cosentino et al., 2015; Ghazzawi et al., 2022; Hayes et al., 2018; Herrera et al., 2013; Lawson et al., 2019; Mac Iver & Mac Iver, 2019; Pyne et al., 2020; Robles, 2018). The academic focus is often complemented by social activities, such as university visits and cultural activities, and independent time to allow students to connect with peers, engage in shared experiences, and build a sense of community (Burgess et al., 2021; Cohodes et al., 2022; Ghazzawi et al., 2022; Hayes et al., 2018; Herrera et al., 2013; Lei et al., 2020). As for transition support and raising aspirations programmes, US‐based programmes emphasise preparation for university admissions examinations and applying for financial aid, and there is a higher concentration of programmes focused on STEM, than in the UK (Cohodes et al., 2022; Ghazzawi et al., 2022; Mac Iver & Mac Iver, 2019; Robles, 2018).

A further distinction is where summer education programmes target young people from specific vulnerable groups, such as disabled young people, and those with a history of or at risk of offending. These programmes aim to support the retention of the target group in education. The UK‐based Bath Autism Summer school, for example, is a short, raising aspirations programme for young people aged 16 to 30 with a diagnosis of Autistic Spectrum Disorder (ASD) (Lei et al., 2020). This residential 4‐day programme, immerses students in independent living on campus which allows them to experience and practice independent living, preparing them for future transitions. It includes two overnight stays in student accommodation at a campus university and a 3‐day curriculum. The UK‐based Summer Arts Colleges programme, on the other hand, is an intensive, full‐time programme with a focus on deterrence. It is offered to young people aged 14–19 at high‐risk of offending, particularly those on Intensive Supervision and Surveillance Programmes (ISSPs) and recently released from custody. It aims to improve literacy and numeracy through alternative provision – a structured arts curriculum, including visits and activities aimed at raising awareness of work opportunities in the creative sector. Its purpose is to facilitate vulnerable young people's transition into mainstream education, training, and employment, and reduce levels of re‐offending during and after the programme (Tarling & Adams, 2012).

### 13.5 Features of summer employment programmes

Summer employment programmes provide paid work placements or subsidised jobs to participants. These offer participants a chance to earn income, usually close to the minimum wage set by the respective state or city for their work, while gaining valuable work experience and developing key skills for entry to employment. In these programmes, participants typically work an agreed number of hours, usually around 25 h a week. Wraparound support can be offered such as pre‐work training and employability skills support (see below) (Davis & Heller, 2020; Gelber et al., 2016; Heller, 2022; Kessler et al., 2022; Leos‐Urbel, 2014; Modestino & Paulsen, 2019a; Modestino, 2019b; Schwartz et al., 2021; Theodos et al., 2014; Valentine et al., 2017).

In summer employment programmes examined by this review, participants are predominantly offered entry‐level roles with local non‐profit and community‐based organisations, although some government agencies and for‐profit businesses are involved. Settings in which participants gain work experience include summer camps, day care centres, community‐based organisations, law firms, hospitals, museums, and schools, among others. Roles can include summer camp staff, community garden workers, school infrastructure improvement, and administrative support (Davis & Heller, 2020; Gelber et al., 2016; Heller, 2014; Kessler et al., 2022; Lansing et al., 2018; Leos‐Urbel, 2014; Modestino & Paulsen, 2019a; Schwartz et al., 2021; Valentine et al., 2017).

Alongside the work placement, most programmes require participants to take part in work‐related training. This can be pre‐employment training, before the start of job placements, for example, throughout the school year or in the spring months (Reich, 2018; Theodos et al., 2014, 2017), or alongside the job placement (Gelber et al., 2016; Leos‐Urbel, 2014; Modestino, 2019b; Schwartz et al., 2021; Valentine et al., 2017). Training tends to cover employability and work skills, including workplace safety, soft skills (such as dependability, communication, collaboration, and initiative), job search strategies, financial capability, completing online applications, drafting resumes, interview techniques, career exploration, post‐secondary education options, and workplace etiquette. In addition to general pre‐placement training some programmes, such as STEP‐UP, collaborate with the employers involved to provide training specific to the company, career exposure events, and industry‐recognised industry accreditations (Reich, 2018).

Summer employment programmes often offer a mix of coaching, mentoring, and support services as well, guiding young people throughout their work placement. Some programmes assign participants a job mentor or programme coordinator, acting as a coach, who supports young people to become successful employees and overcome barriers to employment. Young people receive job mentoring and general coaching from these staff at their work placement sites, and mentors and coaches help track their performance, including workshop and job attendance, punctuality, work progress, progression planning (where relevant), and progress towards achieving the summer employment programme requirements (Davis & Heller, 2020; Heller, 2014, 2022; Lansing et al., 2018; Modestino & Paulsen, 2019c; Sum, 2015; Theodos et al., 2014, 2017).

### 13.6 Features of summer programmes with high ethnic minority participant rates

A specific area of interest to the review's advisory group is the extent to which summer programmes may explicitly target ethnic minorities and address race equity issues, and whether such programmes that do have these characteristics display distinctive features compared to summer programmes which do not.

When looking at summer programmes with a focus on ethnicity, the review identified three categories under which they broadly fall: summer programmes which do not target participants based on ethnicity but have a vast majority (90% or more) of participants from ethnic minority backgrounds; summer programmes which include ethnicity alongside other characteristics in their eligibility criteria; and programmes which explicitly target ethnic minorities. It is important to note that the majority of programmes which either explicitly focus on ethnicity or have a vast majority of participants from ethnic minority backgrounds take place in the US and include for the most part, raising aspirations (7) and transition support (3) education programmes, and four summer employment programmes. In the UK three raising aspirations programmes had an explicit focus on ethnicity.

Taking the first group of summer programmes (those that do not target participants based on ethnicity but have a vast majority of participants from ethnic minority backgrounds), the evidence highlights that these programmes broadly share similar characteristics when it comes to targeting ethnicity (Barnett et al., 2012; Heller, 2014; Herrera et al., 2013; Mac Iver & Mac Iver, 2019; McEvoy, 2012; Pyne et al., 2020; Reich, 2018; Sum, 2015; Theodos et al., 2014, 2017; Wachen et al., 2018). These are programmes, both in education and employment, which target based on area‐based disadvantage (areas with high poverty or unemployment rates, or low attainment levels or low rates of progression in education). They do not have an explicit focus on ethnicity in their targeting, and the high rate of ethnic minority participants results from the intersection between ethnicity and the other forms of disadvantage targeted. These programmes do not display any distinctive characteristics when it comes to considerations of race equity and share similar features and mechanisms with other programmes in their clusters (transition support, raising aspirations, workplace exposure).

In the second group are summer programmes (all summer education programmes) which include ethnicity among other criteria for programme eligibility, for example being a first‐generation student, coming from an area or school with low rates of progression to higher education, being disabled or having a health condition, being a carer or care‐leaver, and other recognised individual‐level disadvantage characteristics (Robles, 2018; Taylor, 2022; Thompson et al., 2017). In these summer education programmes, and particularly for raising aspirations programmes, it is sometimes the case that coordinators select participants based on a combination of elements, which can also include academic attainment and potential, rather than a single criteria (Robles, 2018). As for the first group, there are no distinctive features or mechanisms in these summer education programmes which can be linked to a focus on ethnicity. The programmes tend to share similar characteristics with other summer education programmes in the same cluster or are distinctive due to the nature or structure of the specific programme and their offer, rather than any element linked to considerations of race and race equity.

There are then three raising aspirations programmes which belong to the third group, those which explicitly target young people from ethnic minorities. The review identified three raising aspirations programmes that fall into this group (Cohodes et al., 2022; Cosentino et al., 2015; Ghazzawi et al., 2022). These three programmes share a key distinctive feature, which is the use of role models for mentoring, supervision, peer support, teaching staff, and alumni events, who are from similar backgrounds to the young people participating in the programmes. In the STEM summer programmes analysed by Cohodes, a key mechanism identified by the author are ethnic minority role models in the form of peer teaching and residential assistants, staff and instructors, and guest speakers, who work with participants on both an individual basis and as part of shared group experiences. In the Scholar Enrichment Program, Ghazzawi et al. (2022) identified career seminars and social events involving alumni in STEM fields as key mechanisms. Former programme students from similar backgrounds as current students are also recruited to serve as office aides, tutors, peer mentors and peer facilitators, as they advance through their academic careers, to keep participants engaged in the Scholar Enrichment Program community. In the Summer medical and Dental Education Program, Cosentino et al. (2015) identified that offerings such as mentoring and exposure to role models and inspirational speakers from similar backgrounds help enhance students' sense of self‐efficacy, promoting the message that if others from similar backgrounds overcame similar barriers, so can they.

Alongside this key feature identified across all three programmes that target ethnic minority students, a further feature which may support the participation and engagement of ethnic minority youth has been identified by Ghazzawi et al. (2022). They argue that financial assistance offered by the Scholar Enrichment Program programme, in the form of a stipend, coupled with the faculty support and mentorship provided to ethnic minority students, alleviates the stress of many ethnic minority students having to simultaneously support themselves and succeed academically through college. While this mechanism was identified as part of a programme explicitly targeting ethnic minority students, it should be noted that it is not exclusive to these types of programmes, and other summer education and employment programmes use stipends, wages, and financial aid as a mechanism to support engagement and participation. Ghazzawi et al. (2022) also identified a potential negative mechanism tied to disparities in educational achievement between African American and Hispanic Scholar Enrichment Program students. Scholar Enrichment Program participation is associated with higher final cumulative GPA and first‐year GPA of Hispanic students but not African American students. The author argues that given that the University of Houston, which delivers the Scholar Enrichment Program, has a large Hispanic population (it is designated a ‘Hispanic Serving Institution’), Hispanic students may feel a greater sense of belonging due to the presence of a large Hispanic community. This finding raises important considerations around race equity and ensuring that programmes aimed at supporting ethnic minorities avoid the risk of creating disparities between different groups.

Finally, an overarching consideration when it comes to race equity is to note that many raising aspirations programmes have a high rate of ethnic minority participants, even when they may not be explicitly targeting ethnic minorities. This may suggest that the inherent ethos and aim of these programmes, which target non‐traditional students and focus on building a sense of belonging and confidence to pursue opportunities which may not be otherwise accessible to them, as well as their wider features, support race equity ambitions.

## 14 IMPACT OF SUMMER PROGRAMMES

This section details the findings from the analysis of quantitative information, through meta‐analysis and/or narrative discussion, investigating the extent of any impact of summer programmes, summer education and summer employment programmes on disadvantaged or ‘at risk’ young people across the outcome domains of interest, and the extent to which this varies based upon study, participant and intervention characteristics.

Firstly, we summarise the main findings from the analysis by discussing the estimated average sizes of summer programmes, summer education programmes and/or summer employment programmes, or where meta‐analysis was not possible, summarising narratively the findings of impact from the literature. We only discuss the pooled findings from both summer programme types when there are not clear differences in findings between summer education and summer employment programmes, to avoid potentially attributing any impact to both summer programme types when this is not the case.

To guide readers as to which findings to have most confidence in, a judgement about the security of the findings has been provided: the precision of the estimated results; the number of studies evaluating the outcome; the study‐design quality of studies evaluating the outcome; the consistency of findings across studies; the similarity in specific outcome measures used across studies; and any other specific issues which might affect our confidence in the summary findings. This approach is based on the GRADE approach to grading the certainty of the evidence as recommended by the Cochrane Handbook (Schünemann et al., 2022), although we then use different criteria in the assessment given that this review is not focussed on a medical intervention, where the use of RCTs in evaluation is far more common and other elements of the approach (including, for instance, uprating based on the presence of a dose–response gradient) are more applicable. This judgement of either high, moderate or low confidence indicates our confidence that the headline finding (i.e., whether there was a significant impact or not, and if so its direction and magnitude) is indicative of a true underlying relationship between summer programme allocation/participation and the outcome, based on the literature included in the review. It is not a judgement on the finding itself; for instance, we can have high confidence that allocation to/participation in a summer programme has no impact on an outcome examined. In the summary table, we summarise succinctly the reasoning behind the decision.

After summarising the findings, we detail the specifics of the analysis of each specific outcome measure across the education, employment, violence and offending, socio‐emotional and health outcome domains. We first detail the studies (split across the interventions they evaluate) and outcome measures (including the relative point in time at which they are measured) that contribute to each of the analyses, noting instances where outcomes measures are based on self‐reported data or are collected as part of the evaluation, and in cases of the latter the extent to which attrition (overall and differential rates) may introduce bias into the impact estimates produced (based on the risk of bias assessment). Next, where meta‐analysis was performed, we discuss the overall findings by summer programme type. We then discuss whether heterogeneity in effect sizes is present. If the *p*‐value from any of the tests of homogeneity indicates the presence of heterogeneity (at the 90% confidence level given that each analysis is generally based on a small sample of studies), we highlight instances of significant difference between the sub‐groups of interest. Note as previously highlighted, given the small sample sizes used for the analysis, any significant differences on average effect size by sub‐group does not imply causal differences in impact between groups, rather this is at best correlational evidence. In all instances we also highlight any differences in effect size between studies of interventions that do and do not target ethnic minorities, given that issues of race equity are of specific interest to the review. Lastly, we then discuss findings from any sensitivity analyses performed. This is done for highly weighted or outlier effect sizes that may be driving the overall finding, or in instances where the specifics of the outcome measure used to construct the effect size means that the validity of including the result in question in the analysis warrants assessment. Note that the term *impact* is reserved for treatment effects that are statistically significant: in instances where an association between allocation to/participation in a summer programme that is not statistically significant is discussed, this is referred to an *effect* and the lack of significance is explicitly noted.

### 14.1 Summary of findings from the meta‐analysis and comparison of quantitative results

The majority of outcomes from summer programmes are observed within the education domain – this is to be expected given that 49 of the 68 included studies evaluate summer education programmes, but given the demographics of the population of interest these outcomes are also commonly evaluated by summer employment programmes. Additionally, as noted earlier, improving educational outcomes can support reduced engagement and likelihood of engagement in the criminal justice system, as well as support better employment outcomes.

Summer education programmes have a significant and moderately secure positive impact on *English scores* overall, equating to a 0.07 standard deviation improvement in scores. In contrast, a significant negative impact on English scores is estimated for summer employment programmes, with the difference on average effect size between summer education and employment programmes being statistically significant. However, this finding for summer employment programmes is driven by the estimate from Leos‐Urbel (2014) who finds that the intervention encourages lower ability students to take the elective, non‐universal test that is used to measure this outcome. It is therefore this ‘compositional’ effect, as opposed to the English attainment of treated individuals truly being lowered through allocation to the summer employment programme, that leads to a negative impact on English test scores.

There does not appear to be any significant impact on *reading and writing scores*. Three of the four studies evaluating both outcomes find larger effects or impacts on writing than reading scores: reading skills may tend to develop more gradually compared to writing skills, and therefore summer programme's short duration may not allow sufficient time for as considerable an improvement in this skill (Johnson, 2020). The findings relating to reading and writing scores are low‐confidence.

Summer education programmes do not appear to have a significant impact on *mathematics scores*, although there is statistically significant heterogeneity in the effect sizes behind this finding. Four of the five studies evaluating this outcome do not find significant impacts. Furthermore, the result from one study (Snipes et al., 2015) that finds particularly large impacts on mathematics scores plays a large role in determining the magnitude of the average effect size. One concern in interpreting the effect size from this study is that participants in the treatment group for this study were assessed based on diagnostic tests, rather than on norm‐referenced or criterion‐referenced state tests, in which improvement may be harder. When comparing student achievement on state tests for those who participated in the summer programme (located in the US) to those who did not, participants' achievement was still below typical achievement for students in the surrounding districts (Snipes et al., 2015). Attrition was a further concern in this study, as the baseline grade scores for mathematics in the treatment group were somewhat higher than those in the control group. While the analysis controlled for these differences, the possibility that unmeasured differences could have affected these estimates remains. As such, we can have moderate confidence that there is no impact on mathematics scores.

While insignificant, the central average effect size of summer education programmes on mathematics scores is approximately double that on English scores. However, when looking at the pool of studies that evaluate *both English and mathematics test scores*, there is an even split between those that find greater effect or impact on English scores than mathematics scores and vice versa. In studies that find a greater effect or impact on English, there is not sufficient evidence provided to understand why this may be the case. Where greater effects or impacts are found on mathematics scores, hypotheses explored by studies are that this could be due to a difference in the quality of the teaching observed, that mathematics is less susceptible than literacy to summer learning loss (Gorard et al., 2014), that students may be more engaged in the mathematics curriculum, or that English curricula may not be as effective with students below the expected level, compared to the mathematics curricula with students below the expected level (Somers et al., 2015).

Summer education programmes have a significant positive impact on *all forms of test scores*, equating to a 0.14 standard deviation improvement, although there is statistically significant heterogeneity in the effect sizes behind this finding. We have moderate confidence in there being a positive impact of allocation to a summer programme on all forms of test scores, as all studies except one evaluating this outcome find a positive association, although for only two of these do they find a significant positive impact. Summer employment programmes have no significant impact, which is fairly consistent across studies and cannot be explained, as before, by the specifics of the outcome measure used, as a range of measures including overall Grade Point Average are used by studies of summer employment programmes. The difference on average effect size between summer education and summer employment programmes is statistically significant.

*Completion of higher education* is an area where it is clearest that summer education programmes have an impact. Young people who participate in a summer education programme are 1.46 times more likely to complete higher education than those who do not, and we have relatively high confidence in the headline finding (i.e., a significant positive impact) for this outcome, although there is statistically significant heterogeneity in the effect sizes behind this finding. The three studies of summer education programmes that estimate above‐average effect sizes do so less precisely than the other two studies evaluating the outcome – were these studies to have been able to produce more precise estimates around the same central estimates of effect, the average effect size would have been higher. Participation in a summer employment programme has no statistically significant impact on the likelihood of completing higher education, although there is statistically significant heterogeneity in the effect sizes behind this finding. The difference on average effect size between summer education and summer employment programmes is not statistically significant at the conventional 95% confidence interval but is at the lower 90% confidence interval.

Allocation to summer education and summer employment programmes does not appear to have an impact on the likelihood that young people *progress to higher education*, although the average effect size for participation in any summer programme is positive and significant despite the average effect sizes for both summer education and summer employment programmes specifically on this outcome being insignificant. Additionally, there is statistically significant heterogeneity in the effect sizes behind these findings.

There are also generally significant positive impacts on *STEM‐related higher education outcomes*, including the likelihood of graduating higher education with a STEM degree or entering medical school.

Summer education programmes are also found to have a significant positive impact on *engagement with, participation in and enjoyment of education activities*, which equates to (using the specific measure of one of the studies evaluating the measures) approximately 60% of those allocated to the summer programme moving from never reading for fun to doing so once or twice a month after the programme. If anything, this may be an overestimate of the impact of summer education programmes, as the effect sizes constructed for three of the five studies evaluating the outcome are insignificant. As such, we have low confidence in this overall finding.

Several *other education outcomes* are assessed by multiple studies, although confidence in the security of findings relating to these is low. There are generally insignificant impacts of summer programme allocation/participation on self‐reported measures of education skills, confidence and self‐efficacy; the likelihood of passing tests (summer education programme may in fact have a positive impact on this outcome, although the wide variation in findings in part leads to no overall finding of significance); attendance rates in secondary education (the one evaluation of a summer education programme evaluating this outcome finds a significant positive impact, and one of the two studies evaluating chronic absence rates finds a significant beneficial impact); the likelihood of completing secondary education on‐time; the likelihood of applying to higher education (all three studies evaluating the outcome find positive effects, so if anything there may be a positive impact that is not borne out due to imprecise estimates); and the likelihood of experiencing a negative behavioural outcome (including chronic absence, unexcused absences, incidents, referrals, removals and suspensions).

The positive (although insignificant) average effect size of summer employment programmes on progression to higher education is interesting to note, especially when considered alongside the effect of summer employment programmes on *entry to employment*. While the negative effects in the short‐term and across the entire period evaluated are insignificant, there is an overall significant negative impact (at least in the short‐term) on the likelihood of being in employment unrelated to the summer employment programme. This is the finding from the two studies evaluating the New York City Summer Youth Employment Program, which find some significant negative impacts on post‐programme *earnings*, although these fade over time. These negative impacts are also conditional on being in employment, suggesting that the ‘quality’ of employment entered by those participating in the summer employment programme is generally lower than entered by those not participating. The overall finding is no significant impact on post‐programme earnings. There are mixed findings of no significant impact and a significant improvement in *job readiness* from summer employment programmes. Modestino and Paulsen (2019a) finds significant positive impacts of allocation to the summer employment programme on job readiness, which alongside a significant negative impact on individuals' self‐reported work intentions and a significant positive impact on individuals' self‐reported intentions to progress to higher education, may suggest that employment is a delayed outcome of summer employment programmes. Arguments provided to explain this are that early work experience provides exposure to new influences (introductions to new occupations, different adult mentors, wider networks, etc.) that can help young people shape their goals. This can in turn raise both career and academic aspirations, with both leading young people towards further participation in education as opposed to more immediate entry into the labour market (Modestino, 2019b). Furthermore, by enabling participants to shift their work experiences to a part of the year when they are not also attending school, summer employment programmes might enable young people to increase the time and attention that they can devote to academics during the school year and reduce their need to work in the post‐programme period (Modestino & Paulsen, 2019a). In addition, young people may gain job readiness skills in the summer but because they are in school during the following year, these short‐term outcomes may not translate into longer‐run improvements related to employment, if they choose not to work while in school (Modestino & Paulsen, 2019a). Evidence for another summer employment programme argues that given the high rate of successive college attendance among programme participants, positive labour market outcomes are likely to develop over a longer timespan (Theodos et al., 2017). To put these findings in context, it must be noted that not detecting a positive impact of summer employment programmes on labour market outcomes is consistent with the findings from Card et al. (2010) in their meta‐analysis of active labour market programmes. As there is wide variation in effect sizes among those studies evaluating the entry to employment outcome (note that there is statistically significant heterogeneity in the effect sizes behind the overall findings for entry to employment in both the short‐term and full period evaluated), we have low confidence in these findings as well as those for earnings and job readiness. Furthermore, some of the evidence behind these findings is weak: the employment‐related outcome data from Modestino and Paulsen (2019a) is based on self‐reported survey measures with highly differential response rates between the treatment and control groups.

Studies evaluating summer employment programmes have mixed findings of either no impact or substantial reductions in *violence and offending outcomes*. The evidence base perhaps provides more support for impacts in relation to violent criminal/offending behaviour, and during the programme months, although the wide variation in findings and limited number of studies that assess these outcomes lowers our confidence as to the overall findings.

Two of the three studies of summer education programmes that assessed *socio‐emotional outcomes* find positive impacts, whilst the other finds no impact. Among studies of summer employment programmes evaluating these outcomes, one finds a positive impact across both measures they use, whilst the other one finds no impact. The two studies evaluating summer employment programmes find beneficial impacts on *health‐related outcomes*, although the specific aspects of health measured by the two studies differ substantially. The evidence base is particularly limited for these wider outcomes.

There are some common themes that emerge from the *sub‐group analyses*, although these analyses come with some caveats. They are characterised by small sample sizes and are often underpowered. This limits the ability to perform meta‐regressions that can account for multiple points of difference between studies when estimating the difference that a study/intervention having a certain characteristic has on the estimated effect size. Alongside this, there is an often high degree of overlap between sub‐groups that affects our confidence in the findings about heterogeneity in effect sizes, as both known and unknown moderators may be confounded with the sub‐groups that we do test. As such, the sub‐group analyses are correlational, and we do not seek to make any claims about causality in relation to them.

Nonetheless, and generally, programmes that target participants by area (such as by targeting socioeconomically disadvantaged areas or areas containing a high proportion of individuals with experience of or at risk of involvement with the criminal justice system) have a relatively weak level of impact (there is one case where these programmes outperform those that do not target socioeconomically disadvantaged areas, which is the impact of summer programme allocation on engagement with, participation in and enjoyment of education activities). Conversely, those that target participants based on individual‐level characteristics (such as individuals with relatively poor academic performance) have a relatively strong level of impact. This might suggest that tighter targeting of those individuals facing individual level as opposed to structural barriers to more positive outcomes that might be able to benefit most from a summer programme may alter its average effectiveness, affecting the case for provision of the intervention.

There are limited differences in findings between programmes that do and do not target individuals from ethnic minorities, although it is worth noting that all the programmes that evaluate higher education outcomes relating to STEM subjects, which generally find positive significant impacts, all target individuals from ethnic minorities. Catch‐up programmes have among the largest impacts on test scores, whilst transition support programmes have among the largest impacts on the likelihood of attending and completing higher education. There are no clear indications of whether summer programmes ‘in whole’ compared with those ‘in part’ have greater benefits. ‘In part’ summer programmes have significantly larger average effect sizes than ‘in whole’ summer programmes for some outcomes for example, related to the impact of participation on all test scores. The meta‐regression for all test scores, which controls for differences in other factors including the programme type and location, the forms of disadvantage targeted and the quality of the study design, found that ‘in whole’ summer programmes have a significantly lower average effect size than ‘in part’ summer programmes. However, this is not always the case and does not hold true, for example, for the impact of summer programme allocation on progression to higher education. Furthermore, there is no clear evidence that studies with high‐quality study designs find higher or lower average effect sizes than studies with lower quality study designs indicating that our findings on the whole are probably not driven by the quality of the evidence, although on a case‐by‐case basis this may have an effect.

It should be noted that whilst for an impact evaluation to be eligible to be included in the review they should draw on a valid comparison group that does not participate in summer programmes covered by the evaluation, the provision under BAU might include some form of programme, potentially another summer programme, which may not be clear in the study. Some transition support programmes in particular might not offer an intervention that is substantially different from that available to those in the comparison group as part of BAU (Garcia et al., 2020), which will naturally affect the estimate of impact.

Table 7 summarises the findings of impact across the outcome measures considered by this review. This covers the findings of impact or effect based on allocation to the summer programme unless otherwise indicated (noted by an asterisk,*). Where meta‐analysis was possible and performed (exceptions were made to the rule requiring studies covering at least four different interventions to evaluate an outcome in order for it to be eligible for meta‐analysis for violence and offending outcomes at the request of the review's advisory group), the average effect size, 95% confidence interval, and overall finding regarding the presence and direction of an impact are reported. Below these we also report the *I* ^2^ statistic and the *p*‐value from the homogeneity test, as two measures of heterogeneity in effect sizes. Where heterogeneity between effect sizes is present, this affects the extent to which the mean effect size may be generalisable to other summer programmes of the same type. Note that when the number of studies is limited, the homogeneity test is known to be underpowered, therefore it may not indicate statistically significant heterogeneity in instances where it is present (Hedges & Pigott, 2001). As such, we use a 90% confidence level to examine whether heterogeneity may be present based on this test.

**Table 7:** Summary of impact/effect findings.

Outcome (effect size type)	Effect size (95% confidence interval) [headline impact finding]			Security of findings
	All summer programmes	Summer education programmes	Summer employment programmes	
*Education*				
Engagement /participation/enjoyment (SMD)	–	0.12 (0.03, 0.20) [Positive impact] *I* ^2^ = 48.76% θ = θ_i_: *p* = 0.10	–	Low: all high‐quality, consistent findings, but sensitivity analysis lowers rating and 4 of 5 studies find no impact
Skills /confidence /self‐efficacy (SMD)*	–	No impact	–	Low: only 3 studies and one finds positive impact
Secondary education attendance (SMD)*	–	0.26 (0.08, 0.44) [Positive impact] *I* ^2^ = N/A θ = θ_i_: *p* = N/A	0.02 (−0.03, 0.07) [No impact] *I* ^2^ = 69.98% θ = θ_i_: *p* = 0.03	Education – low: only 1 study and of moderate quality Employment – low: 3 of 7 studies find positive impact
Chronic absence^a^ (log OR)*	–	Negative impact	No impact	Education – low: only 1 study Employment – low: only 1 study
Passing tests (log OR)	–	0.41 (−0.13, 0.96) [No impact] *I* ^2^ = 95.05% θ = θ_i_: *p* = 0.00	0.02 (0.00, 0.04) [Positive impact] *I* ^2^ = 0.01% θ = θ_i_: *p* = 0.33	Education – low: wide variation, 2 studies find positive impact and one an outlier Employment – low: 2 interventions studied produce different findings
Reading test scores (SMD)	–	0.01 (−0.04, 0.05) [No impact] *I* ^2^ = 0.40% θ = θ_i_: *p* = 0.48	–	High: consistent finding of no impact, 4 of 5 studies are high‐quality
Writing test scores (SMD)*	–	Mixed (positive & no impact)	–	Low: inconsistent findings, only 4 studies, 2 low‐quality, 1 moderate‐quality
Other language test scores (SMD)	–	Mixed (positive & no impact)	–	Low: only 2 studies and differing findings
English test scores (SMD)	–	0.07 (0.00, 0.13) [Positive impact] *I* ^2^ = 27.17% θ = θ_i_: *p* = 0.33	−0.03 (−0.05, −0.01) [Negative impact] *I* ^2^ = 0.00% θ = θ_i_: *p* = 0.76	Education – moderate: 6 studies, 5 high quality, 1 low quality, consistently find positive effect but only one finds positive impact, sensitivity analysis reduces confidence Employment – low: 2 studies, 1 has wide CI, outcome measure used by the other may downward bias estimate
Mathematics test scores (SMD)	0.09 (−0.06, 0.25) [No impact] *I* ^2^ = 94.53% θ = θ_i_: *p* = 0.00	0.14 (−0.09. 0.36) [No impact] *I* ^2^ = 94.15% θ = θ_i_: *p* = 0.00	0.00 (−0.04, 0.05) [No impact] *I* ^2^ = 0.04% θ = θ_i_: *p* = 0.92	All – high: 7 studies, 5 of which find no impact, majority have relatively tight CI, 6 are high quality Education – moderate: 4 of 5 studies find no impact, 1 finds positive impact although may be feature of measure used Employment – moderate: 2 high quality studies, both precisely estimate no impact, outcome measure used by 1 may downward bias estimate
Overall test scores (SMD)*	–	–	−0.01 (−0.08, 0.05) [No impact] *I* ^2^ = 32.39% θ = θ_i_: *p* = 0.20	Employment – high: consistent findings, consistent measure and mostly high quality studies
Test scores, pooled (SMD)	–	0.14 (0.00, 0.27) [Positive impact] *I* ^2^ = 91.07% θ = θ_i_: *p* = 0.00	−0.01 (−0.04, 0.01) [No impact] *I* ^2^ = 0.06% θ = θ_i_: *p* = 0.73	Education – moderate: 7 of 8 studies high quality, all except one study find positive effect but only two find positive impact, one of which low quality, the other is an outlier Employment – high: 4 high quality studies, all find insignificant impact, outcome measure used by 1 may downward bias estimate
On‐time secondary education completion (log OR)*	–	No impact	Mixed (positive & no impact)	Education – low: only 1 low quality study Employment – low: only 2 studies with different findings, 1 low quality
Negative behavioural outcomes^b^ (log OR)*	–	−1.55 (−3.14, 0.03) [Negative impact^c^] *I* ^2^ = N/A θ = θ_i_: *p* = N/A	−0.07 (−0.33, 0.18) [No impact] *I* ^2^ = 88.17% θ = θ_i_: *p* = 0.00	Education – low: only one study of low quality Employment – moderate: largely consistent findings of no impact, although one high quality study finds positive impact
Apply to HE (log OR)	No impact	–	–	Moderate: consistent finding of no impact across all studies, but only 3 studies (2 education, 1 employment)
Progression to HE (log OR)	0.24 (−0.04, 0.52) [No impact] *I* ^2^ = 97.37% θ = θ_i_: *p* = 0.00	0.32 (−0.12, 0.76) [No impact] *I* ^2^ = 96.58% θ = θ_i_: *p* = 0.00	0.10 (−0.07, 0.26) [No impact] *I* ^2^ = 76.61% θ = θ_i_: *p* = 0.02	All – low: wide variation across studies Education – low: 3 studies find no impact, 2 find significant positive Employment – moderate: 2 estimate no impact quite precisely, 1 that estimates positive impact uses intermediate measure
Complete HE (log OR)*	–	0.38 (0.15, 0.62) [Positive impact] *I* ^2^ = 52.52% θ = θ_i_: *p* = 0.06	0.07 (−0.19, 0.33) [No impact] *I* ^2^ = 70.54% θ = θ_i_: *p* = 0.07	Education – high: 4 of 5 find positive impact, mostly quite imprecise although 4 high quality Employment – moderate: 2 high quality studies, both find no impact
STEM‐related higher education outcomes (log OR)*	–	Positive impact	–	Moderate: largely consistent findings of positive impact, but wide range of outcome measures and only 3 interventions studied
*Non‐education*				
Entry to employment (log OR)*				
Short‐term	–	–	−0.19 (−0.45, 0.08) [No impact] *I* ^2^ = 87.81% θ = θ_i_: *p* = 0.00	Low: variation in findings and measures used
Full period	–	–	−0.15 (−0.35, 0.05) [No impact] *I* ^2^ = 78.88% θ = θ_i_: *p* = 0.00	Low: variation in findings and measures used
Earnings (SMD)*	–	–	Mixed (no & negative impact)	Low: inconsistent findings
Job readiness (log OR & SMD)*	Mixed (positive & no impact)	–	–	Low: variation in findings within studies across measures and over time
Whether had a criminal justice outcome^d^ (log OR)*	–	–	−0.05 (−0.15, 0.05) [No impact] *I* ^2^ = 0.00% θ = θ_i_: *p* = 0.76	Low: mix between insignificant and significant, small number of studies means significance highly sensitive
Drug	–	–	0.16 (−0.57, 0.89) [No impact] *I* ^2^ = 65.97% θ = θ_i_: *p* = 0.09	Low: only 2 studies and 1 has mixed findings
Violent	–	–	0.03 (−0.02, 0.08) [No impact] *I* ^2^ = 0.00% θ = θ_i_: *p* = 0.22	Moderate: only 2 studies but consistent findings and both high quality
Property	–	–	0.09 (−0.17, 0.34) [No impact] *I* ^2^ = 45.01% θ = θ_i_: *p* = 0.18	Low: only 2 studies, variation in findings by measure within a high quality study
Number of criminal justice outcomes^e^ (SMD)*				
During	–	–	−0.01 (−0.03, 0.00) [No impact] *I* ^2^ = 2.17% θ = θ_i_: *p* = 0.31	Low: only 2 interventions studied with mixed findings
Post	–	–	−0.02 (−0.05, 0.02) [No impact] *I* ^2^ = 23.57% θ = θ_i_: *p* = 0.37	Low: small number of studies, one finds substantial negative
Drug (post)	–	–	−0.00 (−0.06, 0.06) *I* ^2^ = 55.19% θ = θ_i_: *p* = 0.14	Moderate: only 2 interventions studied but consistent findings and all 4 studies high quality
Violent (post)	–	–	−0.02 (−0.08. 0.03) *I* ^2^ = 44.48% θ = θ_i_: *p* = 0.18	Low: only two interventions studied, mixed findings across studies, all high quality
Property (post)	–	–	−0.02 (−0.10, 0.05) *I* ^2^ = 64.93% θ = θ_i_: *p* = 0.09	Low: only 2 interventions studied and wide mix of findings
Socio‐emotional skills and engagement (SMD)*	–	Positive impact	Mixed (positive & no impact)	Education – low: only 2 studies, different measures Employment – low: only 2 studies with different findings
Community engagement (log OR)	–	No impact	Positive impact	Education – low: only 1 study Employment – low: only 1 study
Health (log OR & SMD)*	–	–	Beneficial impact^f^	Low: consistent findings but only 2 studies measuring different outcomes

^a^ For this outcome, a negative effect size indicates an improvement.

^b^ For this outcome, a negative effect size indicates an improvement.

^c^ The impact estimate from the one study of a summer education programme evaluating this outcome is itself significant: the specifics of the process for constructing the effect size means that the constructed effect size is insignificant.

^d^ For this outcome, a negative effect size indicates an improvement.

^e^ For this outcome, a negative effect size indicates an improvement.

^f^ One of the studies measuring a health outcome measures an outcome for which a negative effect size indicates an improvement, whilst the other measures an outcome for which a positive effect size indicates an improvement.

*Source*: IES (2024).

The findings are reported for summer education programmes and summer employment programmes where relevant. We report the overall findings across both summer programme types where the overall findings are consistent between summer education and summer employment programmes. Where, instead of meta‐analysis the findings from studies were summarised narratively, the modal finding (whether a significant impact was detected and if so in which direction) is reported. The security of findings, as previously detailed, indicate our level of confidence (low, moderate or high) that the overall finding of impact likely reflects the true impact of the programme type on the outcome.

### 14.2 Detailed findings on the impacts and effects of summer programmes on education outcomes

#### 14.2.1 Engagement with, participation in and enjoyment of education

*Summary*:

▪ Summer education programmes appear to have a positive impact on this outcome, although we have low confidence in this finding as the majority of studies evaluating the outcome find no significant impact.

Five studies across five interventions report impacts of allocation to a summer programme on self‐reported measures of engagement with, participation in and enjoyment of education activities. These studies which all focused on summer education programmes, and the outcomes they covered, were:

▪ Higher Achievement:
  - Herrera et al. (2013) – proportion that participated in range of academic/enrichment activities; spring after first programme summer.
▪ Summer Active Reading Programme:
  - Maxwell et al. (2014) – enjoyment of reading index score/motivation to read index; 3 months after the summer programme.
▪ Tenmarks:
  - Lynch and Kim (2017) – family home mathematics engagement index score/mathematics intrinsic motivation index score/mathematics enjoyment index score; autumn after the summer programme.
▪ Building Educated Leaders for Life:
  - Somers et al. (2015) – engagement (behavioural and emotional) index score; autumn after the summer programme.
▪ STEM summer programmes:
  - Cohodes et al. (2022) – likes intellectual activities index score; spring after the summer programme.

Note that each of these studies source their self‐reported outcome measures from surveys. The extent of attrition (overall and differential rates) for the surveys used by Hererra (2013) and Lynch and Kim (2017) to source the outcome measures is highly concerning, and moderately concerning for that used by Maxwell et al. (2014), introducing a potential source of bias into the estimates produced. Attrition is not a significant concern for the survey‐based measures used by Somers et al. (2015) and Cohodes et al. (2022).

Herrera et al. (2013) also report this outcome two and four springs after first programme summer. However, as all other studies measure this outcome within 1 year after the programme summer these results are not included in the analysis. For each of the specific measures, the effect size is largest after 4 years, on average twice as large as after 1 year, whilst the effect sizes after 2 years are approximately equal to those after 1 year.

**Figure 4:**
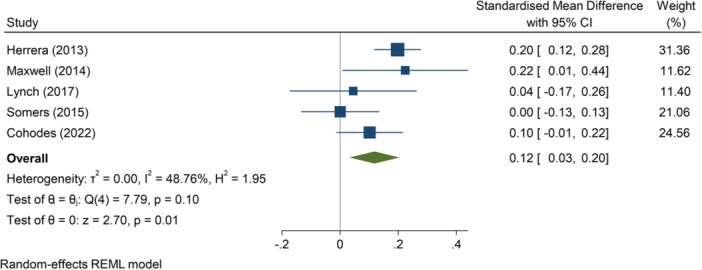
Impact of summer programme allocation on engagement with, participation in and enjoyment of education activities. *Source*: IES (2024).

Figure 4 displays the forest plot from the meta‐analysis of the impact of allocation to a summer programme on engagement with, participation in and enjoyment of education activities. As all the interventions included in this analysis are summer education programmes, there is no need to split by programme type.

The average effect for summer education programmes is positive and significant (SMD = 0.12, 95% confidence interval = 0.03, 0.20), which suggests that allocation to a summer education programme has a positive impact on engagement with, participation in and enjoyment of education activities. An SMD of 0.12 translates to, for instance, approximately 60% of those allocated to the summer programme moving from never reading for fun to doing so once or twice a month as measured by Maxwell et al. (2014). Note that the two impact estimates underlying the effect size from Maxwell et al. (2014) are themselves separately insignificant – by pooling the effect sizes produced together the aggregate effect size for the study becomes marginally significant.

The *p*‐value from the homogeneity test (*p* = 0.10) indicates that there is no evidence of statistically significant between‐study heterogeneity. There is no significant difference in effect size between the one study of an intervention that targets ethnic minorities (Cohodes et al., 2022) and the others that do not.

As sensitivity analysis, we exclude the estimate from Herrera et al. (2013), the highest weighted study, from the analysis. Doing so reduces the average effect size estimate to 0.08, and this average effect size is now marginally insignificant (95% confidence interval = −0.00, 0.16). The effect size estimate from Herrera et al. (2013) is derived by first combining a range of dichotomous outcomes (whether the individual participated in range of academic/enrichment activities or not) before then converting to a standardised mean difference to be comparable with the other outcome measures used – this process might affect the quality of the effect size constructed. Given that it is the resulting effect size form this that drives the significance level for the overall finding for the outcome, this reduces our confidence in the security of this finding.

Additionally, one study reported impacts of participation in a summer programme on self‐reported measures of engagement with, participation in and enjoyment of education activities:

▪ Elevate Math summer programme:
  - Snipes et al. (2015) – mathematics interest index score; autumn after the summer programme.

The extent of attrition (overall and differential rates) for the survey used by Snipes et al. (2015) to source the outcome is highly concerning, introducing a potential source of bias into the estimate produced.

Snipes et al. (2015) finds no significant impact of participation in the summer programme on engagement with, participation in and enjoyment of education activities.

#### 14.2.2 Education skills, confidence and self‐efficacy

*Summary*:

▪ Summer education programmes generally appear to have no impact on this outcome, although only three studies evaluate this outcome, one of which finds a positive impact.
  One study reports impacts of allocation to a summer education programme on self‐reported measures of educational skills, confidence and self‐efficacy:
▪ STEM summer programmes:
  - Cohodes et al. (2022) – attention span index score/study skills index score/confidence index score; spring after the summer programme.

Note that Cohodes et al. (2022) sources their self‐reported outcome measures from a survey, although the extent of attrition (overall and differential rates) is not a significant concern.

Cohodes et al. (2022) finds no significant impact of allocation to the summer education programme on educational skills, confidence and self‐efficacy. There is also a consistent lack of significant impact across each of the specific indexes that they measured.

Two studies across two interventions reported impacts of participation in a summer education programme on self‐reported measures of educational skills, confidence and self‐efficacy. These are:

▪ Department for Education Summer Schools Programme:
  - Martin et al. (2013b) – pupil confidence index score/school readiness index score; autumn after the summer programme.
▪ Elevate Math summer programme:
  - Snipes et al. (2015) – mathematics self‐efficacy index score; autumn after the summer programme.

The extent of attrition (overall and differential rates) for the survey‐based measure used by Snipes et al. (2015) is highly concerning, introducing a potential source of bias into the estimate produced. Insufficient information is provided by Martin et al. (2013b) to assess the extent to which attrition for their survey‐based measure may introduce bias into the estimates produced, which is also concerning.

Martin et al. (2013b) finds that participation in the summer education programme has a positive significant impact on educational skills, confidence and self‐efficacy compared with those that do not after participating in the programme, which equates to approximately 42% of those participating in the summer programme moving from agreeing to strongly agreeing that they understand most of the work at school. This translates to an effect size (SMD) of 0.07 (95% confidence interval = 0.05, 0.10).

Snipes et al. (2015) finds no significant impact of participation in the summer education programme on educational skills, confidence and self‐efficacy.

#### 14.2.3 Secondary education attendance

*Summary*:

▪ The one study of a summer education programme that evaluates this outcome finds a positive impact, although it is of moderate quality.
▪ Summer employment programmes appear to have no impact on this outcome, although three of the seven studies evaluating this outcome do find a positive impact.
▪ The one study of a summer education programme that examines chronic absence rates finds a significant beneficial impact, whilst the one study of a summer employment programme that also evaluates this finds no impact.

Six studies across three interventions report impacts of allocation to a summer programme either on secondary education (or high school in the US) attendance rates, or on the number of days attended or absent from which an attendance rate can be derived:

▪ New York City Summer Youth Employment Program:
  - Leos‐Urbel (2014) – attendance rate; year after the summer programme.
  - Valentine et al. (2017) – attendance rate; year after the summer programme.
▪ Boston Summer Youth Employment Program:
  - Modestino (2019b) – attendance rate; year after the summer programme.
▪ One Summer Chicago:
  - Heller (2014) – number of days attended; year after the summer programme.
  - Davis and Heller (2020) – number of days attended; year after the summer programme.
  - Heller (2022) – number of days absent; year after the summer programme.

Leos‐Urbel (2014) estimates a positive significant impact of allocation to the summer employment programme on attendance rate, equal to a 1.3 percentage point increase in attendance rate which translates to an effect size (SMD) of 0.01 (95% confidence interval = 0.00, 0.01). Valentine et al. (2017) found no significant impact of allocation to the summer employment programme on attendance rate.

Modestino (2019b) finds a positive significant impact of allocation to the summer employment programme on attendance rate, equal to a 0.8 percentage point increase in attendance rate which translates to an effect size (SMD) of 0.07 (95% confidence interval = −0.02, 0.16).

Heller (2014), Davis and Heller (2020) and Heller (2022) all estimate no significant impact of allocation to the summer employment programme on attendance rate.

Eight studies across five interventions report impacts of participation in a summer programme on secondary education attendance rates, or report impacts of allocation to a summer programme which could be transformed into impacts of participation using differences in participation rates between the treatment and control groups. In addition to the six studies previously detailed, these are:

▪ Robotics Summer Learning Program:
  - Mac Iver and Mac Iver (2019) – attendance rate; year after the summer programme.
▪ STEP‐UP:
  - Reich (2018) – attendance rate; year after the summer programme.

**Figure 5:**
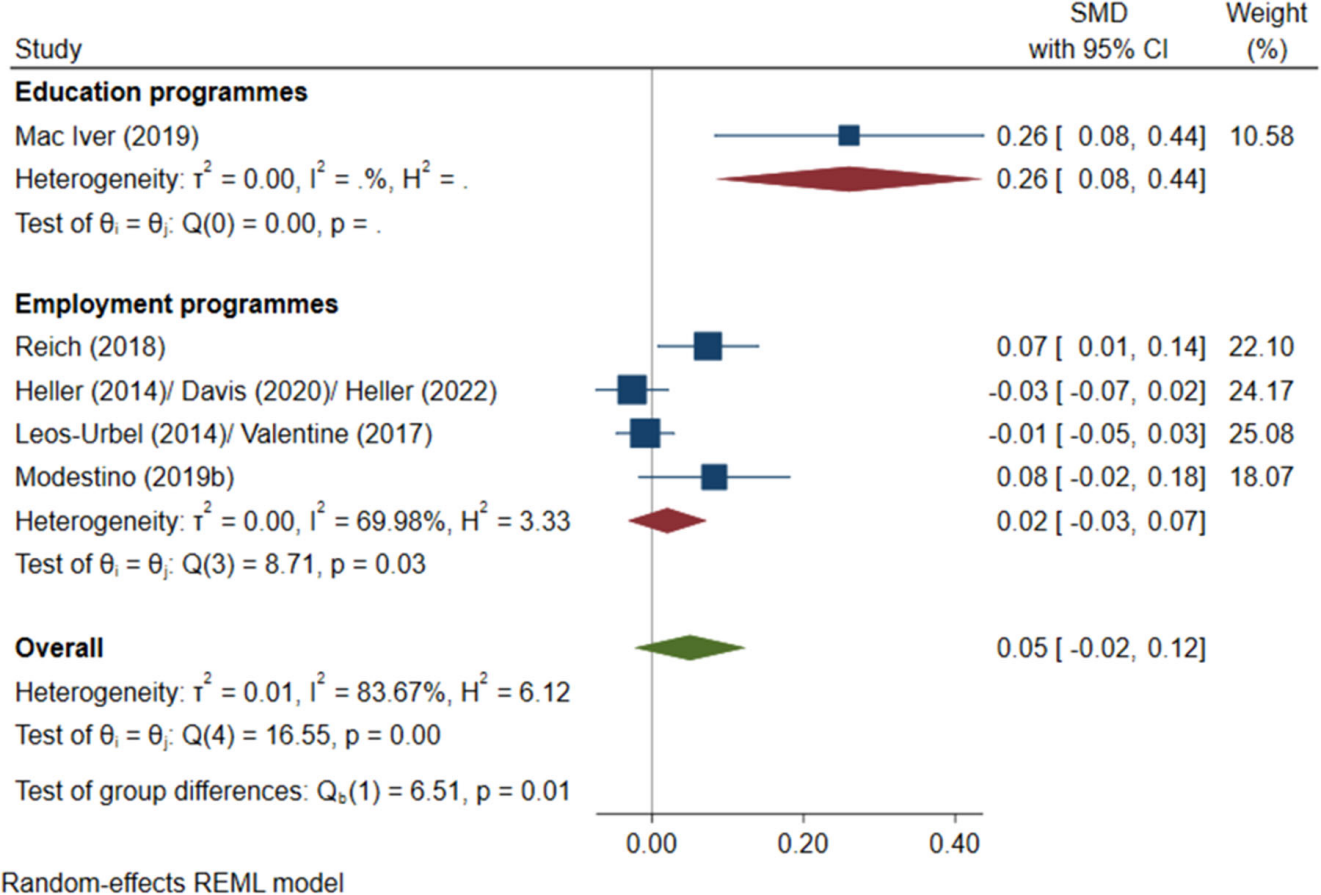
Impact of summer programme participation on secondary education attendance rates. *Source*: IES (2024).

Figure 5 displays the forest plot from the meta‐analysis of the impact of participation in a summer programme on secondary education attendance rates, split by summer education and employment programmes.

The test of group differences indicates that the difference in the average effect size between summer education and summer employment programmes is statistically significant, therefore we do not focus on the combined findings for both summer programme types. The one evaluation of a summer education programme by Mac Iver and Mac Iver (2019) finds a statistically significant impact of participation in the summer education programme on attendance rates (SMD = 0.26, 95% confidence interval = 0.08, 0.44). Summer employment programmes have a positive although insignificant average effect size (SMD = 0.02, 95% confidence interval = −0.03, 0.07), suggesting that participation in a summer employment programme does not have a significant impact on secondary education attendance rates.

Within summer employment programmes, the *p*‐value from the homogeneity test (*p* = 0.03) indicates that there is statistically significant between‐study heterogeneity, reducing the applicability of the average effect size to all summer employment programmes. As there are less than 10 interventions covered in this analysis, sub‐group analysis is used to examine heterogeneity. Instances of statistically significant differences on average effect size between sub‐groups are:

▪ between raising aspirations and workplace exposure programmes (i.e., all the other included interventions), and those programmes that target individuals with poor academic performance and those that do not, although Mac Iver and Mac Iver (2019) is the only study that evaluated a raising aspirations programme or a programme that targeted individuals with poor academic performance; and
▪ between the one programme that targeted areas containing individuals with experience of or at risk of involvement with the criminal justice system (studied by Heller (2014), Davis and Heller (2020) and Heller (2022)) and those programmes that did not target these areas (SMD = 0.08, 95% confidence interval = −0.01, 0.16).

None of the interventions included in this analysis target individuals from ethnic minorities.

As the average effect size of summer employment programmes is insignificant, as is the effect size from the highest weighted intervention evaluated by Leos‐Urbel (2014) and Valentine et al. (2017) which is weighted only slightly more highly than most of the other studies, there is no need to remove this estimate as a form of sensitivity analysis. Additionally, the estimates of impact of participation in the programmes from Leos‐Urbel (2014), Valentine et al. (2017) and Modestino (2019b) are based on transformations of reported estimated impacts of allocation to the programme – removing these from the analysis has a negligible impact on the findings.

Additionally, two studies across two interventions report impacts of allocation to or participation in a summer programme on whether an individual is chronically absent:

▪ Urban Alliance:
  - Theodos et al. (2017) – proportion chronically absent; year after the summer programme.
▪ Aim High:
  - Pyne et al. (2020) – proportion chronically absent, across 3 years after the summer programme.

Theodos et al. (2017) does not provide a definition for being chronically absent – the US Department of Education (2016) define it as missing at least 15 days of school in a year, but it is also commonly defined as missing more than 10% of the days in a school year (Centre for Research in Education & Social Policy, 2018). They do not find that allocation to the summer employment programme has a significant impact on the likelihood of being chronically absent.

Pyne et al. (2020) defines chronic absence as missing more than 10% of the days in a school year. They find that participation in the summer education programme has a significant negative (i.e., reducing the likelihood) impact on the likelihood that an individual is chronically absent, equal to a 1.4 percentage point reduction in the chronic absence rate. This translates to an effect size (log odds ratio) of −0.28 (95% confidence interval = −0.69, 0.14 – note that the specifics of the process for constructing the effect size means that it is insignificant despite the underlying impact estimate being significant) and an odds ratio of 0.76 (95% confidence interval = 0.50, 1.15). So, an individual who participated in the summer education programme is 0.76 times as likely to be chronically absent across the 3 years after the summer programme as someone who did not.

#### 14.2.4 Passing tests

*Summary*:

▪ Summer education programmes appear to have no impact on this outcome – two of the three studies evaluating the outcome find a positive impact, although one of these is an outlier which may be explained by the specifics of the measure they use.
▪ Summer employment programmes appear to have a positive impact on this outcome, although this is driven by one study that uses an elective test where the increased pass rate appears to be a result of increased test taking rather than increased aptitude.

Six studies across five interventions report impacts of allocation to a summer programme on the likelihood of passing tests:

▪ Texas developmental summer bridge programme:
  - Wathington et al. (2016) – probability of passing first college‐level mathematics/reading/writing course; within 2 years after the summer programme.
▪ STEM summer programmes:
  - Cohodes et al. (2022) – proportion able to answer calculus question; spring after the summer programme.
▪ Elevate Math summer programme:
  - Snipes et al. (2015) – proportion passing three or more areas of the mathematics Diagnostic Testing Project Algebra Readiness test; autumn after the summer programme.

▪ New York City Summer Youth Employment Program:
  - Leos‐Urbel (2014) – probability of passing English/mathematics Regents exam; within the year after the summer programme.
  - Schwartz et al. (2021) – probability of passing Regents exam; within the year after the summer programme.
▪ Boston Summer Youth Employment Program:
  - Modestino (2019b) – proportion proficient or better in Massachusetts Comprehensive Assessment System English language arts/mathematics test; within the year after the summer programme.

The extent of attrition (overall and differential rates) for the post‐intervention test used to source the outcome measure for Snipes et al. (2015) is highly concerning, potentially introducing a source of bias into the estimates. For Cohodes et al. (2022) which uses a survey‐based measure, the extent of attrition is not concerning.

**Figure 6:**
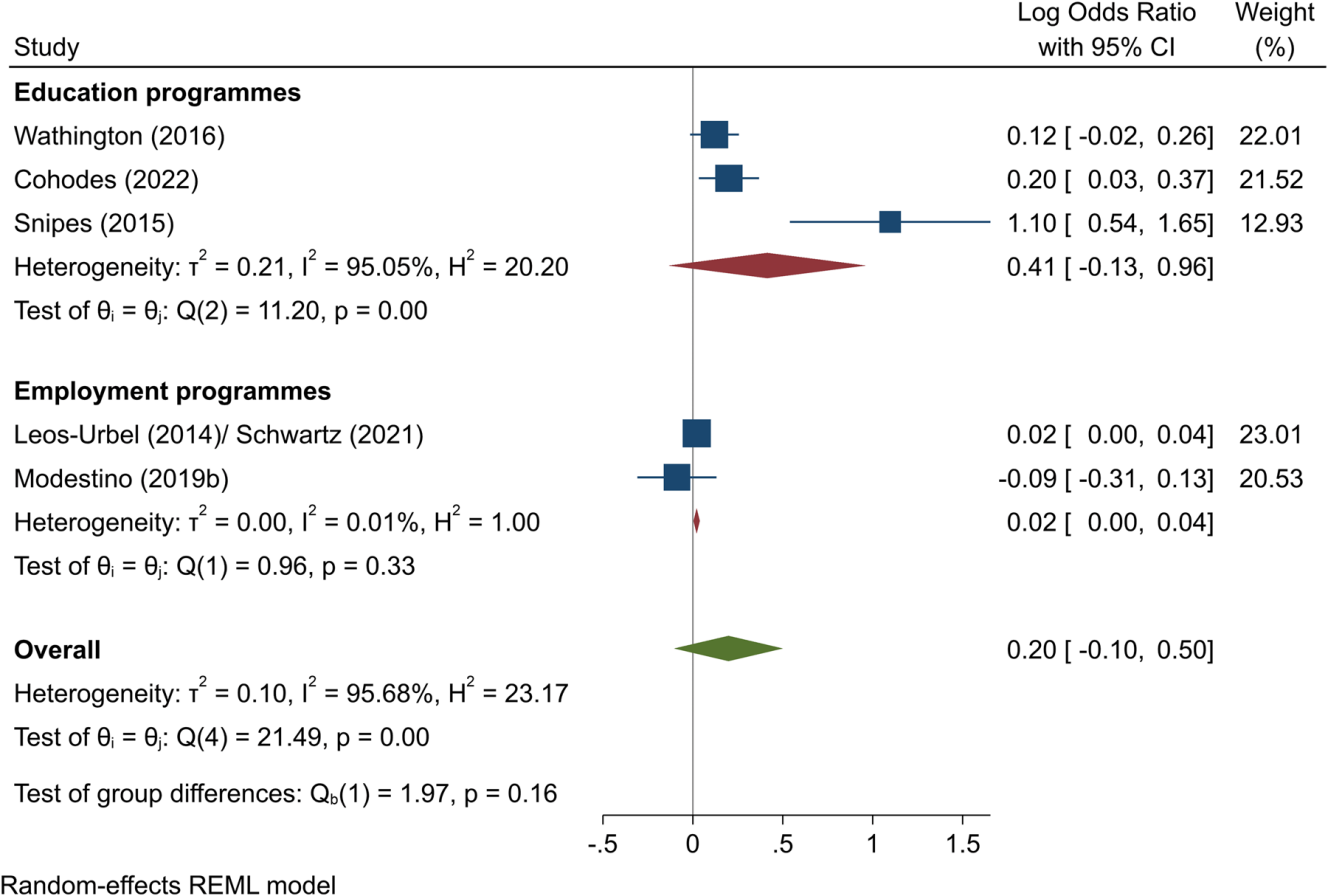
Impact of summer programme allocation on likelihood of passing tests. *Source*: IES (2024).

Figure 6 displays the forest plot from the meta‐analysis of the impact of allocation to a summer programme on the likelihood of passing tests, split by summer education and employment programmes.

Summer programmes and summer education programmes have positive although insignificant average effect sizes (log odds ratio (summer programmes) = 0.20, 95% confidence interval = −0.10, 0.50; log odds ratio (summer education programmes) = 0.41, 95% confidence interval = −0.13, 0.96), suggesting that allocation to a summer programme or summer education programme does not have a significant impact on the likelihood of an individual to pass tests. The average effect size for summer employment programmes is positive and statistically significant (log odds ratio = 0.02, 95% confidence interval = 0.00, 0.04). A log odds ratio of 0.02 translates to an odds ratio of 1.02, which means that individuals who were allocated to a summer employment programme are 1.02 times more likely to pass tests after the programme than those who were not. Moreover, using as the assumed comparator risk the average in the proportions of control individuals passing the test from Leos‐Urbel (2014) and Schwartz et al. (2021), this equates to a need to treat 206 young people to see this outcome for one of them as a result of allocation to a summer programme. The test of group differences indicates that there is not a significant difference on average effect size between education and summer employment programmes.

Within summer education programmes, the *p*‐value from the homogeneity test (*p* = 0.00) indicates that there is statistically significant between‐study heterogeneity, reducing the applicability of the average effect size to all summer education programmes. This is not the case for summer employment programmes (*p* = 0.33). As there are less than 10 interventions covered in this analysis, sub‐group analysis is used to examine heterogeneity. Instances of statistically significant differences on average effect size between sub‐groups are:

▪ between intervention types – the one catch‐up programme studied by Snipes et al. (2015) has a much larger effect size than the one raising aspirations intervention studied by Cohodes et al. (2022), the one transition support programme studied by Wathington et al. (2016), or the two workplace exposure interventions (average effect size = 0.02, 95% confidence interval = 0.00, 0.04).

There is no significant difference in effect size between the one programme that targets individuals from ethnic minorities, studied by Cohodes et al. (2022), and the other studies that do not.

The significance of the average effect size from summer employment programmes is driven by the combined result from Leos‐Urbel (2014) and Schwartz et al. (2021). It should be noted that the test that Leos‐Urbel (2014) and Schwartz et al. (2021) use to measure this outcome, the Regents Exam, is an elective, non‐universal examination that can be taken multiple times, therefore the positive impact they estimate on the likelihood of passing the test may be a result of both an increase in ability as well as an increased willingness to take the test (Schwartz et al., 2021) estimates positive effects on the likelihood of taking the Regents Exam). Removing the result from these studies naturally results in the average effect size of summer employment programmes mirroring the effect size from Modestino (2019b), that is, the only other summer employment programme.

Additionally, Schwartz et al. (2021) estimates the impact of participation in the summer employment programme on the likelihood of passing tests. The result is broadly in line with their estimate of the impact of allocation to the summer employment programme on this outcome, that is, a positive and significant impact, equal to a 0.9 percentage point increase in the likelihood of passing the test which translates to an effect size (log odds ratio) of 0.04 (95% confidence interval = 0.01, 0.06).

#### 14.2.5 Test scores

*Summary*:

▪ Summer education programmes appear to have a positive impact on any form of test score and English test scores specifically, although they appear to have no impact on reading, writing or mathematics scores.
▪ Summer employment programmes appear to have a negative impact on English test scores, although this is driven by the finding from one study that uses an elective test to measure the outcome where the negative impact is likely driven by a ‘compositional’ effect of the summer employment programme encouraging lower ability students to take the test. They also appear to have no impact on all forms of test scores or mathematics test scores specifically.
▪ There is an even split in studies evaluating both English and mathematics scores between those that find a greater effect or impact on one than the other – reasons put forward by studies finding greater effects/impacts on mathematics than English are differences in teaching quality, susceptibility to summer learning loss, student engagement with the curricula, or effectiveness of the curricula among students performing below grade level.

A large number of studies report estimates of impact of allocation to/participation in a summer programme on scores across a wide range of tests. To explore these outcomes we first look at the impact of summer programmes on test scores within specific areas of English language – reading, writing, spelling and grammar, listening, and speaking.

Five studies across five interventions report impacts of allocation to a summer programme on reading scores:

▪ Higher Achievement:
  - Herrera et al. (2013) – standardised Stanford Achievement Test 10 reading score; spring after first programme summer.
▪ Building Educated Leaders for Life:
  - Somers et al. (2015) – Group Reading Assessment and Diagnostic Examination score; autumn after the summer programme;
▪ Summer Active Reading Programme:
  - Maxwell et al. (2014) – New Group Reading Test score; 3 months after the summer programme.
▪ Discover Summer School:
  - Torgerson et al. (2014) – Progress in English reading score; 3 weeks after the summer programme.
▪ English Learner Summer School:
  - Johnson (2020) – standardised California English Language Development Test reading score; year after the summer programme.

The extent of attrition (overall and differential rates) to the post‐programme tests used by Herrera et al. (2013) and Torgerson et al. (2014) are highly concerning, and are moderately concerning for Maxwell et al. (2014), introducing a potential source of bias into the estimates produced. Attrition is not a significant concern for Somers et al. (2015).

The specific measure used by Herrera et al. (2013) is referenced in Garcia et al. (2020). Herrera et al. (2013) also report this outcome two and four springs after the first programme summer. However, as all other studies measure this outcome within 1 year after the programme summer these results are not included in the analysis (they also do not find significant impacts at these time points).

**Figure 7:**
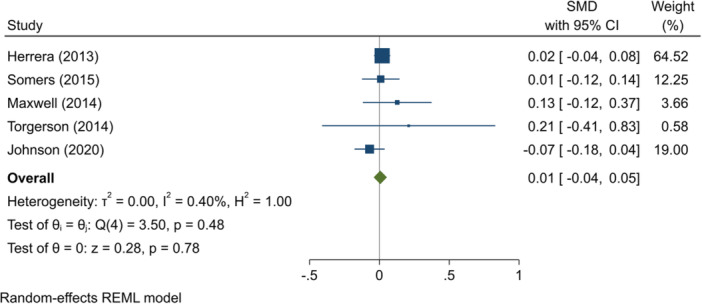
Impact of summer programme allocation on reading scores. *Source*: IES (2024).

Figure 7 displays the forest plot from the meta‐analysis of the impact of allocation to a summer programme on reading scores. As all the interventions included in this analysis are summer education programmes, the analysis is not split by programme type.

The average effect size for summer education programmes is positive although not significant (SMD = 0.01, 95% confidence interval = −0.04, 0.05), suggesting that allocation to a summer education programme does not have a significant impact on reading scores.

The *p*‐value from the homogeneity test (*p* = 0.48) indicates that there is no statistically significant evidence of between‐study heterogeneity. None of the interventions included in this analysis targeted individuals from ethnic minorities.

Removing the result from Herrera et al. (2013), by far the highest weighted study, means that the average effect size becomes negative (SMD = −0.01) although it is still insignificant (95% confidence interval = −0.10, 0.08).

Five studies across five interventions report impacts of participation in a summer programme on reading scores or report impacts of allocation to a summer programme which could be transformed into impacts of participation. The estimates of impact of allocation to the summer programme on reading scores noted above for Herrera et al. (2013) and Johnson (2020) cannot be translated to estimates of impact of participation in the summer programme, as they report standardised test scores with insufficient information to transform them. It is possible to convert the results from Somers et al. (2015), Maxwell et al. (2014) and Torgerson et al. (2014) previously detailed, and two further studies can be added into this analysis:

▪ Future Foundations summer school programme:
  - Siddiqui et al. (2014) – Key Stage 2 to post‐test reading gain score; autumn after the summer programme.
▪ Summer Learning Journey:
  - Williamson et al. (2020) – PAT reading comprehension gain score; autumn after the summer programme.

The extent of attrition (overall and differential rates) to the post‐programme test administered as part of the evaluation in Siddiqui et al. (2014) is highly concerning, introducing a potential source of bias into the estimate produced.

Siddiqui et al. (2014) and Williamson et al. (2020) both use gain scores (i.e., the change in score) as their outcome measures. By comparing differences in these between the treatment and control/comparison group, measures of the impact of the summer programme on academic performance at the point in time of the post‐test are found, and therefore they can be considered alongside purely cross‐sectional measures.

**Figure 8:**
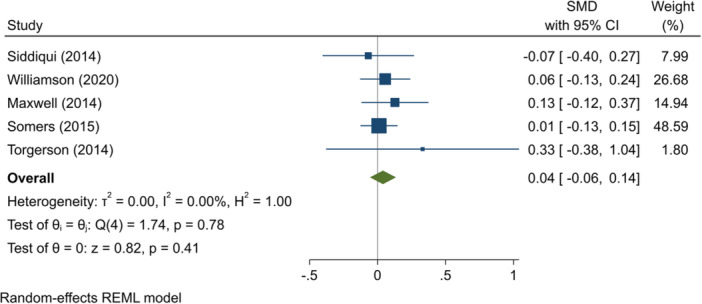
Impact of summer programme participation on reading scores. *Source*: IES (2024).

Figure 8 displays the forest plot from the meta‐analysis of the impact of participation in a summer programme on reading scores. As all the interventions included in this analysis are summer education programmes, the analysis is not split by programme type.

The average effect size for summer education programmes is positive although insignificant (SMD = 0.04, 95% confidence interval = −0.06, 0.14), suggesting that participation in a summer education programme does not have a significant impact on reading scores.

The *p*‐value from the homogeneity test (*p* = 0.78) indicates that there is no statistically significant evidence of between‐study heterogeneity. None of the interventions included in this analysis targeted individuals from ethnic minorities.

Removing the result from Somers et al. (2015), by far the highest weighted study, has a negligible impact on the findings. Removing both the results from Somers et al. (2015) and Torgerson et al. (2014), that is, the two studies that report impacts of allocation that require transformation, also has a negligible impact on the findings.

The third investigation is into the effect of summer programmes on writing scores. Two studies across two interventions report the impacts of allocation to an educational summer programme on writing scores. These are:

▪ Discover Summer School:
  - Torgerson et al. (2014) – Progress in English writing score; 3 weeks after the summer programme.
▪ English Learner Summer School:
  - Johnson (2020) – standardised California English Language Development Test writing score; 1 year after the summer programme.

The extent of attrition (overall and differential rates) to the post‐programme test administered as part of the evaluation in Torgerson et al. (2014) is highly concerning, introducing a potential source of bias into the estimate produced.

Torgerson et al. (2014) finds no significant impact of allocation to the summer education programme on writing scores.

Johnson (2020) finds a positive significant impact of allocation to the summer education programme on writing scores, equating to an effect size (*z*‐score) of 0.11 (95% confidence interval = 0.02, 0.20). It is worth noting this in comparison with the negative although insignificant effect of allocation to the summer education programme they estimated for reading scores. Johnson (2020) suggests as an explanation for this that reading skills may tend to develop more gradually compared with writing skills, and therefore the short duration of summer programmes may not allow sufficient time for as considerable an improvement in reading ability compared with writing.

Two studies across two interventions report impacts of participation in a summer education programme on writing scores:

▪ Future Foundations summer school programme:
  - Siddiqui et al. (2014) – Key Stage 2 to post‐test writing gain score, autumn after the summer programme.
▪ Summer Learning Journey:
  - Williamson et al. (2020) – e‐asTTle writing gain score, autumn after the summer programme.

The extent of attrition (overall and differential rates) to the post‐programme test administered as part of the evaluation in Siddiqui et al. (2014) is highly concerning, introducing a potential source of bias into the estimates produced.

The estimate of impact of allocation to the summer programme on writing scores noted above for Johnson (2020) cannot be translated to estimates of impact of participation in the summer programme, as they report standardised test scores with insufficient information to transform them. Therefore, meta‐analysis is not performed specifically for this outcome as only three studies across three interventions produce results that could be synthesised together, and so we discuss the results from Siddiqui et al. (2014) and Williamson et al. (2020) narratively.

Siddiqui et al. (2014) finds no significant impact of participation in the summer education programme on writing scores.

Williamson et al. (2020) estimates a positive significant impact of participation in the summer education programme on writing scores, equating to an effect size (SMD) of 0.22 (95% confidence interval = 0.05, 0.39). This study has a low‐quality study design, and the intervention studied is the only one included in this review occurring outside the UK and US (in New Zealand).

Two studies report impacts of allocation to a summer education programme on other types of (English) language test scores:

▪ Discover Summer School:
  - Torgerson et al. (2014) – Progress in English spelling and grammar score; 3 weeks after the summer programme.
    English Learner Summer School:
  - Johnson (2020) – standardised California English Language Development Test listening/speaking score; 1 year after the summer programme.

The extent of attrition (overall and differential rates) to the post‐programme test administered as part of the evaluation in Torgerson et al. (2014) is highly concerning, introducing a potential source of bias into the estimate produced.

Torgerson et al. (2014) finds no significant impact of allocation to the summer education programme on spelling and grammar scores.

Johnson (2020) finds a positive significant impact of allocation to the summer education programme on listening scores and on speaking scores, equating to an effect size (*z*‐score) of 0.18 (95% confidence interval = 0.04, 0.31) for listening and of 0.16 (95% confidence interval = 0.06, 0.26) for speaking.

Eight studies across eight interventions report impacts of allocation to a summer programme (either education or employment) on English scores, either for the subject as a whole or for specific areas which can be treated (either on their own or in combination across areas) as the impact on English scores. These are:

▪ Future Foundations summer school programme:
  - Gorard et al. (2014) – Key Stage 2 to GL Assessment Progress in English gain score; autumn after the summer programme.
▪ Higher Achievement:
  - Herrera et al. (2013) – standardised Stanford Achievement Test 10 reading score; spring after first programme summer.
▪ Summer Active Reading Programme:
  - Maxwell et al. (2014) – New Group Reading Test score; 3 months after the summer programme.
▪ Discover Summer School:
  - Torgerson et al. (2014) – Progress in English reading/writing/spelling and grammar score; 3 weeks after the summer programme.
▪ Building Educated Leaders for Life:
  - Somers et al. (2015) – Group Reading Assessment and Diagnostic Examination score; autumn after the summer programme.
▪ English Learner Summer School:
  - Johnson (2020) – standardised California English Language Development Test overall score; year after the summer programme.
▪ New York City Summer Youth Employment Program:
  - Leos‐Urbel (2014) – standardised English Regents exam score; year after the programme.
▪ Boston Summer Youth Employment Program:
  - Modestino (2019b) – English Grade Point Average/standardised Massachusetts Comprehensive Assessment System English language arts score; year after the programme.

The specific measure used by Herrera et al. (2013) is referenced in Garcia et al. (2020).

**Figure 9:**
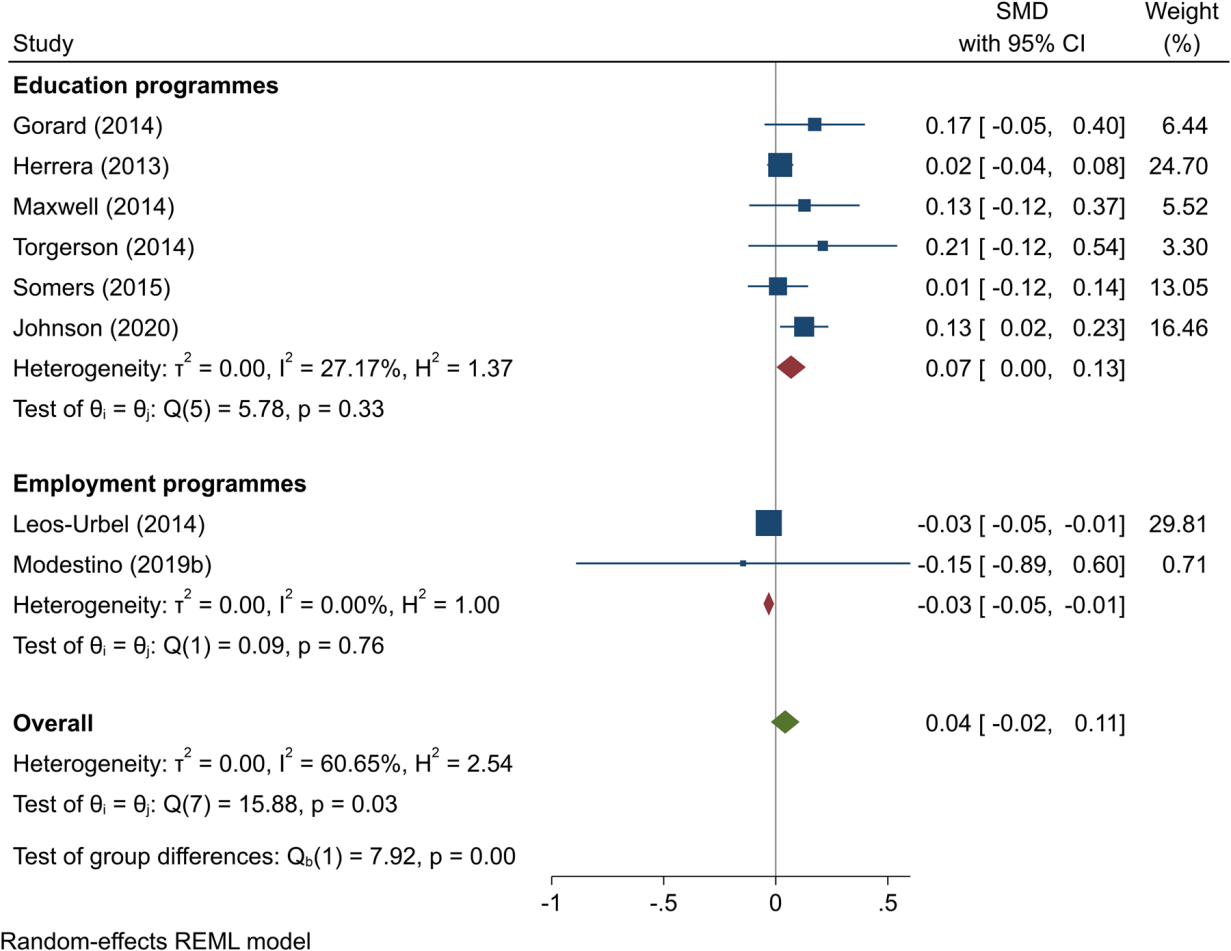
Impact of summer programme allocation on English scores. *Source*: IES (2024).

Figure 9 displays the forest plot from the meta‐analysis of the impact of allocation to a summer programme on English scores, split by summer education and employment programmes.

The test of group differences indicates that the difference in the average effect size between summer education and summer employment programmes is statistically significant, therefore we do not focus on the combined findings for both summer programme types. The average effect size for summer education programmes is positive and significant (SMD = 0.07, 95% confidence interval = 0.00, 0.13, *p*‐value = 0.04), suggesting that individuals who are allocated to a summer education programme have significantly better English scores than those that are not post‐programme. An SMD of 0.07 translates to, for instance, an increase in the English Grade Point Average (as measured by Modestino, 2019b) of 0.08 for those allocated to a summer education programme. The average effect size for summer employment programmes on English scores is negative and statistically significant (SMD = −0.03, 95% confidence interval = −0.05, −0.01), suggesting that individuals who are allocated to a summer employment programme have significantly worse English scores than those that are not post‐programme.

Within summer education and summer employment programmes, the *p*‐values from the homogeneity tests (*p* = 0.33 and *p* = 0.76 respectively) indicate that there is no statistically significant evidence of between‐study heterogeneity within the two groups of summer programme, although across all interventions there is statistically significant heterogeneity (*p* = 0.03). As there are less than 10 interventions covered in this analysis, sub‐group analysis is used to examine heterogeneity. Instances of statistically significant differences on average effect size between sub‐groups are:

▪ between intervention types – the one transition support programme, studied by Maxwell et al. (2014), has the largest effect size, followed by catch‐up programmes (SMD = 0.10, 95% confidence interval = 0.02, 0.18) whilst the one raising aspirations programme, studied by Herrera et al. (2013), and workplace exposure interventions (SMD = −0.03, 95% confidence interval = −0.06, −0.01) have much lower effect sizes;
▪ between programmes that target socioeconomically disadvantaged areas (SMD = −0.01, 95% confidence interval = −0.06, 0.04) and those that do not (SMD = 0.10, 95% confidence interval = 0.02, 0.18);
▪ between those programmes that target young people with English as a second language (SMD = 0.14, 95% confidence interval = 0.04, 0.23), studied by Gorard et al. (2014) and Johnson (2020), and those that do not (SMD = −0.00, 95% confidence interval = −0.05, 0.04); and
▪ between Johnson (2020) that is the only study with a low‐quality study design and the only one that studies a programme that targets individuals with specific needs, and all the other studies that have high‐quality study designs and study programmes that do not target individuals with specific needs (SMD = 0.01, 95% confidence interval = −0.04, 0.06). Given this is based on a sub‐group of one study, the external validity of this finding is quite limited.

None of the interventions included in this analysis targeted individuals from ethnic minorities.

The finding is largely driven by the result from Leos‐Urbel (2014) who finds that allocation to the summer employment programme has a statistically significant negative impact on English Regents exam scores. It is suggested that this finding is likely a result of the summer employment programme encouraging lower performing students to take this elective, non‐universal examination meaning that the average score of treatment individuals that take the test is lowered due to this ‘compositional’ effect, rather than allocation to a summer programme resulting in an actual decrease in English attainment. Removing the results from Leos‐Urbel (2014) and Herrera et al. (2013), as these are by far the highest weighted studies, increases the average effect size (SMD) of summer employment programmes to 0.10 which now becomes significant (95% confidence interval = 0.03, 0.17) and increases the average effect size of summer education programmes to 0.10 (95% confidence interval = 0.03, 0.18) respectively.

The next analysis focuses on the effects of participation in summer programmes on English scores. Nine studies across eight interventions report impacts of participation in a summer programme on English scores, or report impacts of allocation to a summer programme which could be transformed into impacts of participation. The estimates of impact of allocation to the summer programme previously outlined from Leos‐Urbel (2014), Herrera et al. (2013), Johnson (2020), and one of the measures evaluated by Modestino (2019b) cannot be translated to estimates of impact of participation in the summer programme, as they report standardised test scores with insufficient information to transform them. Hence, in addition to those others previously outlined, studies that can be included in this analysis are:

▪ Future Foundations summer school programme:
  - Siddiqui et al. (2014) – Key Stage 2 reading/writing to post‐test gain score; autumn after the summer programme.
▪ Summer Learning Journey:
  - Williamson et al. (2020) – PAT reading comprehension/e‐asTTle writing gain score; autumn after the summer programme.
▪ Summer Success Academy:
  - Mariano and Martorell (2013) – standardised English assessment score; spring after the summer programme.
▪ Aim High:
  - Pyne et al. (2020) – California Assessment of Student Performance and Progress English language arts score; within 2 years after the summer programme.

**Figure 10:**
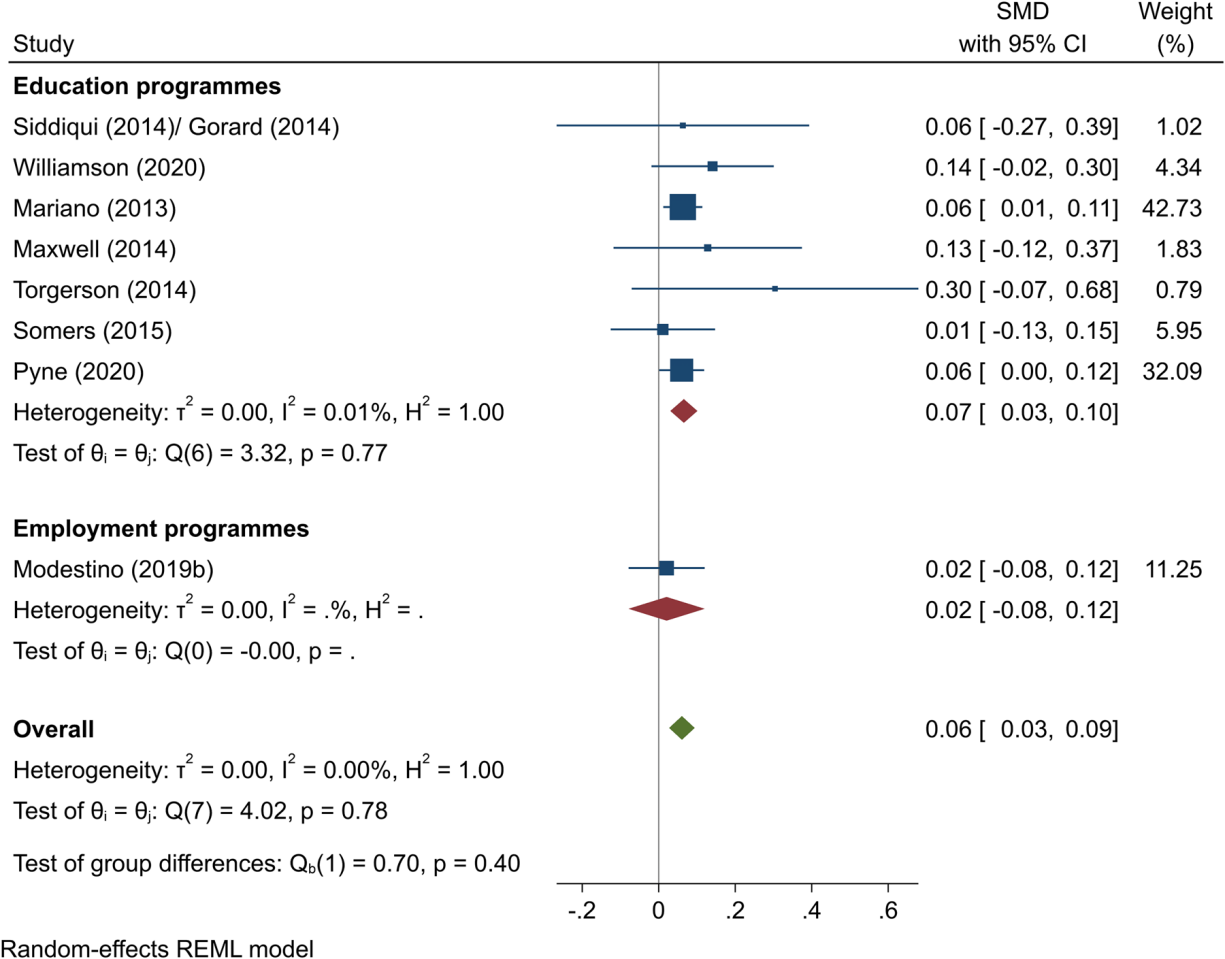
Impact of summer programme participation on English scores. *Source*: IES (2024).

Figure 10 displays the forest plot from the meta‐analysis of the impact of participation in a summer programme on English scores, split by summer education and employment programmes.

Despite the test of group differences not finding evidence of statistically significant differences on average effect size between summer education and summer employment programmes, the overall findings for the two programme types do differ. The average effect size for summer education programmes is positive and significant (SMD = 0.07, 95% confidence interval = 0.03, 0.10), suggesting that individuals who participate in a summer education programme have significantly better English scores than those who do not post‐programme. An SMD of 0.07 translates to, for instance, an increase in the English Grade Point Average (as measured by Modestino (2019b)) of 0.07 for those participating in a summer education programme. The one evaluation of a summer employment programme by Modestino (2019b) finds no statistically significant impact of participation in the summer employment programme on English test scores.

The *p*‐value from the homogeneity test (*p* = 0.77) suggests that there is no statistically significant heterogeneity. None of the interventions evaluating this outcome target individuals from ethnic minorities.

The average effect size for summer education programmes is largely driven by the results from Mariano and Martorell (2013) and Pyne et al. (2020). Removing the results from these studies reduces this to 0.09 which is marginally insignificant (95% confidence interval = −0.00, 0.18), as well as the average effect size for all summer programmes to 0.06 which is also now insignificant (95% confidence interval = −0.01, 0.12). Removing the results from Gorard et al. (2014), Torgerson et al. (2014), Somers et al. (2015) and Modestino (2019b), so from those studies that report impacts of allocation to a summer programme that are transformed into impacts of participation, has a negligible effect on the findings.

The next outcome in focus is mathematics scores. Seven studies across seven interventions report impacts of allocation to a summer education programme on mathematics scores. These are:

- Future Foundations summer school programme:
- Gorard et al. (2014) – Key Stage 2 to GL Assessment Progress in Mathematics gain score; autumn after the summer programme.
- Higher Achievement:
- Herrera et al. (2013) – standardised Stanford Achievement Test 10 mathematics (problem‐solving) score; spring after first programme summer.
- Building Educated Leaders for Life:
- Somers et al. (2015) – Group Mathematics Assessment and Diagnostic Examination score; autumn after the summer programme.
- Elevate Math summer programme:
- Snipes et al. (2015) – Mathematics Diagnostic Testing Project Algebra Readiness test score; autumn after the summer programme.
- Tenmarks:
- Lynch and Kim (2017) – standardised National and District curriculum‐based mathematics assessment score; autumn after the summer programme.
- New York City Summer Youth Employment Program:
- Leos‐Urbel (2014) – standardised mathematics Regents exam score; across the year after the programme.
- Boston Summer Youth Employment Program:
- Modestino (2019b) – mathematics Grade Point Average/standardised Massachusetts Comprehensive Assessment System mathematics score; the year after the programme.

The extent of attrition (overall and differential rates) to the post‐programme test administered as part of the evaluation in Herrera et al. (2013) and Snipes et al. (2015) are highly concerning, and moderately concerning for Somers et al. (2015), introducing a potential source of bias into the estimates produced. The extent of attrition to the post‐programme test administered as part of the evaluation in Gorard et al. (2014) is not concerning.

The specific measure used by Herrera et al. (2013) is referenced in Garcia et al. (2020). Herrera et al. (2013) also reports this outcome two and four springs after the first programme summer, however as all other studies measure this outcome within 1 year after the programme summer these results are not included in the analysis. They estimate impacts two and four springs after the first programme summer that translate to effect sizes (SMD) of 0.10 (95% confidence interval = 0.00, 0.20) and 0.11 (95% confidence interval = 0.00, 0.22) respectively.

**Figure 11:**
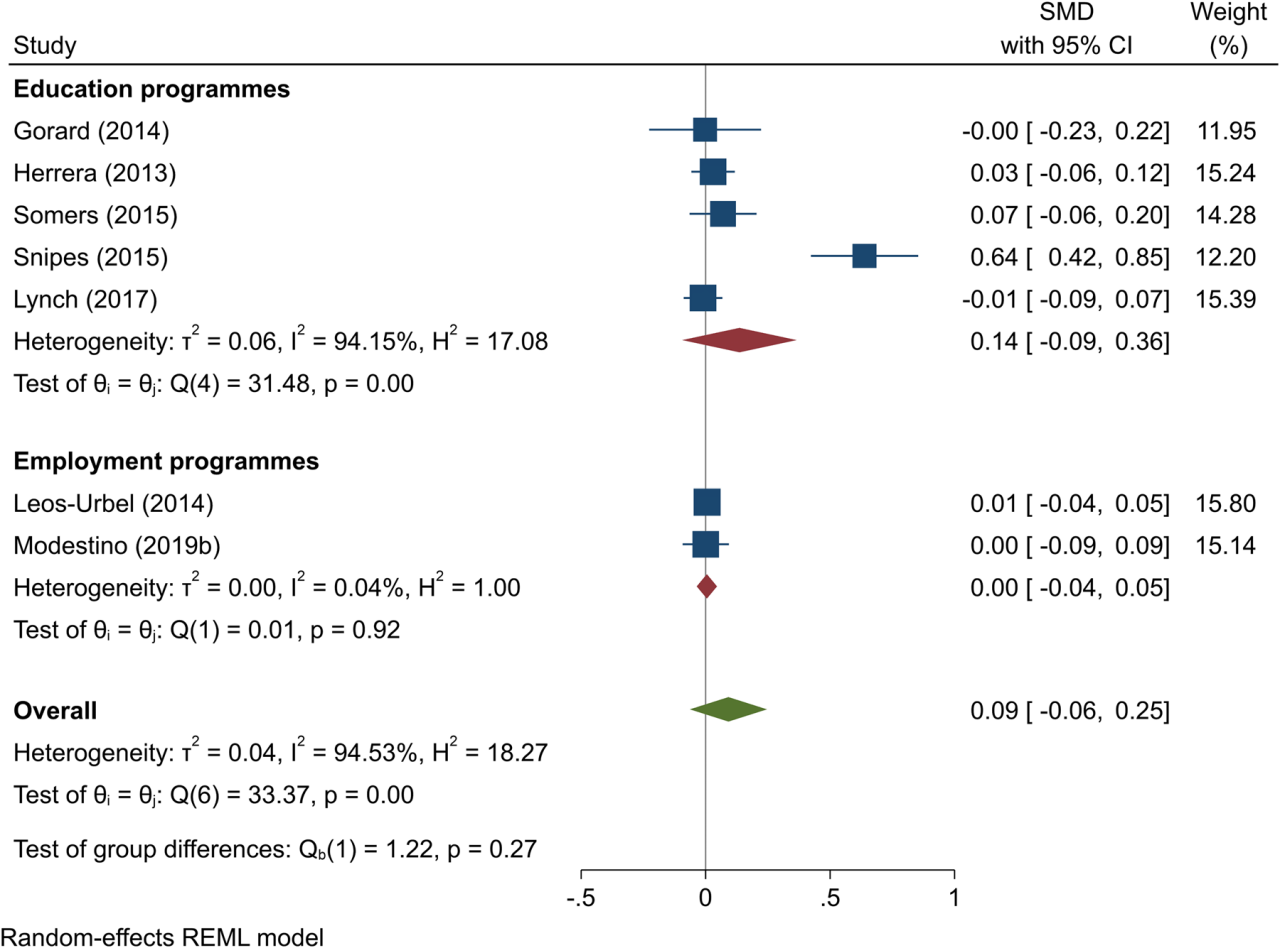
Impact of summer programme allocation on mathematics scores. *Source*: IES (2024).

Figure 11 displays the forest plot from the meta‐analysis of the impact of allocation to a summer programme on mathematics scores, split by summer education and employment programmes.

Both summer education and summer employment programmes have positive (and larger than their equivalent for English scores) although insignificant average effect sizes (SMD (summer education programmes) = 0.14, 95% confidence interval = −0.09, 0.36; SMD (summer employment programmes) = 0.00, 95% confidence interval = −0.04, 0.05). This suggests that allocation to a summer programme of either type has no impact on mathematics scores. The test of group differences indicates that there is no statistically significant difference on average effect size between summer education and employment programmes.

Within the summer education programmes sub‐group, the *p*‐value from the homogeneity test (*p* = 0.00) indicates that there is statistically significant evidence of between‐study heterogeneity, reducing the applicability of the average effect size to all summer education programmes. This is not the case for summer employment programmes (*p* = 0.92). There are no statistically significant differences on average effect size between any sub‐groups of studies (by region of the intervention, intervention type, ‘in whole’ vs. ‘in part’ summer programmes, forms of disadvantage targeted, or study quality). None of the interventions evaluating this outcome targeted individuals from ethnic minorities.

Removing the result from Snipes et al. (2015), which is somewhat an outlier, reduces the average effect size of summer education programmes which remains insignificant (SMD = 0.02, 95% interval = −0.04, 0.07).

Turning to the effect of participation in summer programmes, seven studies across six interventions report the impact of this on mathematics scores, or impacts of allocation to a summer programme that could be transformed into impacts of participation. The estimates of impact of allocation to the summer programme previously outlined from Leos‐Urbel (2014), Herrera et al. (2013), Lynch and Kim (2017) and one of measures evaluated by Modestino (2019b) cannot be translated to estimates of impact of participation in the summer programme, as they report standardised test scores with insufficient information to transform them. In addition to those others previously outlined, studies that can be included are:

▪ Future Foundations summer school programme:
  - Siddiqui et al. (2014) – Key Stage 2 mathematics to post‐test gain score; autumn after the summer programme.
▪ Summer Success Academy:
  - Mariano and Martorell (2013) – standardised mathematics assessment score; spring the year after the summer programme.
▪ Aim High:
  - Pyne et al. (2020) – California Assessment of Student Performance and Progress mathematics score; the 2 years after the summer programme.

The extent of attrition (overall and differential rates) to the post‐programme test administered as part of the evaluation in Siddiqui et al. (2014) is highly concerning, introducing a potential source of bias into the estimate produced.

**Figure 12:**
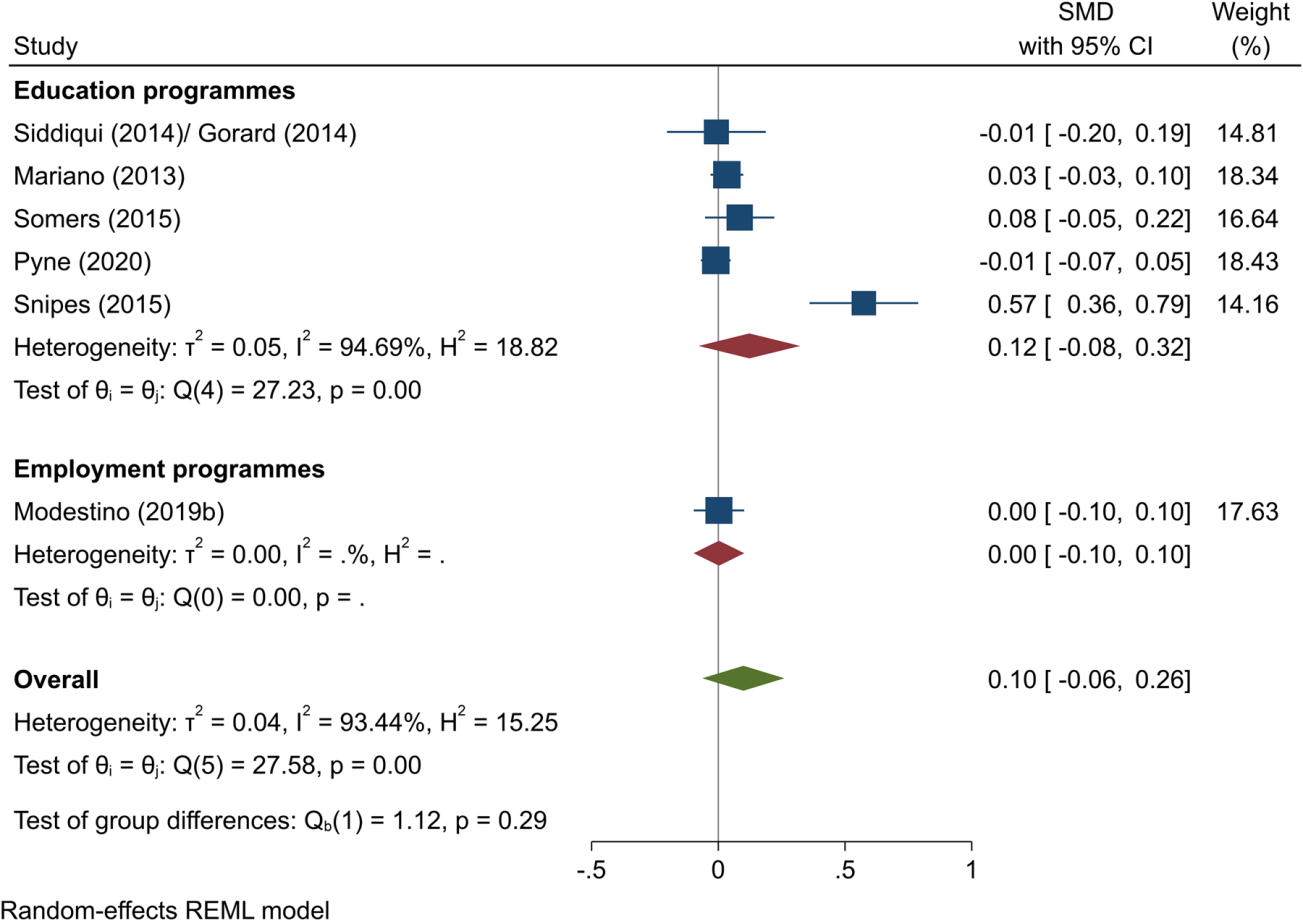
Impact of summer programme participation on mathematics scores. *Source*: IES (2024).

Figure 12 displays the forest plot from the meta‐analysis of the impact of participation in a summer programme on mathematics scores, split by summer education and employment programmes.

Again, both summer education and summer employment programmes (noting that there is only one evaluation of the latter by Modestino (2019b)) have positive (and larger than their equivalent for English scores) although insignificant average effect sizes (SMD (summer education programmes) = 0.12, 95% confidence interval = −0.08, 0.32; SMD (Modestino, 2019b) = 0.00, 95% confidence interval = −0.10, 0.10). This suggests that participation in a summer programme of either type has no impact on mathematics scores. The test of group differences indicates that the difference on average effect size between summer education and employment programmes is not statistically significant.

Within summer education programmes, the *p*‐value from the homogeneity test (*p* = 0.00) indicates that there is statistically significant evidence of between‐study heterogeneity, reducing the applicability of the average effect size to all summer education programmes. The only statistically significant difference on average effect between sub‐groups is between ‘in part’ (effect size = 0.57, 95% confidence interval = 0.36, 0.79) and ‘in whole’ summer programmes (effect size = 0.01, 95% confidence interval = −0.02, 0.05). However, this is because Snipes et al. (2015) finds a much larger effect size than any of the other studies included in this analysis and is the only ‘in part’ summer programme. None of the interventions included in this analysis targeted individuals from ethnic minorities.

Removing the result from Snipes et al. (2015) from the analysis reduces the average effect size of summer education which remains insignificant (SMD = 0.02, 95% confidence interval = −0.03, 0.06). Additionally, removing the results from Gorard et al. (2014), Somers et al. (2015) and Modestino (2019b) along with Snipes et al. (2015), that is, all studies that report impacts of allocation to a summer programme that are transformed into impacts of participation, has a negligible effect on the average effect size compared with just removing the result from Snipes et al. (2015).

Comparing studies that evaluate impacts on both English and mathematics scores, there is an even split between those that find greater effects for English than mathematics (Gorard et al., 2014; Mariano & Martorell, 2013; Modestino, 2019b; Siddiqui et al., 2014) and those that find greater effects for mathematics than English (Herrera et al., 2013; Leos‐Urbel, 2014; Somers et al., 2015), although it should be noted that the majority of these studies find no significant impact on either outcome. Where greater effects or impacts are found on mathematics scores, reasons suggested by the studies are that this could be due to a difference in the quality of the teaching observed, that mathematics is less susceptible than literacy to summer learning loss (Gorard et al., 2014), that students may be more engaged in the mathematics curriculum, or that English curricula may not be as effective with students below the expected level compared with the mathematics curricula (Somers et al., 2015). In studies that find a greater effect or impact on English, there is not sufficient evidence provided to understand why this may be the case.

Next, we consider the five studies across three interventions that report impacts of allocation to a summer employment programme on overall academic scores. These are:

▪ Boston Summer Youth Employment Program:
  - Modestino (2019b) – overall Grade Point Average; year after the summer programme.
▪ Urban Alliance:
  - Theodos et al. (2017) – cumulative Grade Point Average; year after the summer programme.
▪ One Summer Chicago:
  - Heller (2014) – overall Grade Point Average; year after the summer programme.
  - Davis and Heller (2020) – overall Grade Point Average; year after the summer programme.
  - Heller (2022) – overall Grade Point Average; year after the summer programme.

None of these studies find a significant impact of allocation to the summer programme on overall academic scores.

Turning to the effect of participation on this outcome measure, six studies across four interventions report impacts of participation in a summer programme on overall academic scores, or report impacts of allocation to a summer programme which could be transformed into impacts of participation. In addition to those previously outlined, the other is:

▪ STEP‐UP:
  - Reich (2018) – overall Grade Point Average; year after the summer programme.

**Figure 13:**
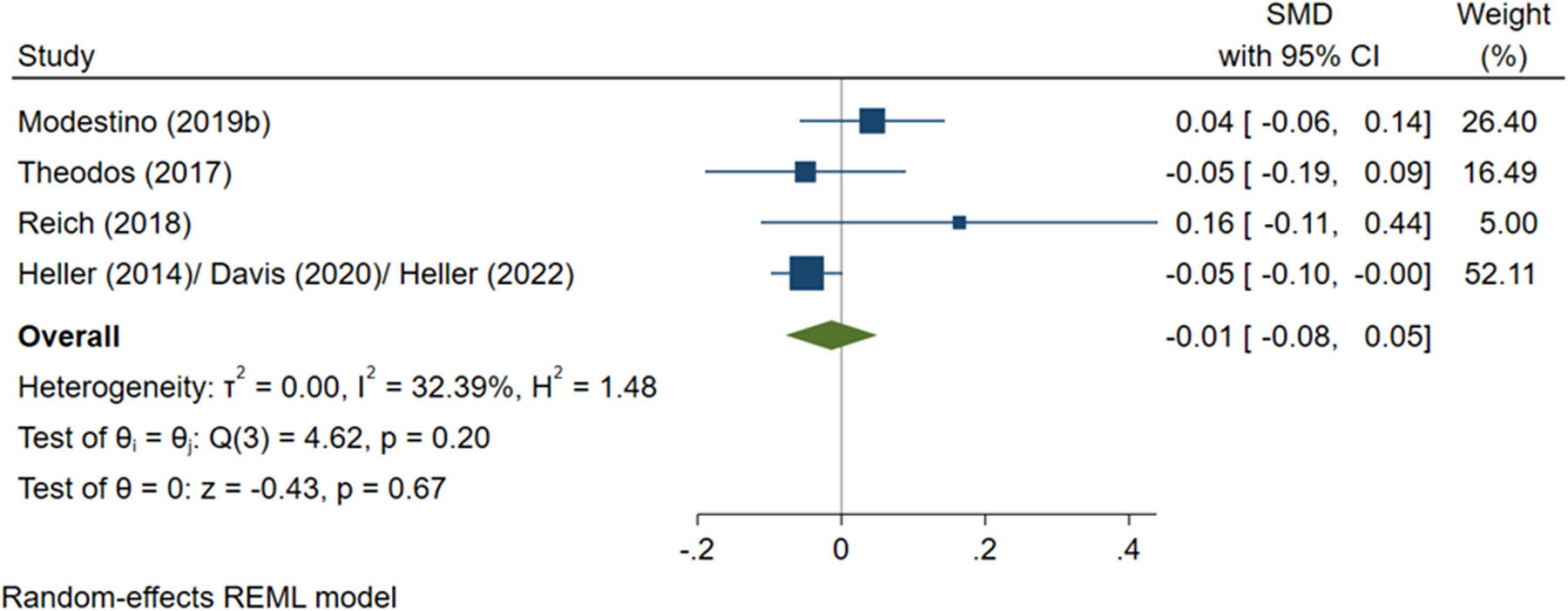
Impact of summer programme participation on overall test scores. *Source*: IES (2024).

Figure 13 displays the forest plot from the meta‐analysis of the impact of participation in a summer programme on overall academic scores. As all the interventions included in this analysis are summer employment programmes, there is no need to split by programme type.

The average effect size for summer employment programmes is negative although insignificant (SMD = −0.01, 95% confidence interval = −0.08, 0.06), suggesting that participation in a summer employment programme does not have a significant impact on overall test scores. When pooled together, the previously mentioned insignificant impacts from the three studies evaluating One Summer Chicago (once transformed to impacts of participation) produces a significant effect size (SMD = −0.05, 95% confidence interval = −0.10, 0.00).

The *p*‐value from the homogeneity test (*p* = 0.20) indicates that there is no statistically significant evidence of between‐study heterogeneity. None of the interventions included in this analysis targeted individuals from ethnic minorities.

Removing the result from Modestino (2019b) as it is the only study in this analysis that reports impacts of allocation that require transforming to impacts of participation, or the combined result from the studies evaluating One Summer Chicago because this is the highest weighted effect size, has negligible impacts on the overall findings.

Lastly, the impacts of summer programmes on English, mathematics and overall academic scores are pooled. Fourteen studies across 12 interventions report impacts of allocation to a summer education programme on some test scores:

▪ Future Foundations summer school programme:
  - Gorard et al. (2014) – Key Stage 2 to GL Assessment Progress in English/Mathematics gain score; autumn after the summer programme.
▪ New York City Summer Youth Employment Program:
  - Leos‐Urbel (2014) – standardised English/mathematics Regents exam score; year after the summer programme.
▪ Boston Summer Youth Employment Program:
  - Modestino (2019b) – overall Grade Point Average; year after the summer programme.
▪ Higher Achievement:
  - Herrera et al. (2013) – standardised Stanford Achievement Test 10 reading/mathematics (problem‐solving) score; spring after first programme summer.
▪ Summer Active Reading Programme:
  - Maxwell et al. (2014) – New Group Reading Test score; 3 months after the summer programme.
▪ Discover Summer School:
  - Torgerson et al. (2014) – Progress in English reading/writing/spelling and grammar score; 3 weeks after the summer programme.
▪ Building Educated Leaders for Life:
  - Somers et al. (2015) – Group Mathematics/Reading Assessment and Diagnostic Examination score; autumn after the summer programme.
▪ Urban Alliance:
  - Theodos et al. (2017) – cumulative Grade Point Average; 1 year after the summer programme.
▪ English Learner Summer School:
  - Johnson (2020) – standardised California English Language Development Test overall score; year after the summer programme.
▪ Elevate Match summer programme:
  - Snipes et al. (2015) – Mathematics Diagnostic Testing Project Algebra Readiness test score; autumn after the summer programme.
▪ Tenmarks:
  - Lynch and Kim (2017) – standardised National and District curriculum‐based mathematics assessment score; autumn after the summer programme.
▪ One Summer Chicago:
  - Heller (2014) – overall Grade Point Average; year after the summer programme.
  - Davis and Heller (2020) – overall Grade Point Average; year after the summer programme.
  - Heller (2022) – overall Grade Point Average; year after the summer programme.

**Figure 14:**
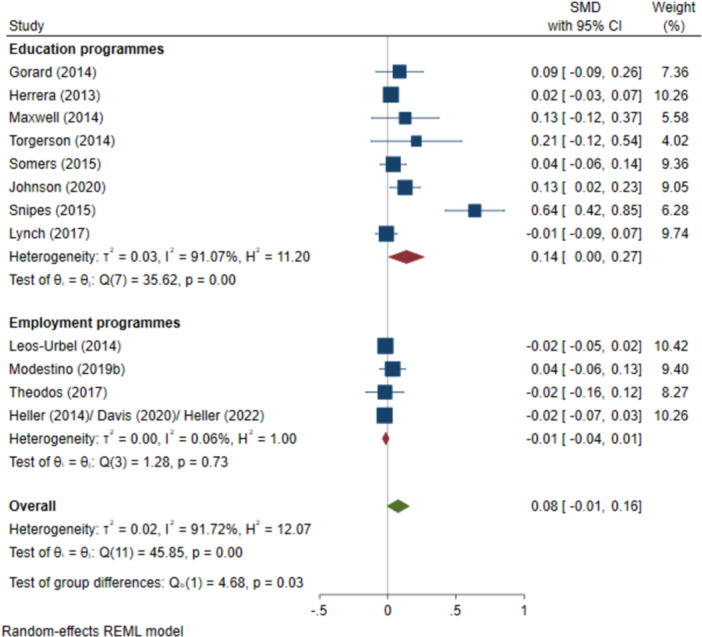
Impact of summer programme allocation on all test scores. *Source*: IES (2024).

Figure 14 displays the forest plot from the meta‐analysis of the impact of allocation to a summer programme on test scores, split by summer education and employment programmes.

The test of group differences indicates that the difference in the average effect size between summer education and summer employment programmes is statistically significant, therefore we do not focus on the combined findings for both summer programme types. The average effect size for summer education programmes is positive and statistically significant (SMD = 0.14, 95% confidence interval = 0.00, 0.27), suggesting that individuals who are allocated to a summer education programme have significantly better test scores following the programme than those who are not. An SMD of 0.14 translates to, for instance, an increase in overall Grade Point Average (as measured by Modestino (2019b)) of 0.14 for those allocated to a summer education programme. In contrast, summer employment programmes have a negative although insignificant average effect size (SMD = −0.01, 95% confidence interval = −0.04, 0.01), suggesting that allocation to a summer employment programme does not have a significant impact on test scores post‐programme.

Within the summer education programmes sub‐group, the *p*‐value from the homogeneity test (*p* = 0.00) indicates that there is statistically significant evidence of between‐study heterogeneity, reducing the applicability of the average effect size to all summer education programmes. This is not the case for summer employment programmes (*p* = 0.73). As there are 12 interventions covered by this analysis, meta‐regression as well as sub‐group analysis are used to examine heterogeneity. The only statistically significant differences on average effect size between sub‐groups are:

▪ between the one programme that targets disadvantaged areas (schools) with a high proportion of individuals with experience of or at risk of involvement with the criminal justice system, studied by Heller (2014), Davis and Heller (2020) and Heller (2022) (SMD = −0.02, 95% confidence interval = −0.07, 0.03), and those programmes that do not (SMD = 0.09, 95% confidence interval = −0.01, 0.18) (given this is based on a sub‐group of one study, the external validity of this finding is quite limited); and
▪ between those programmes that target socioeconomically disadvantaged areas (SMD = −0.01, 95% confidence interval = −0.03, 0.02) and those that do not (SMD = 0.21, 95% confidence interval = −0.00, 0.42), apart from Lynch and Kim (2017) it is the summer employment programmes that target socioeconomically disadvantaged areas and the summer education programmes that do not.

None of the interventions included in this analysis targeted individuals from ethnic minorities.

Removing the result from Snipes et al. (2015), which is something of an outlier, reduces the average effect size of summer education programmes to 0.04 which is still marginally significant (95% confidence interval = 0.00, 0.08).

Table 8 displays the results from running meta‐regression for this outcome.

**Table 8:** Results from meta‐regression for impact of summer programme allocation on test scores.

	Coefficient (SE)	Significance	95% Confidence interval	
			Lower	Upper
Intercept	0.485 (0.296)	0.101	−0.095	1.064
Education programme	−0.089 (0.145)	0.538	−0.374	0.195
UK intervention	0.077 (0.149)	0.605	−0.215	0.369
‘In whole’ summer programme	−0.268 (0.150)	0.073	−0.561	0.025
High quality study design	−0.004 (0.190)	0.983	−0.377	0.368
Targets area‐based socioeconomic disadvantage	−0.213 (0.125)	0.089	−0.459	0.033
Targets area‐based experienced/at risk of involvement with the criminal justice system	−0.022 (0.162)	0.893	−0.340	0.296

*Source*: IES (2024).

The results from the meta‐regression indicate that, after controlling for other factors, there are no statistically significant differences on average effect size across any of the sub‐groups included. Some of the estimated coefficients are relatively large however, particularly for the dummy of whether the intervention was an ‘in whole’ summer programme and whether it targeted socioeconomically disadvantaged areas. The high degree of overlap between various sub‐groups, as well as the relatively small number of studies included in this analysis, is likely partly responsible for a lack of statistically significant differences. For instance, removing the study quality moderator, which is highly insignificant, results in the estimated differences in effect size between ‘in whole’ and ‘in part’ summer programmes and those that do and do not target socioeconomically disadvantaged areas becoming statistically significant.

Fifteen studies across 12 interventions report impacts of participation in a summer programme on test scores, or report impacts of allocation that could be transformed into impacts of participation. The estimates of impact of allocation to the summer programme previously outlined from Leos‐Urbel (2014), Herrera et al. (2013), Johnson (2020) and Lynch and Kim (2017) cannot be translated to estimates of impact of participation in the summer programme, as they report standardised test scores with insufficient information to transform them. The additional studies in this analysis are:

▪ Summer Learning Journey:
  - Williamson et al. (2020) – PAT reading comprehension/e‐asTTle writing gain score; autumn after the summer programme
▪ STEP‐UP:
  - Reich (2018) – overall Grade Point Average; year after the summer programme.
▪ Future Foundations summer school programme:
  - Siddiqui et al. (2014) – Key Stage 2 mathematics/reading/writing to post‐test gain score; autumn after the summer programme.
▪ Summer Success Academy:
  - Mariano and Martorell (2013) – standardised English/mathematics assessment score; spring after the summer programme.
▪ Aim High:
  - Pyne et al. (2020) – California Assessment of Student Performance and Progress English/mathematics score; within the 2 years after the summer programme.

**Figure 15:**
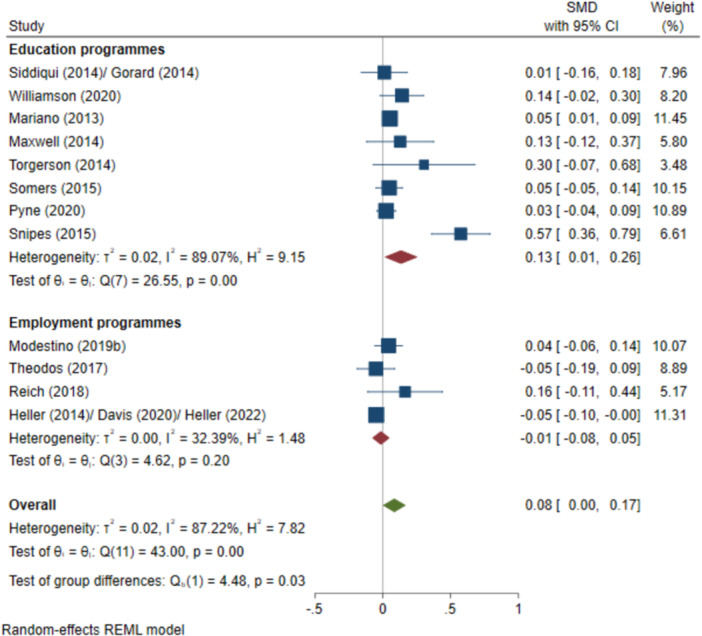
Impact of summer programme participation on all test scores. *Source*: IES (2024).

Figure 15 displays the forest plot from the meta‐analysis of the impact of participation in a summer programme on test scores, split by summer education and employment programmes.

The test of group differences indicates that the difference in the average effect size between summer education and summer employment programmes is statistically significant, therefore we do not focus on the combined findings for both summer programme types. The average effect size of summer education programmes is positive and statistically significant (SMD = 0.13, 95% confidence interval = 0.01, 0.26), suggesting that participation in a summer education programme has a positive impact on test scores. An SMD of 0.13 translates to, for example, an increase in overall Grade Point Average (as measured by Modestino (2019b)) of 0.13 for those participating in a summer education programme. Summer employment programmes have a negative although insignificant average effect size (SMD = −0.01, 95% confidence interval = −0.08, 0.06), suggesting that participation in a summer employment programme does not have a significant impact on test scores.

Within the summer education programmes sub‐group, the *p*‐value from the homogeneity test (*p* = 0.00) indicates that there is statistically significant evidence of between‐study heterogeneity, reducing the applicability of the average effect size to all summer education programmes. This is not the case for summer employment programmes (*p* = 0.20). As there are 12 interventions covered in this analysis, meta‐regression as well as sub‐group analysis are used to examine heterogeneity. Instances of statistically significant differences on average effect size between sub‐groups are:

▪ between the one programme that targets areas (schools) with a high proportion of individuals with experience of or at risk of involvement with the criminal justice system, studied by Heller (2014), Davis and Heller (2020) and Heller (2022) (effect size (SMD) = −0.04, 95% confidence interval = −0.12, 0.4), and those programmes that do not (SMD = 0.09, 95% confidence interval = −0.01, 0.18); and
▪ between ‘in whole’ summer programmes (SMD = 0.03, 95% confidence interval = −0.00, 0.07) and the one ‘in part’ summer programme, studied by Snipes et al. (2015) (SMD = 0.57, 95% confidence interval = 0.36, 0.79).

As both of these findings are based on a sub‐group of one study, the external validity of these findings are quite limited. None of the interventions included in this analysis targeted individuals from ethnic minorities.

Removing the result from Snipes et al. (2015), as an outlier, from the analysis reduces the average effect size (SMD) of summer education programmes to 0.05 which remains significant (95% confidence interval = 0.02, 0.08), and of all summer programmes to 0.03 which becomes marginally insignificant (95% confidence interval = −0.00, 0.07). Removing results that are produced by transforming impacts of allocation to impacts of participation has a negligible impact on the findings.

Table 9 displays the results from running meta‐regression for this outcome (a dummy for interventions targeting socioeconomically disadvantaged areas was not included as these largely overlap with interventions targeting socioeconomically disadvantaged areas).

**Table 9:** Results from meta‐regression for impact of summer programme participation on test scores.

	Coefficient (SE)	Significance	95% Confidence interval	
			Lower	Upper
Intercept	0.537 (0.124)	0.000	0.293	0.781
Education programme	0.028 (0.043)	0.523	−0.057	0.112
UK intervention	0.033 (0.069)	0.634	−0.103	0.169
‘In whole’ summer programme	−0.523 (0.111)	0.000	−0.740	−0.305
High‐quality study design	0.010 (0.035)	0.779	−0.059	0.078
Targets area‐based experienced/at risk of involvement with the criminal justice system	−0.073 (0.047)	0.120	−0.164	0.019

*Source*: IES (2024).

The results from the meta‐regression indicate that, after controlling for other factors, the average effect size of ‘in whole’ summer programmes is 0.52 lower than ‘in part’ summer programmes which is significant at the 95% confidence level. Differences across the other sub‐groups are not significant. Given the relatively small number of studies included in this analysis, and the significant overlap between various sub‐groups, these results should be treated with a degree of caution.

#### 14.2.6 On‐time completion of secondary education

*Summary*:

▪ The one study of a summer education programme that evaluates this outcome finds no impact, although it is of low quality.
▪ Of the two studies evaluating summer employment programmes, the one of high quality finds no impact whilst the one of low quality finds a positive impact.

Three studies across three interventions report impacts of allocation to/participation in a summer programme on the likelihood of completing secondary education on time:

▪ New York City Summer Youth Employment Program:
  - Valentine et al. (2017) – proportion that graduated high school in 4 years; up to 5 years after the programme.
▪ English Learner Summer School:
  - Johnson (2020) – proportion that graduated high school in 5 years; up to 3 years after the programme.
▪ STEP‐UP:
  - Reich (2018) – proportion on track to graduate in 4 years; up to 1 year after the programme.

Theodos et al. (2017) also evaluates the likelihood of graduating high school, however it is not certain that individuals within the sample will be graduating on time. In the context of the group of individuals studied by Johnson (2020) (ESOL learners that are recent migrants to the US), graduation from high school in 5 years constitutes graduating on time.

Valentine et al. (2017) found that allocation to the summer employment programme had no significant impact on the proportion of students that graduated high school on time.

Johnson (2020) found that allocation to the summer education programme had no significant impact on the proportion of students that graduated high school on time.

Reich (2018) found that participation in the summer employment programme had a significant impact on the likelihood of completing secondary education on time, equating to an effect size (log odds ratio) of 0.96 (95% confidence interval = 0.32, 1.60). This translates to an odds ratio of 2.61 (95% confidence interval = 1.38, 4.95), meaning that individuals who participate in the summer employment programme are 2.61 times more likely to complete secondary education on time than those that do not. To support interpretation of these findings, it is important to note that the two studies that find larger positive effect sizes, Johnson (2020) and Reich (2018) – whilst insignificant, the central estimate of the log odds ratio from the latter is 0.78 – have low‐quality study designs, while Valentine et al. (2017), who finds a much smaller and overall insignificant effect size, has a high‐quality study design.

#### 14.2.7 Negative behavioural outcomes

*Summary*:

▪ The one study of a summer education programme that evaluates this outcome finds a negative (i.e., beneficial) impact, although it is of low quality.
▪ Summer employment programmes appear to have no impact on this outcome, although one study of high quality finds a positive (i.e., detrimental) impact.

Three studies across three interventions report impacts of allocation to a summer programme on the likelihood of having a negative behavioural outcome post‐programme:

▪ Urban Alliance:
  - Theodos et al. (2017) – proportion suspended; year after the summer programme.
▪ Boston Summer Youth Employment Program:
  - Modestino (2019b) – proportion that have had a disciplinary incident; year after the summer programme.
▪ One Summer Chicago:
  - Heller (2022) – proportion suspended; year after the summer programme.

Theodos et al. (2017) does not find that allocation to the summer employment programme has a significant impact on the likelihood of being suspended or chronically absent.

Modestino (2019b) found that allocation to the summer employment programme had no significant impact on the likelihood of having a disciplinary incident.

Heller (2022) found that allocation to the summer employment programme had a significant positive impact on the likelihood an individual had a suspension, equating to an effect size (log odds ratio) of 0.17 (95% confidence interval = 0.01, 0.34) and an odds ratio of 1.19 (95% confidence interval = 1.01, 1.40). So, an individual who participated in the summer employment programme was 1.19 times more likely to have a suspension in the first year post‐randomisation that someone who did not.

Five studies across five interventions report impacts of participation in a summer programme on the likelihood of having a negative behavioural outcome, or report impacts of allocation to a summer programme which could be transformed into impacts of participation. In addition to the three studies previously detailed, the others are:

▪ STEP‐UP:
  - Reich (2018) – proportion having removal/behaviour referral/suspension; year after the summer programme.
▪ Aim High:
  - Pyne et al. (2020) – proportion suspended; across the 3 years after the summer programme.
▪ Youth Violence Prevention Funder Learning Collaborative summer employment programme:
  - Sum (2015) – change in the proportion that reported skipping classes without an excuse to not doing so; from the start to the end of the programme summer.

The extent of attrition (overall and differential rates) for the self‐reported survey‐based outcome measure used by Snipes et al. (2015) is highly concerning, introducing a potential source of bias into the estimate produced.

Reich (2018) largely finds that participation in the summer employment programme has no significant impact on the likelihood of having a negative behavioural outcome, apart from the likelihood of having a suspension where one of the two treatment arms has a significant positive (i.e., increasing the likelihood) impact.

Pyne et al. (2020) found that participation in the summer education programme has a significant negative (i.e., reducing the likelihood) impact on the likelihood that an individual has a suspension, equating to an effect size (log odds ratio) of −1.55 (95% confidence interval = −3.13, 0.03 – note that the specifics of the process for constructing the effect size means that the constructed effect size is insignificant) and an odds ratio of 0.21 (95% confidence interval = 0.04, 1.03). So, an individual who participated in the summer education programme is 0.21 times as likely to have a suspension across the 3 years after the summer programme as someone who did not.

Sum (2015) found that participation in a summer employment programme has no significant impact on the proportion of individuals who went from reporting that they had skipped classes without an excuse to not doing so from the start to the end of the summer (insufficient information was available to construct an effect size, therefore this is not included in the meta‐analysis that follows).

**Figure 16:**
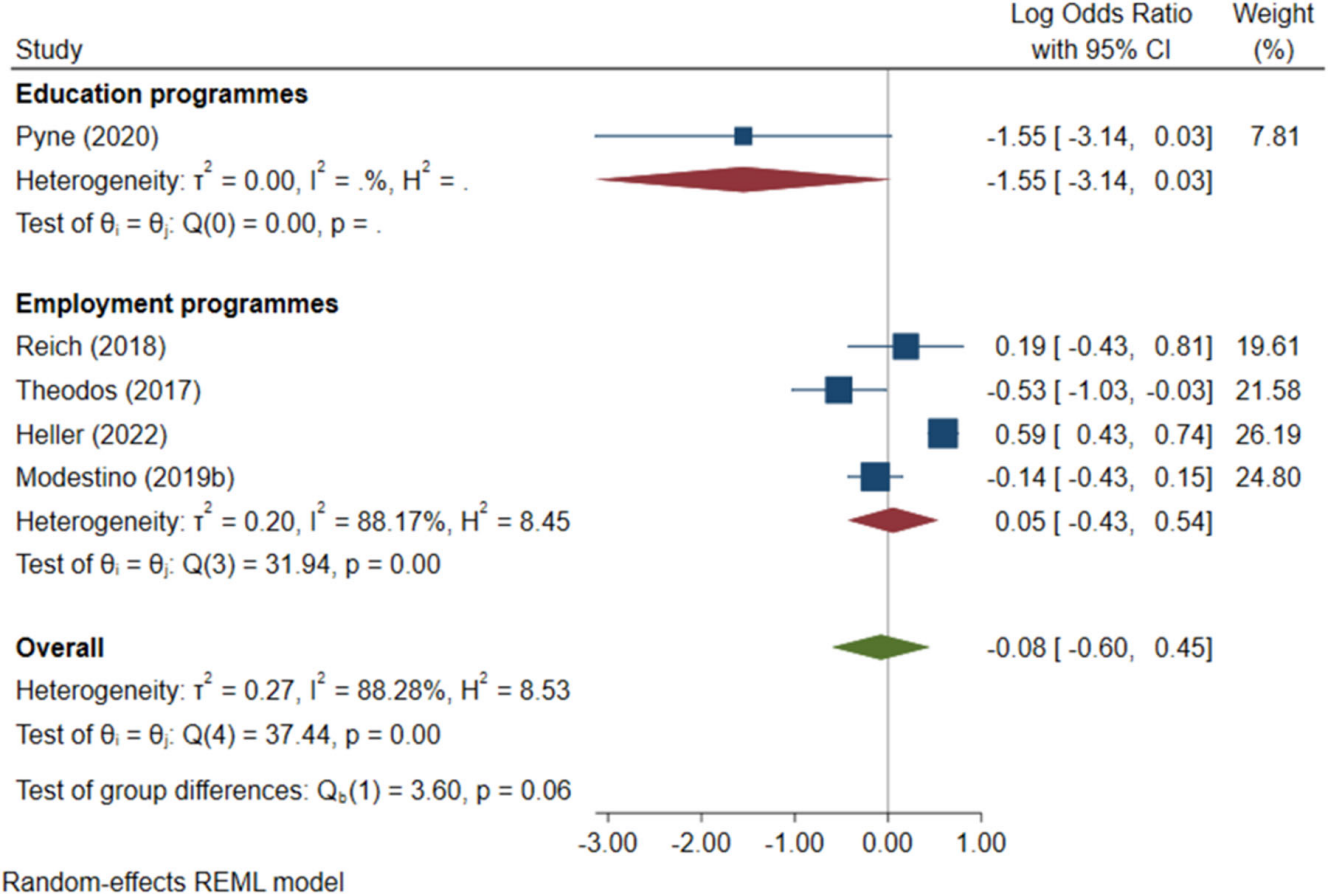
Impact of summer programme participation on likelihood of having negative behavioural outcome. *Source*: IES (2024).

Figure 16 displays the forest plot from the meta‐analysis of the impact of participation in a summer programme on the likelihood of having a negative behavioural outcome, split by summer education and employment programmes.

The average effect size for summer education programmes is negative whilst for summer employment programmes it is positive, although both are insignificant (log odds ratio (summer education programmes) = −1.55, 95% confidence interval = −3.14, 0.03; log odds ratio (summer employment programmes) = 0.05, 95% confidence interval = −0.43, 0.54). This suggests that participation in either a summer education nor employment programme does not have a significant impact on the likelihood that an individual has a negative behavioural outcome. Note that this finding for summer education programmes is only based on the result from one study. The test of group differences indicates that there is no statistically significant difference on average effect size between summer education and employment programmes.

Within summer employment programmes, the *p*‐value from the homogeneity test (*p* = 0.00) indicates that there is evidence of between‐study heterogeneity, reducing the applicability of the average effect size to all summer employment programmes. As there are less than 10 interventions covered in this analysis, sub‐group analysis is used to examine heterogeneity:

▪ the one programme that targeted areas containing individuals with experience of or at risk of involvement with the criminal justice system, studied by Heller (2022), had a significantly different (higher, i.e., less of a reduction) effect size to those programmes that did not target these areas (average effect size (log odds ratio) = −0.24, 95% confidence interval = −0.59, 0.11).

None of the interventions included in this analysis targeted individuals from ethnic minorities.

#### 14.2.8 Application to higher education

*Summary*:

▪ Consistent finding across studies of both summer programme types of no impact on this outcome, although this is based on only three studies.

Three studies across three interventions report impacts of allocation to/participation in a summer programme on the likelihood of applying to higher education. These are:

▪ Widening participation summer schools:
  - Taylor (2022) – proportion that applied to university; 16–18 months after the summer programme.
▪ Urban Alliance:
  - Theodos et al. (2017) – proportion that applied to college; year after the summer programme.
▪ STEM summer programmes:
  - Cohodes et al. (2022) – proportion that applied to 4‐year college; year after the summer programme.

The extent of attrition (overall and differential rates) for the self‐reported survey‐based outcome measures used by Taylor (2022) and Theodos et al. (2017) are highly concerning, introducing a potential source of bias into the estimates produced.

Taylor (2022) finds no significant impact of allocation to the summer education programme on the likelihood of applying to higher education.

Cohodes et al. (2022) finds no significant impact of allocation to the summer education programme on the likelihood of applying to higher education.

Note that Taylor (2022) and Cohodes et al. (2022) study interventions that target ethnic minority young people.

Theodos et al. (2017) finds no significant impact of allocation to or participation in the summer employment programme on the likelihood of applying to higher education. Oddly, the reported estimated impact of participation in the summer employment programme is less than the reported estimated impact of allocation to the summer employment programme – this might suggest that drop‐out from the treatment group was non‐random.

#### 14.2.9 Progression to higher education

*Summary*:

▪ Summer education programmes appear to have no impact on this outcome, although two of the five studies evaluating this find positive impacts.
▪ Summer employment programmes appear to have no impact on this outcome – the one study that does find a positive impact uses an intermediate measure of this outcome that differs from those used by the two other studies that find no impact.

Nine studies across eight interventions report impacts of allocation to a summer programme on the likelihood of attending higher education:

▪ New York City Summer Youth Employment Program:
  - Valentine et al. (2017) – proportion enroled in college; across the 5 years after the summer programme.
▪ Higher Achievement:
  - Garcia et al. (2020) – proportion ever attended college; across 12–14 years after first programme summer.
▪ No‐MisMatch Program:
  - Gehring et al. (2018) – proportion enroled in college; year after the summer programme.
▪ Texas developmental summer bridge programme:
  - Wathington et al. (2016) – proportion enroled in college; across 2 years after the summer programme.
▪ Scholars Academy:
  - Henson (2018) – proportion retained in college; second year after the summer programme.
▪ Urban Alliance:
  - Theodos et al. (2017) – proportion attended college; up to 1/2 years after the summer programme.
▪ STEM summer programmes:
  - Cohodes et al. (2022) – proportion enroled in/attended 4‐year college; second, third, fourth and fifth years after the summer programme.
  - Robles (2018) – proportion enroled in college; across the 3 years after the summer programme.
▪ Boston Summer Youth Employment Program:
  - Modestino and Paulsen (2019a) – proportion that plan to attend 2‐/4‐year college; end of the summer programme.

The measure used by Modestino and Paulsen (2019a) is a somewhat different measures to those evaluated by other studies – intention to progress to higher education as opposed to realised progression to higher education – which should be borne in mind. The survey that this outcome is sourced from also has highly differential response rates between the treatment and control groups, introducing a potential source of bias into the estimates produced.

**Figure 17:**
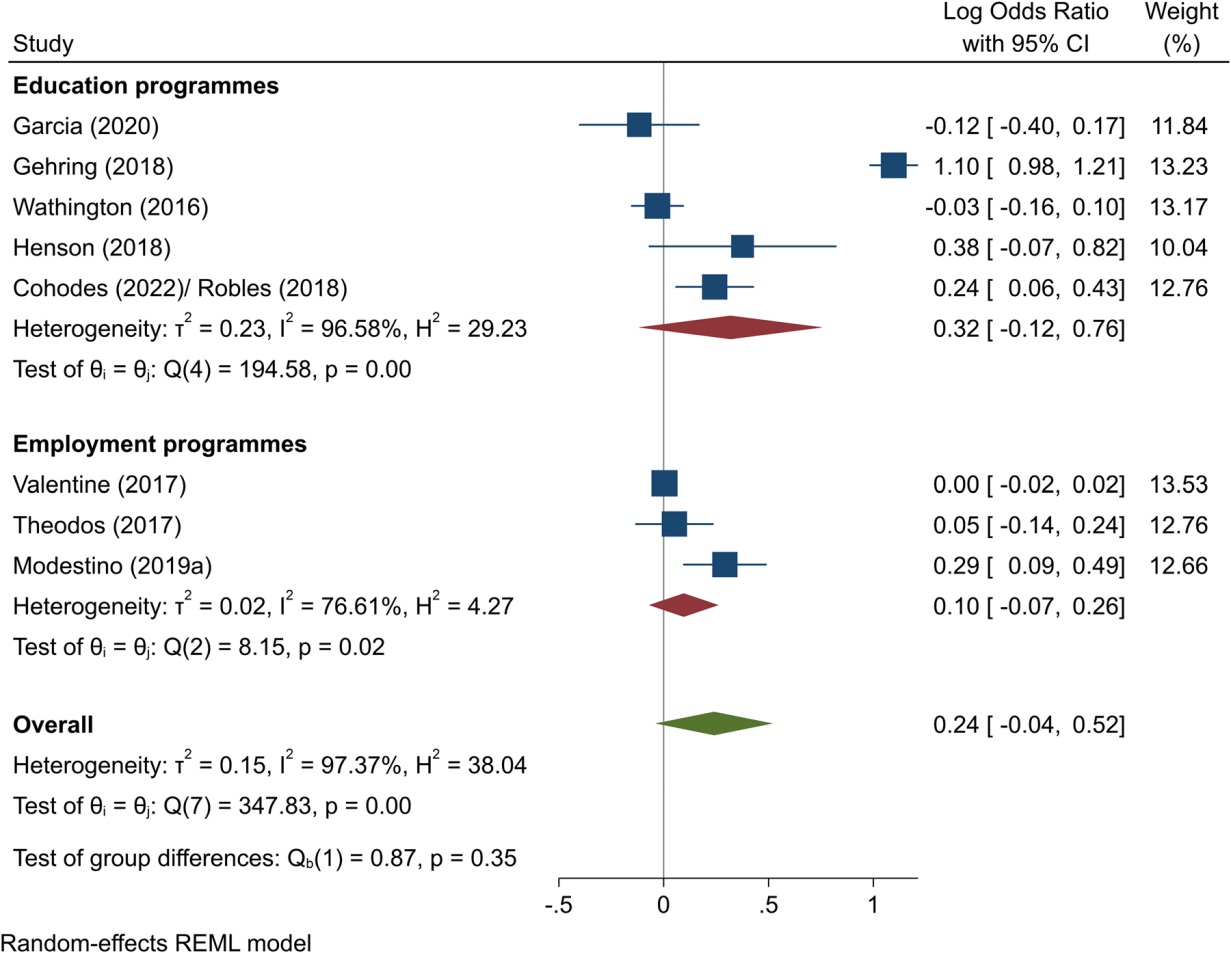
Impact of summer programme allocation on likelihood of attending higher education. *Source*: IES (2024).

Figure 17 displays the forest plot from the meta‐analysis of the impact of allocation to a summer programme on the likelihood of attending higher education, split by summer education and employment programmes.

Summer education programmes have a positive although insignificant average effect size (SMD = 0.32, 95% confidence interval = −0.12, 0.76), suggesting that allocation to a summer education programme does not have a significant impact on the likelihood of attending higher education. Summer employment programmes also have a positive although insignificant average effect size (SMD = 0.10, 95% confidence interval = −0.07, 0.26) suggesting that allocation to a summer employment programme does not have a significant impact on the likelihood of attending higher education. The test of group differences indicates that there is no significant difference on average effect size between summer education and employment programmes. Overall, allocation to a summer programme has a positive although insignificant average effect size (SMD = 0.24, 95% confidence interval = −0.04, 0.52), suggesting that allocation to a summer programme does not have a significant impact on the likelihood of attending higher education.

Within each of the sub‐groups, the *p*‐values from the homogeneity tests (summer education programmes: *p* = 0.00; summer employment programmes: *p* = 0.02) indicate that there is evidence of statistically significant between‐study heterogeneity, reducing the applicability of the average effect sizes to all summer programmes of each type. As there are less than 10 interventions covered in this analysis, we use further sub‐group analysis to examine heterogeneity. There are no statistically significant differences on average effect size between any sub‐groups of studies (by region of the intervention, intervention type, whether the intervention is an ‘in whole’ or ‘in part’ summer programme, the forms of disadvantage targeted, or study quality). Cohodes et al. (2022) and Robles (2018) study the one intervention evaluating this outcome that targets individuals from ethnic minorities (the impact estimates underlying the constructed effect size are themselves insignificant apart from one of the three treatment arms studied by Cohodes et al. (2022) 5 years after the summer programme).

Removing the result from Gehring et al. (2018) which is somewhat an outlier reduces the average effect size of summer education programmes (SMD = 0.09, 95% confidence interval = −0.11, 0.28) and all summer programmes (SMD = 0.09, 95% confidence interval = −0.03, 0.20) although both estimates are now marginally closer to being significant with narrower confidence intervals. Removing Modestino and Paulsen (2019a) which uses an intermediate (and potentially biased) outcome measure has a negligible impact on the findings.

Thirteen studies across 11 interventions report impacts of participation in a summer programme on the likelihood of entering higher education, or report impacts of allocation to a summer programme which could be transformed into impacts of participation. In addition to the nine studies previously detailed, the others are:

▪ University of North Carolina summer bridge programme:
  - Wachen et al. (2018) – proportion retained in college; second and third year after the summer programme.
▪ Excel State University summer bridge programme:
  - Anthony (2019) – proportion enroled in college; first and second year after the summer programme.
▪ New York City Summer Youth Employment Program:
  - Gelber et al. (2016) – proportion enroled in college; first, second, third and fourth year year after the summer programme.
▪ California State University (Los Angeles) Bridge Learning Community Model:
  - McEvoy (2012) – proportion retained in college; second year after the summer programme.

**Figure 18:**
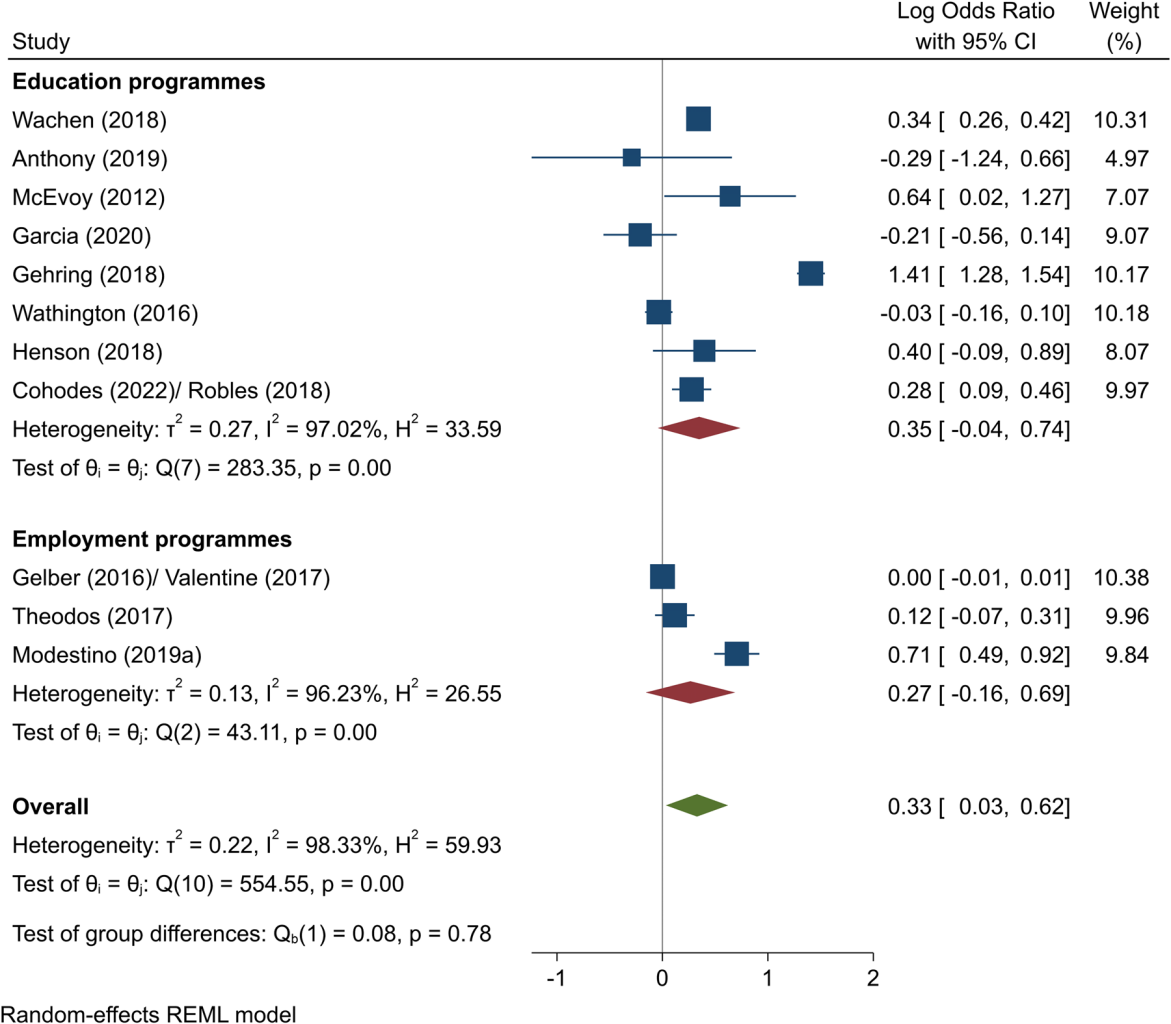
Impact of summer programme participation on likelihood of attending higher education. *Source*: IES (2024).

Figure 18 displays the forest plot from the meta‐analysis of the impact of participation in a summer programme on the likelihood of entering higher education, split by summer education and employment programmes.

Summer programmes have a positive and significant average effect size (log odds ratio = 0.33, 95% confidence interval = 0.03, 0.62), suggesting that participation in a summer programme has a positive impact on the likelihood of attending higher education. A log odds ratio of 0.33 translates to an odds ratio of 1.39 (95% confidence interval = 1.04, 1.90) meaning that individuals who participate in a summer programme are 1.39 times more likely to be in higher education than those that do not, post‐programme. Moreover, using as the assumed comparator risk the average in the proportions of control individuals entering higher education across all the included studies where this is reported, and excluding for Modestino and Paulsen (2019a) which measures an intermediate outcome, the number needed to treat through participation in a summer programme to see this outcome among one more individual is 14. The average effect size for summer education programmes is positive although insignificant (log odds ratio = 0.35, 95% confidence interval = −0.04, 0.74), as is the case for summer employment programmes (log odds ratio = 0.27, 95% confidence interval = −0.16, 0.69). The test of group differences indicates that there is no significant difference on average effect size between summer education and employment programmes.

Within each of the sub‐groups, the *p*‐values from the homogeneity tests (*p* = 0.00 for both summer programme types) indicate that there is evidence of statistically significant between‐study heterogeneity. As there are 11 interventions covered in this analysis, meta‐regression as well as sub‐group analyses are used to examine heterogeneity. There are no statistically significant differences on average effect size between any sub‐groups of studies (by region of the intervention, intervention type, whether the intervention is an ‘in whole’ or ‘in part’ summer programme, the forms of disadvantage targeted, or study quality), including between the one intervention that targets ethnic minority individuals, studied by Cohodes et al. (2022) and Robles (2018), and those that do not.

Removing the result from Gehring et al. (2018), an outlier, reduces the average effect size of summer education programmes (log odds ratio = 0.17, 95% confidence intervals = −0.03, 0.38) and all summer programmes (log odds ratio = 0.21, 95% confidence intervals = 0.02, 0.39) although both figures are now more precisely estimated with narrower confidence intervals. Removing Modestino and Paulsen (2019a) which uses an intermediate (and potentially biased) outcome measure has an impact on the findings – participation in any summer programme no longer has a significant impact on the likelihood of progression to higher education (log odds ratio = 0.29, 95% confidence interval = −0.07, 0.11). Removing results that are produced by transforming reported impacts of allocation to impacts of participation has a negligible impact on the findings.

Table 10 displays the results from running meta‐regression for this outcome.

**Table 10:** Results from meta‐regression for impact of summer programme participation on entry to higher education.

	Coefficient (SE)	Significance	95% Confidence interval	
			Lower	Upper
Intercept	0.489 (1.833)	0.790	−3.103	4.081
Education programme	−0.427 (1.162)	0.713	−2.705	1.852
‘In whole’ summer programme	0.055 (0.946)	0.953	−1.798	1.908
High quality study design	0.215 (0.585)	0.713	−0.932	1.362
Targets area‐based socioeconomic disadvantage	−0.488 (0.822)	0.552	−2.100	1.123
Targets individuals with poor academic performance	0.354 (0.637)	0.578	−0.895	1.603

*Source*: IES (2024).

Each of the coefficients are insignificant, suggesting that once controlling for other factors, there are no statistically significant differences in effect size across the sub‐groups of interest. However, the direction and magnitude of some of the coefficients suggest some large differences across sub‐groups; this may suggest insignificance is a function of the analysis being underpowered due to a small sample of studies as opposed to there being no true differences in effect size for participation in a summer programme with these different characteristics.

#### 14.2.10 Completion of higher education

*Summary*:

▪ Summer education programmes appear to have a positive impact on this outcome.
▪ Summer employment programmes appear to have no impact on this outcome.

Four studies across three interventions report impacts of allocation to a summer programme on the likelihood of completing higher education:

▪ New York City Summer Youth Employment Program:
  - Valentine et al. (2017) – proportion that graduated college; across the 5 years after the summer programme;
▪ Urban Alliance:
  - Theodos et al. (2017) – proportion that completed 2‐years of college; up to 2 years after the summer programme;
▪ STEM summer programmes:
  - Cohodes et al. (2022) – proportion that graduated college; across the 7 years after the summer programme;
  - Robles (2018) – proportion that graduated college; across the 5 years after the summer programme.

Valentine et al. (2017) finds no significant impact of allocation to the summer employment programme on the likelihood of completing higher education.

The outcome measure from Theodos et al. (2017) is slightly different from the others, given the period these data cover. It might be expected that the impact of the programme on the completion of higher education would wear off over time, meaning that using this impact estimate would marginally overestimate the impact of allocation to summer programmes on completion of higher education. However, the effect size constructed using outcomes after 2 years from Theodos et al. (2017) that we include in the meta‐analysis is greater than the one constructed after 1 year, which should alleviate these concerns. Theodos et al. (2017) finds no significant impact of allocation to the summer employment programme on the likelihood of completing higher education.

Cohodes et al. (2022) and Robles (2018) largely find no significant impact of allocation to the summer education programme on the likelihood of completing higher education, aside for one of the three treatment arms from Cohodes et al. (2022). When pooled together, the effect size produced is significant.

Eight studies across seven interventions report impact of participation in a summer programme on the likelihood of completing higher education, or report impacts of allocation to a summer programme which could be transformed into impacts of participation. In addition to those previously outlined, the others are:

▪ University of North Carolina summer bridge:
  - Wachen et al. (2018) – proportion that graduated college; up to 6 years after the summer programme;
▪ California State University (Los Angeles) Bridge Learning Community Model:
  - McEvoy (2012) – proportion that graduated college; up to 9 years after the summer programme;
▪ RWJF Summer Medical and Dental Education Program:
  - Cosentino et al. (2015) – proportion that obtained a bachelor's degree; up to 5/6/7 years after the summer programme;
▪ STEM Enrichment Summer Bridge Program:
  - Ghazzawi et al. (2022) – proportion that graduated college; up to 4 years after the summer programme.

The outcome data in Consentino et al. (2015) is available up to a point, and there are three cohorts passing through the programme, therefore with different length evaluation periods.

**Figure 19:**
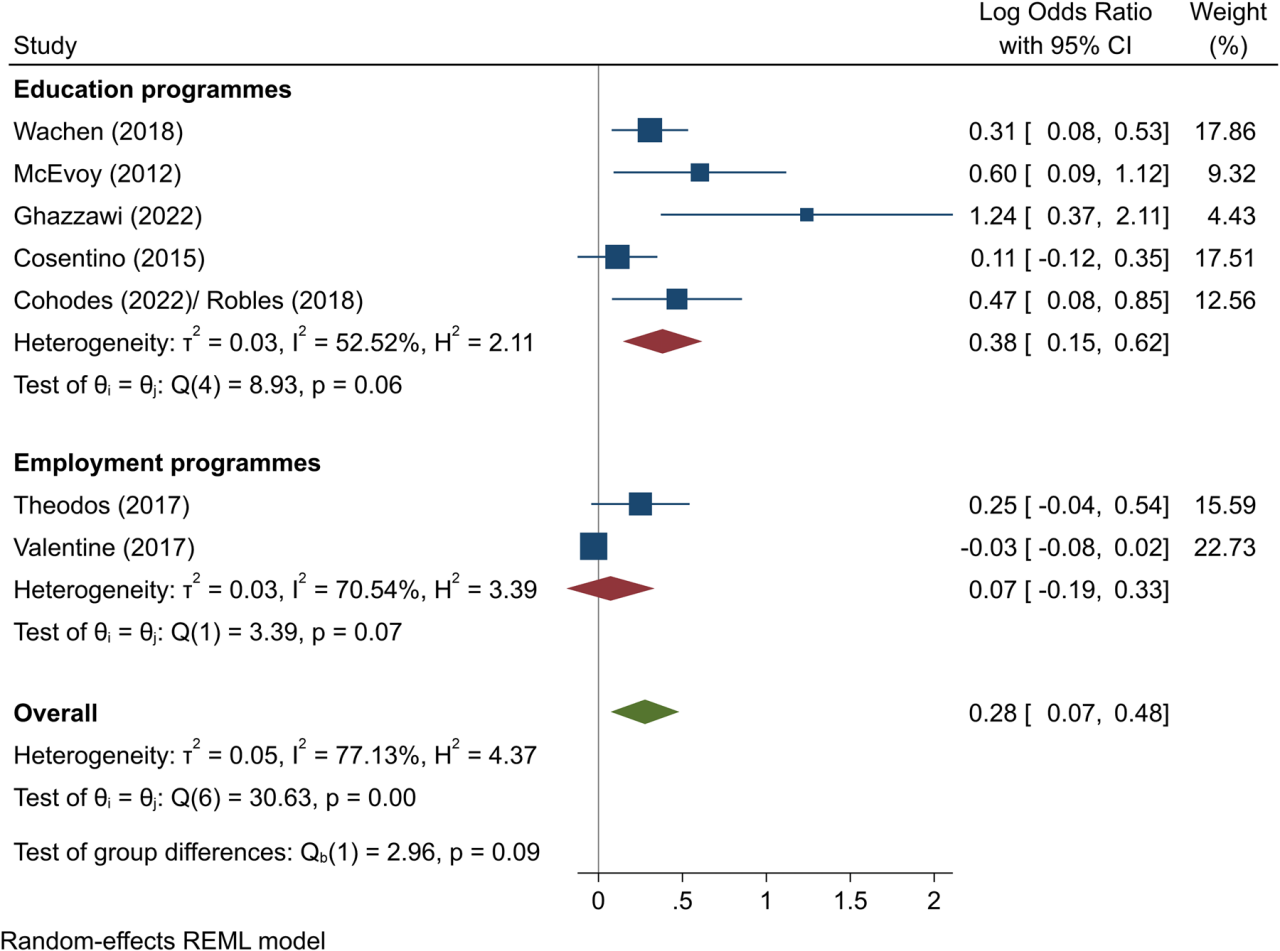
Impact of summer programme participation on likelihood of completing higher education. *Source*: IES (2024).

Figure 19 displays the forest plot from the meta‐analysis of the impact of participation in a summer programme on the likelihood of completing higher education, split by summer education and summer employment programmes.

The average effect size for summer education programmes is positive and significant (log odds ratio = 0.38, 95% confidence interval = 0.15, 0.62), suggesting that participation in a summer education programme has a positive impact on the likelihood of completing higher education. A log odds ratio of 0.38 translates to an odds ratio of 1.46 (95% confidence interval = 1.16, 1.86), meaning that individuals who participate in a summer education programme are 1.46 times more likely to complete higher education than those who do not, post‐programme. Additionally, using as the assumed comparator risk the average in the proportions of control individuals completing higher education across all the included studies evaluating summer education programmes for which it is reported, the number needing to be treated through participation in a summer education programme to see this outcome for one individual as a result of participation is 13. For summer employment programmes, the average effect size is positive but not significant (log odds ratio = 0.07, 95% confidence interval = −0.19, 0.33), suggesting that participation in a summer employment programme does not have a significant impact on the likelihood of completing higher education. The test of group differences does not indicate that there is a statistically significant difference on average effect size between summer education and summer employment programmes (at least, at the conventional 95% confidence level). Summer programmes overall have a positive and significant average effect size (log odds ratio = 0.28, 95% confidence interval = 0.07, 0.48); however, the analysis split by programme types suggests that any significant impact of participation in any summer programme on the likelihood of completing higher education is a result only of summer education programmes.

For both summer programme types, summer education programmes and summer employment programmes, the *p*‐value from the homogeneity test (*p* = 0.06, *p* = 0.07 and *p* = 0.00 respectively) indicate that there is statistically significant evidence of between‐study heterogeneity, reducing the applicability of the average effect size to all summer programmes of that type. As there are fewer than 10 interventions covered in this analysis, we use sub‐group analyses to examine heterogeneity. Instances of statistically significant differences on average effect size between sub‐groups are:

▪ between programmes that targeted socioeconomically disadvantaged areas (log odds ratio = 0.07, 95% confidence interval = −0.19, 0.33) and those that did not (log odds ratio = 0.38, 95% confidence interval = 0.15, 0.62);
▪ between programmes that targeted individuals with poor academic performance (log odds ratio = 0.57, 95% confidence interval = 0.13, 1.02) and those than that did not (log odds ratio = 0.14, 95% confidence interval = −0.06, 0.34); and
▪ between programmes that targeted first‐generation students individuals (log odds ratio = 0.52, 95% confidence interval = 0.21, 0.83) and those that did not (log odds ratio = 0.19, 95% confidence interval = −0.01, 0.40).

There is no statistically significant difference on average effect size between the interventions studied by Robles (2018), Cohodes et al. (2022), Ghazzawi et al. (2022) and Cosentino et al. (2015) that target ethnic minority individuals and those that do not.

Removing the result from Valentine et al. (2017), as it is the highest weighted effect size, increases the average effect size of summer programmes (log odds ratio = 0.32, 95% confidence interval = 0.16, 0.47) which is estimated more precisely with tighter confidence intervals. Also removing the result from Cohodes et al. (2022), as it is the other study, along with Valentine et al. (2017), that reports impacts of allocation which require transforming to impacts of participation, has a negligible additional effect on the findings.

#### 14.2.11 Higher education outcomes relating to STEM fields

*Summary*:

▪ Summer education programmes appear to have a positive impact on this outcome, although studies evaluate a wide range of outcomes and studies covering only three interventions evaluated this outcome.

Four studies across three interventions report impacts of allocation to or participation in a summer programme on higher education outcomes relating specifically to STEM fields:

▪ STEM summer programmes:
  - Robles (2018) – proportion graduated with STEM degree; up to 4/5/6 years after the summer programme.
  - Cohodes et al. (2022) – proportion graduated with STEM degree; up to 4/5 years after the summer programme.
▪ STEM Enrichment Summer Bridge Program:
  - Ghazzawi et al. (2022) – proportion graduated with STEM degree from the initial field of study, up to 4 years after the summer programme.
▪ RWJF Summer Medical and Dental Education Program:
  - Cosentino et al. (2015):
    – proportion that applied to medical or dental school; up to 5/7 years after the summer programme;
    – proportion that matriculated in medical or dental school; up to 5/7 years after the summer programme;
    – proportion that obtained bachelor's degree in health‐related or medical preparatory programme; up to 5/7 years after the summer programme; and
    – proportion that obtained bachelor's degree in STEM field; up to 5/7 years after the summer programme.

Robles (2018) finds that allocation to or participation in the summer education programme has a significant positive impact on the likelihood of graduating with a STEM degree, with an effect size (log odds ratio) of 0.52 (95% confidence interval = 0.24, 0.80) after 4 years. This translates to an odds ratio of 1.68 meaning individuals allocated to or participating in the summer education programme are 1.68 times more likely to graduate with a STEM degree than those who do not, after 4 years. The effect size decreases to 0.45 (95% confidence interval = 0.12, 0.77) after 5 years, translating to an odds ratio of 1.57, that is, individuals allocated to or participating in the summer education programme are 1.57 times more likely to graduate with a STEM degree than those who do not, after 5 years. After 6 years the impact is no longer significant.

Cohodes et al. (2022) finds that allocation to the summer education programme had a significant positive impact on the likelihood of graduating with a STEM degree, with an effect size (log odds ratio) of 0.33 (95% confidence interval = 0.11, 0.55) after 4 years. This translates to an odds ratio of 1.39 so individuals allocated to the summer education programme are 1.39 times more likely to graduate with a STEM degree than individuals who are not, after 4 years. After 5 years, the effect size increases to 0.52 (95% confidence interval = 0.15, 0.90), translating to an odds ratio of 1.68 so individuals allocated to the summer education programme are 1.68 times more likely to graduate with a STEM degree than individuals who are not, after 5 years. Cohodes et al. (2022) also finds that participation in a summer education programme has a significant positive impact on the likelihood of graduating with a STEM degree after 4 years, with an effect size (log odds ratio) of 0.44 (95% confidence interval = 0.15, 0.73). This translates to an odds ratio of 1.55, meaning individuals participating in the summer education programme are 1.55 times more likely to graduate with a STEM degree than individuals who do not post‐programme.

Ghazzawi et al. (2022) finds that participation in the summer education programme has a significant positive impact on the likelihood of graduating with a STEM degree from the initial field of study, estimating an effect size (log odds ratio) of 0.94 (95% confidence interval = 0.21, 1.67) and an odds ratio of 2.55 so individuals participating in the summer education programme are 2.55 times more likely to graduate with a STEM degree from the initial field of study than individuals who do not.

Cosentino et al. (2015) finds that participation in the summer education programme has a significant positive impact on the likelihood of applying to medical or dental school (effect size = 0.33, 95% confidence interval = 0.18, 0.48), enroling in medical or dental school (effect size = 0.44, 95% confidence interval = 0.28, 0.61) and achieving a bachelor's degree in a STEM field (effect size = 0.24, 95% confidence interval = 0.06, 0.41). These translate to odds ratios of 1.39 (95% confidence interval = 1.20, 1.62), 1.56 (95% confidence interval = 1.32, 1.83) and 1.27 (95% confidence interval = 1.06, 1.52) respectively. However, participation in the summer education programme has no impact on the likelihood of achieving a bachelor's degree in a health‐related or medical preparatory programme.

It is worth noting that all three of the interventions covered by these studies target individuals from ethnic minorities.

### 14.3 Employment outcomes

#### 14.3.1 Entry to employment

*Summary*:

▪ Summer employment programmes appear to have no impact on this outcome, although there is quite wide variation in findings across studies.
▪ Summer employment programmes also appear to have a significant negative impact on entry to employment outside the summer employment programme after the initial programme summer where the summer employment programme can be extended across years.

Four studies across three interventions report impacts of allocation to a summer programme on the likelihood of entering employment. These are:

▪ New York City Summer Youth Employment Program:
  - Gelber et al. (2016) – probability of being employed; across first, second, third and fourth years after the summer programme.
  - Valentine et al. (2017) – probability of being employed; across first, second, third, fourth and fifth years after the summer programme.
▪ Boston Summer Youth Employment Program:
  - Modestino and Paulsen (2019a) – proportion that plan to work in the autumn; end of the programme summer.
▪ One Summer Chicago:
  - Davis and Heller (2020) – probability of being formally employed; across first year and a half after the summer programme.

The measure used by Modestino and Paulsen (2019a) is somewhat different to those evaluated by other studies – intention to enter employment as opposed to realised entry to employment, that is, an intermediate employment outcome measure – which should be borne in mind. The survey that this outcome is sourced from also has highly differential response rates between the treatment and control groups, introducing a potential source of bias into the estimate produced.

Gelber et al. (2016) finds a positive significant impact of allocation to the summer employment programme on the probability of an individual being employed in the first year after the summer employment programme, equating to an effect size (log odds ratio) of 0.05 (95% confidence interval = 0.04, 0.07) and an odds ratio of 1.05 (95% confidence interval = 1.04, 1.07). So, individuals allocated to the summer employment programme are 1.05 times more likely to be employed in the first year after the summer employment programme than those who are not. Gelber et al. (2016), however, does not find any significant impacts in the second, third or fourth years after the summer employment programme.

Valentine et al. (2017) finds that allocation to the summer employment programme has a positive impact on the likelihood of entering employment in the short term, with a significant average effect size (log odds ratio) 1 year after the programme equal to 0.04 (95% confidence interval = 0.02, 0.05), which translates to an odds ratio of 1.04 (95% confidence interval = 1.02, 1.05). This means that individuals who are allocated to the summer employment programme are 1.04 times more likely to enter employment than those who are not, 1 year post‐programme. However, there is no significant impact across the other periods across which the outcome is measured.

Modestino and Paulsen (2019a) finds that allocation to the summer employment programme has a significant negative impact on short‐term work plans, equating to an effect size (log odds ratio) of −0.30 (95% confidence interval = −0.51, −0.08) which translates to an odds ratio of 0.74 (95% confidence interval = 0.60, 0.92). So, individuals allocated to the summer employment programme are 0.74 times as likely to report that they planned to work in the autumn at the end of the programme summer than those who are not.

Davis and Heller (2020) does not find any significant impact of allocation to the summer employment programme on the probability of an individual being formally employed in the first year and a half after the summer employment programme.

Five studies across four interventions report impacts of participation in a summer programme on the likelihood of entering employment, or report impacts of allocation to a summer programme which could be transformed into impacts of participation. In addition to the two previously outlined, these are:

▪ Urban Alliance:
  - Theodos et al. (2017) – proportion employed; one and 2 years after the summer programme.

Note that Theodos et al. (2017) uses a self‐reported measure of employment and is based on a single point in time, whereas the other studies apart from Modestino and Paulsen (2019a) use administrative datasets to construct the outcome and consider whether the individual was employed across a period of time. The extent of attrition (overall and differential rates) for the survey used to source the outcome measure for Theodos et al. (2017) is highly concerning, introducing a potential source of bias into the estimates produced.

Theodos et al. (2017) finds no significant impact of participation in the summer employment programme on whether an individual reported as being employed 1 or 2 years after the summer employment programme.

**Figure 20:**
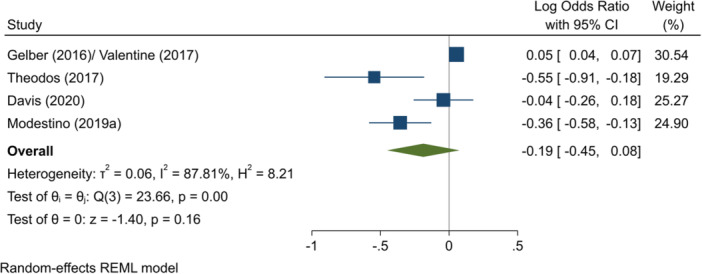
Short‐term impact of summer employment programme participation on likelihood of entering employment. *Source*: IES (2024).

Figure 20 displays the forest plot from the meta‐analysis of the impact of participation in a summer programme on the likelihood of entering employment in the shorter term, that is, 1 year post‐programme. As all the interventions included in this analysis are summer employment programmes, no split is included by programme type.

Summer employment programmes have a negative although insignificant average effect size (log odds ratio = −0.19, 95% confidence interval = −0.45, 0.08), suggesting that participation in a summer employment programme does not have a significant impact on the likelihood of entering employment in the short term. The impact estimate used to construct the effect size for Theodos et al. (2017) is itself insignificant – the effect size being significant is a feature of the construction process.

The two studies evaluating the New York City Summer Youth Employment Program, Gelber et al. (2016) and Valentine et al. (2017), both find positive and significant impacts on entry to employment. However, both also find that this is entirely a result of the design of the summer employment programme; the New York City Summer Youth Employment Program allows individuals to reapply in subsequent years, and individuals in the treatment group are more likely to reapply than individuals in the control group, and their summer employment programme is the employment picked up in the analysis. Both studies find that allocation to or participation in the summer programme has a negative impact on the likelihood of being employed outside of the summer programme. Davis and Heller (2020) also finds that participation in the summer employment programme has a negative (although in this case insignificant) effect on the likelihood of being employed by a non‐provider employer post‐programme. Using the impact estimates for employment outside of the summer employment programme for Gelber et al. (2016), Valentine et al. (2017) and Davis and Heller (2020) decreases the average effect size which is now significant (log odds ratio = −0.23, 95% confidence interval = −0.42, −0.03), suggesting that participation in a summer employment programme may decrease the likelihood of being employed outside of summer employment programme in the short term. Note that there is potential for a mismatch in the outcome measures between these studies where we are able to strip out future employment relating to the summer employment programme and the results from Modestino and Paulsen (2019a) and Theodos et al. (2017) where this is not possible.

The *p*‐value from the homogeneity test (*p* = 0.00) indicates that there is evidence of between‐study heterogeneity, reducing the applicability of the average effect size to all summer employment programmes. There were no statistically significant differences in impact across the sub‐groups of interest. None of the interventions included in this analysis targeted individuals from ethnic minorities.

Removing the result for the New York City Summer Youth Employment Program from Gelber et al. (2016) and Valentine et al. (2017), as this is the highest weighted intervention, decreases the average effect size to −0.29 which becomes marginally significant (95% confidence interval = −0.57, −0.01). Additionally, removing the results from Valentine et al. (2017) and Modestino and Paulsen (2019a) which report impacts of allocation to a summer programme that are then transformed to impacts of participation has a negligible effect on the findings.

**Figure 21:**
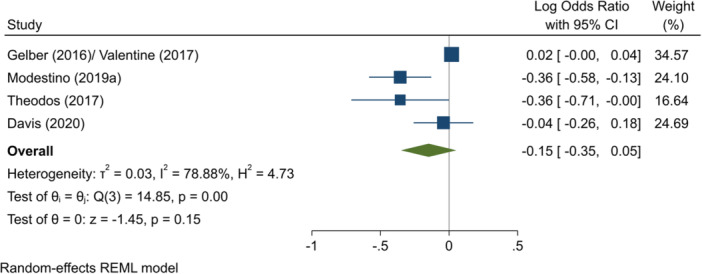
Overall impact of summer employment programme participation on likelihood of entering employment. *Source*: IES (2024).

Figure 21 displays the forest plot from the meta‐analysis of the impact of participation in a summer programme on the likelihood of entering employment, pooling the results over all time points together for Gelber et al. (2016), Valentine et al. (2017) and Theodos et al. (2017). Again, as all these interventions are summer employment programmes there is no split by programme type.

The story is largely the same as when looking at impacts over the shorter term – summer employment programmes have a negative although insignificant average effect size (log odds ratio = −0.15, 95% confidence interval = −0.35, 0.05), suggesting that participation in a summer employment programme does not have a significant impact on the overall likelihood of entering employment. As before, using instead the impact estimates for employment outside of the summer employment programme for Gelber et al. (2016), Valentine et al. (2017) and Davis and Heller (2020) decreases the average effect size although in this instance it remains marginally insignificant (log odds ratio = −0.18, 95% confidence interval = −0.35, 0.00).

As before, the *p*‐value from the homogeneity test (*p* = 0.00) indicates that there is evidence of between‐study heterogeneity, reducing the applicability of the average effect size to all summer employment programmes. There were no statistically significant differences in impact across the sub‐groups of interest. None of the interventions included in this analysis targeted individuals from ethnic minorities.

Again, removing the result from the New York City Summer Youth Employment Program studied by Gelber et al. (2016) and Valentine et al. (2017) decreases the average effect size to −0.23 and turns it significant (95% confidence interval = −0.46, −0.01). Additionally, removing the results from Valentine et al. (2017) and Modestino and Paulsen (2019a) has a negligible impact on the findings.

Overall, it appears then that summer employment programmes have a limited impact on entry to employment, and if anything, have a significant negative impact on entry to employment outside of extended employment relating to the summer employment programme itself. This is also interesting to note in combination with the positive although insignificant effect on progression to higher education found by Theodos et al. (2017) and the positive impact on intentions to progress to higher education found by Modestino and Paulsen (2019a). Indeed, Theodos et al. (2017) suggests that given the high rate of successive college attendance among programme participants, positive labour market outcomes are likely to develop over a longer timespan than examined in their evaluation. Modestino and Paulsen (2019a and, 2019b) also puts forward as explanations for these findings that: early work experience can raise career and academic aspirations through greater exposure to employment, which provides experiences (e.g., new occupations, different adult mentors, wider networks) that can help young people shape their goals; and that by enabling participants to shift their work experiences to a part of the year when they are not also attending school, summer employment programmes might enable young people to increase the time and attention that they can devote to academics during the school year and reduce their need to work in the post‐programme period.

#### 14.3.2 Earnings

▪ Studies evaluating summer employment programmes find a mix of no impact and negative impacts on this outcome.
▪ The two studies that find negative impacts on earnings find that this is the case conditional on being in employment.

Four studies across three interventions report impacts of allocation to or participation in a summer programme on earnings:

▪ Urban Alliance:
  - Theodos et al. (2017) – average wages and money accumulated; 1 and 2 years after the summer programme.
▪ One Summer Chicago:
  - Davis and Heller (2020) – quarterly earnings; first year and a half after the programme.
▪ New York City Summer Youth Employment Program:
  - Valentine et al. (2017) – annual earnings; first, second, third, fourth and fifth year after the summer programme.
  - Gelber et al. (2016) – annual earnings; first, second, third and fourth year after the summer programme.

Theodos et al. (2017) finds no significant impact of allocation to the summer employment programme on average wages or money accumulated 1 or 2 years after the programme.

Davis and Heller (2020) also finds no significant impact of participation in the summer employment programme on earnings in the first year and a half after the programme.

Valentine et al. (2017) finds a statistically significant negative impact of allocation to the summer employment programme on earnings in the first and second years after the programme, equal to a $40 decrease in total earnings (or an approximately 3% decrease 1 year after the programme) and equating to an effect size (SMD) of −0.01 (95% confidence interval = −0.02, −0.00) in both years, although there is no significant impact in the third, fourth or fifth years post‐programme. Furthermore, in combination with the impacts on entry to employment that Valentine et al. (2017) estimates, this suggests that allocation to the summer employment programme sees individuals earn less conditional on being in employment.

Gelber et al. (2016) finds a statistically significant negative impact of allocation to the summer employment programme on earnings in the first, second and third years post‐programme, equal to decreases of $73.11, $68.66 and $81.04 respectively (or an approximately 2% decrease in each year), and equating to effect sizes (SMD) of −0.01 (95% confidence interval = −0.02, 0.00), −0.01 (95% confidence interval = −0.01, 0.00) and −0.01 (95% confidence interval = −0.01, 0.00) respectively, although there is no significant impact in the fourth year post‐programme. Furthermore, in combination with the impacts on entry to employment that Gelber et al. (2016) estimates, this suggests that allocation in the summer employment programme sees individuals earn less conditional on being in employment.

#### 14.3.3 Job readiness

*Summary*:

▪ Studies evaluating summer employment programmes find a mix of no impact and positive impacts on this outcome.

Three studies across three interventions report impacts of allocation to or participation in a summer programme on job readiness measures:

▪ Boston Summer Youth Employment Program:
  - Modestino and Paulsen (2019a) – proportion performing a range of job readiness behaviours; end of the summer programme.
▪ Higher Achievement:
  - Herrera et al. (2013) – proportion that have visited a business to see what it would be like to work there; spring of the first, second and fourth year after the summer programme.
▪ Urban Alliance:
  - Theodos et al. (2017) – comfort with soft and hard skills; at 1 and 2 years after the summer programme.

Note that each of these studies source their outcome measures from surveys with highly differential response rates between the treatment and control groups, introducing a potential source of bias into the estimates produced.

Modestino and Paulsen (2019a) finds that allocation to the summer employment programme has a significant positive impact on five of the nine different job readiness measures examined, and no significant impact on the other four. When pooled together, these impacts equate to an overall effect size (log odds ratio) of 0.41 (95% confidence interval = 0.12, 0.71) and an odds ratio of 1.51. So, individuals allocated to the summer employment programme are 1.51 times more likely to report performing a range of job readiness behaviours than those not doing so at the end of the summer employment programme. This is an interesting finding especially when considering the same study, as previously detailed, found that allocation to the summer programme had negative impacts on intentions to enter employment. Within the job readiness measures that Modestino and Paulsen (2019a) evaluates, the least positive impact estimates are for those behaviours most directly measuring job search activity such as completing at least one online job application, asking an adult to serve as a reference, and searching for jobs online – allocation to the summer employment programme has no significant impact on any of these.

It is also worth noting the generally positive impact Modestino and Paulsen (2019a) finds on job readiness in combination with the significant negative impact they find on short‐term work intentions. They suggest that whilst young people may gain job readiness skills through participation in the summer employment programme, because they are in school during the following year these short‐term outcomes may not translate into longer‐term improvements related to employment if they choose not to work while in school, especially during the short evaluation window considered.

Herrera et al. (2013) finds that allocation to the summer education programme has no significant impact on the proportion of individuals who had visited a business to see what it would be like to work there in the first year after the programme. Two and 4 years after the programme, however, they do find significant positive impacts, equating to effect sizes of 0.32 (95% confidence interval = 0.07, 0.59) and 0.61 (95% confidence interval = 0.34, 0.87) respectively, and odds ratios of 1.39 and 1.83 respectively. So, individuals allocated to the summer education programme are 1.39 and 1.83 times more likely have visited a business to see what it would be like to work there 2 and 4 years post‐programme respectively. The intervention Herrera et al. (2013) evaluates lasts across 4 years which likely explains some of the increase in impact over time.

Theodos et al. (2017) finds positive impacts on the soft skills index from allocation to the summer programme after 1 year (SMD = 0.22, 95% confidence interval = 0.04, 0.41) although there is no significant impact after 2 years. Impact was detected from participation in the summer programme after 1 year (SMD = 0.44, 95% confidence interval = 0.26, 0.63) and after 2 years (SMD = 0.31, 95% confidence interval = 0.12, 0.49). Theodos et al. (2017) finds a significant impact of participation on hard skills after 1 year (SMD = 0.29, 95% confidence interval = 0.11, 0.48), apart from this they find no significant impact from allocation after 1 or 2 years, or from participation after 2 years. The specific outcome measure used by Theodos et al. (2017) means that the effect sizes provide the easiest interpretation of the impacts, therefore we do not translate them into another format.

### 14.4 Violence and offending

#### 14.4.1 Whether had a criminal justice outcome (including arrests, arraignments, convictions or incarcerations)

*Summary*:

▪ Findings for the impact of summer employment programmes on this outcome (including when broken down by the type of violent/offending behaviour) are mixed – studies largely either find no impact or substantial negative (i.e., beneficial) impacts.

Three studies across two interventions report impacts of allocation to or participation in a summer programme on whether an individual had an arrest, arraignment, conviction or incarceration for any type of crime or offence. These are:

▪ New York City Summer Youth Employment Program:
  - Kessler et al. (2022) – whether had an arrest/inc conviction; during programme months and 1, 3 and 5 years after the summer programme;
  - Gelber et al. (2016) – whether had an incarceration; across 4 years after the summer programme;
▪ Boston Summer Youth Employment Program:
  - Modestino (2019b) – whether had an arraignment; across 17 months after the summer programme.

Kessler et al. (2022) finds that participation in the summer employment programme has a significant negative (i.e., a beneficial) impact on the probability that an individual has an arrest during the programme months, which translates to an effect size (log odds ratio) of −0.19 (95% confidence interval = −0.31, −0.07) and an odds ratio of 0.83 (95% confidence interval = 0.74, 0.93). So, participants in the summer employment programme were 0.83 times as likely to have an arrest as those that did not participate during the programme months, although there are no significant impacts post‐programme. During the programme months, Kessler et al. (2022) finds a significant negative (i.e., a beneficial) impact on the probability of having a conviction during the programme months, which translates to an effect size (log odds ratio) of −0.38 (95% confidence interval = −0.60, −0.16) and an odds ratio of 0.68 (95% confidence interval = 0.55, 0.85). So, participants in the summer employment programme were 0.68 times as likely to have a conviction as those that did not participate during the programme months. Again, Kessler et al. (2022) did not find any significant impacts on this outcome post‐programme.

Gelber et al. (2016) finds that participation in the summer employment programme has a significant negative (i.e., a beneficial) impact on the probability that an individual has an incarceration across the 4 years post‐programme, which translates to an effect size (log odds ratio) of −0.11 (95% confidence interval = −0.18, −0.03), and an odds ratio of 0.90 (95% confidence interval = 0.83, 0.97). This means that participants in the summer employment programme are 0.90 times as likely to be incarcerated as those that did not participate across the 4 years post‐programme.

Modestino (2019b) finds that allocation to the summer employment programme has no significant impact on the probability that an individual has an arraignment.

At the request of the review advisory group, given the particular interest of the organisations involved in the review (including YEF) in this domain, the requirement of studies covering four separate interventions to evaluate an outcome to perform meta‐analysis was dropped to two for this and the subsequent violence and offending outcomes examined. The rule was introduced to manage the size of the review (i.e., not for methodological purposes, as it is possible to perform meta‐analysis with just two effect sizes) given the large number of individual outcomes examined by a small number of studies across the range of domains of interest.

**Figure 22:**
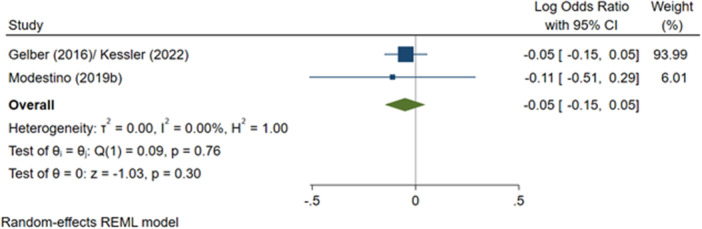
Impact of summer employment programme participation on likelihood of having an arrest/arraignment/conviction/incarceration post‐programme. *Source*: IES (2024).

The effect sizes used were based on the impact estimates from the longest follow up point, that is, 5 years (60 months) post‐programme for Kessler et al. (2022), as only one study (Kessler et al., 2022) produces usable estimates of impact during the programme months. Figure 22 displays the forest plot from the meta‐analysis of the impact of participation in a summer programme on the likelihood that an individual had an arrest, arraignment, conviction or incarceration for any crime or offence across approximately 17–60 months post‐programme. As all the interventions included in this analysis are summer employment programmes, no split is included by programme type.

The average effect size for summer employment programmes is negative although insignificant (log odds ratio = −0.05, 95% confidence interval = −0.15, 0.05), suggesting that they do not appear to have an impact on the likelihood of an individual having an arrest/arraignment/conviction/incarceration post‐programme.

The *p*‐value from the homogeneity test (*p* = 0.76) indicates that there is no evidence of between‐study heterogeneity. None of the interventions included in this analysis targeted individuals from ethnic minorities.

Additionally, three studies across three interventions report impacts of allocation to or participation in a summer programme on whether an individual engaged specifically in drug‐related, violent, or property‐related criminal or offending activity. These are:

▪ Youth Violence Prevention Funder Learning Collaborative summer employment programme:
  - Sum (2015):
    – change in the proportion of individuals that bought or sold illegal drugs, or used marijuana; from start to end of summer programme;
    – change in the proportion of individuals that attacked or threatened someone with a weapon other than a gun; from start to end of the summer in which the summer programme took place; and
    – change in the proportion of individuals that damaged or destroyed someone's property; from start to end of summer programme.
▪ New York City Summer Youth Employment Program:
  - Kessler et al. (2022) – whether had an arrest/inc conviction for drug/violent/property crimes/offences; during programme months and 1, 3 and 5 years after the summer programme.
▪ Boston Summer Youth Employment Program:
  - Modestino (2019b) – whether had an arraignment for drug/violent/property crimes/offences; across 17 months after the summer programme.

Note that the outcomes evaluated by Sum (2015) are self‐reported measures based on surveys, and limited information is provided about the extent of attrition (overall and differential rates), potentially introducing a source of bias into the estimates produced.

Sum (2015) finds that participation in the summer employment programme had no significant impact on: the proportion of individuals that went from reporting that they bought or sold illegal drugs, to not doing so; the proportion of individuals who went from reporting that they had attacked or threatened someone with a weapon other than a gun, to not doing so; the proportion of individuals initially reporting that they had damaged or destroyed someone's property, to not doing so; and the proportion of individuals that went from reporting that they did not use marijuana, to doing so. Sum (2015) does, however, find a significant positive impact on the proportion of individuals that went from reporting that they use marijuana to not doing so, equal to an 8‐percentage point impact on the likelihood of seeing this improvement (insufficient information is available to construct an effect size). It should be noted though that Sum (2015) has a low‐quality study design and limited information on the construction of outcomes or the estimation approach used, therefore one should be cautious about the findings for this or any of the other violence and offending outcomes that Sum (2015) evaluates.

Kessler et al. (2022) finds that participation in the summer employment programme has a significant negative (i.e., beneficial) impact on the probability that an individual has a violent crime or offence conviction during the programme months, which translates to an effect size (log odds ratio) of −1.02 (95% confidence interval = −1.54, −0.51) and an odds ratio of 0.36 (95% confidence interval = 0.21, 0.61). This means that individuals that participate in the summer employment programme are 0.36 times as likely to have a violent crime or offence conviction as those that do not during the programme months. However, after 1, 3 and 5 years post‐programme there is no significant impact of participation in the summer employment programme on this outcome. Additionally, Kessler et al. (2022) estimates no significant effect of summer employment programme participation on the probability that an individual has a violent crime or offence arrest.

Kessler et al. (2022) finds that participation in the summer employment programme has a significant negative (i.e., a beneficial) impact on the probability that an individual has a property crime or offence arrest during the programme months and after 1 year post‐programme, which translate to effect sizes (log odds ratios) of −0.84 (95% confidence interval = −1.23, −0.46) and −0.28 (95% confidence interval = −0.42, −0.14) respectively. These translate to odds ratios of 0.43 (95% confidence interval = 0.29, 0.63) and 0.76 (95% confidence interval = 0.66, 0.87) respectively. This means that individuals who participate in the summer employment programme are 0.43 times and 0.76 times as likely to have a property crime or offence arrest as those that do not during the programme months and 1 year post‐programme respectively. However, after 3 and 5 years post‐programme there is no significant impact of participation in the summer employment programme on the probability that an individual has a property crime or offence arrest, and across all periods considered there is no significant impact on the probability that an individual has a property crime or offence conviction.

Kessler et al. (2022) finds that participation in the summer employment programme has no significant impact on the probability that an individual has a drug crime/offence arrest or conviction during the programme months and 1 year post‐programme. However, they find that participation in the summer employment programme has a significant negative (i.e., a beneficial) impact on the probability that an individual has a drug crime or offence arrest after 3 and 5 years post‐programme, and on the probability that an individual has a drug crime or offence conviction 5 years post‐programme. The impacts on the probability that an individual has a drug crime or offence arrest 3 and 5 years post‐programme translate to effect sizes (log odds ratios) of −0.10 (95% confidence interval = −0.15, −0.04) and −0.06 (95% confidence interval = −0.11, −0.02) respectively. These translate to odds ratios of 0.91 (95% confidence interval = 0.86, 0.96) and 0.94 (95% confidence interval = 0.90, 0.98) respectively. This means that individuals who participate in the summer employment programme are 0.91 and 0.94 times as likely to have a drug crime or offence arrest as those that do not after 3 and 5 years post‐programme respectively. The impact on the probability that an individual has a drug crime or offence conviction 5 years post‐programme translates to an effect size (log odds ratio) of −0.16 (95% confidence interval = −0.27, −0.06). This translates to an odds ratio of 0.85 (95% confidence interval = 0.77, 0.94), which means that individuals who participate in the summer employment programme are 0.85 times as likely to have a drug crime or offence conviction as those that do not 5 years post‐programme.

Modestino (2019b) finds that allocation to the summer employment programme has no significant impact on the probability that an individual has an arraignment for violent, property or drug crimes/offences.

Supporting Information: Appendix 3 contains the results from meta‐analyses of the impact of participation in the summer employment programmes studied by Kessler et al. (2022) and Modestino (2019b) on the likelihood that an individual had an arrest, arraignment or conviction for violent, drug or property crimes or offences across approximately 17–60 months post‐programme.

Again, as only Kessler et al. (2022) produces usable estimates of impact during the programme months, we used effect sizes based on the impact estimates from the longest follow‐up point, that is, 5 years post‐programme for Kessler et al. (2022). The average effect sizes estimated for each of the three analyses are insignificant, suggesting that participation in a summer employment programme has no impact on the likelihood that an individual had an arrest, arraignment or conviction for violent, drug or property crimes or offences post‐programme. The *p*‐values from the homogeneity tests indicate statistically significant heterogeneity for the analysis of drug crimes/offences (*p* = 0.09), although not for violent or property crimes/offences. None of the interventions included in these analyses targeted individuals from ethnic minorities.

#### 14.4.2 Number of criminal justice outcomes (including arrests, arraignments, convictions or incarcerations)

*Summary*:

▪ Findings for the impact of summer employment programmes on this outcome (including when broken down by the type of violent/offending behaviour) are mixed – studies largely either find no impact or substantial negative (i.e., beneficial) impacts.

Five studies across three interventions report impacts of allocation to or participation in a summer programme on the number of arrests, arraignments, convictions or incarcerations an individual has for any type of crime or offence. These are:

▪ New York City Summer Youth Employment Program:
  - Gelber et al. (2016) – number of incarcerations; across 4 years after the summer programme.
  - Kessler et al. (2022) – number of arrests/convictions; during programme months and 1, 3 and 5 years after the summer programme.
▪ Boston Summer Youth Employment Program:
  - Modestino (2019b) – number of arraignments; across 17 months after the programme.
▪ One Summer Chicago:
  - Davis and Heller (2020) – number of arrests; first, second and third year post‐randomisation.
  - Heller (2022) – number of arrests; first, second and third year post‐randomisation.

Gelber et al. (2016) finds that participation in the summer employment programme has a significant negative (i.e., a beneficial) impact on the number of incarcerations that an individual has, which equals a 11% reduction in the number compared with the control group mean, and translates to an effect size (SMD) of −0.01 (95% confidence interval = −0.02, −0.00). Note that the magnitude of reduction reported here differs from the headline figure reported by Gelber et al. (2016) themselves, and this is also the case for several other instances of impact discussed in this section. This is due to differences in the results produced by different specifications of the models used by the study authors, who tend to take as their preferred results to report on those from the approaches that do not control for differences in baseline characteristics between the treatment and comparison groups. Conversely, where there is the option we choose to use those that do control for differences in baseline characteristics.

Kessler et al. (2022) finds that participation in the summer employment programme has no significant impact on the number of times the young person is arrested. Kessler et al. (2022) finds a significant, negative (i.e., a beneficial) impact on the number of convictions an individual has during the programme summer equal to a 28% reduction in the number compared with the control group mean and equating to an effect size (SMD) of −0.01 (95% confidence interval = −0.02, −0.00). However, in the years after the programme summer there is no significant impact.

Modestino (2019b) finds that allocation to the summer employment programme does not have a significant impact on the number of arraignments recorded for young people.

Davis and Heller (2020) finds that allocation to the summer employment programme has no significant impact on the number of arrests an individual has.

Heller (2022) finds that allocation to the summer employment programme has no significant impact on the number of arrests an individual has.

Firstly, we perform meta‐analysis using effect sizes from the period covering the programme months, that is, for Kessler et al. (2022) from during the programme months and for Davis and Heller (2020) and Heller (2022) from the first year post‐randomisation. Note that the results from Davis and Heller (2020) and Heller (2022) cover both during and post‐programme periods.

**Figure 23:**
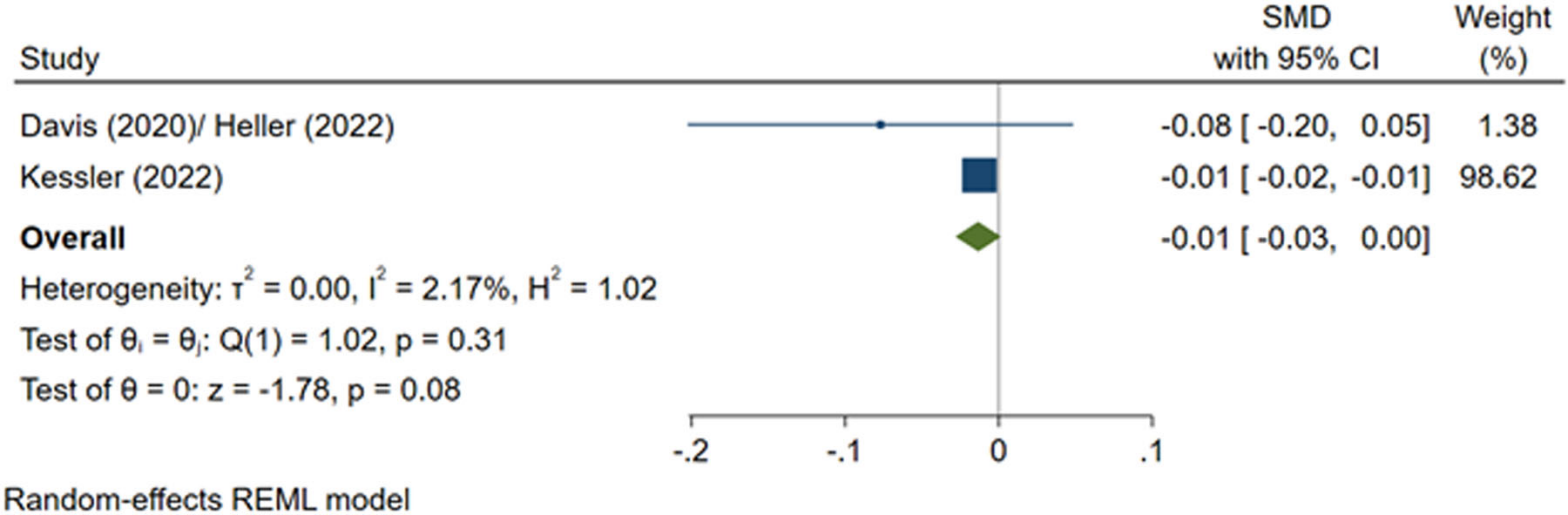
Impact of summer employment programme participation on number of arrests/convictions approx. during programme months. *Source*: IES (2024).

Figure 23 displays the forest plot from the meta‐analysis of the impact of participation in a summer programme on the number of arrests/convictions across a period that approximately corresponds to the period of the summer programme. As all the interventions included in this analysis are summer employment programmes, no split is included by programme type.

Overall, summer employment programmes do not appear to have a significant impact on the number of arrests/arraignments/convictions/incarcerations an individual has (average effect size (SMD) = −0.01, 95% confidence interval = −0.03, 0.00).

The *p*‐value from the homogeneity test (*p* = 0.31) indicates that there is no evidence of between‐study heterogeneity. None of the interventions included in this analysis targeted individuals from ethnic minorities.

Secondly, we perform meta‐analysis using effect sizes based on the impact estimates from the longest follow up point, that is, 5 years (60 months) post‐programme for Kessler et al. (2022) and 3 years post‐randomisation from Davis and Heller (2020) and Heller (2022).

**Figure 24:**
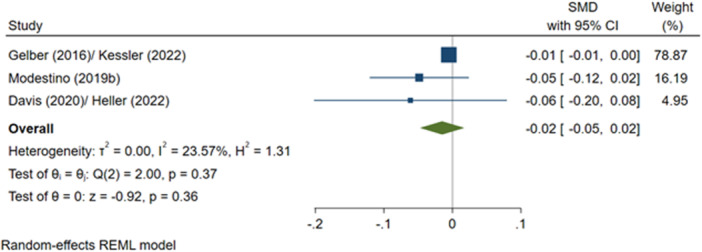
Impact of summer employment programme participation on number of arrests/arraignments/convictions/incarcerations post‐programme. *Source*: IES (2024).

Figure 24 displays the forest plot from the meta‐analysis of the impact of participation in a summer programme on the number of arrests/arraignment/convictions/incarcerations covering approximately 17–60 months post‐programme. As all the interventions included in this analysis are summer employment programmes, no split is included by programme type.

Overall, summer employment programmes do not appear to have a significant impact on the number of arrests/arraignments/convictions/incarcerations an individual has (average effect size (SMD) = −0.02, 95% confidence interval = −0.05, 0.02).

The *p*‐value from the homogeneity test (*p* = 0.37) indicates that there is no evidence of between‐study heterogeneity. None of the interventions included in this analysis targeted individuals from ethnic minorities.

Additionally, four studies across two interventions report impacts of allocation to or participation in a summer programme on the number of arrests, arraignments or convictions an individual has specifically for drug‐related, violent, or property‐related criminal or offending activity. These are:

▪ Boston Summer Youth Employment Program:
  - Modestino (2019b) – number of arraignments for drug/violent/property crimes or offences; across 17 months after the summer programme.
▪ One Summer Chicago:
  - Davis and Heller (2020) – number of arrests for drug/violent/property crimes or offences; first, second and third year post‐randomisation.
  - Heller (2014) – number of arrests for drug/violent/property crimes or offences; across 13 months after the summer programme.
  - Heller (2022) – number of arrests for drug/violent/property crimes or offences; first, second and third year post‐randomisation.

Modestino (2019b) finds that allocation to the summer employment programme has no significant impact on the number of arraignments for drug crimes an individual has. However, they find that allocation to the summer employment programme leads to a significant reduction in the number of arraignments for violent crimes that an individual had, equal to a 52% reduction in the number compared with the control group mean and equating to an effect size (SMD) of −0.06 (95% confidence interval = −0.13, 0.01 – the raw impact estimate from Modestino (2019b) is significant at the 95% confidence level, but as a result of the assumptions used in order the construct an effect size from the reported data, the SMD is marginally insignificant at the 95% confidence level). They also find that allocation to the summer employment programme leads to a significant reduction in the number of arraignments for property crimes an individual has, equal to a 46% reduction in the number compared with the control group mean and equating to an effect size (SMD) of −0.06 (95% confidence interval = −0.13, 0.00 – the raw impact estimate from Modestino (2019b) is significant at the 95% confidence level, but as a result of the assumptions used to construct an effect size from these data, the SMD is marginally insignificant at the 95% confidence level).

Davis and Heller (2020) finds that allocation to the summer employment programme has no significant impact on the number of arrests for drug crimes an individual has. Davis and Heller (2020) finds that allocation to the summer employment programme has a significant negative (i.e., a beneficial) impact on the number of arrests for violent crimes an individual has in the first year post‐randomisation. This impact is equal to a 41% reduction in the number compared with the control group mean and equates to an effect size (SMD) of −0.06 (95% confidence interval = −0.15, 0.04: note that the specifics of the process for constructing the effect size, which in this instance requires imputation of the standard deviation from another study, means that the constructed effect size is insignificant). However, they find no significant impacts on this in the second and third years post‐randomisation. Davis and Heller (2020) finds that allocation to the summer employment programme has no significant impact on the number of arrests for property crimes an individual has in the first year post‐randomisation, but a significant positive (i.e., an increase in the number) impact in the second and third years post‐randomisation, equal to a 95% and 93% increase in the number compared with the control group mean respectively and translating to effect sizes (SMD) of 0.18 (95% confidence interval = 0.09, 0.28) and 0.19 (0.09, 0.28) respectively.

Heller (2014) finds that allocation to the summer employment programme has no significant impact on the number of arrests for drug crimes or property crimes an individual has. However, they find that allocation to the summer employment programme has a significant negative (i.e., a beneficial) impact on the number of arrests for violent crimes an individual has, equal to a 43% reduction in the number compared with the control group mean and equating to an effect size (SMD) of −0.07 (95% confidence interval = −0.16, 0.03: note that the specifics of the process for constructing the effect size, which in this instance requires imputation of the standard deviation from another study, means that the constructed effect size is insignificant).

Heller (2022) finds that allocation to the summer employment programme has a significant negative (i.e., a beneficial) impact on the number of arrests for drug crimes or offences that an individual has in the first year post‐randomisation, equal to a 40% reduction in the number compared with the control group mean and equating to an effect size (SMD) of −0.04 (95% confidence interval = −0.09, 0.02: note that the specifics of the process for constructing the effect size, which in this instance requires imputation of the standard deviation from another study, means that the constructed effect size is insignificant). However, allocation to the summer employment programme has no significant impact on the number of arrests for drug crimes or offences an individual has in the second or third years post‐randomisation, or at all on the number of violent or property crimes or offences an individual has.

Supporting Information: Appendix 3 contains the results from meta‐analyses of the impact of participation in the summer employment programmes studied by Modestino (2019b) and Heller (2014), Davis and Heller (2020) and Heller (2022) on the number of arrests or arraignment for violent, drug or property crimes or offences an individual has across approximately 17–34 months post‐programme.

We used effect sizes based on the impact estimates from the longest follow‐up point, that is, 3 years post‐randomisation (approximately 34 months post‐programme) for Heller (2014), Davis and Heller (2020) and Heller (2022). The average effect sizes estimated for each of the three analyses are insignificant, suggesting that participation in a summer employment programme has no impact on the number of arrests or arraignments for violent, drug or property crimes or offences an individual has post‐programme. The *p*‐values from the homogeneity tests indicate statistically significant heterogeneity for the analysis of property crimes/offences (*p* = 0.09), although not for violent or drug crimes/offences. None of the interventions included in these analyses targeted individuals from ethnic minorities.

### 14.5 Socio‐emotional outcomes

#### 14.5.1 Socio‐emotional skills and engagement

*Summary*:

▪ The two studies of summer education programmes find positive impacts on this outcome.
▪ Among studies of summer employment programmes evaluating this outcome, one finds a positive impact whilst the other finds no impact.

Three studies across three interventions report impacts of allocation to a summer programme on socio‐emotional skills and engagement. These are:

▪ Boston Summer Youth Employment Program:
  - Modestino (2019b) – proportion demonstrating a range of behaviours; end of the summer programme.
▪ Urban Alliance:
  - Theodos et al. (2017) – goal setting index score; time management index score, 2 years after the summer programme.
▪ STEM summer programmes:
  - Cohodes et al. (2022) – life skills index score; spring after the summer programme.

Note that each of these studies source their self‐reported outcome measures from surveys. The extent of attrition (overall and differential rates) for the surveys used by Modestino (2019b) and Theodos et al. (2017) are highly concerning, introducing a potential source of bias into the estimates produced. Attrition is not a significant concern for the survey‐based measure from Cohodes et al. (2022).

Modestino (2019b) finds that allocation to the summer employment programme has a positive significant impact on socio‐emotional skills and engagement (SMD = 0.32, 95% confidence interval = 0.20, 0.45). Translating this back to the specific measures used by Modestino (2019b), this means that those allocated to the summer employment programme were (on average) 1.79 times more likely to report: feeling connected to people in their neighbourhood, knowing how to ask for help when they need it, knowing how to constructively resolve a conflict with a peer, knowing how to manage their emotions and their temper, and feeling that they have a lot to contribute to the groups they belong to; than those that were not.

Theodos et al. (2017) finds that allocation to the summer employment programme has no significant impact on socio‐emotional skills and engagement.

Cohodes et al. (2022) finds that allocation to the summer education programme had a positive significant impact on socio‐emotional skills and engagement (SMD = 0.23, 95% confidence interval = 0.07, 0.39). The specific outcome measure used by Cohodes et al. (2022) means that the effect size provides the easiest interpretation of the impacts, therefore we do not translate this into another format.

Two studies across two interventions report impacts of participation in a summer programme on socio‐emotional skills and engagement. The estimates of impact of allocation from Cohodes et al. (2022) cannot be translated to estimates of impact of participation in the summer programme, as they report standardised index scores with insufficient information to transform them, therefore there are insufficient studies evaluating this outcome to perform meta‐analysis. In addition to Theodos et al. (2017), the other is:

▪ Department for Education Summer Schools Programme:
  - Martin (2013b) – socialisation index score; autumn after the summer programme.

Note that Martin et al. (2013b) sources their self‐reported outcome measures from a survey. Insufficient information is provided to assess the potential for differential response rates between students in the treatment and comparison groups to introduce bias into the estimate produced.

Theodos et al. (2017) finds that participation in the summer employment programme has no significant impact on socio‐emotional skills and engagement.

Martin et al. (2013b) finds that participation in the summer education programme has a positive significant impact on socio‐emotional skills and engagement (SMD = 0.10, 95% confidence interval = 0.07, 0.12). An SMD of 0.10 translates to approximately 10% of students seeing a 1 level improvement (with each item scored from 1 to 5) in their response to each of the five items relating to socialisation (I made friends quickly when I started this school; I am bullied/picked on by people from my school; I feel safe in school; other people listen to what I say; I often feel left out).

#### 14.5.2 Community engagement

*Summary*:

▪ The one study of a summer education programme evaluating this outcome finds no impact. The one study of a summer employment programme evaluating this outcome finds a positive impact.

Two studies across two interventions report impacts of allocation to a summer programme on measures of community engagement. These are:

▪ Boston Summer Youth Employment Program:
  - Modestino and Paulsen (2019a) – sense of community index; end of the programme summer.
▪ Higher Achievement:
  - Herrera et al. (2013) – likelihood of engaging in community service and/or voluntary work; first, second and fourth years after the summer programme.

Note that each of these studies source their outcome measures from surveys with highly differential response rates between the treatment and control groups, introducing a potential source of bias into the estimates produced.

Modestino and Paulsen (2019a) finds that allocation to the summer employment programme has a significant positive impact on 5 of the 12 different sense of community measures examined, and no significant impact on the other seven. When pooled together, these impacts equate to an overall effect size (log odds ratio) of 0.26 (95% confidence interval = 0.12, 0.40) and an odds ratio of 1.30 (95% confidence interval = 1.13, 1.49). So, individuals allocated to the summer employment programme are 1.30 times more likely to respond ‘true’ to the range of statements relating to sense of community measured than those who are not, at the end of the summer employment programme.

Herrera et al. (2013) find no significant impact on the likelihood of engaging in community service and/or voluntary work as a result of allocation to the summer education programme.

### 14.6 Health outcomes

*Summary*:

▪ The one study of a summer employment programme that measures mental health finds a positive impact. The one study of a summer employment programme that measures mortality rates finds a negative (i.e., beneficial) impact.

Whilst the two outcomes within the health domain are only evaluated by one study, each of which would usually not be sufficient for them to be examined within the review, given the importance to this review of examining the wider impacts of summer programmes on disadvantaged or ‘at risk’ young people they are summarised narratively, and, in the summary of findings, are treated together.

#### 14.6.1 Mental health

One study reports impacts of allocation to a summer programme on mental health:

▪ Boston Summer Youth Employment Program:
  - Modestino and Paulsen (2019a) – likelihood of reporting ‘hardly ever’ to a range of statements used to diagnose and assess the severity of depression; end of the programme summer.

Note that Modestino and Paulsen (2019a) sources their outcome measures from a survey with highly differential response rates between the treatment and control groups, introducing a potential source of bias into the estimates produced.

Modestino and Paulsen (2019a) finds that allocation to the summer employment programme has a significant positive impact on all five of the depression scale measures examined. When pooled together, these impacts equate to an overall effect size (log odds ratio) of 0.43 (95% confidence interval = 0.31, 0.56) and an odds ratio of 1.54 (95% confidence interval = 1.36, 1.75), that is, individuals allocated to the summer employment programme are 1.54 times as likely to report hardly ever experiencing symptoms of depression, than those who are not. This is corroborated by the estimated significant negative impact on an individual's depression index based on these questions (insufficient information was available to construct an effect size for this measure).

#### 14.6.2 Mortality rates

One study reports impacts of participation in a summer programme on mortality rates:

▪ New York City Summer Youth Employment Program:
  - Gelber et al. (2016) – mortality rates; 4 years after the programme.

Gelber et al. (2016) finds that participation in the summer employment programme has a significant negative (i.e., beneficial) impact on mortality rates, equal to a 18% reduction in the rate number compared with the control group mean and equating to an effect size (log odds ratio) of −0.20 (95% confidence interval = −0.32, 0.08) and an odds ratio of 0.82 (95% confidence interval = 0.73, 0.93). This means that young people who participate in the summer programme are 0.82 times as likely to die as those who do not, post‐programme.

## 15 COST OF DELIVERING SUMMER PROGRAMMES

Sixteen studies covering 14 different summer programmes reported data on the cost per individual per year of delivering the summer programme. The form of this data varies considerably by study. Barnett et al. (2012) and the studies funded by the Education Endowment Foundation (Gorard et al., 2014; Maxwell et al., 2014; Torgerson et al., 2014) use figures from a formal cost analysis. The figure from Day et al. (2013a) is based on the results from the survey of schools they conducted. Pyne et al. (2020) source their figure from the programme organisation's tax return. Theodos et al. (2014) sources their figure from audited financial records and service delivery records. Snipes et al. (2015) uses the figure estimated by the foundation that designed the programme. Schwartz et al. (2021) performs a rough calculation of potential costs. Given that the (same) cost figure from Gelber et al. (2016) and Kessler et al. (2022), evaluating the same summer employment programme, is based on actual expenditure on the programme (even though the information on the source for this is limited), we use the figure from these studies for the programme instead. There is limited information on the source of the figures reported by Davis and Heller (2020), Herrera et al. (2013), Cohodes et al. (2022), Wachen et al. (2018), and Modestino (2019b). Barnett et al. (2012), Wachen et al. (2018), Davis and Heller (2020), Modestino (2019b) also perform some form of cost–benefit analysis with varying degrees of formality, which we do not report on. The differences in the sources used, as well as the assumptions or approaches taken to derive the cost figures, such as which individuals are counted towards the denominator, affects the generalisability of the cost per individual figure. Furthermore, wide differences between summer programmes and summer programme types, including in the duration, intensity, activities and resources used, limits our ability to provide any *representative* cost figure or list of ingredients needed to deliver a summer programme.

Cost data comes from interventions from both the UK and USA, and from a range of years. To standardise the figures, we inflate the cost figures to 2022 prices (i.e., the end of the period covered by the review) using the historical annual CPI inflation rates from the UK and US (ONS, 2024; US Inflation Calculator, 2024), and then for figures in dollars we convert them to pounds using the average exchange rate for 2022 (IRS, 2024).

Table 11 reports the cost per person of delivering the summer programmes for which cost data was provided, in pounds in 2022 prices. Where summer programmes run over multiple years, annual cost per person figures are used. Where a breakdown in the costs by source is reported, these are listed. We also note relevant assumptions the studies make in producing the figure, as well as instances where we ascribe the cost figure for a multi‐year programme/multi‐cohort study to a certain year for the purposes of inflating it to 2022 prices.

**Table 11:** Cost per person to deliver a summer programme.

Intervention	Source	Cost per person	Breakdown	Notes
*Summer education programmes*				
Department for Education Summer Schools Programme (UK)	Day et al. (2013a)	£370	−	For a two‐week programme, which is the modal duration
Discover Summer School (UK)	Torgerson et al. (2014)	£2197	35% venue hire, food and travel, 43% direct salary costs of staff, 5% promotion and contingency, 16% management and overheads	Assumes 125 pupils on a single site
Future Foundations summer school programme (UK)	Gorard et al. (2014)	£1681	26% administration, resources and activities, 61% staff salaries and training, 14% food and transport	Based on 160 pupils attending a school on a single site
Summer Active Reading Programme (UK)	Maxwell et al. (2014)	£178	–	Does not include volunteer/staff time. Assumes 60–90 pupils transitioning to one secondary school
Aim High (US)	Pyne et al. (2020)	£2612	–	
Elevate Math summer programme (US)	Snipes et al. (2015)	£501	–	Does not include cost of classroom and a site principal (provided by school) or laptop computers provided to every student (provided through a donation). Based on average class size of 30
Higher Achievement (US)	Herrera et al. (2013)	£4897	–	Assume figure is for 2010
STEM summer programmes (US)	Cohodes et al. (2022)	£2001 for the one‐week and online programmes, £15,009 for the six‐week programme	–	–
Texas developmental summer bridge programme (US)	Barnett et al. (2012)	£1458	32% staffing costs, 18% other costs, 27% student resources, 23% overheads	–
University of North Carolina summer bridge (US)	Wachen et al. (2018)	£3908	–	Assume figure is for 2011
*Summer employment programmes*				
Boston Summer Youth Employment Program (US)	Modestino (2019b)	£2001	70% participant wages, 30% administration	–
New York City Summer Youth Employment Program (US)	Gelber et al. (2016), Schwartz et al. (2021), Kessler et al. (2022)	£1428	74% participant wages, 26% administrative costs	Breakdown based on hours participants generally work from Schwartz et al. (2021) and average of hourly minimum wage from 2006 and 2007 from Gelber et al. (2016)
One Summer Chicago (US)	Davis and Heller (2020)	£3099	34% participant wages, 66% administrative costs	Includes only net wages paid to participants that they wouldn't have received had they been in employment outside the summer employment programme
Urban Alliance (US)	Theodos et al. (2014)	£5087 based on the number that attended some pre‐work training, £9157 based on those that completed the programme	46% participant wages and awards, 54% rent, staff wages and administrative costs	–

There is wide variation in the cost per person figures across the summer education programmes. Of the UK based interventions, the Department for Education Summer Schools Programme and Summer Active Reading Programme have relatively low costs per person. The former is designed and delivered through schools, and is largely focused on familiarising incoming pupils with the new education environment they are transitioning to, whilst the cost figure for the latter does not include volunteer or staff time, characteristics which may reduce the cost figures associated with delivering these programmes. Meanwhile, Discover Summer School and Future Foundations summer school programmes have far higher costs: these involve far higher staff costs, and the delivery of wide‐ranging academic workshops/support and enrichment activities.

Four (three entire interventions and an arm of one) of the summer educational programmes in the US have a higher cost per person than the Discover Summer School, the costliest UK‐based summer education programme. The 6‐week version of the STEM summer programmes studied by Cohodes et al. (2022) has the highest cost per person figure of any intervention that this is reported for. It is a residential programme that also includes a range of STEM‐related courses and visits to STEM‐focused companies. Higher Achievement has a substantial term‐time component (the *Afterschool Academy*) alongside the intense summer programme. The University of North Carolina summer bridge is also a residential programme with rigorous academic elements. Aim High combines project‐based learning with a socio‐emotional learning curriculum. Each of these programmes is also longer in duration (the summer education programme components are 5–6 weeks long) than the Discover Summer School (4 weeks). The Elevate Math summer programme, approximately a third as costly as the Texas developmental summer bridge programme which has the second lowest cost per person figure, makes use of free online mathematics instruction through the Khan Academy, and the laptops pupils use are provided through donation.

The summer employment programmes are more similar in terms of average cost per person than the summer education programmes. The Boston Summer Youth Employment Program cost per person figure is approximately 40% higher than that for New York City Summer Youth Employment Program: the maximum duration of the Boston programme is 6 weeks long compared with seven for the New York City programme, but the Boston programme contains a larger amount of training time. This is reflected in the share of the total cost going to participant wages being four percentage points higher for the New York City programme than the Boston programme. Urban Alliance, which has a much higher per person cost figure than the summer employment programmes, has far more substantive additional components alongside the job placement. When assessing the cost of providing a summer employment programme, a key consideration is which organisations (the summer employment programme provider or the employers) pay the participant wages associated with the job placement.

## 16 MECHANISMS TO IMPROVE SUMMER PROGRAMME ENGAGEMENT

This section explores mechanisms identified as increasing engagement in summer programmes – at both the extensive (i.e., whether an individual participates or not) and intensive (i.e., the intensity of an individual's participation) margins. We examine how these facilitate or act as barriers to engagement in summer programmes, through which the impacts on outcomes across the domains of interest resulting from summer programme participation are achieved. In our discussion of mechanisms, we include all studies in the review, both those that detect impact and those that do not. This is because lessons around how to increase engagement in a summer programme are independent of a study's ability to credibly detect an impact on outcomes. Though a range of studies in this review do not detect impact on outcomes, authors' suggestions based on the narrative and contextual evidence from the intervention still provide valuable insight. However, in reviewing this section, it should be noted that where studies do not detect impact, or where studies do not conduct an impact evaluation, we indicate this. Where studies do not detect impact, any discussion on mechanisms is based on study authors' considerations, and therefore confidence in the replicability of the findings is limited. We examine first those that are common to both summer education and employment programmes, before then discussing in turn those unique to summer education and summer employment programmes.

### 16.1 Summary of findings

There are a number of mechanisms which can act as facilitators or barriers to engagement in summer programmes. These include:

▪ **Personalisation**: summer education programmes tend to have lower student‐to‐staff ratios than ‘service as usual’. In summer employment programmes, providers place a strong focus on ensuring a suitable match between participants and work placements, to maximise participation.
▪ **Relationship with staff**: in summer education programmes, the presence of well‐prepared staff who provide effective academic and socio‐emotional support can create a positive learning environment which fosters participation. In summer employment programmes, the positive relationships with staff can support motivation and confidence, which in turn can improve attendance and programme completion.
▪ **Incentives**: for summer education programmes a range of incentives can support participation and engagement (e.g., free transport, no cost to participation, stipend), while in summer employment programmes the incentive is the opportunity to earn from the job placement, and gain work experience and employability skills.
▪ **Recruitment strategies:** in summer education programmes this includes school open evenings, providing written invitations, alumni advocates, and offering incentives (e.g., discount schemes). Summer employment programmes tend to use combinations of first‐come/first‐served and merit‐based recruitment, which may lead to more disadvantaged young people not receiving a place.
▪ **Partnership**: a number of summer employment programmes establish formal partnerships with schools, for example by actively involving school staff in the identification and recommendation of suitable candidates. Sponsorship and support from community action and workforce development agencies provide financial backing, resources, and expertise to engage and support young people.
▪ **Format**: in face‐to‐face summer education programmes, social activities and opportunities to support the formation of connections with peers plays an important role in facilitating young people's engagement, especially for those who face additional barriers to participation such as disabled students and those with health conditions.
▪ **Integration into the workplace**: some summer employment programmes facilitate pre‐placement engagement between young people and employers. This can take the form of orientation days, job fairs, or introductions during the enrolment process. These allow participants to familiarise themselves with the work environment, establish connections with employers, and gain insights into their future roles, fostering motivation and participation.
▪ **Skill acquisition**: in summer employment programmes, improvements in social skills, such as feeling able to ask for help and resolve conflicts with peers, are positively correlated with improved attendance.

### 16.2 Common to summer education and employment programmes

#### 16.2.1 Personalisation

*Summer education programmes* often have a lower student‐to‐staff ratio than standard school classes (Day et al., 2013a; Martin et al., 2013b; Maxwell et al., 2014). This enables individualised attention and support to be delivered to young people, which in turn can support student engagement in the summer programme. This mechanism is suggested in Barnett et al. (2012) specifically in relation to the use of a single tutor participating in lessons alongside students in small‐sized classes, or multiple tutors roaming between a small number of classes; and in Gorard et al. (2017), who observes that there were multiple adults in each class, and every child was seen receiving extra attention and time from the staff; although these studies do not detect impact.

Having programme staff who are trained to understand the unique circumstances and barriers faced by young people can also act as a motivator for young people and facilitate their participation in the programmes. The Scholars Academy specifically targeted low‐income, first‐generation students and had a dedicated programme director who was highly regarded by participants, which Henson (2018) argued supported them to do well and fostered their engagement. This mechanism is also suggested in Anthony (2019) who suggests that the Excel State University summer bridge programme recruited faculty who understood the unique circumstances and barriers of first‐generation and economically disadvantaged students; although neither study detected impact.

As the primary focus of *summer employment programmes* is to provide work insight and experience for young people to improve their employability for future jobs, providers place a strong focus on ensuring a suitable match between participants and work placements, to maximise participation and mitigate the risk of disengagement. This personalised approach emphasises appropriate placement decisions that align with the participants' preferences and experiences. In one programme, providers conduct interviews with young people during enrolment to understand their work histories and interests and then broker suitable placements (Valentine et al., 2017). The One Summer Chicago programme finds that the majority of surveyed young people find their participation meaningful and fulfilling (One Summer Chicago, 2015). By matching participants with jobs that align to their interests and goals, the study authors suggest that the programme helps create a sense of purpose and relevance. When participants feel a strong connection between their work and aspirations, they are more likely to engage at work.

#### 16.2.2 Relationship with programme staff

In *summer education programmes*, the presence of well‐prepared staff who provide effective academic and socio‐emotional support, and who are approachable and open to questions and discussion, can help create a positive learning environment which fosters participation. Younger (2017) suggests that this mechanism may support increased participation, although there is no impact evaluation associated with this study. Positive relationships with staff are also highlighted as playing an important role in supporting participation and engagement within catch‐up programmes (Williamson et al., 2020). Similar views are expressed in Wathington et al. (2016) regarding the role of peer mentoring and counselling as a way of supporting integration; and Gorard et al. (2014) describes the use of a ‘student feedback corner’ where young people could openly share their views and concerns with staff at the end of each lesson; although both these studies do not detect impact.

In *summer employment programmes*, the positive relationships that staff – including programme staff and workplace supervisors – build with participants are also highlighted as likely to be playing a role in improving attendance and programme completion. Where participants in summer employment programmes report improved social skills, such as asking staff for help and support, this is positively correlated with improvements in attendance (Modestino & Paulsen, 2019c; Reich, 2018; Theodos et al., 2014, 2017). This suggests that where staff members foster the development of these social skills, attendance outcomes may improve. Additionally, factors such as gaining a mentor and increasing aspirations to attend the next phase of education also contribute to improved attendance (Modestino & Paulsen, 2019a). In some programmes, participants say they value the help, support, and guidance offered by supervisors during work placements (Sum, 2015). Positive relationships with supervisors are also valued by young people for finding appropriate and interesting tasks, discussing their career, and future education options, fostering a sense of reliability and accessibility (Reich, 2018).

Another factor which may play a role in sustaining engagement in some summer employment programmes is the involvement of job mentors who regularly visit participants' workplaces (Heller, 2014; Sum, 2015). The support received ranges from on‐the‐job assistance to coaching on future opportunities, as well as positive reinforcement to avoid negative behaviours, for example, those associated with street life. This support network is likely to foster a sense of belonging, empowerment, and personal growth, all of which contribute to increased engagement and active participation, as suggested by the evaluation of One Summer Chicago (2015).

#### 16.2.3 Financial and non‐financial incentives

In *summer education programmes* there are a range of incentives, which in UK‐based programmes mainly consist of accessibility measures, such as providing free transport and removing financial barriers by eliminating the costs associated with attending. A number of authors suggest that incentives of this type may lead to increased summer programme attendance, including Ferguson (2018); Siddiqui et al. (2014); and Torgerson et al. (2014); although there is no impact evaluation associated with Ferguson (2018), and Siddiqui et al. (2014) and Torgerson et al. (2014) do not detect impact. Meanwhile, US programmes include stipends (i.e., fixed regular sums of money) and perks, such as gift cards, raffles, and field trips which support engagement and programme completion (Ghazzawi et al., 2022). This mechanism leading to increased summer programme engagement and completion is also suggested in Barnett et al. (2012) and Wathington et al. (2016); although these studies do not detect impact.

The motivations expressed by the participants may also serve as facilitators to participation. Many participants join *summer employment programmes* because they want something productive to do over summer (Lansing et al., 2018). The desire for personal development, understanding of the employability and technical skills required by work, and the opportunity to improve future prospects may support engagement and active participation. Additionally, where summer employment programmes provide financial benefits for participants (such as income), this is often cited as a facilitator to engagement and participation, given the young person's desire to receive the incentive as well as affecting how the opportunity is valued within the young person's household (Modestino, 2019b; Sum, 2015).

#### 16.2.4 Recruitment and targeting approach

Recruitment strategies such as school open evenings, providing written invitations, alumni advocates, and offering incentives such as discount schemes may encourage young people's participation in *summer education programmes* (Day et al., 2013b).

Summer education programmes that specifically prioritise the identification and targeting of disadvantaged at the individual level, such as selecting those pupils eligible for Free School Meals – rather than targeting by area characteristics such as disadvantaged neighbourhoods, as well as those that make efforts to market the programmes to parents/carers in addition to young people, are more successful at engaging their target cohorts (Day et al., 2013b; Robles, 2018). This mechanism is also discussed by Henson (2018), although this study does not detect impact. Identifying and recruiting pupils with English as an Additional Language (EAL) is particularly difficult due to challenges in successfully engaging and gaining the support of their parents and carers (Martin et al., 2013a; Sharp, 2018). This mechanism is also discussed by Torgerson et al. (2014), although this study does not detect impact. Some summer education programmes also face challenges identifying and recruiting disadvantaged pupils due to difficulties in obtaining relevant pupil data from feeder schools (Martin et al., 2013b).

A common challenge to participation in many *summer employment programmes* is that they use combinations of first‐come/first‐served and merit‐based recruitment (e.g., with applications, interviews, and employer selection) (Heller & Kessler, 2017). These approaches, which essentially allow eligible young people and providers to perform the allocation themselves, may lead to more disadvantaged young people not receiving places (Heller & Kessler, 2017).

#### 16.2.5 Partnerships

A number of *summer employment programmes* establish formal partnerships with schools, for example by actively involving school staff in the identification and recommendation of suitable candidates (Theodos et al., 2014). This collaboration enhances the effectiveness of participant selection, as school staff bring valuable insights into students' skills, interests, and career aspirations. School staff distribute application forms and ensure their timely submission, fostering interest and enthusiasm among students. These collaborative efforts between programmes and schools may help secure participants' active involvement (Theodos et al., 2014).

Sponsorship and support from community action and workforce development agencies provide financial backing, resources, and expertise to engage and support young people (Sum, 2015). Multiple organisations, including community‐based providers and employers alongside local government, often come together in summer employment programmes, fostering cooperation and creating a network of support and opportunity for participants (Gelber et al., 2016; Lansing et al., 2018; Modestino & Paulsen, 2019a; Schwartz et al., 2021). These partnerships help establish the summer programmes' infrastructure, enabling better coordination and collaboration with employers and community partners over time (Theodos et al., 2014; Valentine et al., 2017).

Partnerships also extend beyond the summer employment programmes. In one *summer education programme*, the Summer Learning Journey, the providers' collaboration with school staff strengthens communication channels and creates a stronger connection between the programme and participants' academic environments, improving referral processes and warm handovers (Williamson et al., 2020). Beyond logistical support, partnerships can also have legal and systemic implications. Participation in summer programmes may yield positive outcomes within the legal system, functioning as a mitigating factor and leading to more favourable responses from system actors (Kessler et al., 2022). These partnerships between programmes and legal entities create opportunities for better outcomes for young participants.

In a number of summer programmes, engaging parents/carers through activities like cookery classes or family learning, and celebration events, may support their increased involvement and promote a positive home–school partnership, which may in turn support young people's sustained participation and engagement (Day et al., 2013b).

### 16.3 Unique to summer education programmes

#### 16.3.1 Format

The format of a summer education programme may play an important facilitating role for some target groups in enabling their participation in the programme. This is discussed in particular in studies of online summer education programmes, especially for students with care responsibilities, part‐time jobs, or other constraints on their availability, with their participation being facilitated through a flexible format. This mechanism leading to increased summer programme participation is suggested by Taylor (2022), although this study does not detect impact. Other barriers to young people's participation, including scheduling conflicts due to holiday plans or family commitments (Maxwell et al., 2014; Somers et al., 2015), in some instances might not be able to be overcome by the use of an online format.

Programmes that are delivered online can however pose a barrier to participation for students who lack access to reliable internet connections and IT resources, although the provision of laptops and IT support may mitigate, to some extent, this barrier, as suggested by Lynch and Kim (2017). However, this study does not detect impact across the outcomes previously examined. An additional challenge to sustained participation faced by online summer schools can be digital fatigue, particularly following the shift to online learning during the pandemic, which can make these online programmes less attractive to some students. This mechanism leading to decreased summer programme participation is suggested by Taylor (2022), although this study does not detect impact.

In face‐to‐face summer education programmes, social activities and opportunities to support the formation of connections may play a role in facilitating young people's engagement, especially for those who face additional barriers to participation such as disabled students and those with health conditions (Day et al., 2013b). This mechanism is suggested in Lei et al. (2020) in relation to the role of sessions about ‘play’, experiencing shared meals on campus, and social outings and opportunities for informal socialising, although there is no impact evaluation associated with this study.

### 16.4 Unique to summer employment programmes

#### 16.4.1 Integration into the workplace

Some summer employment programmes facilitate pre‐placement engagement between young people and employers. This can take the form of orientation days, job fairs, or introductions during the enrolment process. These allow participants to familiarise themselves with the work environment, establish connections with employers, and gain insights into their future roles, which may in turn foster motivation and participation (Valentine et al., 2017).

#### 16.4.2 Skill acquisition

Improvements in social skills, such as feeling able to ask for help and resolve conflicts with peers, are positively correlated with improved attendance (Modestino & Paulsen, 2019c). Similarly, where young people cannot access the specific support they want from co‐workers or supervisors, they may turn to each other, which may then supports their continued engagement with the programme, as suggested in Valentine et al. (2017).

## 17 CAUSAL PROCESSES FOR SUMMER PROGRAMMES

This section explores the causal processes leading from engagement in summer programmes to outcomes as observed in eligible studies included in the analysis of the impact of summer programmes. As such, we include only studies of summer programmes which are shown to be effective in achieving impacts by achieving at least one significant impact on any of the specific outcome measures examined.

While when examining the impacts of summer programmes across the outcome domains the impacts of allocation and of participation were treated separately, in this analysis these are referred to as impacts of participation interchangeably as we are interested in how engaging with the programmes, which allocation to the treatment group denotes, leads to the outcomes observed. It should be noted that there are unlikely to be perfect linear relationships between all mechanisms and outcomes; for example, the full impact of summer programme participation on pupil confidence measured by Martin et al. (2013a) cannot be entirely attributed to the positive relationships built between staff and young people. Instead, we report on the key mechanisms identified in the literature as affecting young people's outcomes and detail the studies identifying these mechanisms as key.

In the section on how the intervention might work, five key mechanisms (relationships with staff and peers; financial and non‐financial incentives; location; skills acquisition; partnerships) were, ex ante, identified as potential mechanisms through which summer programmes might affect outcomes across the domains of interest. Interrogating the literature provided support for the important role these play in certain instances, while other mechanisms also emerged as facilitators of and/or barriers to positive outcomes among disadvantaged young people. We examine first those that are common to both summer education and employment programmes, before then discussing in turn those unique to summer education and those unique to summer employment programmes. In Supporting Information: Appendix 4, we also spotlight the links between context, mechanism and outcome in specific cases where studies identified an impact, and it is clearest that participation in a summer programme of that type has an impact – namely, the impact of participation in a summer education programme on educational engagement/participation/enjoyment, English test scores, all test scores, completion of higher education and STEM‐related higher education outcomes.

### 17.1 Summary of findings

The causal processes identified in studies as leading from engagement in to outcomes from summer programmes (that are shown to achieve impact) include:

▪ **Skill acquisition**: skills including academic, employability, social, emotional, and life skills, resulting from participation in a summer programme, play an important role in supporting young people's outcomes in both summer education and employment programmes.
▪ **Relationship with peers**: summer education programmes that involve older students as mentors provide valuable support to the young person and helps build participants' networks in the new environment, facilitating the transition to the next stage of education, and leading towards further outcomes.
▪ **Relationships with staff**: the lower student‐to‐staff ratio in summer education programmes enables individualised attention and support to be delivered to young people that in turn promotes student engagement.
▪ **Location:** in transition programmes, emphasis on navigating new educational settings and creating familiar learning environments all contribute to the effectiveness of these programmes. In summer employment programmes, locating young people in an organisation for the job placement builds familiarity and confidence in work settings.
▪ **Creating links to ‘business as usual’:** in some summer education programmes, creating connections between the summer education programme and the students' learning at home (e.g., with worksheets, activities, reading materials) helps maintain continuity and reinforces learning.
▪ **Raising aspirations**: in employment programmes, when participants find purpose and meaning in their work, often facilitated through the provision of financial and/or non‐financial incentives, they are more likely to see the importance of education in achieving their life goals, leading to raised aspirations.
▪ **Repeat participation**: long‐term participation in a summer employment programme is also associated with larger positive impacts on academic performance and test taking. The effects tend to be more significant for second and third‐time participants, suggesting that the benefits may accumulate over multiple years. Note however that ascribing any causality to this mechanism is especially problematic given the issue of self‐selection by those who repeatedly participate in the summer employment programme.

### 17.2 Common to summer education and employment programmes

#### 17.2.1 Skill acquisition

The evidence highlights that skill acquisition, including academic, social, emotional, and life skills, resulting from participation in a summer programme plays an important role in supporting young people's outcomes in summer programmes.

*Summer education programmes* that combine a variety of social activities, such as sports, arts, and curricular activities delivered in a creative way, facilitate positive outcomes for young people given the holistic approach they take (Day et al., 2013b). By offering a blend of fun and educational experiences, they address the educational and psychosocial needs of young people. The majority of evidence for summer education programmes pertains to raising aspirations and transition support programmes, where the predominant focus is on social aspects of participation and enrichment. Martin et al. (2013b) highlights how students highly value these features, and particularly notes the significance of making new friends, participating in team‐building activities, experiencing shared social activities, and interacting with peers in the structured learning environments, to their outcomes. These social interactions provide opportunities to develop social skills, build confidence, and form new friendships, while also improving motivation to engage in education thereby facilitating transitions. Day et al. (2013b) also highlights that incorporating activities that support the development of independent learning skills is an important facilitator of planned outcomes. Summer education programmes that support students to learn independently help to develop their autonomy and preparation for future academic endeavours. Martin et al. (2013a), evaluating the same intervention as these studies, estimates significant impacts of participation in the summer programme on self‐reported measures of pupil confidence, school readiness, and socialisation.

Summer programmes focused on *raising aspirations and transition support*, in both the UK and US, often focus on the development of soft skills that can support transitions and educational progression. Alongside a focus on supporting students to develop socio‐emotional skills and confidence to support personal growth, this also includes the ability to make effective university applications, study skills, and life skills, particularly in programmes focused on higher education. Cohodes et al. (2022) and Robles (2018), that highlight the importance of soft skill development in supporting transitions and progression, estimate significant impacts on the likelihood of applying to, progressing to and completing higher education. These studies evaluate a summer education programme with a focus on progression within STEM, and find larger effect sizes than Wachen et al. (2018) that evaluates a transition support programme, or Cosentino et al. (2015) that evaluates a raising aspirations programme, do on the likelihood of progression across all subject areas. This might indicate that the specialist nature of the skills this programme develops amplifies its impact on supporting transitions and progression to higher education, intermediate outcomes on the way to completion. Additionally, some programmes, particularly those supporting transitions from primary to secondary school, have dedicated activities on building relationships, understanding identity, navigating new challenges, and challenging stereotypes. This might lead to increased engagement in school, raising attainment: Pyne et al. (2020) estimates a significant positive impact of summer programme participation on English test scores, for instance.

In *summer employment programmes* (evidence for which is only available from the US) a focus on skills acquisition and employability attribute development, alongside the job‐specific technical skills that the job placement provides, aims to instil transferable skills for the labour market, which should aid entry to employment and employment‐related outcomes. In these programmes the main skills and attributes of focus are communication, problem‐solving, work‐readiness, social and life skills (Gelber et al., 2016; Leos‐Urbel, 2014; Sum, 2015; Theodos et al., 2014; Valentine et al., 2017). Modestino and Paulsen (2019a) finds a significant impact of summer programme participation on self‐reported measures of job readiness. However, there is no clear evidence of summer employment leading to positive labour market outcomes (although these may appear over the longer‐term while the evaluations included in this review typically only consider relatively short term outcomes). If anything there is evidence of a negative impact on the likelihood of entering employment.

The opportunities summer employment programmes provide for young people to develop social and emotional skills (such as processing social information, managing thoughts and emotions, and setting and achieving goals), alongside participating in group discussions with co‐workers and meeting new people who can support their growth (Heller, 2014), are also soft skills necessary to facilitate the achievement of wider education, socio‐emotional and violence and offending outcomes. In one programme, the Youth Violence Prevention Funder Learning Collaborative summer employment programme, specifically targeting young people with a history of, or at risk of offending, there is a strategy of engaging participants in group talks and team problem‐solving activities to foster communication, social skills, and critical thinking – Sum (2015) finds some evidence of participation in the programme reducing engagement in violent, offending and/or anti‐social behaviour. Modestino (2019b), Heller (2014), Davis and Heller (2020) also estimate negative (i.e., beneficial) impacts on the likelihood of experiencing criminal justice outcomes from participation in the summer programme; Modestino and Paulsen (2019a) finds a positive impact on the likelihood of progressing to higher education, suggesting a diversionary effect through improved soft skills increasing the ability of summer employment participants to apply and progress to higher education.

#### 17.2.2 Relationship with peers

Where *summer education programmes*, particularly those focused on transition support, involve older students as mentors, this provides valuable support to the young person while making their transition; building participants' networks in the new environment facilitates the transition, leading towards further outcomes (Day et al., 2013b).

Interactions with peers also provides valuable learning opportunities in *summer employment programmes*, as highlighted by the Urban Alliance programme where, during a pre‐work training, an episode of confusion and frustration among teammates led to a spontaneous lesson on the importance of patience and helpfulness (Theodos et al., 2014), with Theodos et al. (2017) estimating positive impacts on soft skill comfort from participation in the programme.

#### 17.2.3 Personalised and positive relationships with staff

The lower student‐to‐staff ratio that *summer education programmes* often have (Day et al., 2013a; Martin et al., 2013b; Maxwell et al., 2014) enables individualised attention and support to be delivered to young people that in turn promotes student engagement. This also means that programme staff can better address each student's specific needs and thereby ensure participants' academic and personal growth. Martin et al. (2013a), who studies an intervention with a lower student‐to‐staff ratio than would be expected in BAU, finds a positive impact of participation in the summer education programme on indexes of pupil confidence, school readiness and socialisation, and Maxwell et al. (2014) finds a positive impact on participants enjoyment of reading and motivation to read. Receipt of mentoring and opportunities for leadership within summer programme activities further enhance the positive relationships between staff and students. The evidence highlights that these positive relationships foster a sense of competence and belonging, promoting positive learning experiences and increased engagement and integration into the college or school community. Wachen et al. (2018), who highlights the importance of the relationships between staff and students, finds positive impacts of programme participation on retention in and completion of higher education. One of the benefits of *transition support programmes*, in particular, is that these provide students with the chance to meet their new teachers before the academic year commences, with emphasis also placed on the social aspect of the relationship. This early interaction helps students build positive relationships with staff, creating a sense of belonging, increasing confidence and comfort, and easing the transition. In the early phases of the new academic year, staff utilise the rapport established in summer transition support programmes to individualise support and foster engagement in the BAU classroom – note the aforementioned impacts of programme participation found by Martin et al. (2013b) on indexes of pupil confidence, school readiness and socialisation, and of Wachen et al. (2018) on retention in and completion of higher education.

In *summer employment programmes*, the positive relationships staff – including programme staff and workplace supervisors – build with participants also play a vital role in supporting positive outcomes, such as improved well‐being. There is evidence of participants in summer employment programmes reporting improved social skills, such as asking staff for help and support. Furthermore, as detailed in the background section on how the intervention might work, adult relationships are also formed with employees in the employing organisation which, along with the employer's expectation of performance from the young person, builds responsibility, maturity and self‐esteem. Modestino (2019b) finds positive impacts from programme participation on socio‐emotional engagement.

#### 17.2.4 Location of the summer programme

In the case of *transition support programmes*, there is evidence showing that allowing students to familiarise themselves with the new campus environment, can alleviate anxiety and help them feel more comfortable, which in turn promotes the achievement of wider soft outcomes including measures of school readiness (Martin et al., 2013a). Immersive experiences on university campuses, access to school resources, emphasis on navigating the college environment, and creating familiar learning environments all contribute to the effectiveness of these programmes: the 5‐week University of North Carolina summer bridge programme provides students with experience in navigating the university campus, using the instructional technologies and accessing academic support services, with Wachen et al. (2018) estimating positive impacts on the likelihood of retention in and completion of college as a result of participation.

Location is also an important mechanism to the outcomes from *summer employment programmes*. Locating young people in an organisation for the job placement builds familiarity and confidence in this new setting. I also increases expectations for conduct in this adult environment, with Modestino and Paulsen (2019a) finding positive impacts of summer programme participation on job readiness.

### 17.3 Unique to summer education programmes

#### 17.3.1 Creating links to ‘business as usual’ learning over the summer

In some summer education programmes, it is highlighted that creating connections between the summer programme and the students' learning at home (through worksheets, activities, or recommended reading materials throughout the summer holidays) helps maintain continuity and reinforces learning (Day et al., 2013b).

### 17.4 Unique to summer employment programmes

#### 17.4.1 Improving prospects and aspirations

A further notable finding pertains to summer employment programmes' effect on education outcomes. When participants find purpose and meaning in their work, potentially further facilitated through the provision of financial and/or non‐financial incentives, they are more likely to see the importance of education in achieving their life goals (Leos‐Urbel, 2014; Modestino & Paulsen, 2019c). As detailed in the background section on how the intervention might work, Modestino (2019) identifies a mechanism through building aspiration, self‐belief, emotion control and a longer‐term work ambition. The summer job encourages young people to improve their engagement with education as a precursor to achieving newly found higher quality employment goals. The post‐participation survey results from One Summer Chicago reveal that 70% of participants recognised the importance of education in achieving their life goals. This suggests that the programme successfully instils the importance of gaining qualifications to building the career you want. By emphasising the connection between education and future aspirations, these programmes motivate participants to actively pursue further academic success. As previously mentioned, Modestino and Paulsen (2019a), who evaluates the similar Boston Summer Youth Employment Program, finds a positive impact of participation on the likelihood of progressing to higher education.

#### 17.4.2 Financial and non‐financial incentives

The financial incentives provided by summer employment programmes, as suggested in the background section on how the intervention might work, may also help to alleviate financial constraints on future education, increasing investment in human capital – Modestino and Paulsen (2019a) finds positive impacts of summer programme participation on the likelihood of progression to higher education.

#### 17.4.3 Repeat participation

Long‐term participation in a summer employment programme is also associated with larger positive impacts on academic performance and test taking. The effects tend to be more significant for second and third‐time participants, suggesting that the benefits may accumulate over multiple years – self‐selection may however play a role here, as motivated students who are more likely to achieve better outcomes are more likely to apply for additional years of participation (Schwartz et al., 2021). It should be noted here that ascribing any causality to this mechanism is especially problematic. While there may be a dosage effect whereby participating in the summer employment programme multiple times provides greater benefits, findings of a greater impact on outcomes because of this could be partly or largely a result of self‐selection. For instance, those who benefit the most from participation may select into re‐applying and repeatedly participating in the programme.

## 18 IMPLEMENTATION ISSUES

The evidence in this section spans that which finds impact with evidence that does not, since implementation issues may be encountered in either, that is informative for future programme design.

### 18.1 Summary of findings

A range of studies provide information on implementation of summer programmes that can support improved designed, and this includes:

▪ In summer education programmes, elements that positively support design include interactive and alternative learning, iterative and progressive content building, incorporating confidence building activities, careful lesson planning, and adequate teacher support. Barriers to effective design are insufficient or delayed funding for the programme, limited reach in targeting key groups, and inadequate allocation of teacher and pupil groups.
▪ When it comes to implementation, effective practice for summer education programmes includes clear programme delivery guidance and good governance mechanisms, alongside clear communications about funding arrangements, and involving key stakeholders in monitoring progress, to allow foresight on funding adequacy. Ensuring high‐quality delivery through a structured curriculum, and engaged mentors and teachers also plays a key role. Partnering with external organisations and drawing upon their expertise in curricular and enrichment activities, such as sports, arts, and drama, enhances the success of summer education programmes. Also key to successful implementation is integrating programme evaluation and the sharing of results. Challenges in implementation arise when there is insufficient planning and lead time, recruitment challenges leading to lower‐than‐anticipated enrolment numbers, variability in teaching quality, and inadequate briefing of participants to set expectations of the programme.
▪ In summer employment programmes, design strengths include the use of employer orientation materials and supervisor handbooks before programme inception, careful consideration of programme staff roles to facilitate successful working relationships between employers and participants, providing a wide range of job opportunities, and building a network of engaged employers. Design challenges arise when there is uncertainty over funding and budget agreements, variation in delivery and quality of training between providers, difficulty in recruiting employers to the programme, and large caseload size and challenges in caseload management.
▪ When it comes to implementation, strengths of summer employment programmes include effective job matching which places participants in roles that match their interests, supportive relationships with programme staff and supervisors, pre‐work training, and mitigating attrition. Weaknesses include insufficient staff support for the number of participants, and limited employer availability to provide placements at the right time.

### 18.2 Summer education programmes

#### 18.2.1 Design strengths and weaknesses

Building on the causal pathways and facilitators to participation and outcomes, some key design concepts can be gleaned for *summer education programmes*. Features of effective design include:

▪ Using approaches to learning that are interactive and/or differ from mainstream schooling to motivate students and promote their interest in learning activities (Cosentino et al., 2015; Sharp, 2018). This is particularly evident in catch‐up programmes (Lynch & Kim, 2017; Somers et al., 2015; Torgerson et al., 2014; Wathington et al., 2016).
▪ Building up content iteratively and progressively over the programme to support the learning pace and development of each student (CooperGibson, 2022; Day et al., 2013a; Williamson et al., 2020). This includes building on their existing attainment on starting programmes, as well as careful allocation to learning groups, and offering applied learning opportunities (Cosentino et al., 2015; Day et al., 2013b). Crucially this needs well trained staff.
▪ Incorporating activities to help students become familiar with new educational environments, providing additional study support and mentoring, and including activities to build networks, are effective in transition support programmes (Day et al., 2013a; Henson, 2018; Smith et al., 2013) as well as programmes aimed at raising aspirations through a residential component (Lei et al., 2020).
▪ Careful lesson planning and adequate teacher support, drawing on experts in developing lesson plans and providing teachers with clear aims, activities, and resources for sessions, alongside feedback systems to ensure consistent quality (Day et al., 2013b; Gorard et al., 2014; Gorard et al., 2017; Wachen et al., 2018).
▪ Collaboration with core curriculum providers, providers of transition destinations, young people themselves and their parents, to take full account of young people's situations and circumstances, to design well matched programmes and marketing schemes (CooperGibson, 2022; Day et al., 2013b; Sharp, 2018; Wachen et al., 2018).

Across summer education programmes, the evidence highlights the importance of designing in planned progress indicators and developing tools to measure these (Day et al., 2013b). Delivery organisations also need to carefully assess what they can provide ‘in‐house’ (based on their own strengths) and where partnerships are needed (Day et al., 2013b).

▪ The evidence also highlights design weaknesses and pitfalls to avoid. These involve a lack of consideration at the design phase of:
▪ how the funding scheme affects delivery organisations – the funding scheme may need to allow for upfront funding of programme costs for some providers. Providing this can enable the involvement of small community and not‐for‐profit organisations (CooperGibson, 2022);
▪ the effect of the recruitment approach – limiting programme reach to specific settings can potentially exclude a broader population who are eligible and would benefit (Anthony, 2019; Cosentino et al., 2015);
▪ consequences of self‐selection – this can result in higher‐achieving, eligible students enrolling, potentially limiting the programme's impact on the planned population (Williamson et al., 2020); and
▪ careful consideration regarding allocation of pupils and teachers to learning groups – grouping pupils based on prior academic achievement may not be the most effective option, depending on planned programme outcomes (Gorard et al., 2014; Siddiqui et al., 2014); mixing age groups might present a challenge to teachers in providing tailored instruction suited to a range of abilities (Gorard et al., 2017); and a misalignment between the education stage of the pupils that programme staff are assigned to compared to those they work with in BAU, might reduce the effectiveness of the teaching (Gorard et al., 2015).

In deciding whether to take forward summer education programmes and the designs that are more feasible, it is also relevant to consider cost‐effectiveness. Some evidence highlights that some programmes, particularly those centred on catch‐up, have high ongoing costs per pupil, compared to other catch‐up approaches delivered during the regular school year (Torgerson et al., 2014).

#### 18.2.2 Implementation strengths and weaknesses

Overall, the evidence from summer education programmes highlights some key themes that underpin successful implementation. Successful summer education programmes include clear programme delivery guidance and good governance, high quality academic instruction, mentoring support, and strong partnerships.

Schools participating in a UK programme, highlighted funding and guidance provided by the funding and administering authority, which in this case was the Department for Education (DfE), as key to successful implementation. Clear communications about funding arrangements and involving the school governors in monitoring progress ensured transparency and accountability. Insight from this programme also highlights the importance of ensuring school administrators are aware of the funding arrangements and level of funding, are effectively managing these resources, and keep school governors informed about how funding is utilised. This allows foresight on funding adequacy, which means additional resources can be sought where necessary. In this example, additional funds were raised through local businesses, use of volunteers, and in‐kind support (CooperGibson, 2022).

Another UK‐based transition support programme, the Future Foundations summer school programme, highlights the importance of ensuring high‐quality delivery through a structured curriculum, and engaged mentors and teachers. Regarding the latter, a key aspect of successful implementation is that in the third week of the Summer School, a changeover phase takes place where a new batch of teachers replaces the previous ones, which provides alternating teaching and rest periods to staff. It helps to ensure that those teaching the programme can bring their full energy, while allowing rest in between the standard academic terms. It also works to ensure that the Summer School maintains a high level of teaching quality. Before the changeover, a handover session is organised to allow new teachers to meet their colleagues, learn about the students, and understand the site's rhythms and routines. This enables a smooth transfer of information and ensures that the new teachers are well‐prepared to continue the programme effectively. Both batches of teachers receive the same training, ensuring consistency to help maintain the quality of instruction and ensure that all teachers are well‐prepared for their roles. A further key feature is that although the teachers changed, mentors and peer mentors remained the same. This consistency ensured some continuity for participants throughout the programme (Siddiqui et al., 2014).

The key role played by highly engaged mentors in facilitating successful delivery is also highlighted in other programmes. In one US‐based transition support programme, Scholars Academy, participants cite peer mentors as one of the best parts of the summer school. Peer mentors are described by participants as someone to relate to outside of their teachers and families who can guide them on which societies to join, and introduce them to other students, facilitating their successful transition into their first year of university (Henson, 2018).

Partnering with external organisations and drawing upon their expertise in curricular and enrichment activities, such as sports, arts, and drama, may enhance the success of summer education programmes. In particular, in effective transition support programmes such as the DfE Summer Schools, secondary schools engage with feeder primaries early on and co‐design programmes to ensure relevance and effectiveness for prospective participants. The DfE Summer Schools work particularly well where they offer curricular and enrichment activities with an emphasis on ‘fun’. This enables pupils to enjoy new experiences, build confidence, reinforce learning and develop positive patterns of behaviour. Additionally, embedding proactive measures to address students' concerns, such as bullying, or anxiety about making new friends, is highlighted as an aspect of successful delivery (Day et al., 2013b). Providing specific activities to help new bonds to form, alongside support and opportunities for pupils to mix with their peers and school staff, to become familiar with the expected behaviours and boundaries at secondary school, is suggested as fostering a safe and supportive environment which facilitates delivery.

Also key to successful implementation is integrating programme evaluation and the sharing of results. The UK DfE Summer Schools programme conducted a thorough review of implementation and delivery with staff, and shared the learnings and effectiveness information with stakeholders. The report identified key success factors to be built upon and improved in future planning, including: the provision of diverse and engaging learning activities; individual target‐setting and mentoring for disadvantaged pupils; open discussion of topics that might be causing pupils concern (e.g., bullying, transitioning to the next stage of education, and the unfamiliar school environment); involving older students to welcome incoming students; involving parents in the delivery of the summer school and organising a celebration event; and identifying and assessing students' strengths and weaknesses during the summer school, to inform planning for the school year. Integrating evaluation activities also supports in identifying transferable learning from the summer programme to other areas of the school, such as wider transition support programmes, curricula, and learner support, ensuring that the benefits extend beyond the summer period.

In contrast to the implementation strengths, the review also highlights challenges encountered in implementing the summer education programmes. In some cases, these are the opposites of the strengths identified above. The key weaknesses cover:

▪ insufficient planning and lead in time, particularly common in transition support programmes between primary to secondary education, which in some cases results in schools not being able to run the programmes (CooperGibson, 2022);
▪ recruitment challenges leading to lower‐than‐anticipated enrolment numbers. In the Future Foundations summer school programme, one site was abandoned as the delivery partner could not secure schools to collaborate with, which was problematic since schools took the lead on recruiting through parents/carers (Gorard et al., 2014). The Switch On Reading programme faced similar challenges, with potential reasons identified by evaluators as the summer programme lacking appeal to the target population, alternative summer activities already having been booked and concerns about being part of the programme evaluation (Gorard et al., 2017) – this though may be judged specific to the trial rather than implementation per se;
▪ variability in teaching quality and training – in the Future Foundations summer school programme example, the programme embedded a formal teaching approach which mimicked a school environment. However, teaching practices were poor and errors were noted in content in some classes. In parallel, teachers appeared to lack interest in delivering the provision (Siddiqui et al., 2014). In another programme, teachers, all of whom were certified, reported that they would have felt more prepared to deliver classes, if training had focused on the BELL programme curriculum, rather than wider instructional practices and pedagogy (Somers et al., 2015);
▪ inadequate briefing of participants to set expectations – in the Higher Horizons+ Unify residentials programme, participants said that they were supervised to a higher degree than expected and not given enough independent time, while also saying activities were not as varied as they had thought (Hayes et al., 2018); and
▪ variation in implementation – across different sites for large scale programmes, leading to differences in goals, strengths, and institutional resources that impacted the intervention effectiveness (Cosentino et al., 2015).

### 18.3 Summer employment programmes

#### 18.3.1 Design strengths and weaknesses

The evidence that meets the inclusion criteria for this review, which is all drawn from the US, highlights a number of key design features in successful summer employment programmes which support their implementation. These include:

▪ Use of employer orientation materials and supervisor handbooks, such as those used by the STEP‐UP programme – these resources are used by the public employment service to assist with recruitment, and to match participants with suitable positions, as well as providing guidance on payroll paperwork, and managing time reporting throughout the summer programme. These materials inform, engage, and prepare employers and supervisors, fostering successful work placements that feature structured work plans and measurable goals (Reich, 2018).
▪ Careful consideration of programme staff roles – staff act as intermediaries between participants and employers, facilitating successful working relationships. To support the recruitment of young people, the delivery organisation in the Urban Alliance programme, which provides pre‐placement training during the school year before the summer work placement, meets with school staff to explain the programme, gather information, and align expectations. Communication is then consistent throughout the year between these partners, strengthening the support system for participants (Theodos et al., 2014).
▪ A wide range of job opportunities – this increases the choice available to participants, promoting increased engagement. In many programmes, participants are employed by community action and workforce development agencies, non‐profit agencies, especially in childcare, day care services, healthcare, and social services (Sum, 2015).
▪ Building a network of engaged employers – the New York City Summer Youth Employment Program builds a network of employers which it seeks to engage with each year. As part of this, it reviews the programme with these engaged employers, to identify learnings and adapt accordingly. This continuity and focus on continuous improvement helps to sustain employer engagement, and employers feel they can rely on a source of talent for the summer period each year (Valentine et al., 2017).

Some summer employment programmes also exhibit design weaknesses, which impact the quality of delivery. This can include:

▪ Uncertainty over funding and budget agreements – this was observed in the New York City Summer Youth Employment Program. Where providers do not receive final funding commitments within good time before a programme begins, it makes it challenging to plan services effectively (Valentine et al., 2017).
▪ Variation in delivery and quality of training between providers – while some have certified teachers, college staff, or financial education advisers delivering educational services, others rely on seasonal staff who may lack experience. This disparity can impact the consistency and effectiveness of educational components within summer employment programmes (Valentine et al., 2017).
▪ Recruitment of employers – this is a problem, particularly where multiple providers deliver in a geographic area. Competition among providers to secure sufficient employer placements can result in difficulties securing enough suitable placements for all participants. This can limit the range of work experience available (Valentine et al., 2017).
▪ Caseload size and management – for the Urban Alliance programme, the ideal caseload size was considered to be around 30–35 participants. However, programme coordinators express a preference for smaller caseloads, as the workload can be overwhelming and exceed the contracted working hours (Theodos et al., 2014).

#### 18.3.2 Implementation strengths and weaknesses

In successful summer employment programmes, effective job matching, supportive relationships, pre‐work training, and mitigating attrition are all features of effective implementation.

In terms of job matching, insight from studies of One Summer Chicago highlights that matching young people to jobs that align with their interests and career goals enhances their overall experience of the summer employment programme, as they are more likely to be engaged and motivated in their work and work environment, and more likely to perform well (Lansing et al., 2018).

The quality of the summer work experience and the support provided by supervisors in the workplace are also important, as highlighted in the Youth Violence Prevention Funder Learning Collaborative summer employment programme. Positive relationships between supervisors and programme participants contributed to a valuable learning experience, a sense of contribution, and the development of soft skills. Supportive supervisors played a significant role, with the majority of participants in the programme evaluation study reporting that the programme supervisors had provided various types of help – ranging from positive assistance, including exploring new avenues for the future, to supporting the young person to avoiding negative behaviours by staying off the street (Sum, 2015).

Similarly, in the Urban Alliance programme coordinators played a key role in providing support and guidance to students outside of their placement, particularly for those requiring additional assistance or support. Successful relationships with programme coordinators and mentors were considered to lead to improved job performance among participants and overall better programme outcomes (Theodos et al., 2014). The STEP‐UP programme also highlighted that the quality of the summer employment programmes is closely related to the availability of job supervisors. In the Discover track, 128 people supervised interns, of which 29 worked with a single intern and 99 had multiple interns. There were 385 Achieve supervisors, of which 298 worked with a single intern and 87 had multiple interns. Approximately 93% of Achieve supervisors and 95% of Discover supervisors attended an orientation before the summer programme began (Reich, 2018).

Some summer employment programmes also exhibited implementation challenges. An important element in the implementation of the New York City Summer Youth Employment Program was the use of ‘monitors’, provider staff members that visited each work site weekly to check with young people and supervisors, to ensure participants' regular participation, safety, and well‐being. However, there were not sufficient monitors for the number of participants, especially at work sites employing a large number of participants. In the implementation study, young people reported having limited interactions with their monitors, and those mostly concerning timesheet collection, with some not having seen their monitors regularly at all (Valentine et al., 2017).

The review also highlighted that one programme, Urban Alliance, experienced challenges with employer availability at the planned commencement time, resulting in some young people starting their work placements later than anticipated. This had an effect on young people's overall experience and limited participants' engagement in meaningful work (Theodos et al., 2014). Urban Alliance also faced significant attrition at various stages of the programme, including between the application and the start of pre‐work training, which happened during the school year, and throughout the work placement. The reasons for attrition were not fully observed or predictable, but factors such as changes in training classes timetables, extracurricular activities which clashed with the training, and family issues were highlighted as likely to be influencing attrition. Urban Alliance programme coordinators also reported that some participants needed a lot of support and guidance from their mentors and, if matched successfully, they performed well in their jobs. However, participants who were matched with mentors who were unsupportive or too busy with other work were at higher risk of disengagement (Theodos et al., 2017).

## 19 DISCUSSION

Given the wide‐ranging scope of this review, we examine the impact of summer education and employment programmes across several outcomes and estimate an average effect size for 15 different outcome measures. For the majority of the specific outcomes considered, based on the identified literature, it is fairly clear that allocation to/participation in a summer programme has no impact.

Nonetheless, there are some outcomes where it is clearer that summer education programmes have a positive impact. These are: English and all forms of test scores; the completion of higher education; and STEM‐related higher education outcomes. The impacts on test scores are quite small, translating to an increase in English and overall Grade Point Average of 0.08 and 0.14 respectively. In marginal cases, this degree of impact might be sufficient to alter the higher education pathways available to an individual for instance, but on the whole, this is unlikely to affect an individual's longer‐term outcomes. An impact equating to summer education programme participants being 1.5 times more likely to complete higher education is, however, notable. Programmes that achieve an outcome of this magnitude may well generate a large enough social value to more than outweigh the costs associated with delivery.

While it is fairly clear that summer education programmes have positive impacts across some of the education outcomes evaluated, summer employment programmes largely do not appear to have clear positive impacts across the outcomes evaluated. Where beneficial impacts of summer employment programmes on criminal justice outcomes are identified, they are substantial. However, it is hard to be confident in providing an overall assertion regarding these outcomes given the volume of evidence available and, within this, the variation in findings. Additionally, summer employment programmes appear to potentially have a negative effect on entry to employment (although the overall finding is insignificant), and potentially a significant negative impact on entry to employment unrelated to the summer programme itself, with a potential ‘raising aspirations’ effect diverting participants away from work into higher education. In interpreting this, perhaps a view to longer‐term outcomes is helpful. Should raised aspiration lead to higher levels of attainment and continued engagement in education, this in turn will unlock improved employment and health and wellbeing outcomes, as well as reduced anti‐social and criminal outcomes, according to a number of sources (e.g., Bell et al., 2018; OECD, 2022; Raghupathi & Raghupathi, 2020).

It is also important to note that there are limitations in the body of evidence available, that affect the ability of this review to determine whether summer programmes have an impact on young people's outcomes across the domains of interest or not. Firstly, there are concerns about the quality of the research conducted. Regarding the literature assessing the effectiveness of summer programmes, nearly three‐quarters of the studies were categorised as low confidence in their ability to remove risks of bias. In particular, high levels of overall and differential attrition, often resulting from relatively low rates of take up of treatment among the treatment group, risked a potential for selection bias to affect the results, for a large number of studies. To advance our knowledge and understanding of whether summer programmes are effective and which outcomes they are able to achieve, a focus is needed on high quality evidence with study designs and methodologies that negate the potential for selection bias. Regarding the qualitative evidence provided by the literature, which is crucial to understand the structures and features of summer programmes that lead to the outcomes they achieve, over nine in ten studies included in the review were rated as being of low quality. This further highlights the need for improvements in the quality of qualitative evidence available, in particular through the use of dedicated, formalised process evaluations, rather than the informal gathering of process and qualitative information within impact evaluations.

In addition to improvements in quality, more evidence on the effect of summer programmes on a wider range of outcomes is required, so that conclusions can be drawn with more certainty. Across the majority of analyses performed in this review, the number of studies evaluating any particular outcome measure is relatively low. Furthermore, given the differences in effect often found between summer education programmes (as well as across the specific intervention types within these) and summer employment programmes, the number of studies required to accurately estimate the average effect size of each of these programme types is further increased. In particular, further evidence is required regarding the impact of: both summer programme types on health and socio‐emotional outcomes, as the limited evidence available indicates that summer programmes may have some significant impacts on these outcomes; summer education programmes on employment, and violence and offending outcomes, as these were only evaluated by studies of summer employment programmes; summer employment programmes on violence and offending outcomes, as the limited evidence available indicates that summer employment programmes may have some significant impacts on these outcomes, as well as on all outcomes in the UK context (as all of the summer employment programmes included in the review occurred in the USA); and both summer programme types across all outcome domains over the longer‐term, particularly for attainment measured through test scores, to better understand the persistence of any identified impacts.

This review identifies a range of structures and features of summer programmes that seemingly influence their effectiveness in achieving outcomes among disadvantaged and ‘at risk’ young people. For instance, differences in effectiveness between programmes that primarily target disadvantage at the area level and those that do not, and between those that have clear targeting criteria at the individual level and those that do not, emerge in the sub‐group analysis performed. As previously noted, given that the sample sizes available for the meta‐analysis are generally quite low, the findings from the sub‐group analysis are correlational at best, and any differences in effectiveness between sub‐groups as identified through the meta‐analysis may be the result of other confounding characteristics of the intervention/study. Nonetheless, should this finding point to a true difference in effectiveness between programmes with and without these characteristics, it would suggest that clear identification and targeting of disadvantaged individuals that might benefit most from the summer programme plays a significant role in determining the outcomes achieved by summer programmes. This perhaps also suggests that a relatively small‐scale intervention such as a summer programme may not be sufficient to help individuals overcome ‘structural’ barriers to positive outcomes, such as the quality and availability of education or employment opportunities in the individual's community (for discussion on the importance of structural barriers in shaping outcomes, see for instance Rodgers, 2022; Hong et al., 2022). Instead, interventions like summer programmes may be most effective when targeted at young people who, if their individual‐level barriers (for instance, poor academic performance) are reduced or removed through participation in the programme, would be able to achieve positive outcomes. This does not diminish the case for tackling structural barriers to positive outcomes, but rather acknowledges that larger‐scale, systems changes are required to overcome these. Therefore, tighter targeting of those individuals who might be able to benefit most from a summer programme, and where the summer programme has the potential to make a difference to their outcomes, will alter the average effectiveness of the programme, which in turn affects the case for investing in the intervention.

The findings from the review also suggest that summer programmes cannot be considered a ‘one size fits all’ intervention. The wide variation (including instances of significant difference on average effect size) in findings between the summer education programme clusters identified, might lead to the recommendation for future reviews of summer education programmes to focus their research on more specific types of summer education programmes. The ability to do this however is dependent on the extent to which the evidence base grows, as much of the sub‐group analysis (including that looking at differences in effectiveness by summer education programme type) was based on limited sample sizes. Furthermore, wide variation in the context, aims, participant population and features of the interventions surfaced by the review, affects the external validity of the findings of each study. Those organisations involved in the design and delivery of summer programmes need to give appropriate consideration to the circumstances of and challenges faced by the disadvantaged or ‘at risk’ groups which the summer programme is intended to support, so that the intervention's aims might be achieved. Where this is done, interventions are often more likely to be effective in achieving impact.

In discussion with the review's advisory group, we were challenged on whether we had considered the systematicity facets of the selected programmes – meaning how well the systems in these contexts worked to meet the needs of the young people targeted. We acknowledge this would make a useful line of enquiry but did not extract and summarise evidence with this aim in mind for the current review, and it was not possible to tackle this sufficiently well without fully revisiting the primary papers. It is therefore an evidence gap that future work could aim to fill.

The case for investing in summer programmes, in terms of social return on investment, might best be made based on impacts on the likelihood of completing higher education (summer education programmes) and on violence and offending outcomes (summer employment programmes), should the latter of these emerge consistently through further research. The per unit benefit of achieving/avoiding these outcomes as a result of summer programme participation are relatively high, especially when considering the effects on an individual's opportunities over the longer‐term, through gaining further qualifications or avoiding engagement/further engagement with the criminal justice system. Britton (2022) estimated a lifetime return to the exchequer of an individual attending higher education of £110,000 for men (equal to £115,050 in 2022) and £30,000 (equal to £31,377 in 2022) for women. While the sources of economic benefits will be largely similar (student loan repayments, increased income tax revenue, etc.), the social return from *completing* higher education will likely be even greater than those arising from *attending* higher education; the National Audit Office (2011) cited in GMCA (2022) estimated a cost per first time entrant to the criminal justice system of an under 18 year‐old in the year following the offence of £3152 (equal to £4151 in 2022) (GMCA, 2022). Indeed, in their rough comparisons of the benefit of the impact of the summer employment programmes to the costs of delivery, Modestino (2019b) and Davis and Heller (2020) suggest that the benefits of One Summer Chicago and Boston Summer Youth Employment Program are greater than the costs. In attempting to more formally assess the social value of summer programmes targeted at disadvantaged or ‘at risk’ young people using these (as well as other) outcomes that they may lead to, further research would be required to understand, for instance, the marginal social benefit of completing higher education among different disadvantaged groups and the impact of summer education and employment programmes on the likelihood of experiencing criminal justice outcomes in the UK context.

## CONTRIBUTIONS OF AUTHORS

Becci Newton (IES) had overall responsibility for the drafting of the review, with ultimate control of content decisions. Daniel Muir (IES) took on day‐to‐day management of the review, leading the contribution on technical synthesis of the meta‐analysis. Cristiana Orlando (IES) led the drafting of the narrative synthesis of the thematic analysis.

## DECLARATIONS OF INTEREST

The authors declare no conflicts of interest due to any present or past affiliations or other involvements in any organisation or entity with an interest in the review's findings.

## SOURCES OF SUPPORT

The review was funded by a grant provided by the Youth Endowment Fund and Youth Futures Foundation through the Campbell Collaboration. The funding organisations provided experts, alongside individuals from other organisations, to the review's advisory group.

## Supporting information

Supporting information.
