# Supplementary material for: Impact of summer programmes on the outcomes of disadvantaged or ‘at risk’ young people: A systematic review
Source: Campbell Syst Rev. 2024 Jun 13;20(2):e1406. doi: 10.1002/cl2.1406 (PMC11170337; doi:10.1002/cl2.1406)
Supplement: Supplementary file 1 — Supporting information. [file CL2-20-e1406-s001.docx]

Appendices

Appendix 1: Details of search strategy

The majority of searches took place in the second week of January 2023. After further consultation on which databases should be interrogated as part of the process of developing the review protocol, further searches were run in February and May 2023. Table 12 displays the details of the search strategy used for each of the databases interrogated for literature potentially relevant to the review.

Table 12: Details of search strategy

| Database | Search method | Search string used | Manual filters | Hits |
| --- | --- | --- | --- | --- |
| Scopus | Full search | TITLE-ABS-KEY ( ( “summer school*“ OR “summer learn*“ OR “summer education*“ OR “educational summer” OR “summer bridge” OR “summer employ*“ OR “summer work” OR “summer place*“ OR “summer job*“ OR “summer apprentice*“ OR “summer intern*“ OR “summer camp*“ OR “summer program*“ ) AND ( “youth” OR “young” OR “child*“ OR “student*“ OR “pupil*“ OR “teenage*“ OR “adolescen*“ OR “juvenile” ) AND ( “disadvantage*“ OR “vulnerab*“ OR “at risk” OR “at-risk” OR “marginalised” OR “marginalized” OR “youth offend*“ OR “young offend*“ OR “delinquent” OR “anti-social” ) ) AND ( LIMIT-TO ( PUBYEAR , 2022 ) OR LIMIT-TO ( PUBYEAR , 2021 ) OR LIMIT-TO ( PUBYEAR , 2020 ) OR LIMIT-TO ( PUBYEAR , 2019 ) OR LIMIT-TO ( PUBYEAR , 2018 ) OR LIMIT-TO ( PUBYEAR , 2017 ) OR LIMIT-TO ( PUBYEAR , 2016 ) OR LIMIT-TO ( PUBYEAR , 2015 ) OR LIMIT-TO ( PUBYEAR , 2014 ) OR LIMIT-TO ( PUBYEAR , 2013 ) OR LIMIT-TO ( PUBYEAR , 2012 ) ) | - | 165 |
| PsycINFO | Full search | (ab(summer school*) OR ab(summer learn*) OR ab(summer education*) OR ab(educational summer) OR ab(summer bridge) OR ab(summer employ*) OR ab(summer work) OR ab(summer place*) OR ab(summer job*) OR ab(summer apprentice*) OR ab(summer intern*) OR ab(summer camp*) OR ab(summer program*)) AND (ab(youth) OR ab(young) OR ab(child*) OR ab(student*) OR ab(pupil*) OR ab(teenage*) OR ab(adolescen*) OR ab(juvenile)) AND (ab(disadvantage*) OR ab(vulnerab*) OR ab(at risk) OR ab(at-risk) OR ab(marginalised) OR ab(marginalized) OR ab(youth offend*) OR ab(young offend*) OR ab(delinquent) OR ab(anti-social)) | Publication date from 2012 to 2022 | 270 |
| CDAS | Full search | AB ( ( “summer school*“ OR “summer learn*“ OR “summer education*“ OR “educational summer” OR “summer bridge” OR “summer employ*“ OR “summer work” OR “summer place*“ OR “summer job*“ OR “summer apprentice*“ OR “summer intern*“ OR “summer camp*“ OR “summer program*“) ) AND AB ( ( “youth” OR “young” OR “child*“ OR “student*“ OR “pupil*“ OR “teenage*“ OR “adolescen*“ OR “juvenile” ) ) AND AB ( ( “disadvantage*“ OR “vulnerab*“ OR “at risk” OR “at-risk” OR “marginalised” OR “marginalized” OR “youth offend*“ OR “young offend*“ OR “delinquent” OR “anti-social”) ) | Publication date from 2012 to 2022 | 19 |
| ERIC | Full search | abstract:(( “summer school*“ OR “summer learn*“ OR “summer education*“ OR “educational summer” OR “summer bridge” OR “summer employ*“ OR “summer work” OR “summer place*“ OR “summer job*“ OR “summer apprentice*“ OR “summer intern*“ OR “summer camp*“ OR “summer program*“) AND (“youth” OR “young” OR “child*“ OR “student*“ OR “pupil*“ OR “teenage*“ OR “adolescen*“ OR “juvenile”) AND (“disadvantage*“ OR “vulnerab*“ OR “at risk” OR “at-risk” OR “marginalised” OR “marginalized” OR “youth offend*“ OR “young offend*“ OR “delinquent” OR “anti-social”)) pubyearmin:2012 | - | 71 |
| BEI | Full search | AB ( ( “summer school*“ OR “summer learn*“ OR “summer education*“ OR “educational summer” OR “summer bridge” OR “summer employ*“ OR “summer work” OR “summer place*“ OR “summer job*“ OR “summer apprentice*“ OR “summer intern*“ OR “summer camp*“ OR “summer program*“) ) AND AB ( ( “youth” OR “young” OR “child*“ OR “student*“ OR “pupil*“ OR “teenage*“ OR “adolescen*“ OR “juvenile” ) ) AND AB ( ( “disadvantage*“ OR “vulnerab*“ OR “at risk” OR “at-risk” OR “marginalised” OR “marginalized” OR “youth offend*“ OR “young offend*“ OR “delinquent” OR “anti-social”) ) | Publication date from 2012 to 2022 | 12 |
| YFF EGM | Full search | “summer” | - | 15 |
| Pathways to Work Evidence Clearinghouse | Full search | “summer” | - | 8 |
| Clearinghouse for Labor Evaluation and Research | Full search | “summer” | - | 67 |
| OPRE, Administration for Children and Families | Full search | “summer” | - | 46 |
| MDRC | Full search | “summer” | Publication date since 2012 | 26 |
| Journal of Youth Studies | Full search | ( “summer school*“ OR “summer learn*“ OR “summer education*“ OR “educational summer” OR “summer bridge” OR “summer employ*“ OR “summer work” OR “summer place*“ OR “summer job*“ OR “summer apprentice*“ OR “summer intern*“ OR “summer camp*“ OR “summer program*“ ) AND ( “youth” OR “young” OR “child*“ OR “student*“ OR “pupil*“ OR “teenage*“ OR “adolescen*“ OR “juvenile” ) AND ( “disadvantage*“ OR “vulnerab*“ OR “at risk” OR “at-risk” OR “marginalised” OR “marginalized” OR “youth offend*“ OR “young offend*“ OR “delinquent” OR “anti-social” ) | Publication date from 2012 to 2022 | 6 |
| Youth & Society | Full search | ( “summer school*“ OR “summer learn*“ OR “summer education*“ OR “educational summer” OR “summer bridge” OR “summer employ*“ OR “summer work” OR “summer place*“ OR “summer job*“ OR “summer apprentice*“ OR “summer intern*“ OR “summer camp*“ OR “summer program*“ ) AND ( “youth” OR “young” OR “child*“ OR “student*“ OR “pupil*“ OR “teenage*“ OR “adolescen*“ OR “juvenile” ) AND ( “disadvantage*“ OR “vulnerab*“ OR “at risk” OR “at-risk” OR “marginalised” OR “marginalized” OR “youth offend*“ OR “young offend*“ OR “delinquent” OR “anti-social” ) | Publication date from 2012 to 2022 | 7 |
| IJAY | Full search | ( “summer school*“ OR “summer learn*“ OR “summer education*“ OR “educational summer” OR “summer bridge” OR “summer employ*“ OR “summer work” OR “summer place*“ OR “summer job*“ OR “summer apprentice*“ OR “summer intern*“ OR “summer camp*“ OR “summer program*“ ) AND ( “youth” OR “young” OR “child*“ OR “student*“ OR “pupil*“ OR “teenage*“ OR “adolescen*“ OR “juvenile” ) AND ( “disadvantage*“ OR “vulnerab*“ OR “at risk” OR “at-risk” OR “marginalised” OR “marginalized” OR “youth offend*“ OR “young offend*“ OR “delinquent” OR “anti-social” ) | Publication date from 2012 to 2022 | 2 |
| Journal of Social Policy | Full search | ( “summer school*“ OR “summer learn*“ OR “summer education*“ OR “educational summer” OR “summer bridge” OR “summer employ*“ OR “summer work” OR “summer place*“ OR “summer job*“ OR “summer apprentice*“ OR “summer intern*“ OR “summer camp*“ OR “summer program*“ ) AND ( “youth” OR “young” OR “child*“ OR “student*“ OR “pupil*“ OR “teenage*“ OR “adolescen*“ OR “juvenile” ) AND ( “disadvantage*“ OR “vulnerab*“ OR “at risk” OR “at-risk” OR “marginalised” OR “marginalized” OR “youth offend*“ OR “young offend*“ OR “delinquent” OR “anti-social” ) | Publication date from 2012 to 2022 | 0 |
| Youth | Full search | summer school* OR “summer learn*“ OR “summer education*“ OR “educational summer” OR “summer bridge” OR “summer employ*“ OR “summer work” OR “summer place*“ OR “summer job*“ OR “summer apprentice*“ OR “summer intern*“ OR “summer camp*“ OR “summer program*“ | - | 3 |
| Google scholar | Manually title appraise first 100 hits | “summer school*” OR “summer education*” OR “educational summer” OR “summer bridge” OR “summer employ*” OR “summer work” OR “summer place*” OR “summer job*” OR “summer intern*” OR “summer camp*” OR “summer program* | Publication date since 2012 | 17,100 |
| Open Grey | Manually title appraise first 100 hits | ( “summer school*“ OR “summer learn*“ OR “summer education*“ OR “educational summer” OR “summer bridge” OR “summer employ*“ OR “summer work” OR “summer place*“ OR “summer job*“ OR “summer apprentice*“ OR “summer intern*“ OR “summer camp*“ OR “summer program*“ ) AND ( “youth” OR “young” OR “child*“ OR “student*“ OR “pupil*“ OR “teenage*“ OR “adolescen*“ OR “juvenile” ) AND ( “disadvantage*“ OR “vulnerab*“ OR “at risk” OR “at-risk” OR “marginalised” OR “marginalized” OR “youth offend*“ OR “young offend*“ OR “delinquent” OR “anti-social” ) | - | 1,756 |
| gov.uk | Manually title appraise first 50 hits | Separate searches for “summer school*, “summer learn*“, “summer education*“, “educational summer”, “summer bridge”, “summer employ*“, “summer work”, “summer place*“, “summer job*“, “summer apprentice*“, “summer intern*“, “summer camp*“ and “summer program*“ | Research and Statistics content type, sorted by relevance | 12,791-172,816 |
| gov.scot | Manually title appraise first 50 hits | “summer” | In Statistics and Research | 28 |
| gov.wales | Manually title appraise first 50 hits | Separate searches for “summer school*, “summer learn*“, “summer education*“, “educational summer”, “summer bridge”, “summer employ*“, “summer work”, “summer place*“, “summer job*“, “summer apprentice*“, “summer intern*“, “summer camp*“ and “summer program*“ | - | 1,082-10,890 |
| northernireland.gov.uk | Manually title appraise first 50 hits | “summer” | Sorted by relevance | 60 |
| gov.ie | Manually title appraise first 50 hits | Separate searches for “summer school*, “summer learn*“ , “summer education*“, “educational summer”, “summer bridge”, “summer employ*“, “summer work”, “summer place*“, “summer job*“, “summer apprentice*“, “summer intern*“, “summer camp*“, “summer program*“ | Sorted by relevance | 0-71 |
| National Lottery Community Fund | Manually title appraise first 50 hits | “summer” | - | 0 |
| Care Leavers Association | Manually title appraise first 50 hits | “summer” | - | 10 |
| Children’s and Young People’s Centre for Justice | Manually title appraise first 50 hits | “summer” | Published since 2012 | 0 |
| JRF | Manually title appraise first 50 hits | “summer” | Article type = reports (vs blogs and news) | 32 |
| Centre on the Dynamics of Ethnicity | Manually title appraise first 50 hits | “summer” | - | 5 |
| Nuffield Foundation | Manually title appraise first 50 hits | “summer” | - | 76 |
| RSA | Manually title appraise first 50 hits | “summer” | - | 1 |
| Centrepoint | Manually title appraise first 50 hits | “summer” | - | 18 |
| Youth Employment UK | Manually title appraise first 50 hits | “summer” | - | 233 |
| Impetus | Manually title appraise first 50 hits | “summer” | - | 14 |
| Edge | Manually title appraise first 50 hits | “summer” | - | 18 |
| Education and Employers | Manually title appraise first 50 hits | “summer” | - | 4 |
| NFER | Manually title appraise first 50 hits | “summer” | - | 8 |
| EEF Toolkit (Summer schools) | Full search | - | - | 59 |
| Sutton Trust | Manually title appraise first 50 hits | “summer” | - | 12 |
| TASO | Full search | “summer” | - | 50 |
| TASO Toolkit (Summer schools) | Full search | - | - | 12 |
| RAND review (McCombs 2019) | Full search | - | - | 63 |
| NBER | Manually title appraise first 50 hits | Separate searches for “summer school*, “summer learn*“, “summer education*“, “educational summer”, “summer bridge”, “summer employ*“, “summer work”, “summer place*“, “summer job*“, “summer apprentice*“, “summer intern*“, “summer camp*“ and “summer program*“ | Publication date from 2012 to 2022, sorted by relevance | 4,216-16,526 |
| TRIP | Manually title appraise first 50 hits | Separate searches for “summer school*, “summer learn*“, “summer education*“, “educational summer”, “summer bridge”, “summer employ*“, “summer work”, “summer place*“, “summer job*“, “summer apprentice*“, “summer intern*“, “summer camp*“ and “summer program*“ | Ordered by relevance | 0-3,651 |

Source: IES, 2024

Appendix 2: Characteristics of included summer programmes/studies

Table 13 summarises the key details of the summer programmes/studies included in the study.

Table 13: Details of summer programmes/studies included in the review

| Programme | Study | Population | Location of and period covered by study | (Main) sample size | Features of the programme | Study methods/design | In meta-analysis |
| --- | --- | --- | --- | --- | --- | --- | --- |
| Aim High | Pyne (2020) | Rising 6th to 9th grade students from low SES backgrounds | San Francisco, California, US  2009-2018 | 7,908 | 5 week summer programme. Activities include instruction and project-based learning on core subjects (mathematics, humanities, and science), and a socio-emotional learning (SEL) curriculum. | Quant. (DID) | Yes |
| Aimhigher West Midlands UniConnect | Horton (2020) | Disadvantaged Year 13 students, living in areas with low HE participation rates | West Midlands, England, UK  2018-2020 | 1,366 | Two widening participation outreach programmes which include a summer school component lasting 20/30▒hours | Mixed methods process evaluation (including case study research) | No |
| “ | Burgess (2021) | 18–19-year-old learners from rural and urban deprived areas | West Midlands, England, UK  2017-2019 | 1,386 | “ | Quant. process evaluation | No |
| Bath Autism Summer School | Lei (2018) | Students aged 16-19 with an Autism Spectrum Disorder (ASD) diagnoses aiming to apply to university | University of Bath, England, UK  2013-2017 | 122 | Summer school involving 2 overnight stays at a university campus, with a curriculum delivered over 3 days. This included sessions on the nature of university life, stress reduction and wellbeing. Participants were supported by ‘student ambassadors’ | Mixed methods process evaluation (survey and qualitative feedback) | No |
| Boston Summer Youth Employment Program | Modestino (2019a) | Young people aged 14-24 living in highly disadvantaged urban areas | Boston, US  2015-2016 | 4,235 | 6 weeks voluntary work experience programme, with 20▒hours of job readiness training. Participants are placed into either a subsidised position or a job with a private-sector employer. Participants are paid the Massachusetts minimum wage | Quant. (RCT) | Yes |
| “ | Modestino (2019b) | Young people aged 14-24 living in highly disadvantaged urban areas | Boston, US  2015-2016 | 1,327 | “ | “ | Yes |
| “ | Modestino (2019c) | Young people aged 14-24 living in highly disadvantaged urban areas | Boston, US  2014-2017 | 2,249 | “ | “ | Yes |
| Boston Summer Youth Employment Program; New York City Summer Youth Employment Program; One Summer Chicago | Juffras (2016) | Young people aged 14-24 living in highly disadvantaged urban areas | Baltimore, Boston, Chicago, Detroit, Los Angeles, New York, San Francisco, Seattle, US  2015 | Missing | 5-7 weeks paid employment programmes, which include career readiness coaching or a social emotional learning (SEL) curriculum | Quant. review | No |
| Building Educated Leaders for Life | Somers (2015) | Students aged 10-14 performing one to two years below grade level | US  2012 | 919 | 5-week middle school summer catch-up programme. Includes academic instruction in mathematics and English language arts covering material from the prior school year, social and academic enrichment instruction, community time and service, field trips and guest speaker visits. | Quant. (RCT) | Yes |
| California State University (Los Angeles) Bridge Learning Community Model | McEvoy (2012) | College freshmen from low socioeconomic background, low-educated families and experiencing poor academic outcomes | Los Angeles, US  2000-2009 | 470 | 6 weeks voluntary summer bridge programme. Includes 5 academic core components and 4 student development components, with materials intentionally crossing disciplines. Designed to assist students moving through catch-up support and create connections among students, faculty and disciplines. | Quant. (logistic reg.) | Yes |
| DANCOP summer school | Church (2018) | Year 10 students who met NCOP (Nottinghamshire Collaborative Outreach Programme) or POLAR targeting criteria | University of Derby, England, UK  2018 | 33 | Two-night residential summer school aiming to increase participants’ knowledge of HE. Activities include information sessions on applying to university and the courses on offer, social activities, and experiencing what living in halls of residence is like. | Mixed methods process evaluation (pre and post survey, reflective logs) | No |
| Department for Education Summer Schools Programme | Day (2013a) | FSM eligible pupils and those experiencing care who are transitioning from primary to secondary school | UK  2012 | 877, 10 | Voluntary summer school programme lasting 1-2 weeks. Each school tailored the programme to the needs of the incoming Year 7 cohort, with the overall aim being to ensure pupils make a successful transition to secondary school. | Mixed methods (survey research, programme data and qual. fieldwork) | No |
| “ | Martin (2013b) | “ | England, UK  2012 -2013 | 1,597 | “ | Mixed methods (survey, case studies) | No |
| “ | Day (2013b) | “ | England, UK  2012 -2013 | N/A | “ | Qual. (practitioner guide) | No |
| “ | Martin (2013a) | “ | England, UK  2012-2013 | 19,629 | “ | Quant. (PSM) | No |
| “ | Sharp (2018) | “ | England, UK  Period not specified | 21,065 | “ | Mixed methods (surveys and case studies) | No |
| Department for Education Summer Schools Programme (Covid-19) | Cooper  Gibson Research (2022) | Schools determined which pupils would benefit the most. Focus was on pupils transitioning into Year 7, vulnerable children and those with SEN | England, UK  2021-2022 | 5,536 | 1–2-week summer school involving academic catch-up learning and enrichment activities. Aim is to make up for learning lost during the Covid-19 pandemic | Mixed methods (survey data, MI, interviews) | No |
| Discover Summer School | Torgerson (2014) | Pupils transitioning from Year 6 to Year 7 experiencing poor academic outcomes | London, England, UK  2013-2013 | 124 | 4-week voluntary summer school delivered by trained staff, teachers and volunteers. Participants attend literacy and poetry workshops, and enrichment activities including drama, sports, and trips around London. | Quant. (RCT) | Yes |
| Elevate Math summer programme | Snipes (2015) | Students aged 12-13 transitioning to the 8th grade experiencing poor academic outcomes in mathematics. | California, US  2014 | 477 | 19-day mathematics-focused educational programme. Involves 4 instructional modules in mathematics and geometry, as well as one hour a day of computer-based exercises reflecting topics covered that day. Participants also attend a field trip to a college/university and a college information night for students and their families. | Quant. (RCT – wait list design) | Yes |
| English Learner Summer School | Johnson (2020) | Rising 10th, 11th, or 12th graders living in an urban district, who had arrived in the US less than 3 years before the programme | California, US  2005-2016 | 40,651 | 5-week courses in English language arts, mathematics, science, and social science which met graduation requirements. Programme aims to provide ESOL students with opportunities to earn academic credits, develop English competence and build supportive communities. | Quant. (DDD) | Yes |
| Excel State University summer bridge programme | Anthony (2019) | Young people aged 16-20 from low SES background and low-education families | US  2013-2016 | 868 | summer bridge programme provided by the university. Students take two credit courses and one college success course. Aim is to help academically and socially integrate students into college life. | Quant. (non-equivalent control group design adjusting for baseline equiv.) | Yes |
| Future Foundations summer school programme | Gorard (2014) | Pupils aged 9 to 11 eligible for FSM, who have low literacy scores, English as an additional language or a special educational need, | England, UK  2013 | 303 | 4-week summer programme. Components include: two 75-minute numeracy and literacy lessons, delivered to small groups by teachers and mentors; a reading book; and a variety of art, sports and outdoor activities. | Mixed methods (RCT, observations and interviews) | Yes |
| “ | Gorard (2015) | Pupils in Year 5 and Year 6 living in urban areas who are eligible for free school meals and experiencing poor academic outcomes. | London and South East of England, UK  2013-2013 | 303 | “ | Mixed methods (RCT, observations and interviews) | No |
| “ | Siddiqui (2014) | Pupils transitioning from Year 5 to Year 6 who are from low socioeconomic background and live in urban areas | London, England, UK  2012-2012 | 160 | “ | Mixed methods (PSM; observations and interviews) | Yes |
| “ | Gorard (2017) | Disadvantaged pupils transitioning to secondary school | England, UK | Missing | Evaluated 7 literacy interventions for disadvantaged pupils transitioning to secondary school | Quant. process evaluation | No |
| Higher Achievement | Garcia (2020) | Young people aged 10-14 from ‘at risk’ communities and considered academically motivated | Washington, DC, US  2006-2012 | 951 | Year-round out-of-school programme taking place over the course of 4 years, with a 6-week summer component. The latter involves four academic classes and two electives. Participants also take part in field trips, academic competitions and university trips. | Quant. (RCT) | Yes |
| “ | Herrera (2013) | “ | “ | 952 | “ | “ | Yes |
| Higher Horizons+ Unify residentials | Hayes (2018) | Students with a range of disadvantage characteristics (including those with experience of care, with a disability, young carers) | Keele and Staffordshire Universities, England, UK  2017 | 105 | Two-night residential summer programmes providing participants with information, advice and guidance on the process of applying to a HE course, as well as a preview of what university life is like. Activities included seminars, campus tours, inspirational talks and social activities. | Mixed methods process evaluation (repeated measures survey) | No |
| Imperial College London summer school | Smith (2013) | Year 12 students who have an interest in medicine, attend schools in areas of deprivation, and either come from low-education families, low SES background, or receive FSM | England, UK  2008-2010 | 20 | One week summer school providing participants with experience of university education and the opportunity to interact with doctors and medical student mentors. Activities include theoretical and practical lectures, team building and skills exercises. | Mixed methods (survey) | No |
| ISL Summer School | Lawson (2019) | Year 10 students | Canterbury Christ Church University, England, UK  2018 | 146 | Summer school aimed to encourage progression into HE. Activities include engineering workshops, afternoon trips, sports events. | Mixed methods (repeated measures survey) | No |
| New York City Summer Youth Employment Program | Gelber (2016) | Young people aged 14-21 living in low-income urban communities in New York | New York, New York, US  2005-2013 | 294,100 | 7-week paid work experience programme administered by community-based organisations. Participants are placed into jobs at day-care centres, government agencies, hospitals, law firms, museums, and retail organisations. Participants also attend around 15▒hours of job readiness, career exploration and financial literacy workshops. | Quant. (RCT) | Yes |
| “ | Kessler (2022) | “ | New York, NY, US  2005-2010 | 163,447 | “ | “ | Yes |
| “ | Leos-Urbel (2014) |  | New York, NY, US  2007-2008 | 36,550 | “ | “ | Yes |
| “ | Schwartz (2021) | “ | New York, NY, US  2005-2009 | 134,366 | “ | “ | Yes |
| “ | Valentine (2017) | “ | New York, NY, US  2006-2016 | 264,075 | “ | “ | Yes |
| New York City Summer Youth Employment Program, One Summer Chicago | Heller (2017) |  | New York, NY; Chicago, Illinois, US | N/A | See evaluations of New York City Summer Youth Employment Program and One Summer Chicago for details | “ | No |
| No-MisMatch Program | Gehring (2018) | Underprepared college students at risk of dropping out | US  2002-2012 | 6,219 | 4-year mandatory education programme provided by and taking place at the University running the programme. Runs throughout the year, with a summer component. Participants attend summer preparation courses, extensive tutoring, and multiple advisor meetings. | Quant. (RDD) | Yes |
| Nottingham Potential summer school | Younger (2017) | Not reported | England, UK  2017 | 553 | Not reported | Quant. (survey) | No |
| One Summer Chicago | Davis (2020) | Young people aged 14-21 attending high schools in high violence neighbourhoods in Chicago | Chicago, Illinois, US  2012-2013 | 1,634 | 8-week summer employment programme at minimum wage. Young people are assigned a job mentor and spend 2 of the 5 daily hours in a social emotional learning (SEL) curriculum | Quant. (RCT) | Yes |
| “ | Heller (2014) | Young people aged 14-21 attending high schools in high violence neighbourhoods in Chicago | Chicago, Illinois, US  2012-2013 | 1,634 | “ | “ | Yes |
| “ | Heller (2022) | Young people aged 16-21 attending high schools in high violence neighbourhoods in Chicago | Chicago, Illinois, US  2015-2017 | 5,405 | “ | “ | Yes |
| “ | Lansing (2018) | Young people aged 14-24 attending high schools in high violence neighbourhoods in Chicago | Chicago, Illinois, US  2015-2016 | 113 | “ | Qual. (interviews with stakeholders, mentors and young people) | No |
| “ | One Summer Chicago (2015) | Young people aged 13-24 attending high schools in high violence neighbourhoods in Chicago | Chicago, Illinois, US | Not provided | “ | Mixed methods | No |
| Realising Opportunities | Lamont (2014) | Academically prepared Year 12 students attending schools in deprived areas, who are from low SES backgrounds or have experience of care | England, UK  2010-2012 | 126 | Two-year programme delivered by 12 universities, aiming to raise students’ aspirations to apply to university. Activities include residential experiences, subject taster events, an online study skills module, and online mentoring. | Process evaluation (repeated measures survey) | No |
| “ | Kettlewell (2014a) | “ | England, UK  2010-2012 | 97 | “ | “ | No |
| “ | Kettlewell (2014b) | “ | England, UK  2012-2013 | 194 | “ | Quant. (pre and post survey) | No |
| Robotics Summer Learning Program | Mac Iver (2019) | Students aged 10-13 attending school district in which most students are eligible for free or reduced-price lunch. Most are also part of an ethnic minority group | US  2012-2013 | 652 | 5-week summer programme providing middle school students additional OST focused on science, mathematics and robotics instruction. Aims to increase students’ achievement and develop their interest in STEM majors and careers. Students received robotics instruction for 2 hours-a-day and over the 5 weeks worked on developing their robots, they also engaged in city wide robotics competitions | Quant. (PSM/matching) | Yes |
| RWJF Summer Medical and Dental Education Program | Cosentino (2015) | Rising 10th and 11th Grade students from demographic groups that are underrepresented among doctors and dentists (for example ethnic minority and low SES background students) and have an interest in attending medical or dental school | US  2006-2013 | 2,864 | 6-week science enrichment programme. Components include science instruction, exposure to medical or dentistry practice through clinical experiences, lectures and workshops providing other relevant exposure and skills. | Mixed methods (document review, interviews, site visits; PSM) | Yes |
| Scholars Academy | Henson (2018) | First-time freshmen who were also first-generation college students | Tennessee, US  2016-2017 | 1,105 | Two-week educational programme held before the beginning of the semester to give students a preview of what to expect from university. Programme involves activities taking place in a classroom setting, social events, and meetings with peer mentors. Students pay a fee of less than $50 to participate. | Quant. (logistic reg.) | Yes |
| Sheffield Outreach & Access to Medicine Scheme | Thompson (2017) | Year 9-12 students form schools with low rates of progression to HE. Students are selected based on individual indicators of disadvantage and under-representation in HE | Sheffield, England, UK  2008-2012 | 24 | 5-year programme aiming to raise awareness of HE opportunities and increase attainment for medicine-related courses. Includes a 4-day residential summer programme which involves tailored one-to-one support through mock interviews and a work placement. | Qual. process evaluation (interviews) | No |
| STEM Enrichment Summer Bridge Program | Ghazzawi (2022) | Under-represented minority students in STEM degrees at the University of Houston | University of Houston, Texas, US  2013-2015 | 3,018 | 9-week summer bridge programme aiming to increase graduation rates in STEM fields for under-represented students. Activities include STEM courses, academic skills workshops, mentoring and social activities. Participants receive a $3,000 stipend to cover the cost of the course. | Quant. (PSM and logistic reg.) | Yes |
| STEM summer programmes | Cohodes (2022) | High achieving, rising high school seniors who have an interest in STEM subjects and are from background that are underrepresented in STEM | US  2014-2021 | 2,084 | Three educational programmes, of which two were residential and one was online. Programmes aim to increase access to STEM careers. Activities include STEM courses, videos and webinars; workshops with industry leaders, academics and admissions officers. | Quant. (RCT) | Yes |
| “ | Robles (2018) | “ | US  2015-2011 | 6,324 | 6-week educational programme taking place at the host university campus. Activities involve STEM courses, project-based classes, workshops with academics and industry leaders. Participants are also provided with advice on the college admission process. | Quant. (PSM) | Yes |
| STEP-UP | Reich (2018) | Young people aged 14-21 enrolled in Minneapolis Public schools, and experiencing barriers to employment (including young people from low-income families, young people with disabilities and young peoplefrom immigrant families) | Minneapolis, Minnesota, US  2015-2017 | 836 | Employment programme aiming to help young people explore diverse career interests, gain skills and make professional connections. Involves an internship component taking place during the summer, and work readiness training in the spring. There are two programmes (Achieve and Discover) which have the same key components but are adjusted based on participants’ age. | Quant. (PSM) | Yes |
| Summer Active Reading Programme | Maxwell (2014) | Pupils transitioning to secondary school who are experiencing poor academic outcomes | Yorkshire and Humberside, England, UK  2013-2013 | 182 | Educational programme mainly taking place during the summer before pupils transition to secondary school, with some activities taking place shortly before and after the summer term. The intervention involves volunteers gifting four book packs to children, and two events including creative and physical activities, and one-to-one reading with volunteers. | Quant. (RCT) | Yes |
| Summer Arts Colleges | Tarling (2012) | Young people aged 14-19 recently released from custody | UK  2007-2011 | 1,535 | 3–6-week education programme involving an arts-based curriculum in which literacy and numeracy skills are embedded, and employer visits. Aims to improve literacy and numeracy skills, facilitate transition into education, training and employment, and reduce re-offending. | Quant. (pre and post using MI/admin. data) | No |
| Summer Learning Journey | Williamson (2020) | Students in years 4-8 from schools serving predominantly low SES status communities | New Zealand  2017-2018 | 513 | Online education summer programme. Activities include creative writing, storytelling, poetry writing, expressing personal opinions; on which participants write reflective blogs. Participants then receive individualised feedback on each blog post. | Quant. (matching) | Yes |
| Summer Success Academy | Mariano (2013) | Students transitioning from elementary to middle school scoring less than a specified cut off on the 5th grade spring assessment in either English language arts or mathematics | New York, NY US  2005-2008 | 45,087 | Mandatory education programme for students experiencing poor academic outcomes. Involves 20 additional days of academic instruction in English language arts and mathematics | Quant. (RDD) | Yes |
| Sutton Trust Summer Schools | Hoare (2012) | Students at the end of Year 12 from a non-traditional HE background | UK | Not reported | Not reported | Quant. | No |
| Tenmarks | Lynch (2017) | Students in the 3rd to 9th grade living in high-poverty urban neighbourhoods | US | 263 | 10-week online mathematics programme. Components include mathematics curriculum materials based on participants’ skill level, text and video “hints” and digital games. Students also received weekly text messages encouraging them to log into the programme. | Quant. (RCT) | Yes |
| Texas developmental summer bridge programme | Barnett (2012) | High school seniors in need of catch-up in a subject offered by the summer programme | Texas, US  2011 - 2011 | 1,318 | 5-week summer education programme delivered by 8 colleges. Programmes varied in delivery and implementation, but they all involved accelerated developmental education, a college knowledge component, and academic support. Participants also receive a stipend. | Quant. (RCT) | Yes |
| “ | Wathington (2016) | High school seniors or recent high school graduates in need of catch-up | Texas, US  2009 - 2011 | 1,318 | 4-5 weeks developmental summer bridge programme for high school graduates in need of catch-up. Participants receive accelerated instruction in their area of need (mathematics, reading or writing), mentoring and counselling, and instruction on the soft skills required to be a successful college student. Those who successfully complete the programme also receive a stipend. | Quant. (RCT) | Yes |
| UEA outreach summer schools | Ferguson (2018) | Year 9-11 students, mainly from POLAR quintiles 1 and 2 areas | UK | 186 respondents to the survey | Summer school | Mixed methods process evaluation (pre and post survey, focus groups) | No |
| University of North Carolina summer bridge | Wachen (2018) | Recent high school graduates who are either first-generation college students or require additional college preparation | North Carolina, US  2008-2014 | 23,914 | 5–6-week intense summer residential programme requiring students to complete a college-level mathematics and English course with a GPA of 2.0 or higher. Programme also involves counselling sessions or monitored study time in the evening. | Quant. (PSM) | Yes |
| Urban Alliance | Theodos (2014) | High school seniors (aged 17-18) attending schools considered to have a high proportion of young people at risk of not connecting to further education or meaningful work | Washington, DC and Baltimore, Maryland, US  2011-2015 | Nearly 50 interviews | Employment programme taking place during the senior year of high school. Involves four primary components: pre-work training, coaching and mentoring, and alumni services which take place during term-time. The paid internship component is completed during the summer. | Qual. (process evaluation) | No |
| “ | Theodos (2017) | “ | “ | 1,062 | “ | Quant. (RCT) | Yes |
| Widening participation summer schools | Taylor (2022) | Pupils in Years 9-10 and in Year 12 or first year of pst-16 education, who either: live in a deprived area, are refugees/asylum seekers, are young carers, have a disability, come from a low-education background, are FSM eligible, are from a minority background | England, UK  2021-2022 | 802 | Education programme delivered by universities. Involves subject-specific lectures and workshops, and activities such as personal statement writing. | Quant. (RCT) | Yes |
| Youth Violence Prevention Funder Learning Collaborative summer employment programme | Sum (2015) | Young people aged 14-24 coming from disadvantaged homes or experiencing care | US  2012-2012 | 587 | Employment programme providing young people with a paid work experience, quality supervision, a well-designed learning plan, and development and mentoring activities | Quant. (DID) | Yes |

Source: IES, 2024

Table 14 lists the sub-groups which each summer programme/study falls under.

Table 14: Summer programme/study sub-groups

| Programme | Study | Region | Programme type | Cluster type | Types of disadvantage targeted | In whole vs in part | Study design quality (impact evaluations only) |
| --- | --- | --- | --- | --- | --- | --- | --- |
| Aim High | Pyne (2020) | US | Education | Raising aspirations | individual-based socioeconomic disadvantage; individual-based first-generation; individual-based specific needs | In whole | Low |
| Aimhigher West Midlands UniConnect | Burgess (2021) | UK | Education | Raising aspirations | area-based poor academic performance | In part | - |
| “ | Horton (2020) | UK | Education | Raising aspirations | “ | In part | - |
| Bath Autism Summer School | Lei (2018) | UK | Education | Raising aspirations | individual-based disability/SEN | In whole | - |
| Boston Summer Youth Employment Program | Modestino (2019a) | US | Employment | Workplace exposure | area-based socioeconomic disadvantage | In whole | High |
| “ | Modestino (2019b) | US | Employment | Workplace exposure | “ | In whole | High |
| “ | Modestino (2019c) | US | Employment | Workplace exposure | “ | In whole | High |
| Boston Summer Youth Employment Program; New York City Summer Youth Employment Program; One Summer Chicago | Juffras (2016) | US | Employment | Workplace exposure | area-based socioeconomic disadvantage; area-based experienced/at risk of criminal justice system | In whole | - |
| Building Educated Leaders For Life | Somers (2015) | US | Education | Catch-up | individual-based poor academic performance | In whole | High |
| California State University (Los Angeles) Bridge Learning Community Model | McEvoy (2012) | US | Education | Catch-up | individual-based poor academic performance; individual-based socioeconomic disadvantage; individual-based first-generation | In whole | Low |
| DANCOP summer school | Church (2018) | UK | Education | Raising aspirations | area-based poor academic performance | In whole | - |
| Department For Education Summer Schools Programme | Day (2013a) | UK | Education | Transition support | individual-based socioeconomic disadvantage; individual-based specific needs | In whole | - |
| “ | Day (2013b) | UK | Education | Transition support | “ | In whole | - |
| “ | Martin (2013a) | UK | Education | Transition support | “ | In whole | Moderate |
| “ | Martin (2013b) | UK | Education | Transition support | “ | In whole | - |
| “ | Sharp (2018) | UK | Education | Transition support | “ | In whole | - |
| Department For Education Summer Schools Programme (Covid-19) | Cooper Gibson (2022) | UK | Education | Transition support | individual-based poor academic performance; individual-based socioeconomic disadvantage; individual-based disability/SEN; individual-based specific needs | In whole | - |
| Discover Summer School | Torgerson (2014) | UK | Education | Catch-up | individual-based poor academic performance | In whole | High |
| Elevate Math summer programme | Snipes (2015) | US | Education | Catch-up | individual-based poor academic performance | In part | High |
| English Learner Summer School | Johnson (2020) | US | Education | Catch-up | individual-based ESOL; individual-based specific needs | In whole | Low |
| Excel State University summer bridge programme | Anthony (2019) | US | Education | Transition support | individual-based socioeconomic disadvantage; individual-based first-generation | In whole | Low |
| Future Foundations summer school programme | Gorard (2015) | UK | Education | Transition support | individual-based poor academic performance; individual-based socioeconomic disadvantage; individual-based ESOL; individual-based disability/SEN | In whole | - |
| “ | Siddiqui (2014) | UK | Education | Transition support | “ | In whole | Moderate |
| “ | Gorard (2014) | UK | Education | Transition support | “ | In whole | High |
| Higher Achievement | Garcia (2020) | US | Education | Raising aspirations | area-based socioeconomic disadvantage | In part | High |
| “ | Herrera (2013) | US | Education | Raising aspirations | “ | In part | High |
| Higher Horizons+ Unify Residentials | Hayes (2018) | UK | Education | Raising aspirations | area-based poor academic performance | In whole | - |
| Imperial College London summer school | Smith (2013) | UK | Education | Transition support | area-based socioeconomic disadvantage; individual-based socioeconomic disadvantage; individual-based first-generation; area-based poor academic performance | In whole | - |
| ISL Summer School | Lawson (2019) | UK | Education | Raising aspirations | area-based poor academic performance | In part | - |
| New York City Summer Youth Employment Program | Gelber (2016) | US | Employment | Workplace exposure | area-based socioeconomic disadvantage | In whole | High |
| “ | Kessler (2022) | US | Employment | Workplace exposure | “ | In whole | High |
| “ | Leos-Urbel (2014) | US | Employment | Workplace exposure | “ | In whole | High |
| “ | Schwartz (2021) | US | Employment | Workplace exposure | “ | In whole | High |
| “ | Valentine (2017) | US | Employment | Workplace exposure | “ | In whole | High |
| New York City Summer Youth Employment Program, One Summer Chicago | Heller (2017) | US | Employment | Workplace exposure | area-based socioeconomic disadvantage; area-based experienced/at risk of criminal justice system | In whole | - |
| No-Mismatch Program | Gehring (2018) | US | Education | Transition support | individual-based poor academic performance | In whole | High |
| Nottingham Potential summer school | Younger (2017) | UK | Education | Raising aspirations | area-based socioeconomic disadvantage; individual-based socioeconomic disadvantage; individual-based first-generation; area-based poor academic performance; individual-based specific needs | In whole | - |
| One Summer Chicago | One Summer Chicago (2015) | US | Employment | Workplace exposure | area-based socioeconomic disadvantage; area-based experienced/at risk of criminal justice system | In whole | - |
| “ | Davis (2020) | US | Employment | Workplace exposure | “ | In whole | High |
| “ | Heller (2014) | US | Employment | Workplace exposure | “ | In whole | High |
| “ | Heller (2022) | US | Employment | Workplace exposure | “ | In whole | High |
| “ | Lansing (2018) | US | Employment | Workplace exposure | “ | In whole | - |
| Realising Opportunities | Kettlewell (2014a) | UK | Education | Raising aspirations | area-based socioeconomic disadvantage; individual-based socioeconomic disadvantage; area-based poor academic performance; individual-based specific needs | In part | - |
| “ | Kettlewell (2014b) | UK | Education | Raising aspirations | “ | In part | - |
| “ | Lamont (2014) | UK | Education | Raising aspirations | “ | In part | - |
| Robotics Summer Learning Program | Mac Iver (2019) | US | Education | Raising aspirations | individual-based poor academic performance | In whole | Moderate |
| RWJF Summer Medical And Dental Education Program | Cosentino (2015) | US | Education | Raising aspirations | individual-based socioeconomic disadvantage; individual-based ethnic minority | In whole | High |
| Scholars Academy | Henson (2018) | US | Education | Transition support | individual-based socioeconomic disadvantage; individual-based first-generation | In whole | Low |
| Sheffield Outreach & Access to Medicine Scheme | Thompson (2017) | UK | Education | Raising aspirations | individual-based socioeconomic disadvantage; individual-based first-generation; area-based poor academic performance; individual-based ethnic minority; individual-based specific needs | In part | - |
| STEM Enrichment Summer Bridge Program | Ghazzawi (2022) | US | Education | Raising aspirations | individual-based poor academic performance; area-based poor academic performance; individual-based ethnic minority | In part | High |
| STEM summer programmes | Cohodes (2022) | US | Education | Raising aspirations | individual-based first-generation; area-based poor academic performance; individual-based ethnic minority | In part | High |
| “ | Robles (2018) | US | Education | Raising aspirations | “ | In whole | Moderate |
| STEP-UP | Reich (2018) | US | Employment | Raising aspirations | individual-based socioeconomic disadvantage; individual-based experienced/at risk of criminal justice system; individual-based ESOL; individual-based specific needs | In whole | Moderate |
| Summer Active Reading Programme | Maxwell (2014) | UK | Education | Transition support | area-based socioeconomic disadvantage; individual-based poor academic performance | In whole | High |
| Summer Arts Colleges | Tarling (2012) | UK | Education | Raising aspirations | individual-based experienced/at risk of criminal justice system | In whole | - |
| Summer Learning Journey | Williamson (2020) | New Zealand | Education | Catch-up | area-based socioeconomic disadvantage | In whole | Low |
| Summer Success Academy | Mariano (2013) | US | Education | Catch-up | individual-based poor academic performance | In whole | High |
| Sutton Trust Summer Schools | Hoare (2012) | UK | Education | Raising aspirations | individual-based socioeconomic disadvantage; individual-based first-generation; area-based poor academic performance | In whole | - |
| Switch-On Reading | Gorard (2017) | UK | Education | Transition support | individual-based poor academic performance; individual-based socioeconomic disadvantage; individual-based ESOL | In part | - |
| Tenmarks | Lynch (2017) | US | Education | Catch-up | area-based socioeconomic disadvantage | In whole | High |
| Texas Developmental summer bridge programme | Barnett (2012) | US | Education | Transition support | individual-based poor academic performance | In whole | - |
| “ | Wathington (2016) | US | Education | Transition support | “ | In whole | High |
| UEA outreach summer schools | Ferguson (2018) | UK | Education | Raising aspirations | area-based poor academic performance | In whole | - |
| University Of North Carolina summer bridge | Wachen (2018) | US | Education | Transition support | individual-based poor academic performance | In whole | Moderate |
| Urban Alliance | Theodos (2014) | US | Employment | Workplace exposure | area-based socioeconomic disadvantage | In whole | - |
| “ | Theodos (2017) | US | Employment | Workplace exposure | “ | In whole | High |
| Widening participation summer schools | Taylor (2022) | UK | Education | Raising aspirations | area-based socioeconomic disadvantage; individual-based socioeconomic disadvantage; individual-based first-generation; area-based poor academic performance; individual-based disability/SEN; individual-based ethnic minority; individual-based specific needs | In whole | High |
| Youth Violence Prevention Funder Learning Collaborative summer employment programme | Sum (2015) | US | Employment | Workplace exposure | area-based socioeconomic disadvantage; area-based experienced/at risk of criminal justice system | In whole | Low |

Source: IES, 2024

Appendix 3: Results from meta-analyses of violence and offending outcomes by specific crime type

Figure 25, Figure 26 and Figure 27 display the forest plots from the meta-analyses of the impact of participation in a summer programme on the likelihood of having an arrest, arraignment or conviction for violent, drug or property crimes or offences post-programme respectively. As all the interventions included in this analysis are summer employment programmes, no split is included by programme type.

Figure 25: Impact of summer employment programme participation on likelihood of having a violent crime arrest/arraignment/conviction post-programme


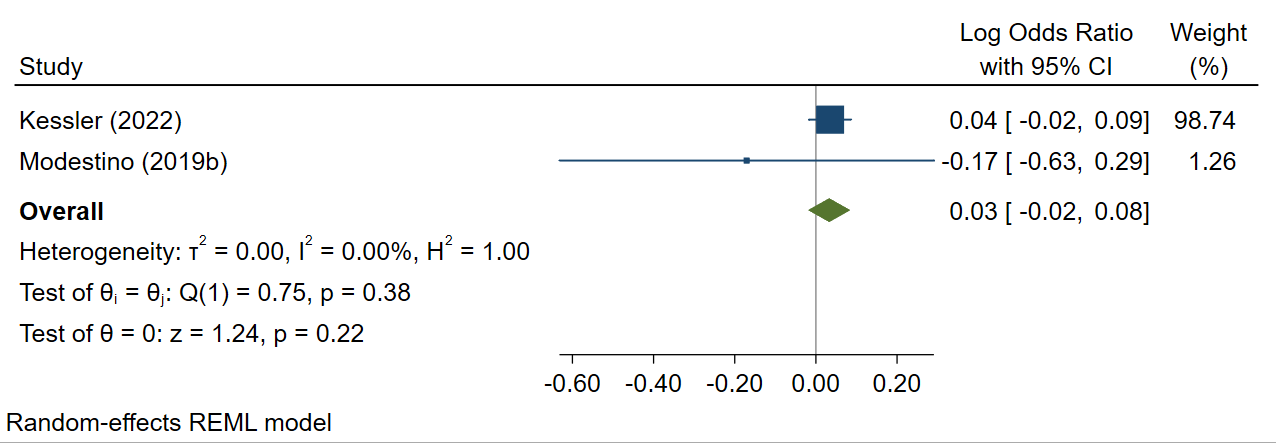


Source: IES, 2024

Figure 26: Impact of summer employment programme participation on likelihood of having a drug crime arrest/arraignment/conviction post-programme


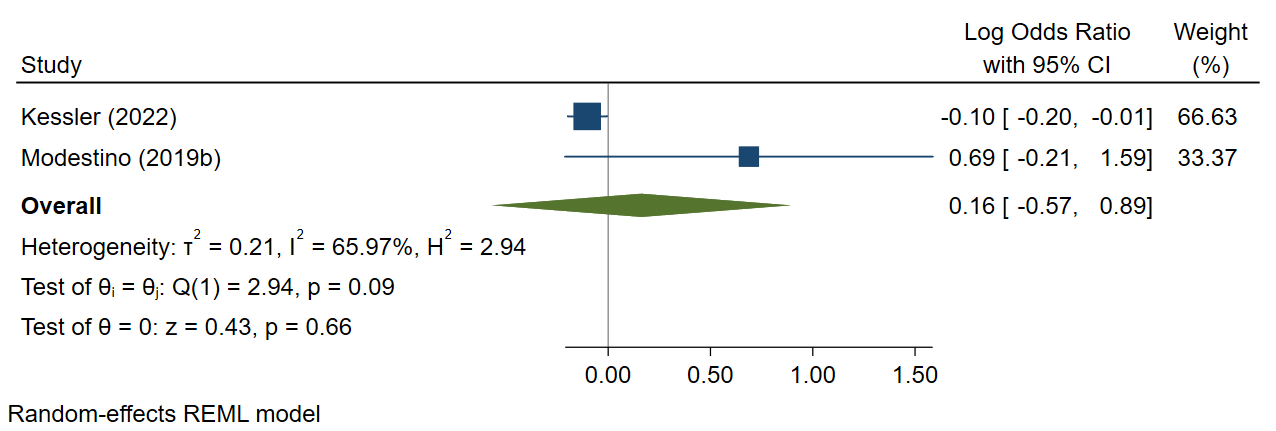


Source: IES, 2024

Figure 27: Impact of summer employment programme participation on likelihood of having a property crime arrest/arraignment/conviction post-programme


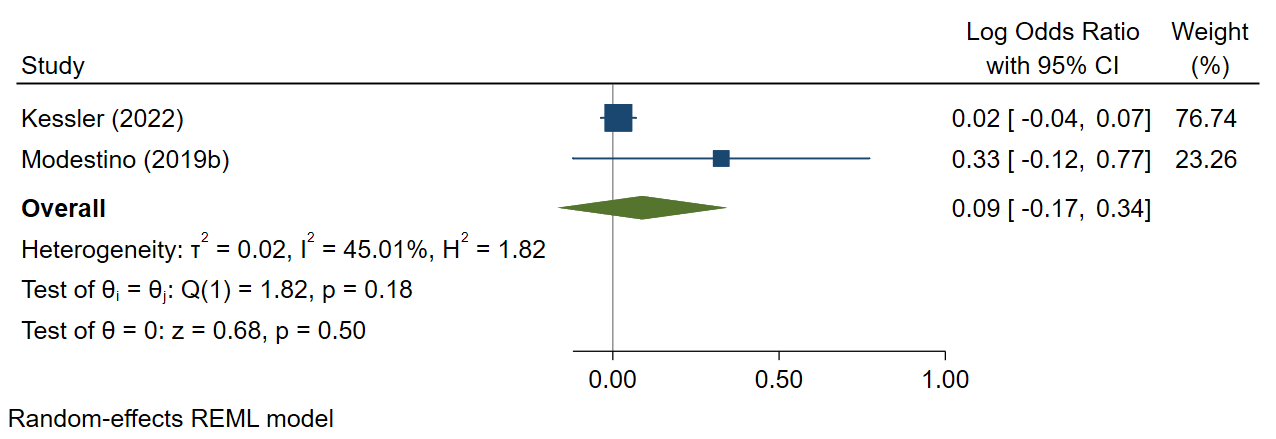


Source: IES, 2024

Figure 28, Figure 29 and Figure 30 display the forest plots from the meta-analyses of the impact of participation in a summer programme on the number of arrests or arraignments for violent, drug or property crimes or offences an individual has post-programme respectively. As all the interventions included in this analysis are summer employment programmes, no split is included by programme type.

Figure 28: Impact of summer employment programme participation on number of violent crime arrests/arraignments post-programme


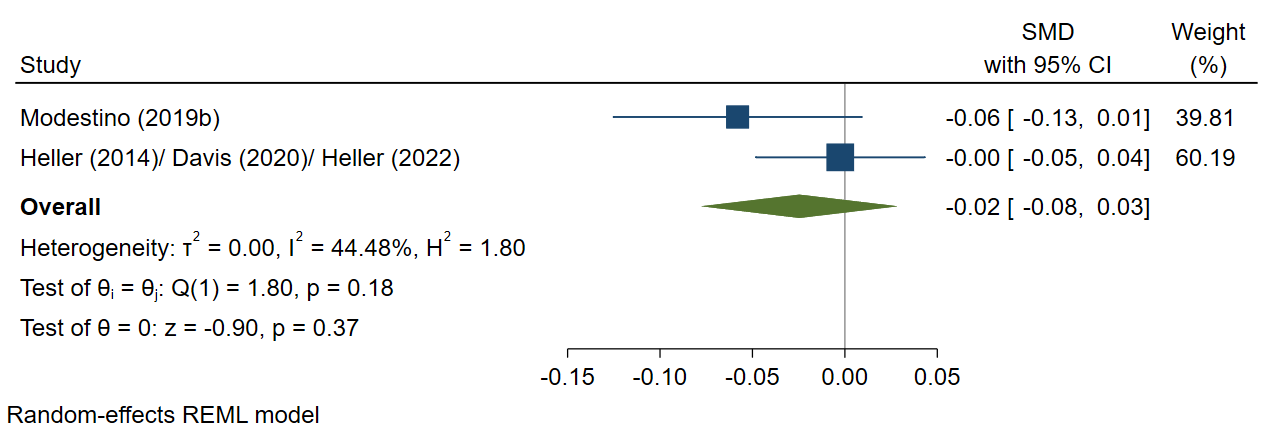


Source: IES, 2024

Figure 29: Impact of summer employment programme participation on number of drug crime arrests/arraignments post-programme


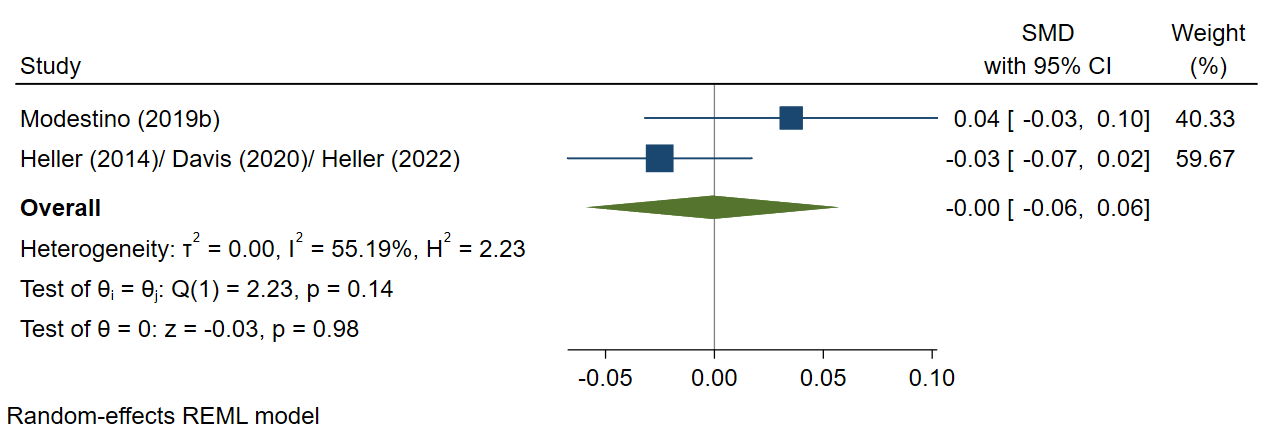


Source: IES, 2024

Figure 30: Impact of summer employment programme participation on number of property crime arrests/arraignments post-programme


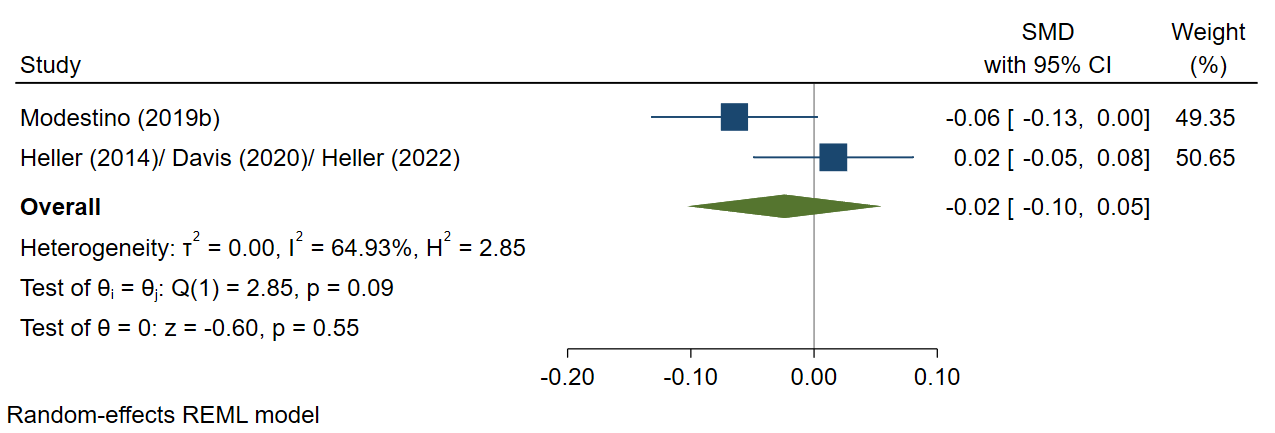


Source: IES, 2024

Appendix 4: Instances of impact across outcomes where summer programmes have the clearest effect

Herrera (2013) finds a positive impact of participation in Higher Achievement on educational engagement/participation/enjoyment. The intervention targets ‘at risk’ 10-14 year-olds, typically from low income families and/or ethnic minorities, who are deemed to be academically motivated. The six-week summer programme component Includes weekly field trips, academic competitions and a three-day out of town university trip, where students get to experience college life, alongside academic instruction. Exposing participants to these alternative forms of educational engagement might highlight to students who may not have had the opportunity to experience them before their value, leading to increased engagement in academic and/or enrichment activities outside of the summer programme and the BAU classroom.

Maxwell (2014) finds a positive impact of participation in the Summer Active Reading Programme on educational/participation/enjoyment. The intervention targets students in Year 6 i.e., those transitioning from primary to secondary education, that were identified by their teachers as not likely to achieve Level 4 in reading by the end of KS2, or likely to achieve a Level 4b or 4c. The intervention comprised two events during the summer which included creative and physical activities and a focus on one-to-one reading with programme staff, along with the gifting of four book packs to encourage further reading outside of the events, The combination of guided and (encouraged) independent reading might increase the suitability of the intervention to students with a wide range of learning styles, maximising the effectiveness of the programme in affecting participants’ enjoyment of and motivation to read.

Johnson (2020) finds a positive impact of participation in the English Learner Summer School on English (and therefore all forms of) test scores. The intervention, targeted at ESOL students enrolled in middle or high school that recently arrived in the US, provides five-week long courses with instruction five days-a-week, five hours-a-day in English language, mathematics, sciences or social sciences, all of which meet the district’s high school graduation requirements. The focus on a population of newly arrived immigrants who had developed academic literacy in their home language but had low levels of English proficiency that can be assumed to be keen to learn (having limited English proficiency due to a lack of exposure to the language as opposed to a lack of motivation or ability) might thus be a context whereby the increased and more intensive provision that a summer programme can offer leads to significant improvements in attainment.

Snipes (2015) finds a positive impact of participation in the Elevate Math summer programme on mathematics (and therefore all forms of) test scores. The programme evaluated targets 12-13 year-olds attending schools in areas with a high proportion of ESOL students, students eligible for free or reduced-price lunches, and students from ethnic minorities. Within this, students with relatively poor mathematics performance were intended to be targeted, although students of all abilities were admitted given that the number of applicants was less than the number of available places. The summer component, lasting 19 days, covered four instructional modules in mathematics plus an hour-a-day of online Khan Academy exercises reflecting topics covered in class that day, with laptops provided to the students to remove a potential barrier to engagement. Participants also attend a field trip to a local college or university as well as a college information night for students and their families to increase awareness of pathways to higher education. The additional, intensive subject-focussed instruction may have led to increased proficiency in mathematics resulting in higher test scores. Raised aspirations as a result of the sessions promoting higher education to students might also have increased effort and therefore attainment in the subject, although the mechanisms acting directly through the increased mathematics instruction is likely to have played the central role.

McEvoy (2012) finds a positive impact of participation in the California State University (Los Angeles) Bridge Learning Community Model programme on the likelihood of completing higher education. This intervention targeted first-year college students that are first-generation, are from low income backgrounds, are at risk of dropping out and/or are academically underprepared for higher education. The intensive 6-week, 5 days-a-week, 8 hours-a-day programme provided instruction on ‘core’ components relating to reading, writing, mathematics and study group skills, as well as student ‘development’ components relating to personal development, physical education, housing and mentoring. The curriculum and materials intentionally cut across courses and disciplines. The aim of the programme was to create an academically and socially supportive atmosphere, with the goals of building a sense of belonging and increasing essential academic skills. The focus on a holistic approach may have been key in supporting students transition to and retain in higher education, thus increasing the likelihood of college completion.

Wachen (2018) finds a positive impact of participation in the University of North Carolina summer bridge programme on the likelihood of completing higher education. The intervention targeted incoming first year college students that are first-generation or require ‘additional college preparation’ based on their SAT scores and high school GPA, and whether they were conditional admissions. The 5-6 week residential programme sought to avoid the programme being perceived as focussed on catch-up. Each day typically involved lengthy and intensive mathematics and English instruction through tutoring, support labs, mentoring and counselling. Social opportunities were also integrated into the programme to encourage the development of networks with faculty staff and peers, and students were monitored through the first term to ensure that they were fully integrated into the university. The intensity of the instruction may have to lead to a closing of the attainment gap with peers (students were required to take a pre- and post-programme test that measured skills levels relating to English and mathematics which might indicate whether participants’ proficiency levels improved across the programme, although the results of these are not reported on), whilst the development of social networks may have supported integration into the college environment, improving retention and increasing the likelihood of completing higher education.

Cohodes (2022) finds positive impacts of participation in the STEM summer programmes on the likelihood of completing higher education and of graduating with a STEM degree, and Robles (2018) who also evaluates one of the strands of the same intervention in a different cohort also finds positive impacts on the same outcomes. The STEM summer programmes they study were targeted at high achieving 16/17-year olds with an interest in STEM. Eligibility in terms of disadvantage was based on a range of factors including being a first-generation student, coming from a family with no STEM background, and coming from a academically poorly performing high school. The intervention aims to increase access to STEM careers through progression to higher education in STEM fields, by providing subject matter instruction through alternative provision including videos and webinars, as well as workshops with industry leaders, academics and admissions officers which aim to stimulate interest in progression within the field. Cohodes (2022) highlights that the increased knowledge about the college admissions process (the process for applying to the programme was designed to mimic the college application process) as well as the range of colleges available improves the effectiveness of participants’ college applications. Robles (2018) also highlights that participants are given evaluations from their instructors at the end of the programme to submit with their college applications, further supporting the young person’s transition to higher education in a STEM-related field and therefore the likelihood of graduating and graduating with a STEM-related degree.

Cosentino (2015) finds positive impacts of participation in the Summer Medical and Dental Education Programme on the likelihood of applying to medical or dental school, enrolling in medical or dental school and achieving a bachelor’s degree in a STEM field. On the latter of these, there is some evidence that this might reflect a diversion away from other degree areas, as they find a negative effect on the likelihood of obtaining a bachelor’s degree in a non-STEM subject or in a health-related or preparatory field (not considered as STEM), although neither of these effects are significant, and no significant impact on the overall proportion of participants obtaining a bachelor’s degree. Similar to Cohodes (2022) and Robles (2018), the intervention targets 15-17 year-old students with an interest in attending medical or dental school that come from demographic groups underrepresented in the field. The intervention provides STEM-related instruction as well as exposure to a medical or dentistry practice through clinical experiences, and lectures and workshops on the application process for medical or dental schools, financial planning and study skills. These features may increase the comfort of participants with the application process, as well as providing them the skills needed to make effective applications and, should they be successful, sustain in higher education, as well as further stimulating interest in the field, thus increasing the likelihood of graduating in a STEM field.

Ghazzawi (2022) finds a positive impact of participation in the STEM Enrichment Summer Bridge Program on the likelihood of graduating with a STEM degree from the initial field of study. Similar to Cohodes (2022), Robles (2018) and Cosentino (2015), the intervention also targeted individuals from demographic groups underrepresented in STEM fields. The intervention targeted individuals entering college that were, like Cohodes (2022), high performing students who attended low performing high schools, and, unlike Cohodes (2022), underperforming individuals – those admitted to a STEM focussed college after a special admissions review because they fell below the requirements for automatic admission. The summer programme covered content from first-year STEM courses with a degree of choice on the specific subjects, increasing subject knowledge as well as creating sense of comfort with the content and wider college life, supporting the student’s transition, increasing retention rates and thus the likelihood of graduation. Furthermore, throughout the following academic year participants are also engaged in additional roles including as office aides, tutors, peer mentors or peer facilitators as they advance through their academic careers to keep them engaged in the community, which might further support their retention leading to graduation.

Appendix 5: Mid-level Theory of Change

Figure 31 displays the mid-level theory of change which informed the review protocol (Muir 2023).

Figure 31: Mid-level Theory of Change


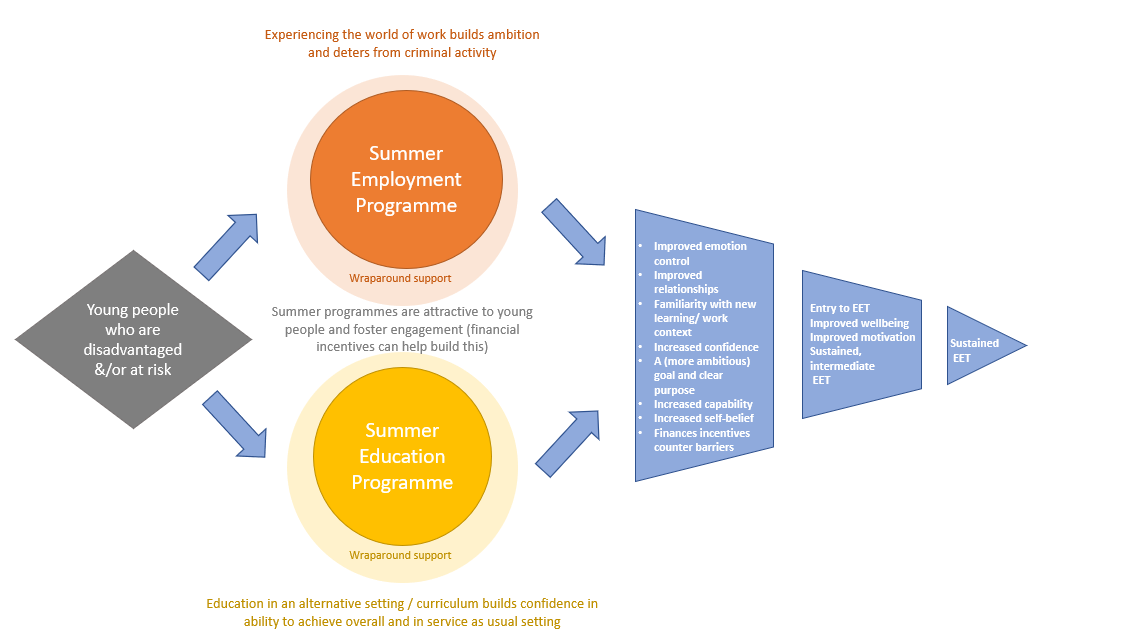


Source: IES, 2024
